# Carboxylic Acid Directed γ-Lactonization of Unactivated Primary C–H Bonds Catalyzed by Mn Complexes: Application to Stereoselective Natural Product Diversification

**DOI:** 10.1021/jacs.2c08620

**Published:** 2022-10-13

**Authors:** Arnau Call, Marco Cianfanelli, Pau Besalú-Sala, Giorgio Olivo, Andrea Palone, Laia Vicens, Xavi Ribas, Josep M. Luis, Massimo Bietti, Miquel Costas

**Affiliations:** † Institut de Química Computacional i Catàlisi (IQCC) and Departament de Química, Universitat de Girona, Campus Montilivi, Girona E-17003, Catalonia, Spain; ‡ Dipartimento di Scienze e Tecnologie Chimiche, Università “Tor Vergata”, Via della Ricerca Scientifica 1, I-00133 Rome, Italy

## Abstract

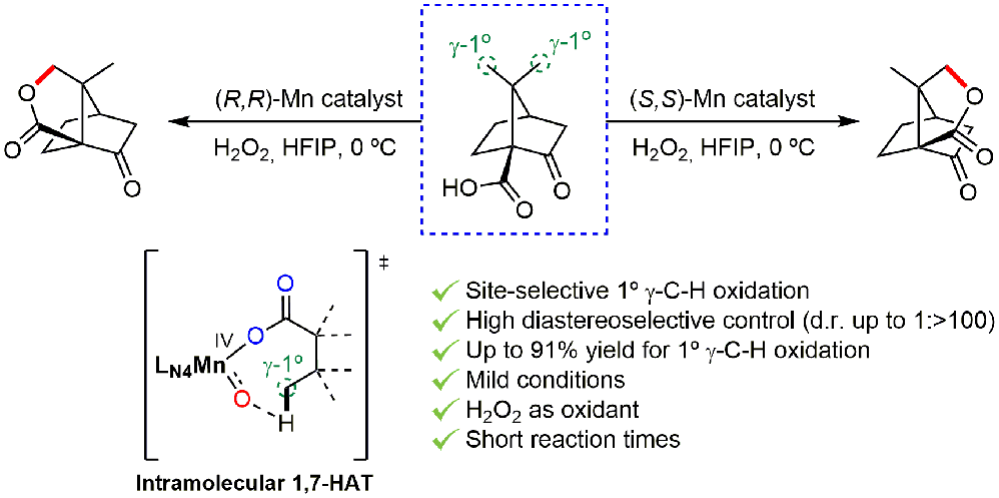

Reactions that enable selective functionalization of strong aliphatic C–H bonds open new synthetic paths to rapidly increase molecular complexity and expand chemical space. Particularly valuable are reactions where site-selectivity can be directed toward a specific C–H bond by catalyst control. Herein we describe the catalytic site- and stereoselective γ-lactonization of unactivated primary C–H bonds in carboxylic acid substrates. The system relies on a chiral Mn catalyst that activates aqueous hydrogen peroxide to promote intramolecular lactonization under mild conditions, via carboxylate binding to the metal center. The system exhibits high site-selectivity and enables the oxidation of unactivated primary γ-C–H bonds even in the presence of intrinsically weaker and a priori more reactive secondary and tertiary ones at α- and β-carbons. With substrates bearing nonequivalent γ-C–H bonds, the factors governing site-selectivity have been uncovered. Most remarkably, by manipulating the absolute chirality of the catalyst, γ-lactonization at methyl groups in *gem*-dimethyl structural units of rigid cyclic and bicyclic carboxylic acids can be achieved with unprecedented levels of diastereoselectivity. Such control has been successfully exploited in the late-stage lactonization of natural products such as camphoric, camphanic, ketopinic, and isoketopinic acids. DFT analysis points toward a rebound type mechanism initiated by intramolecular 1,7-HAT from a primary γ-C–H bond of the bound substrate to a highly reactive Mn^IV^-oxyl intermediate, to deliver a carbon radical that rapidly lactonizes through carboxylate transfer. Intramolecular kinetic deuterium isotope effect and ^18^O labeling experiments provide strong support to this mechanistic picture.

## Introduction

The selective functionalization of ubiquitous and unactivated C(sp^3^)–H bonds has become a powerful tool in modern organic chemistry because of the opportunities it offers to increase molecular complexity without the manipulation of pre-existing functional groups.^1^ Due to the high chemical versatility and the biological significance of oxygenated hydrocarbon skeletons, C–H oxygenation reactions are considered as particularly important transformations in the field.^2^

**Figure 1:**
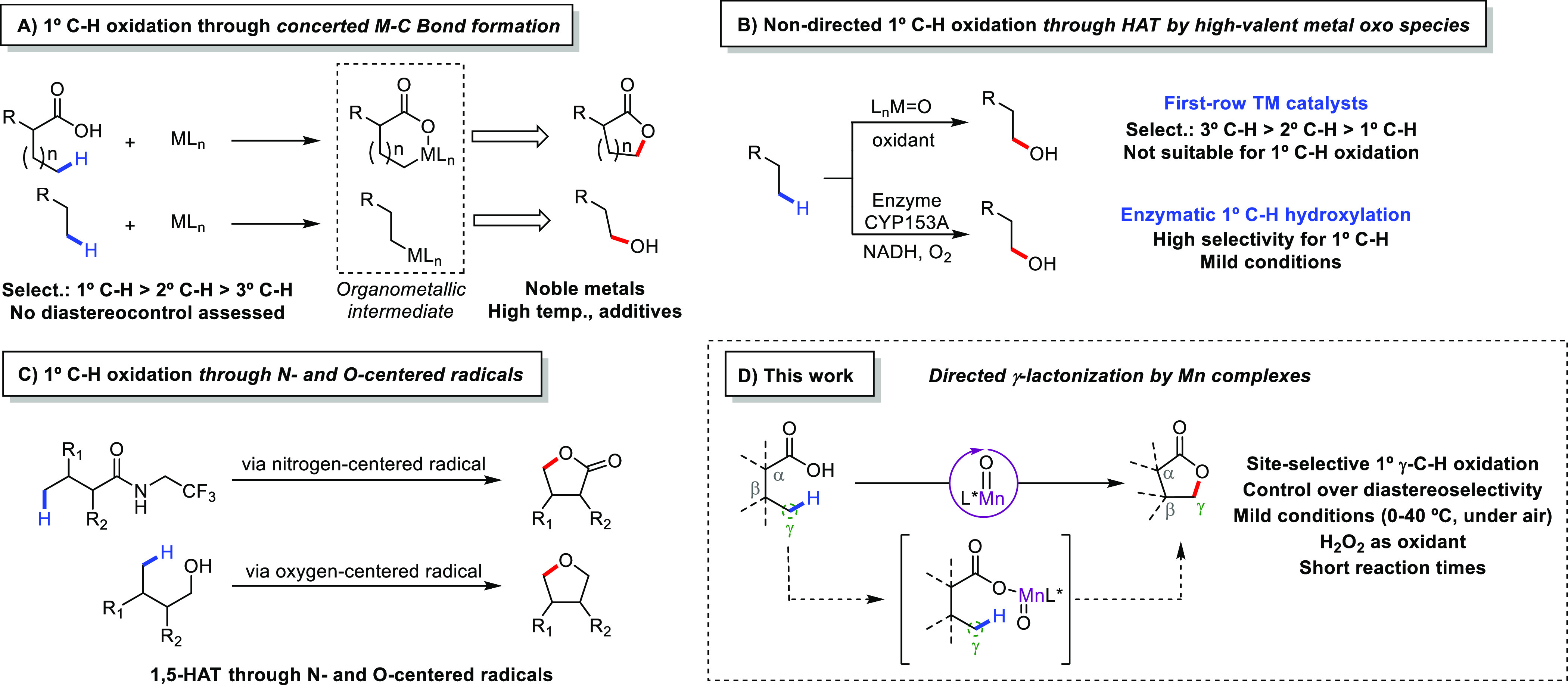
Summary of the existing strategies for the oxidation of unactivated primary C–H bonds. (A) General approaches for the directed and nondirected oxidation by organometallic intermediates. (B) Nondirected terminal oxidation by metal complexes and engineered enzymes. (C) Site-selective γ-functionalization via 1,5-HAT. (D) Summary of the main features of the present work.

The presence of multiple, nonequivalent C–H bonds in most organic molecules makes site-selective oxidation a largely sought and pursued challenging goal. A general consensus exists that C–H oxidation site-selectivity is intrinsically related to the nature of the reaction by which the C–H bond is cleaved. For instance, oxidations mediated by noble 5d and 6d transition metal-based catalysts (Pd, Ir, Pt) proceed via the formation of organometallic intermediates, favoring functionalization at terminal positions because of the strength of the corresponding metal–alkyl bond (Figure 1, A).^3^ In contrast, oxidations promoted by organic radicals and radical-like high-valent metal oxo species entail a hydrogen atom transfer (HAT) mechanism, for which homolytic cleavage of the weakest C–H bond to generate the corresponding alkyl radical predominantly governs selectivity (Figure 1, B). Accordingly, the relative reactivity of C–H bonds toward HAT generally decreases in the following order: 3 °C–H > 2 °C–H ≫ 1 °C–H, along the corresponding progression in C–H BDEs. It must be noted, however, that the selectivity exhibited by electrophilic oxidants against C–H bonds is not only dependent on the BDE of the latter, but also on a combination of electronic, steric, and stereoelectronic factors.^4^ In this regard, sterically hindered reagents have been designed to override relative C–H bond strengths to favor reactions at more accessible secondary over weaker tertiary sites. However, the notoriously high BDE of unactivated primary C–H bonds (>100 kcal·mol^–1^) renders their selective hydroxylation an extraordinary and rarely met challenge;^5^ selective oxidation of primary C–H bonds has been accomplished by making use of enzymes, optimized for the task by directed evolution,^6^ and by exploiting the reactivity of highly electrophilic *N*- and *O*-centered radicals in hydrogen atom transfer reactions (Figure 1, C).^2d,7,8^

An even more demanding and interesting but standing challenge is the selective oxidation of a single over multiple methyl groups present in the same substrate. Such a goal is particularly appealing because it may have broad synthetic relevance. Most notoriously, the *gem*-dimethyl moiety is a structural feature frequently found in many natural products of clinical interest,^9^ and currently the incorporation of the hydroxyl functionality into a single methyl group of such a moiety cannot be performed via direct C–H hydroxylation but leverages instead on the laborious manipulation of preexisting functionality, requiring several synthetic steps.^10^ However, given the relevance of this structural motif, it naturally follows that a reaction which enables stereoselective functionalization of a single methyl group will facilitate novel strategic disconnections that will streamline synthetic paths toward numerous complex natural products, significantly expanding the chemical space.

Since the first examples, reported more than 20 years ago, the field of C(sp^3^)–H oxidation catalyzed by bioinspired Fe and Mn complexes has grown exponentially.^11^ Nevertheless, although a deeper understanding of the factors that govern site-selectivity has allowed significant progress in predictability and the first examples of enantioselective oxidations,^12^ the tremendous potential of this reaction is far from being fully uncovered. General and synthetically reliable methodologies struggle to emerge since the interpretation of the complex selectivity patterns behind the reaction delays the development of a broad substrate scope. In this regard, by making use of simple carboxylic acid functionality as a native directing group, we recently developed an exceptionally γ-selective and enantioselective lactone-forming intramolecular oxidation mediated by tetradentate chiral N_2_Py_2_-type Mn complexes^12a,12b,13−15^ Such complexes promote the heterolytic cleavage of H_2_O_2_ to generate electrophilic high-valent metal-oxo species, which mimic the C–H hydroxylation via HAT/hydroxyl rebound mechanism displayed in nature by a variety of metalloenzymes.^11a,16,17^ The reaction operates satisfactorily on carboxylic acid substrates bearing several a priori oxidation sensitive functionalities, delivering chemically versatile γ-lactones. Such feature is also interesting because the carboxylic acid moiety is a common functionality in organic molecules, inter alia in several natural products. Furthermore, it can be easily introduced, tracelessly removed, or converted into a wide variety of functionalities, features that further expand the interest of carboxylic acid containing compounds.^18^

We reasoned that all of the above-mentioned challenges related to primary C–H bond oxidation could be potentially addressed by employing such a class of Mn catalysts. In addition, as the carboxylic moiety binds to the catalyst, such coordination will define a specific orientation of the substrate with respect to the reactive oxo ligand in the chiral catalyst, enabling stereoselective primary C–H bond oxidations in substrates bearing diastereotopic *gem*-dimethyl units. Initial support for this hypothesis has been obtained from the selective γ-C–H lactonization of *N*-phthalimido protected amino acids valine and *tert*-leucine.^12a^

Building on these precedents, herein we report a general method for the carboxylic acid directed γ-lactonization of unactivated primary aliphatic C–H bonds (Figure 1, D). The reactions occur with low loadings of a Mn catalyst, using hydrogen peroxide as the oxidant under mild conditions and short reaction times. The catalytic system enables site-selective lactonization of primary C–H bonds in the presence of secondary and tertiary ones. Most remarkably, catalyst chirality can be used to exert an unprecedented control over diastereoselectivity when oxidizing substrates containing a *gem*-dimethyl structural unit, and such control enables catalyst-chirality dependent selective oxidation of either of the two methyl groups in rigid camphor-derived carboxylic acids, opening novel paths for the elaboration of these important scaffolds. The work shows that the present system, based on an earth-abundant element catalyst, offers a unique tool for synthetic planning, orthogonal to the traditional synthetic disconnections, and therefore enabling powerful alternative retrosynthetic strategies.

## Results and Discussion

### Reaction Optimization

With the recently established methodology for secondary γ-C–H lactonization in hand,^12b^ we considered whether simple carboxylic acids could also enable the challenging oxidation of unactivated primary γ-C–H bonds. In order to address this question, 3,3-dimethylbutanoic acid (**1**) was selected as a model substrate, reasoning that on statistical grounds, the presence of 9 equiv primary γ-C–H bonds would facilitate the reaction (Table 1). In a typical experiment, 1 equiv of H_2_O_2_ was delivered over 30 min by syringe pump to a 2,2,2-trifluoroethanol (TFE) solution of the Mn(^TIPS^pdp) catalyst (1 mol %) and the substrate (25 mM) at 0 °C. The reaction mixture was further stirred for additional 15 min, quenched with 2-propanol, and products were analyzed by GC. Under these conditions γ-lactone (**1a**) resulting from oxidation at a methyl group was obtained as a single product in 44% yield (entry 1). After a first screening of the reaction conditions (Supporting Information, Table S1), it was found that increases in catalyst (2 mol %) and H_2_O_2_ (1.5 equiv) loading provided the best results in terms of product yield (64% isolated yield, entry 2). Despite substrate conversion is almost complete, GC and ^1^H NMR analysis showed only trace amounts of several side products that could not be identified. Products deriving from a decarboxylation pathway (alcohols, carbonyl compounds and alkenes) were not observed. As previously observed in secondary γ-C–H bond lactonization,^12b^ the Fe(pdp) catalyst delivered a lower yield than the corresponding Mn counterpart (entries 3 and 4). While the electron rich Mn(^DMM^pdp) catalyst failed in affording a higher product yield (entry 5).

**Table 1:**
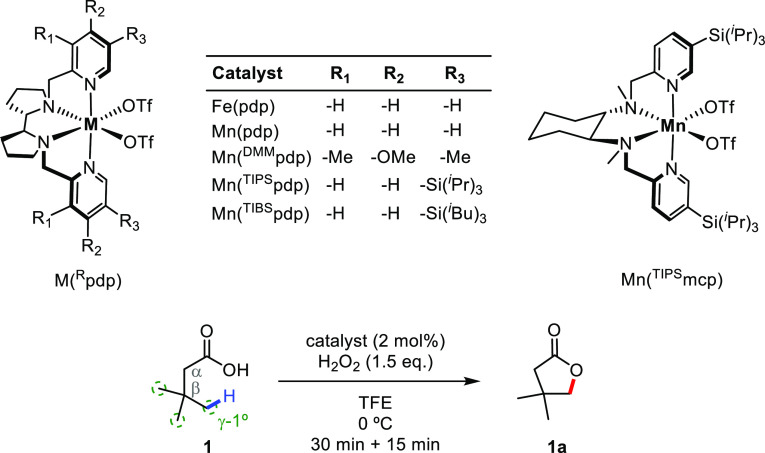
Optimization of Primary C–H Bond Lactonization in 3,3-Dimethylbutanoic Acid (**1**)^a^

entry	catalyst	variation	conversion (%)	yield (%)
1	Mn(^TIPS^pdp)^b^	–	75	44
2	Mn(^TIPS^pdp)	–	96	66 (64)^c^
3	Mn(pdp)	–	92	54
4	Fe(pdp)		82	25
5	Mn(^DMM^pdp)	–	81	28
6	Mn(^TIBS^pdp)	–	95	55
7	Mn(^TIPS^mcp)	–	71	41
8	Mn(^TIPS^pdp)	TfOH^d^	97	62
9	Mn(^TIPS^pdp)	HFIP^e^	98	64
10	Mn(^TIPS^pdp)	CH_3_CN^f^	57	11

a Reaction conditions: Substrate (25 mM), and (*S*,*S*)-catalyst (2 mol %) were dissolved in TFE. 1.5 equiv of H_2_O_2_ (0.9 M solution in TFE) were delivered over 30 min with a syringe pump, at 0 °C.

b Reaction performed using 1.0 equiv of H_2_O_2_ and 1 mol % catalyst. Conversions and yields determined by GC analysis using biphenyl as internal standard.

c Isolated yield in parentheses.

d TfOH (0.1 equiv) was added as a 0.09 M TFE solution over 30 min with a syringe pump.

e HFIP solvent.

f CH_3_CN solvent.

The nature of the chiral diamine backbone turned out to be important in determining catalyst activity. By employing the Mn(^TIPS^mcp) catalyst, where the chiral diamine backbone is a 1,2-cyclohexanediamine instead of bipyrrolidine, lactone yield substantially decreased (entry 7). The addition of triflic acid (TfOH), that was previously demonstrated to be crucial for lactonization at secondary C–H bonds,^12b^ was found to be not beneficial for this substrate (entry 8), suggesting that, under similar conditions, the limiting factor(s) of the yields for the catalytic oxidation of the two types of C–H bonds may diverge. Solvent variation to 1,1,1,3,3,3-hexafluoro-2-propanol (HFIP) did not alter reaction outcome (entry 9), while a significant drop in reaction efficiency was recorded by using MeCN (entry 10). The higher activities observed in fluorinated alcohol solvents are reasonably attributed to their substantial contribution to the H_2_O_2_ activation step as a result of their strong hydrogen bond donor character.^19^ Presumably, these solvents also determine an enhancement in the electrophilic character of the Mn-oxo species, which become more reactive HAT reagents.

### Investigation of the Factors That Affect Selectivity toward 1 °C–H Bonds

**Figure 2:**
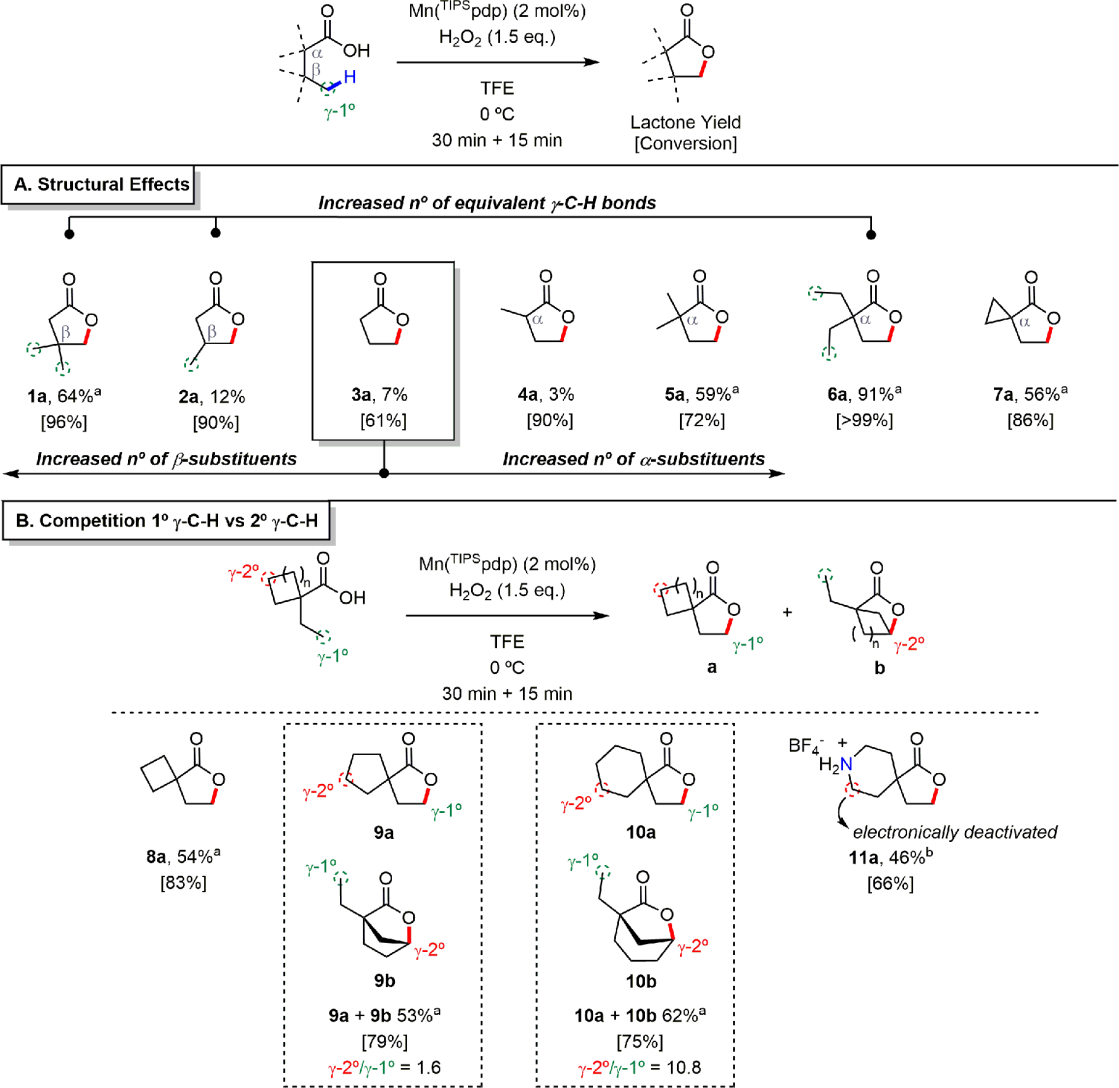
Substrate scope for primary γ-C–H bond lactonization. (A) Scope of butanoic acid derivatives for the rationalization of structural effects. (B) Intramolecular competition in primary vs secondary γ-C–H bond lactonization experiments. Conversions and yields were determined by GC analysis using internal standard calibration (see Supporting Information for details). ^*a*^Isolated yield. ^*b*^Yield determined by GC after product acetylation. The site-selectivity is expressed as the γ-2°/γ-1° ratio.

We subsequently explored the general applicability of the reaction to various types of substrates containing primary γ-C–H bonds. Toward this end, a systematic study was undertaken by investigating the reactivity of a series of butanoic acid derivatives (Figure 2, A). At first, we were interested in expanding the substrate scope toward analogues of **1** that bear a reduced number of equivalent primary C–H bonds. Oxidation of 3-methylbutanoic acid (**2**) and butanoic acid (**3**) delivered the corresponding γ-lactones in 12% and 7% yield, respectively, as the only detectable oxidation product, but high substrate consumption was observed. Side products, observed in trace amounts, were not identified. The decreased lactone yield observed upon sequential removal of β-methyl groups along the **1**, **2**, and **3** series is indicative of a strong dependence of product yield on the number of primary γ-C–H bonds and may suggest the existence of competitive paths for the oxidizing species whose relative contribution increases along the series. A similar outcome was observed in the oxidation of 2-methylbutanoic acid (**4**) that delivered γ-lactone **4a** in 3% yield. However, when the α-position was quaternized by introduction of an additional methyl groups as in 2,2-dimethylbutanoic acid (**5**), γ-reactivity was restored (59% isolated yield of γ-lactone **5a**), indicating that, besides the number of equivalent primary C–H bonds, angle compression associated with the operation of the Thorpe–Ingold effect, facilitates the reaction.^20^ Building on this finding, a series of α,α-disubstituted butanoic acids were examined. An outstanding 91% yield was obtained for lactone **6a**, as a result of the synergy between the Thorpe–Ingold effect and the presence of 9 equiv primary γ-C–H bonds in the parent substrate. In analogy, an α,α-cyclopropyl substituent showed the same beneficial effect allowing the formation of the interesting highly strained spirolactone **7a** in good isolated yield (56%). Along this line, sequential expansion of the flanking cycle to 4-, 5-, and 6-carbons (Figure 2, B, **8**–**10**) introduces within the substrates a competitive γ-methylenic site which a priori may be expected to be more reactive considering its lower BDE. Interestingly, in the oxidation of cyclobutanecarboxylic acid derivative **8**, exclusive lactonization at the exocyclic primary site was observed, providing lactone **8a** in 54% isolated yield, while leaving the cyclobutyl framework untouched, presumably because of substrate rigidity that makes the γ-methylenic site inaccessible to the active oxidant combined with the fact that cyclobutyl C–H bonds have increased *s*-character, which translates into stronger bond dissociation energies.^21^ However, the less strained architecture of cyclopentanecarboxylic (**9**) and cyclohexanecarboxylic acid (**10**) derivatives allows for a more favorable orientation of the methylenic γ-C–H bonds toward the oxidant, delivering bicyclic lactones **9b** and **10b** as the major product, progressively attenuating, with increasing ring size, competition for the primary site, quantified by the γ-2°/γ-1° product ratio.

Reasonably, the secondary γ-C–H bonds in the conformationally more rigid and flat cyclopentane ring of **9**, upon carboxylic acid binding, are not optimally directed toward the Mn-oxo unit, thus affording a mixture of primary and secondary C–H lactonization products (**9a** and **9b**) in comparable amounts (γ-2°/γ-1° = 1.6). On the other hand, the higher conformational flexibility of the cyclohexane ring in **10** makes the secondary γ-C–H bonds more accessible to the Mn-oxo and results in a drastic increase in site-selectivity for secondary C–H lactonization (γ-2°/γ-1° = 10.8).

Based on these results, we next wondered whether electronic deactivation of the methylenic sites in substrates that are structurally related to **10** might be used to alter the intramolecular site-selectivity toward the primary site. It is well established that protonation of basic nitrogen centers by Brønsted acids deactivates adjacent C–H bonds toward HAT to electrophilic reagents.^22^ Inspired by this concept, we considered the incorporation of a NH unit within the cyclohexane scaffold (**11**). Remarkably, upon protonation of the piperidine moiety with HBF_4_, lactonization occurred exclusively at the primary C–H bond (**11a**) leaving the electronically deactivated γ-methylenes untouched. On the other hand, oxidation of the nonprotonated substrate **11** lead to an intractable mixture of unidentified products. Albeit preliminary, this strategy provides evidence that selective primary C–H bond oxidation initiated by HAT can occur even in the presence of intrinsically weaker C–H bonds, as long as the latter are electronically deactivated or structurally inaccessible.

### Combined Experimental and Computational Analysis of the Reaction Mechanism

#### 1 Mechanism of C–H Cleavage and Nature of the Oxidizing Species

**Figure 3:**
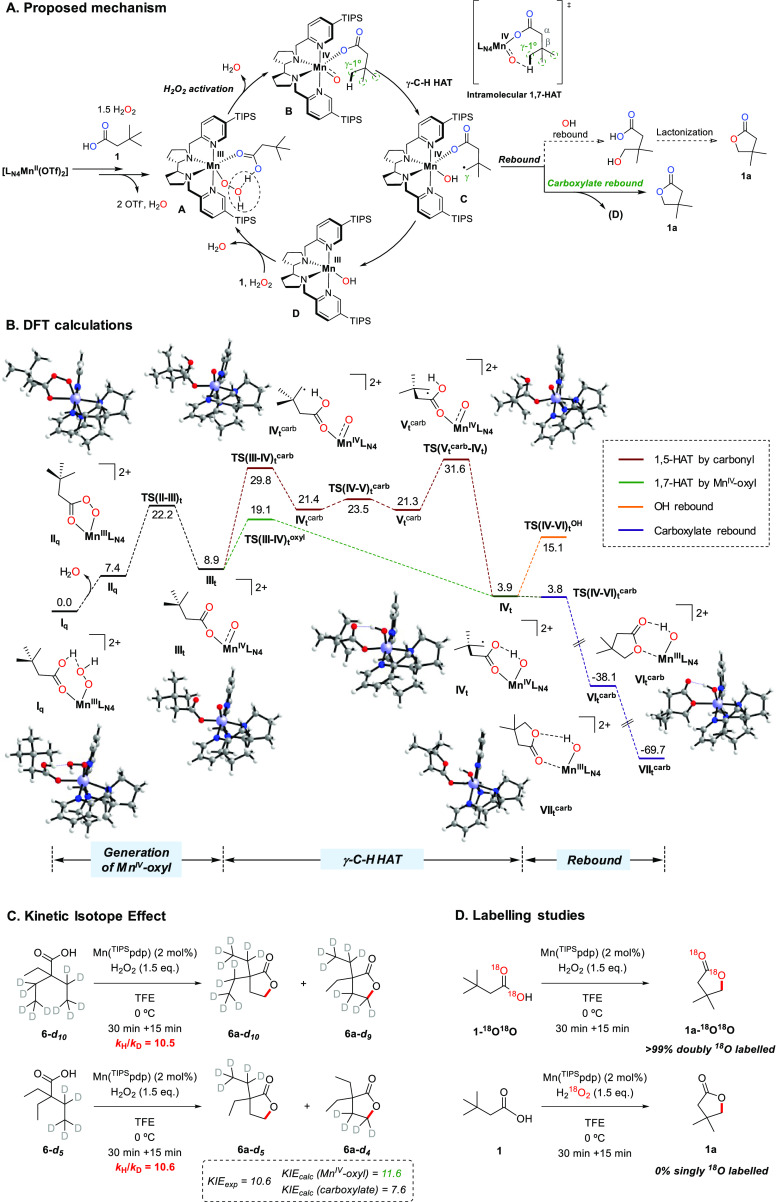
(A) Proposed general mechanism for the Mn-catalyzed primary γ-C–H bond lactonization. (B) Reaction profile for the γ-C–H lactonization of 3,3-dimethylbutanoic acid (**1**) computed at the B3LYP-D3BJ/Def2TZVPP/SMD(TFE)//B3LYP-D3BJ/Def2SVP/SMD(TFE) level of theory for the Mn(pdp) complex. Free energies are given in kcal·mol^–1^. Subscripts t and q represent spin states S = 1 and S = 3/2, respectively. (C) Kinetic isotope effect experiments. (D) ^18^O labelling experiments.

Building on the previously proposed mechanism for the intermolecular reactions, a general reaction energy profile for γ-C–H bond lactonization has been computed using **1** as substrate (see SI section 6 for detailed information) and is depicted in Figure 3, A.^12b,23^ There is ample evidence that the initial Mn^II^ complexes are precatalysts that undergo oxidation to Mn^III^, which in turn is the catalytically competent species that binds and activates, with the assistance of the metal-bound carboxylic acid, H_2_O_2_ to generate the active oxidant.^24^ Therefore, a DFT analysis of the catalytic reaction was undertaken starting from Mn^III^-hydroperoxo (**I**_**q**_) formed after H_2_O_2_ binding to the Mn^III^-carboxylato species (Figure 3, B). An initial reaction of the peroxide and the carboxylic acid, favorable on thermodynamic grounds,^25^ was discarded because the peroxide is dosed via syringe pump and rapidly consumed by reaction with the catalyst. The first step of the reaction profile is the cleavage of the O–O bond and the release of a water molecule that leads to the endergonic (7.4 kcal·mol^–1^) formation of Mn^III^-percarboxylate (**II**_**q**_). Then, the reaction proceeds through an equilibrium between **II**_**q**_ and a Mn^IV^-oxyl (**III**_**t**_) species resulting from heterolytic O–O bond cleavage, being the former 1.5 kcal·mol^–1^ more stable, but the latter the active species toward C–H abstraction. The O–O bond cleavage involved in this equilibrium requires overcoming a free energy barrier of 14.8 kcal·mol^–1^ (**TS(II–III)**_**t**_), which also determines the total free energy barrier of the primary γ-C–H bond lactonization (i.e., Δ*G*^‡^ = 7.4 + 14.8 = 22.2 kcal·mol^–1^). Interestingly, the effective oxidation states (EOS) analysis obtained using APOST-3D code^26^ indicates that **III**_**t**_ is best described as a Mn^IV^-oxyl rather than a Mn^V^-oxo species. Such description agrees with the 0.33 and 2.63 spin density located at the oxyl-oxygen and the Mn atoms, respectively; as well as the Mn–O bond order of 1.41. In addition, the calculated Mn–O bond distance of 1.66 Å is in good agreement with the Mn–O bond distance previously obtained by Zhu and Zhang for an active Mn^IV^-oxyl intermediate.^24a^ As expected, such a Mn–O distance is slightly larger than those found in related Mn compounds characterized as Mn^V^-oxo (1.56 Å).^27^ In intermediate **III**_**t**_, the unpaired *p* electron of the oxyl radical interacts with a lone pair of the substrate carbonyl oxygen giving rise to an O–O bond order of 0.5, ruling out the description of **III**_**t**_ as Mn^V^-oxo. The figures of the effective atomic orbitals involved in this O–O interaction are displayed in the SI (Figure S1). The substrate carbonyl and coordinating oxygen of **III**_**t**_ have a spin density of −0.24 and −0.03, respectively, so that the carboxylate can be considered a X-ligand in the context of Kuhn et al. recent manuscript (Table S10).^28^ We note on passing that notable aspects of the electronic structure of **III**_**t**_, namely the elongation of the Mn–O bond and the weak O–O interaction are very similar to those observed for a related iron system, highly reactive in C–H oxygenation, for which the intermediate has been spectroscopically and computationally characterized.^29^

All the attempts to find a reaction path that involves HAT from γ-C–H bonds starting from **II**_**q**_ either failed or proceeded through initial interconversion, along the reaction coordinate, of **II**_**q**_ into **III**_**t**_. Therefore, we have focused on the study of HAT pathways from γ-C–H bonds starting from intermediate **III**_**t**_ by analyzing two possible mechanisms. The first one considers HAT to the catalyst Mn^IV^-oxyl group (Figure 3, B, **TS(III–IV)**_**t**_^**oxyl**^, green line), which may be viewed as a intramolecular 1,7-HAT process, whereas in the alternative path HAT occurs to the carbonyl group of the bound substrate (Figure 3, B, **TS(III–IV)**_**t**_^**carb**^, red line), via a intramolecular 1,5-HAT. A priori, we considered the 1,5-HAT as the most likely mechanistic scenario which may explain the exquisite γ-selectivity because of a favorable 6-membered cyclic transition state. In fact, 1,5-HAT reactions are far more common than 1,7-HAT’s, for which only limited examples have been described.^30^ However, in the present system, C–H cleavage mediated by the Mn^IV^-oxyl via 1,7-HAT, leading to the Mn^IV^-hydroxo species (**IV**_**t**_) bearing a terminal γ-carbon radical in the bound substrate, is strongly preferred over the alternative 1,5-HAT pathway (ΔΔ*G*^‡^ = 10.7 kcal·mol^–1^). In addition, similar differences in activation free energy have also been obtained for the corresponding reactions of other substrates such as hexanoic acid and 2,2-diethylbutanoic acid (**6**) (Table S11), supporting the hypothesis that 1,7-HAT to the Mn^IV^-oxyl is the main pathway for both methylenic and primary γ-C–H bond oxidation. In the **TS(III–IV)**_**t**_^**oxyl**^ 1,7-HAT transition state, the substrate approaches sideways (TS Mn–O–H angle is 114°), thus the reaction proceeds via a π-channel, i.e., through the π_xy_* Mn–O molecular orbital.^31^**IV**_**t**_ has also a X-ligand character,^28^ with spin densities of only 0.1 and −0.01 on the carbonyl and coordinating oxygen atoms, respectively.

The results of intramolecular kinetic deuterium isotope effect studies, carried out on 2,2-diethylbutanoic acids **6-*d***_**10**_ and **6-*d***_**5**_, deuterated at one or two ethyl groups (Figure 3, C), provide additional mechanistic information on the nature of the HAT step. Primary KIEs of 10.5 and 10.6 were experimentally determined for the oxidation of the two substrates. The DFT-predicted KIE for the 1,7- and 1,5-HAT pathways in **6** promoted by the Mn^IV^-oxyl and carbonyl groups, respectively, were calculated including two explicit TFE molecules in the computational model in order to account for explicit solvent hydrogen bonding to the oxygen of the Mn^IV^-oxyl moiety (full details are provided in Table S12 and Figure S2). KIE_calc_ values of 11.6 and 7.6, respectively, were obtained for the two pathways. Comparison of these values with the experimentally determined one shows a good agreement only with the former one, providing additional support to the hypothesis of an intramolecular 1,7-HAT to the Mn^IV^-oxyl group.

The magnitude of the KIE deserves special consideration. In first place the value is higher than the classical limit. In addition, a time-dependent analysis also demonstrates that the KIE value remains constant over the reaction time, indicating that the operative mechanism is retained along all the reaction course, and as a consequence, discarding potential catalyst speciation (Table S8). In second place, such KIE values are substantially higher than those obtained for intermolecular oxygenation reactions initiated by HAT from secondary and tertiary sites to Mn- and Fe-oxo species (KIE = 3.2–5.0)^24d,32^ and for HAT reactions promoted by oxygen centered radicals (KIE ≤ 4).^33^

#### 2 Computational Analysis of the Origin of Site-Selectivity

One of the most notable aspects of the reaction under investigation is that the outstanding site-selectivity for γ-C–H bond lactonization is maintained even in the oxidation of substrates bearing competitive sites characterized by lower BDEs. In order to clarify this aspect, the selectivity toward lactonization at γ-C–H over β- or δ-C–H bonds has been investigated using hexanoic acid as a model substrate (Figure S3). The C–H bond energies for the β-, γ-, and δ-methylene units of the hexanoic acid were computed to be 97.2, 96.7, and 96.5 kcal·mol^–1^, respectively, which indicates comparable thermodynamic features for the three homolytic C–H bond cleavages. Therefore, the site-selectivity cannot be explained on the basis of ground state energy considerations but must instead be ruled by kinetics. This has been studied by computing the free energy barrier for intramolecular HAT from the β-, γ-, and δ-C–H bonds to both the Mn^IV^-oxyl and the carbonyl group of the catalyst bound substrate (Table S13). Consistent with the results presented above for γ-C–H bond lactonization, the free energy barriers are always lower for the pathway promoted by the Mn^IV^-oxyl (Table S13). Most importantly, the free energy barriers obtained for this pathway are 7.3, 10.2, and 15.7 kcal·mol^–1^ for HAT from the γ-, δ-, and β-C–H bonds, respectively, clearly indicating that the Mn^IV^-oxyl group preferentially promotes γ-C–H lactonization via a 1,7-HAT pathway (Figure 3, B).

#### 3 Computational Analysis of the C–O Bond Forming Step

Following C–H cleavage via intramolecular 1,7-HAT, carbon radical **IV**_**t**_ proceeds through a barrierless carboxylate rebound (**TS(IV–VI)**_**t**_^**carb**^), to form the observed γ-lactone coordinated with the most stable linkage isomer **VII**_**t**_^**carb**^ (Figure 3, B, purple line). The results of the DFT calculations indicate that the alternative OH rebound involving **IV**_**t**_ is uphill by 11.3 kcal·mol^–1^ (Figure 3, B, **TS(IV–VI)**_**t**_^**OH**^, orange line), and is thus not expected to compete at any significant extent.

In order to provide experimental support to this picture, ^18^O-enriched 3,3-dimethylbutanoic acid **1-**^**18**^**O**^**18**^**O** (51% doubly labeled) was prepared and oxidized under the standard catalytic conditions using H_2_O_2_. The predominant formation of the doubly ^18^O labelled γ-lactone **1a-**^**18**^**O**^**18**^**O** (>99% doubly ^18^O-labelled after correction for ^18^O% enrichment of the substrate) clearly indicates that the carboxylate rebound mechanism exclusively accounts for lactone formation, in complete agreement with the DFT prediction (Figure 3, D). The same outcome using **1-**^**18**^**O**^**18**^**O** is also observed along the series of Mn catalysts interrogated in this study, irrespective of their steric (Table S9, entries 1–3 and 8) and electronic properties (Table S9, entries 9–10). In addition, the complementary oxidation of **1** using labelled H_2_^18^O_2_ as oxidant, leads to the exclusive formation of the nonlabelled γ-lactone **1a** (Figure 3, D, and Table S9, entry 4). In contrast, in analogous labelling experiments conducted previously with Fe(pdp) and Mn(pdp) analogues in methylene lactonization reactions, partial incorporation of ^18^O stemming from H_2_^18^O_2_ was observed, indicating a contribution of the classic oxygen rebound pathway to lactone formation (see also Table S9, entries 11–14).^12b,34^

In conclusion, these results showcase that Mn-catalyzed lactonization of primary C–H bonds, irrespective of the reaction conditions (ligand, solvent, and additive), follows a well-defined mechanism in which the primary carbon centered radical species, generated by the first 1,7-HAT event, is rapidly trapped by the carboxylate ligand, without the intermediacy of a γ-hydroxy acid.

### Application to Late Stage Oxidation of Complex Molecules

**Figure 4:**
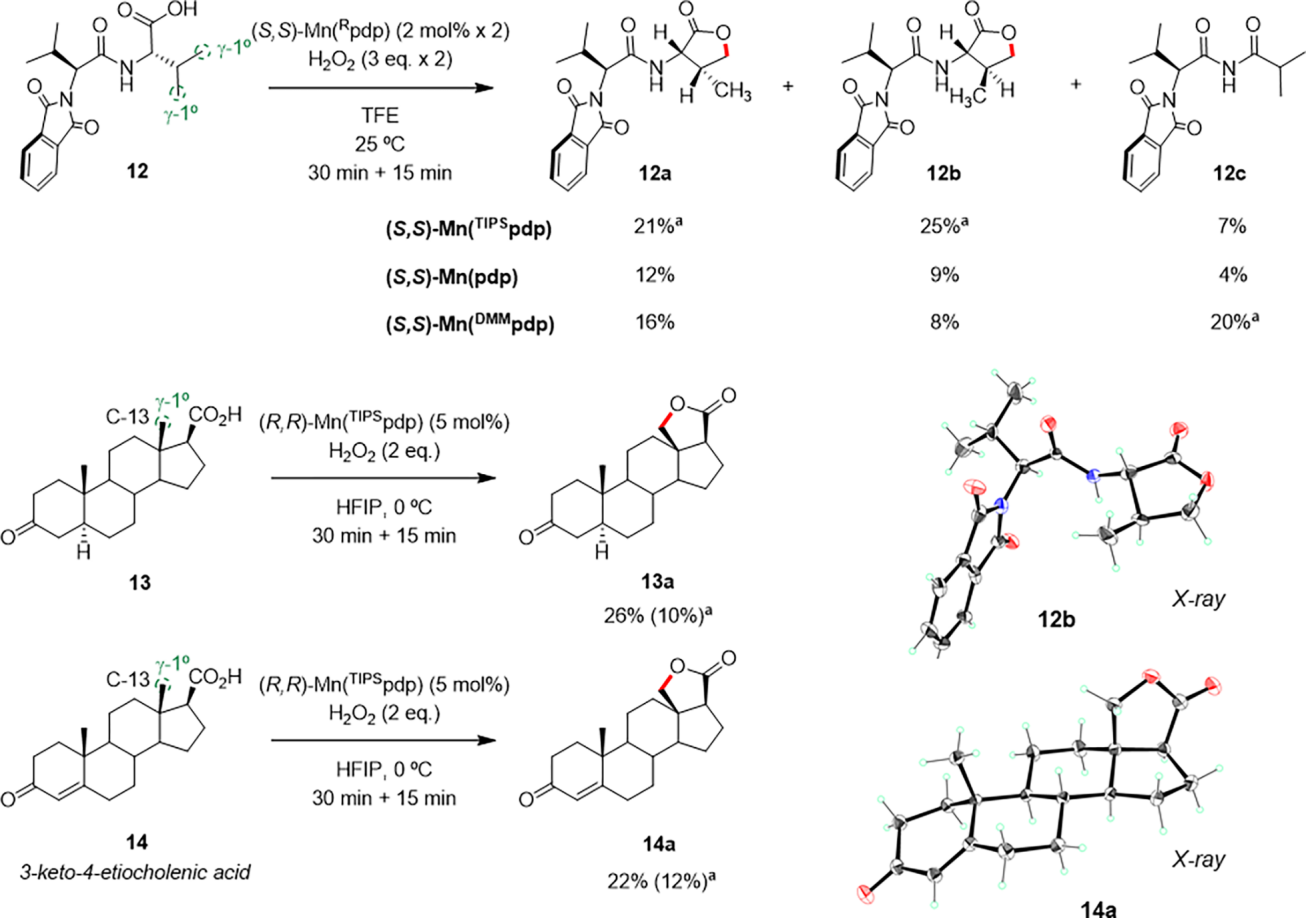
γ-C–H lactonization of primary C–H bonds in complex substrates. Yields were determined by NMR using 1,3,5-trimethoxybenzene as an internal standard. ^*a*^Isolated yield. In all the experiments, no recovery of the starting material was observed.

To explore the potential of this methodology in more complex molecular settings, the *N*-phthalimide protected dipeptide *N*-Phth-Val-Val-OH (**12**) bearing 2 diastereotopic primary sites adjacent to a weaker tertiary β-C–H bond was subjected to the reaction conditions employing the (*S*,*S*)-Mn(^TIPS^pdp) catalyst (Figure 4). As such, the reaction leads to the formation of the two diastereoisomeric γ-lactones **12a** and **12b** deriving from C–H bond oxidation at the C-terminal amino acid residue in a 1:0.8 ratio and overall 46% isolated yield. A smaller amount (7% yield) of product **12c** deriving from decarboxylation was also observed. This result further demonstrates that the carboxylic acid directed γ-lactonization dominates over oxidation of weaker C–H bonds, even when these bonds are in close proximity to the γ position. Similar product distributions, accompanied however by lower yields, were observed when employing the (*S*,*S*)-Mn(pdp) catalyst, indicating that the **12a**:**12b**:**12c** product ratio is influenced to a limited extent by the steric properties of the catalyst. On the other hand, with the more electron-rich (*S*,*S*)-Mn(^DMM^pdp) catalyst, a significant increase in diastereomeric ratio was observed (**12a**:**12b** = 2:1), but most interestingly, **12c**, formed in 20% yield is now the major product. Identification of this compound shows that decarboxylation can compete with C–H oxidation in these reactions.

Biologically relevant substrates such as steroidal compounds are particularly interesting because of their molecular complexity. They contain multiple tertiary and secondary C–H bonds characterized by similar BDEs and their oxygenated pattern can modulate their physical and biological properties.^35^ The selective oxidation of such compounds with bioinspired catalysts has been explored to some extent by manipulating the steric and chiral properties of the catalysts,^36^ and also via supramolecular recognition.^37^ Catalyst dependent selectivities have been observed, demonstrating the powerful reach of these catalysts toward steroid diversification via aliphatic C–H bond oxidation. However, in all cases described so far oxidations occurred at secondary and tertiary sites, leaving primary C–H bonds untouched. It is therefore understandable that targeting unactivated primary C–H bonds by HAT in such complex structures is particularly challenging. Current synthetic methods for this purpose are restricted to the use of N- and O-centered radicals or biocatalytic systems.^7b,7d^ Even with this string of challenges, the oxidation of the angular methyl group at C-13 of steroid **13** occurs with a synthetically valuable yield of 26% for γ-lactone **13a**. Along the same line, the oxidation of 3-keto-4-etiocholenic acid (**14**) delivers γ-lactone **14a** in comparable yield (22%), and although several nonidentified oxidation products are generated in small amounts, the enone functionality at ring A is left untouched (Figure 4). Of notice, despite both TFE and HFIP are suitable solvents, the oxidation of complex substrates consistently proceeds with slightly improved yields in HFIP making it the solvent of choice.

Because the present catalytic system exhibits outstanding γ-selectivity, we next aimed to evaluate the directed oxidation of the *gem*-dimethyl groups present in relevant natural products or derivatives containing carboxylic acid groups. Camphor-based compounds exhibit a range of biological activities, including antifungal, antituberculosis, antiviral, and antimicrobial properties.^38^ The camphor skeleton confers a defined shape and rigidity that we envision will facilitate discrimination between the two sets of nonequivalent primary γ-C–H bonds upon binding to the chiral Mn catalyst. Hence a general method for the chemo- and stereoselective functionalization of these primary sites would provide a new family of camphor-based compounds that would otherwise be extremely difficult to access. Existing chemical manipulations of camphor derivatives mainly focus on the functionalization of the cycloalkane framework, with functionalization at the *gem*-dimethyl moiety being practically disregarded.^38^

**Figure 5:**
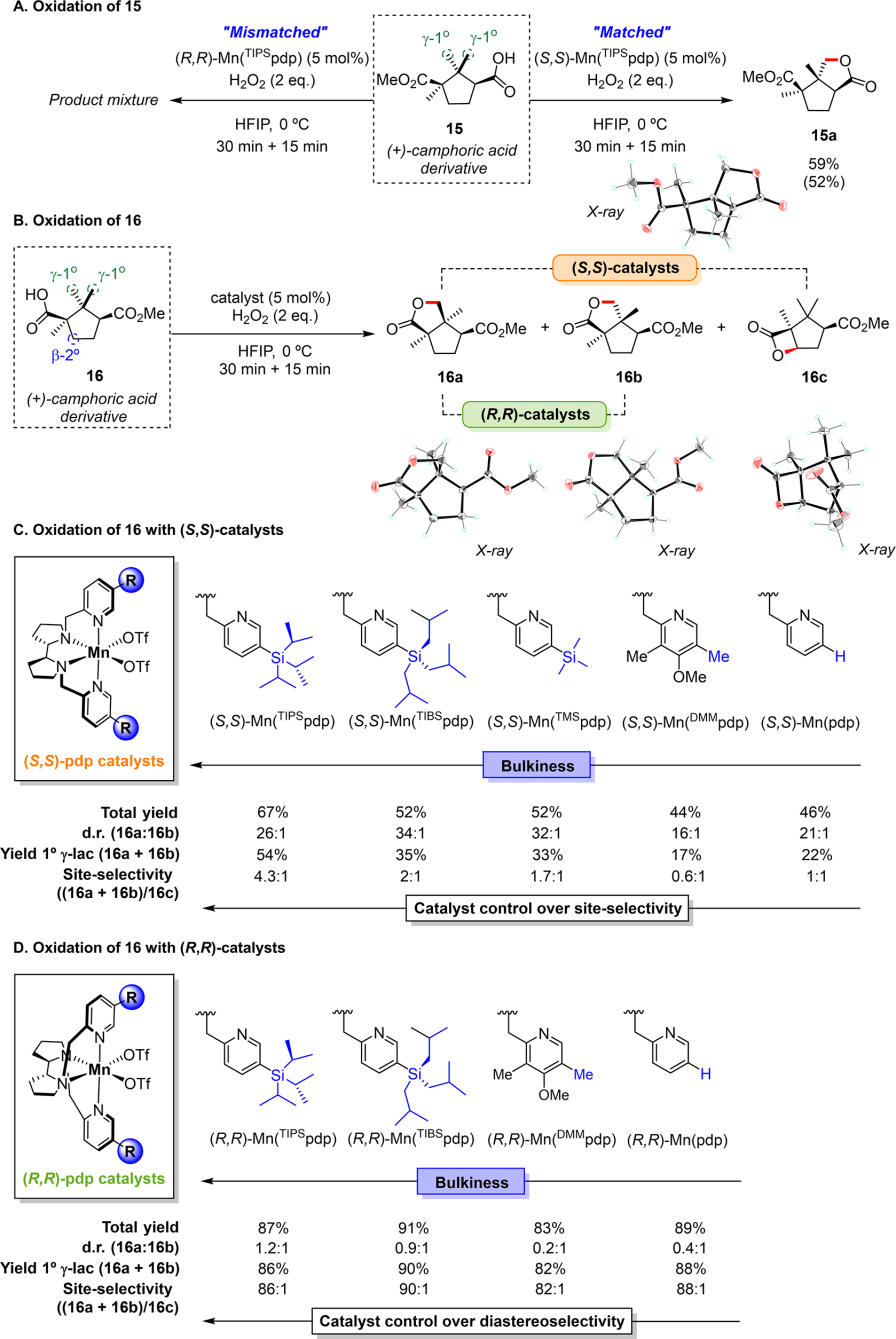
(A) Oxidation of **15** showing the matched/mismatched behavior. (B) Oxidation of **16** showing the three lactone products formed and the impact of the steric properties of the Mn catalyst used (C,D). Diastereoisomeric ratio (d.r.) corresponds to the **16a**:**16b** ratio. Site-selectivity refers to the primary/secondary product ratio, i.e., (**16a** + **16b**):**16c**. Conversions, yields, and detailed reaction conditions are shown in the SI, Tables S3 and S4. Yields were determined by GC analysis using internal standard calibration (see SI). Isolated yield of **15a** is shown in parenthesis.

We started to explore the oxidation of the singly esterified camphoric acid derivative **15** (Figure 5, A). Such a chiral substrate bears two γ primary sites and β- and γ- endocyclic methylenic sites accessible for carboxylic acid directed oxidation. Therefore, several stereo- and regioisomers can be formed upon lactonization. Using the (*S*,*S*)-Mn(^TIPS^pdp) catalyst, oxidation of **15** occurs exclusively at one primary site delivering bicyclic γ-lactone **15a** as a single diastereoisomer in 59% yield. On the other hand, oxidation of **15** with the enantiomeric (*R*,*R*)-Mn(^TIPS^pdp) catalyst results in full conversion of the substrate to multiple unidentified products, detected in small amounts, and may be regarded as a “mismatched combination” (referring to the chirality of catalyst and substrate).

We next evaluated the oxidation of camphoric acid derivative (**16**), a structural isomer of **15** that holds the directing carboxylic acid group bound to a quaternary carbon (Figure 5, B). In this case, γ-C–H lactonization can occur at two primary sites (products **16a** or **16b**) or at a secondary one. Oxidation of **16** with (*S*,*S*)-Mn(^TIPS^pdp) generates bicyclic γ-lactone **16a**, bearing a *cis* ring junction, as the main product in 52% yield (Figure 5, C, and Table S2). Oxidation occurs with an outstanding diastereoisomeric ratio (d.r.) **16a**:**16b** of 26:1, with the bicyclic γ-lactone **16b**, bearing a *trans* ring junction being detected only in trace amount (2% yield). Surprisingly, formation of the four-membered ring β-lactone **16c** originated from lactonization at the proximal secondary site is also observed in 13% yield. No products deriving from oxidation at the more easily accessible γ-methylene site have been detected, likely due to electronic deactivation exerted by the adjacent ester group. We note that such β-C–H bond oxidation is unprecedented in Mn-catalyzed directed lactonization, demonstrating the potential of such procedure beyond γ-functionalization. Nevertheless, the primary/secondary product ratio ((**16a** + **16b**):**16c** = 4.3:1) constitutes a piece of evidence of the high preference of the (*S*,*S*)-Mn(^TIPS^pdp) catalyst for oxidation of the primary γ-C–H bonds over the thermodynamically favored methylenic sites.

Remarkably, site-selectivity can be finely tuned by precisely manipulating the space around the catalytic center, while maintaining the chirality of the metal. While the diastereomeric preference for the *cis* methyl oxidation is retained throughout all the series, systematic decrease of the steric bulk on position 5 of the catalyst pyridine moiety gradually reduces the 1°/2° lactone ratio from 4.3:1 with the sterically demanding (*S*,*S*)-Mn(^TIPS^pdp) to 1:1 with the unhindered (*S*,*S*)-Mn(pdp) catalyst (Figure 5, C and Table S3). As described elsewhere, HAT from more sterically hindered C–H bonds can be efficiently promoted using less sterically congested catalysts. We note on passing that this concept has been applied to favor oxidation of secondary over tertiary sites,^39^ although oxidation of primary C–H bonds has remained inaccessible and such comparison is unprecedented. Along these lines, the present results suggest that the competition between primary and secondary C–H bond oxidation can be also rationally and systematically controlled by changing the steric properties of the Mn catalyst. Importantly, the electron-rich (*S*,*S*)-Mn(^DMM^pdp) catalyst led to an inversion of the primary/secondary product ratio to 0.6:1, suggesting that the electronic properties of the ligand can represent an additional tool for tuning site-selectivity.^11a^ Finally, selectivity toward primary C–H bond oxidation was maximized up to 5.9:1 when using the bulky (*S*,*S*)-Mn(^TIPS^mcp) catalyst, although the yield of lactones **16a** (42%) and **16c** (7%) slightly decreased (Table S3).

As **16** is a chiral substrate we then extended our study by exploring the oxidation of **16** using catalysts with the opposite absolute configuration of the pdp backbone. We were pleased to observe that catalyst (*R*,*R*)-Mn(^TIPS^pdp) furnishes the three lactones **16a**, **16b**, and **16c** in a 47:39:1 ratio, thus providing an outstanding selectivity toward the oxidation at primary sites (86:1 ratio) (Figure 5, D). Interestingly, diastereoselectivity is again controlled by the steric properties of the ligand (Figure 5, D and Table S4). Hence, formation of lactone **16b** is notably promoted when reducing the size of the ligand substituents; up to 67% when using the (*R*,*R*)-Mn(^DMM^pdp) catalyst (d.r. = 0.2:1). Another important observation is that the total yield for primary γ-C–H bond lactonization is maintained virtually constant and close to 90%, whereas **16c** is detected only in trace amounts along the series of (*R*,*R*)-catalysts screened (82–90:1 primary/secondary product ratio) (Figure 5, D and Table S4). Overall, appropriate selection of catalyst structure and chirality allowed functionalization at three different C–H sites on the same camphoric acid skeleton.

**Figure 6:**
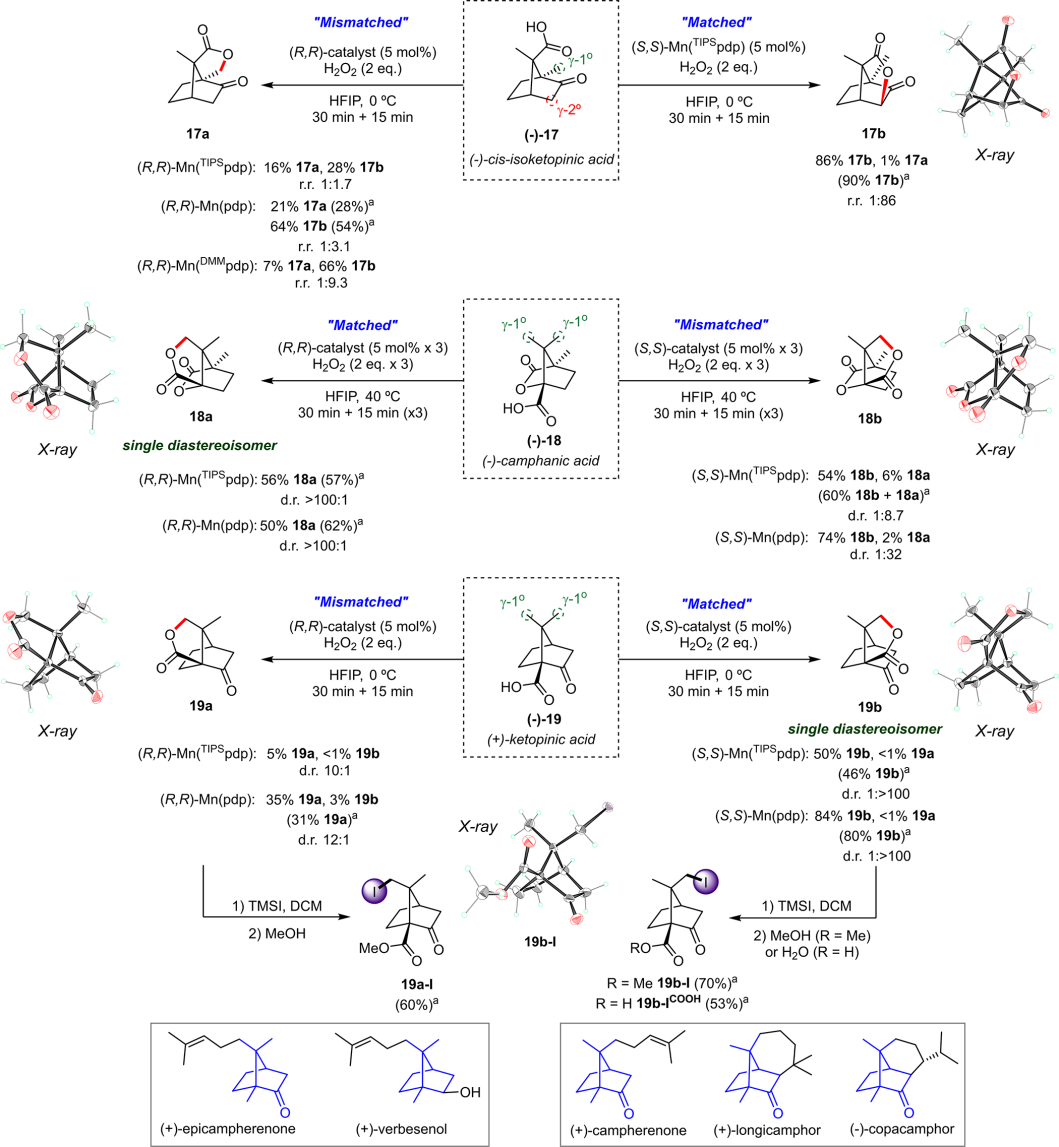
Primary γ-C–H lactonization in natural products and derivatives. Conversions, yields, and detailed reaction conditions are shown in the SI, Tables S5–S7. Yields were determined by GC analysis of 100 μmol scale reactions using internal standard calibration (see SI). ^*a*^Isolated yield on 0.3–0.5 mmol scale reactions (see SI). Regioisomeric ratio (r.r.) refers to the primary/secondary product ratio (i.e., **17a**:**17b**). Diastereoisomeric ratio (d.r.) refers to the ratio of diastereoisomers (i.e., **18a**:**18b** and **19a**:**19b**).

The remarkably rich catalyst dependent site-selective and diastereoselective oxidation of camphoric acid derivatives **15** and **16** prompted us to investigate the oxidation of a series of bicyclic camphor derivatives (**17**–**19**) bearing the carboxylic group bound to a quaternary carbon (Figure 6). We envisioned that the higher rigidity of such systems may enforce recognition of the substrate by the chiral catalyst, potentially affording predictable selectivity patterns. Along this line, the oxidation of (−)-*cis*-isoketopinic acid (**17**) using (*S*,*S*)-Mn(^TIPS^pdp) occurs efficiently at the electronically deactivated γ-methylene site delivering lactone **17b** in 86% yield, together with a negligible amount of the primary γ-C–H bond lactonization product **17a** (**17a**:**17b** = 1:86). The unusual oxidation of this electron-poor, deactivated methylenic site likely occurs because of the conformationally rigid structure, which orients the *exo* C–H bond of the γ-CH_2_ unit toward the reactive Mn oxidant, promoting formation of **17b**. Such outstanding site-selectivity is severely affected by inverting the absolute configuration of the Mn catalyst, which maximizes the formation of **17a** to the detriment of **17b** (16% and 28% yield, respectively, **17a**:**17b** = 1:1.7). No other oxidation product has been detected, in particular the ones deriving from lactonization at the weaker but conformationally inaccessible bridgehead tertiary β-C–H bond. The ratio of the two lactones is again catalyst controlled (Figure 6 and Table S5). Despite the fact that lactonization at the secondary γ-C–H bond prevails over that at the primary site, reducing the space around the catalyst center increases selectivity toward the less sterically hindered γ primary site, although the results obtained with Mn(^DMM^pdp) also suggest a contribution deriving from catalyst electronics.

Under optimized conditions the oxidation of (−)-camphanic acid (**18**) using (*R*,*R*)-Mn(^TIPS^pdp) led to selective γ-lactonization at a single diastereotopic methyl group of the *gem*-dimethyl unit to give product **18a** in 56% yield with an exquisite diastereoselectivity over lactone **18b** (d.r. > 100:1) (Figure 6 and Table S6). The reaction occurs without the formation of byproducts detectable by GC. Interestingly, by using the enantiomeric catalyst (*S*,*S*)-Mn(^TIPS^pdp), the selectivity is drastically switched toward oxidation at the other methyl, affording **18b** in 54% yield accompanied by 6% of **18a** (d.r. = 1:8.7) (Figure 6).

In perspective, the above-described finding demonstrates the unique potential of the reaction to diversify biologically relevant scaffolds via catalyst dependent selective oxidation of methyl groups.

### Theoretical Analysis of the Diastereoselective Oxidation of *gem*-Dimethyl Groups

**Figure 7:**
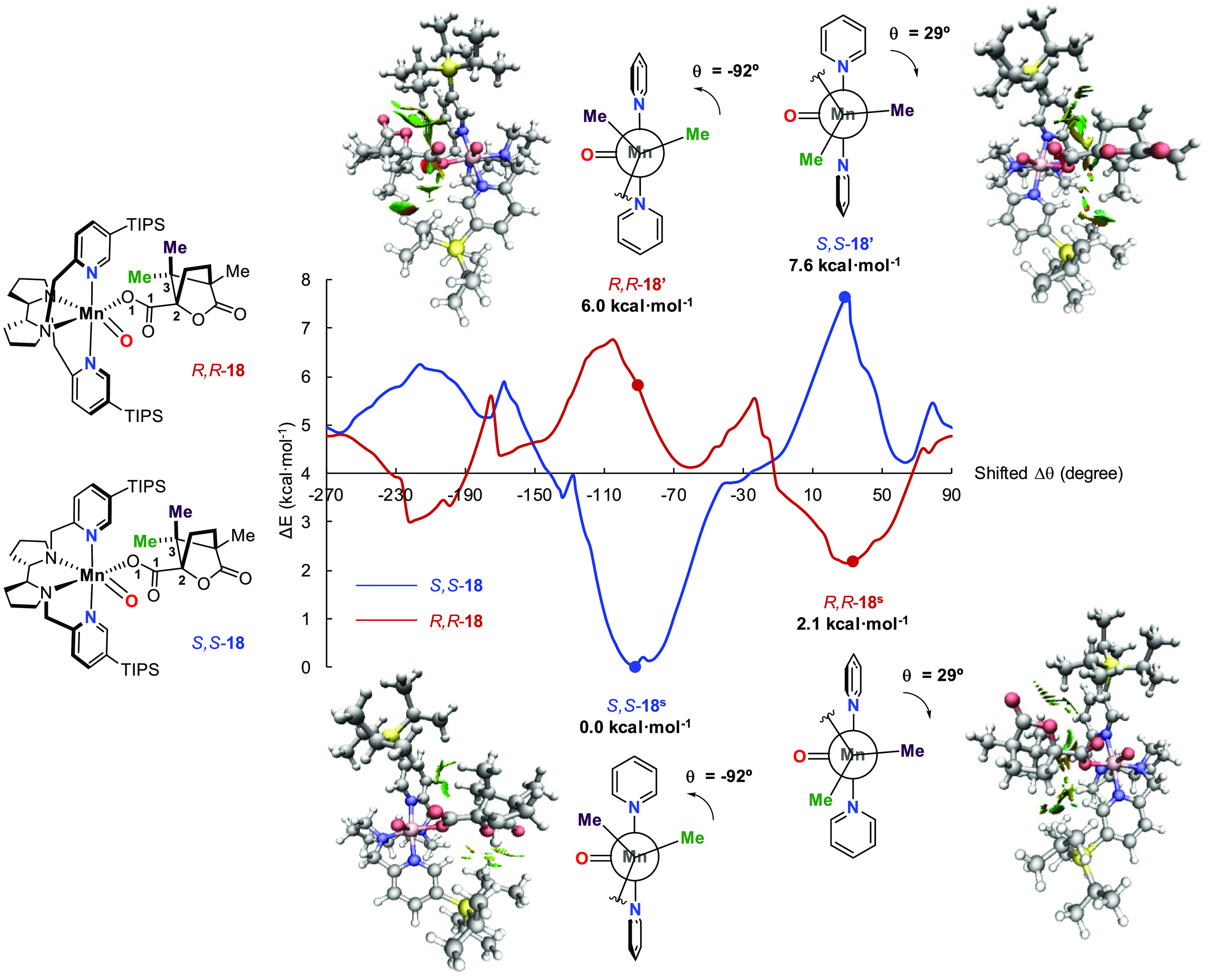
Electronic energy profiles obtained for the dihedral angle (θ) rotation relaxed scan for *S*,*S***-18** (blue) and *R*,*R***-18** (red). The scanned dihedral angle is formed between the atoms O_1_, C_1_, C_2,_ and C_3_ of **18**. Simplified model Newman projections through the axis defined by the Mn and C_3_ atoms. (where, for the sake of simplicity, the groups directly bound to C_2_ and the TIPS groups have been omitted), together with the structures showing the noncovalent interactions obtained using the NCIPLOT program^40^ are depicted for the rotamers at Δθ = −92° and Δθ = 29°. Negative angles indicate anticlockwise rotation, while positive angles indicate clockwise rotation. Details of the computational methods are provided in the SI.

The catalyst dependent diastereoselective oxidation of *gem*-dimethyl groups constitutes a unique feature of the current system. Interestingly, this aspect can be explained by a computational model that permits prediction of the reaction diastereoselectivity. For this purpose, we analyzed the coordination of the chiral substrate **18** via the carboxylic acid moiety to the two enantiomers of the Mn(^TIPS^pdp) catalyst with a simple and fast in silico protocol based on the rotational scan of the dihedral angle (θ) between the atoms O_1_, C_1_, C_2_, and C_3_ of **18** in the two chiral complexes displayed in Figure 7. Such dihedral angle maps the rotation of the C–C σ-bond between the C_1_ and C_2_ which rules the relative orientation of the substrate with respect to the catalyst core, described by means of simplified model Newman projections through the axis defined by the Mn and C_3_ atoms. The two complexes formed upon substrate coordination to (*S*,*S*)-Mn(^TIPS^pdp) and (*R*,*R*)-Mn(^TIPS^pdp) are referred to as *S*,*S***-18** and *R*,*R***-18**, respectively. Figure 7 depicts the θ dihedral angle rotational relaxed scan for a complete rotation for both *S*,*S***-18** and *R*,*R***-18**. It is observed that the most stable rotamer of the *S*,*S***-18** complex (labeled as *S*,*S***-18**^**s**^) is found for Δθ_SS_ = −92°. On the other hand, the lowest energy rotamer for the *R*,*R***-18** complex (i.e., *R*,*R***-18**^**s**^) is found at Δθ_RR_ = 29° and is 2.1 kcal·mol^–1^ higher in energy than *S*,*S***-18**^**s**^. Remarkably, complex *S*,*S***-18′** with a dihedral angle Δθ_SS_ = 29° appears to be the absolute maximum in the rotational scan with a relative electronic energy value of 7.6 kcal·mol^–1^. Analogously, complex *R*,*R***-18′** with a dihedral angle Δθ_RR_ = −92°, almost coincides with the maximum of the scan for *R*,*R***-18**, appearing at 6.0 kcal·mol^–1^. Consequently, the most stable *S*,*S***-18**^**s**^ conformation corresponds to the most unstable one for the *R*,*R***-18** complex, and vice versa.

The reason behind the stability of *S*,*S***-18**^**s**^ and *R*,*R*-**18**^**s**^ with respect to *S*,*S***-18′** and *R*,*R***-18′**, respectively, is the repulsion between the bulky TIPS substituents and the methyl groups of **18**. In this regard, the distance of such groups with respect to the closest carbon of the TIPS groups is in clear correspondence with the trends observed in the rotational scans (see Figure S4 in the SI). Moreover, the relative position of the bulky TIPS groups, which depends on the absolute configuration of the pdp backbone, determines the catalytically active relative orientation of the substrate. The most stable *S*,*S***-18**^**s**^ and *R*,*R***-18**^**s**^ rotamers contain the bound substrate experiencing minimum dispersive interactions and therefore better fitting in the active catalyst pocket, as can be clearly seen in the plots indicating the location of the repulsive noncovalent interactions (Figure 7). Newman-type projections relating the relative conformational orientation of the two methyl groups of the substrate with respect to the Mn-oxo vector are shown in Figure 7. For the energetically more favorable rotamers (*S*,*S***-18**^**s**^ and *R*,*R*-**18**^**s**^) the projections illustrate in a quite straightforward manner the proximity between the Mn oxidant and the reactive methyl group for each enantiomer of the catalyst. Such theoretical analysis provides a clear picture of how the present system differentiates between such diastereotopic methyl groups and is in good agreement with the experimental **18a**:**18b** ratios obtained with the two enantiomeric Mn(^TIPS^pdp) catalysts (Figure 6).

Unexpectedly, the matched/mismatched complementarity between chiral substrate and chiral catalyst is not necessarily improved by the presence of the bulky TIPS groups. In this regard, while the unhindered (*R*,*R*)-Mn(pdp) catalyst behaves similarly as the bulkier (*R*,*R*)-Mn(^TIPS^pdp), the use of (*S*,*S*)-Mn(pdp) magnifies both activity (74% yield for **18b**) and diastereoselectivity for **18b** formation (**18a**:**18b** = 1:32, Figure 6). It is therefore reasonable to consider that the diastereocontrol in the oxidation of **18** is mainly dictated by the chirality at the metal and, to some extent, affected by the steric properties of the ligand. The DFT-computed HAT energy barriers obtained with the Mn(pdp) system indeed support the stereoselectivity observed in the oxidation of **18** at the *gem*-dimethyl unit. Using the (*S*,*S*)-Mn(pdp) catalyst 1,7-HAT from the primary γ-C–H bond to the Mn^IV^-oxyl unit finally leading to **18b** is preferred by 3.8 kcal·mol^–1^. In sharp contrast, and consistent with the experimentally observed reverse diastereoselectivity, the (*R*,*R*)-Mn(pdp) system affords **18a** over **18b** by a significantly larger 4.7 kcal·mol^–1^ (Table S14 and Figure S5).

Similar observations are obtained in the oxidation of (+)-ketopinic acid (**19**), where matching between the (*S*,*S*)-Mn(^TIPS^pdp) catalyst and the substrate delivers γ-lactone **19b** in 50% yield, with an outstanding diastereoselectivity over **19a** (**19a**:**19b** = 1:>100) (Figure 6). In line with the results obtained for **18**, by changing catalyst chirality (i.e., using (*R*,*R*)-Mn(^TIPS^pdp)) preferential oxidation at the other methyl group occurs, leading to **19a** with good diastereoselectivity (**19a**:**19b** = 10:1), albeit in low yield (5%). The less structured cavity of (*R*,*R*)-Mn(pdp) allows to some extent to overcome the mismatch issues, increasing the yield of **19a** up to 35% with no erosion in diastereoselectivity (**19a**:**19b** 12:1). Similarly, the yield of **19b** is maximized to 84% when using the (*S*,*S*)-Mn(pdp) catalyst, again with almost no formation of lactone **19a** (**19a**:**19b** 1:>100). The reaction is completely chemoselective for lactonization at primary γ-C–H bonds, with no traces of products deriving from nondirected oxidations, even at higher temperatures (Table S7 in the SI). These observations demonstrate the ability to undergo high diastereoselective lactonization at nonactivated primary γ-C–H bonds by finely tuning both chirality and structural properties of the Mn catalyst.

### Product Elaboration

The versatility of γ-lactones to undergo a vast array of chemical transformations makes such functionalities ideal platforms for chemical diversification. For instance, such cyclic structural motifs can be straightforwardly converted into hydroxy acids, hydroxy amides, hydroxy esters, and 1,4-diols, while maintaining the γ-primary site oxygenated, enabling its further chemical elaboration. Besides the formation of such valuable oxygenated motifs, lactones can experience other related transformations.^41^ As an example of the synthetic potential of our directed lactonization procedure, lactones **19a** and **19b** were reacted with trimethylsilyl iodide (TMSI) followed by methanol transesterification to afford the ring-opened iodoester compounds **19a-I** and **19b-I** bearing the iodine atom selectively installed at a specific terminal γ-CH_2_ site (Figure 6). Through this strategy, these functionalized camphor motifs could significantly streamline the synthesis of natural sesquiterpenoids, avoiding the remodeling of prefunctionalized chiral terpenes, as recently proposed by Sarpong et al.^10a^ Compounds **19a-I** and **19b-I** could be easily converted into (+)-epicampherenone and (+)-campherenone, respectively, the former being a valuable intermediate en route to the synthesis of (+)-longicamphor and (−)-copacamphor (Figure 6).^42^ Alternatively, an analogous ring-opening procedure with TMSI, followed by in situ hydrolysis with water directly affords the iodo carboxylic acid **19b-I**^**COOH**^, which can be in principle amenable to a successive lactonization at a different site of the molecule with high site-selectivity, increasing molecular complexity in a stepwise fashion. As such, the present results set the stage to streamline late-stage primary stereoselective functionalizations of relevant organic frameworks.

## Conclusions

In summary, herein we report the development of a general catalytic stereoselective γ-lactonization of unactivated primary C–H bonds and its application to the late stage functionalization of natural products such as steroids, peptides, and terpenoids. The system relies on chiral Mn catalysts that activate aqueous hydrogen peroxide to oxidize under mild conditions carboxylic acid substrates, that bind to the metal center via the carboxylate moiety. DFT calculations support a reaction that proceeds through the formation of a highly reactive Mn^IV^-oxyl intermediate, which promotes intramolecular 1,7-HAT from a primary γ-C–H bond to deliver a carbon radical that rapidly lactonizes through a intramolecular carboxylate transfer (rebound) mechanism. An unusually large intramolecular kinetic deuterium isotope effect and ^18^O labeling experiments provide strong support to this mechanistic picture. The ligation of the substrate to the Mn core enables high levels of chemoselectivity for the formation of γ-lactones as the (generally) exclusive products. However, an unprecedented example of a β-lactonization has been discovered, which suggests that the path for its formation must be close in energy to the dominant γ-lactonization, uncovering novel avenues for these oxidation reactions.^43^ Notably, the high site-selectivity observed with the present system enables the oxidation of strong primary γ-C–H bonds even in the presence of intrinsically weaker and a priori more reactive secondary and tertiary ones at α- and β-carbons. Key aspects governing the reaction site-selectivity in substrates bearing nonequivalent γ-C–H bonds have been uncovered and include the following: Thorpe–Ingold effects, catalyst and substrate sterics and electronics, and, when dealing with cyclic and bicyclic carboxylic acids, substrate rigidity. Unlike existing and popularized primary C–H bond oxidation processes, by a judicious choice of catalyst structure and absolute configuration, γ-lactonization at the *gem*-dimethyl unit of rigid cyclic and bicyclic carboxylic acids can be achieved with unprecedented levels of diastereoselectivity, that can be rationalized with a simple computational model. Furthermore, γ-lactones offer a useful synthetic handle for (stereo)selective late-stage functionalization of carboxylic acids, valuable as building blocks for organic synthesis, but also as complex bioactive compounds. We envision that the principles presented in this work for the stereocontrolled oxidation of methyl groups can represent a starting point to incorporate such transformations into synthetic routes, allowing novel alternative powerful retrosynthetic disconnections.

## Supporting Information Available

Experimental details for the preparation of metal complexes and substrates and catalytic reactions and details on isolation and characterization of the reaction products; Details on the computational analyses (PDF)

## References

[ref1] aKarimov R. R.; Hartwig J. F. Transition-Metal-Catalyzed Selective Functionalization of C(sp^3^)-H Bonds in Natural Products. Angew. Chem., Int. Ed. 2018, 57, 4234–4241. 10.1002/anie.201710330. PMC7484946 29228463

[ref2] aWhite M. C.; Zhao J. Aliphatic C-H Oxidations for Late-Stage Functionalization. J. Am. Chem. Soc. 2018, 140, 13988–14009. 10.1021/jacs.8b05195. 30185033 PMC6410715

[ref3] aZhang S.; Zhang J.; Zou H. C(sp^3^)–H oxygenation via alkoxypalladium(II) species: an update for the mechanism. Chem. Sci. 2022, 13, 1298–1306. 10.1039/D1SC06907A. 35222913 PMC8809414

[ref4] aChen M. S.; White M. C. A Predictably Selective Aliphatic C–H Oxidation Reaction for Complex Molecule Synthesis. Science 2007, 318, 783–787. 10.1126/science.1148597. 17975062

[ref5] Tang X.; Gan L.; Zhang X.; Huang Z. *n*-Alkanes to *n*-alcohols: Formal primary C–H bond hydroxymethylation via quadruple relay catalysis. Sci. Adv. 2020, 6, eabc6688 10.1126/sciadv.abc6688. 33219029 PMC7679163

[ref6] aLi Y.; Wang J.; Wang F.; Wang L.; Xu Z.; Yuan H.; Yang X.; Li P.; Su J.; Wang R. Production of 10-Hydroxy-2-decenoic Acid from Decanoic Acid via Whole-Cell Catalysis in Engineered Escherichia coli. ChemSusChem 2022, e20210215 10.1002/cssc.202102152. 34796684

[ref7] aCrossley S. W. M.; Tong G.; Lambrecht M. J.; Burdge H. E.; Shenvi R. A. Synthesis of (−)-Picrotoxinin by Late-Stage Strong Bond Activation. J. Am. Chem. Soc. 2020, 142, 11376–11381. 10.1021/jacs.0c05042. 32573211 PMC8011636

[ref8] aBakhoda A. G.; Jiang Q.; Badiei Y. M.; Bertke J. A.; Cundari T. R.; Warren T. H. Copper-Catalyzed C(sp^3^)-H Amidation: Sterically Driven Primary and Secondary C-H Site-Selectivity. Angew. Chem., Int. Ed. 2019, 58, 3421–3425. 10.1002/anie.201810556. 30675976

[ref9] Talele T. T. Natural-Products-Inspired Use of the *gem*-Dimethyl Group in Medicinal Chemistry. J. Med. Chem. 2018, 61, 2166–2210. 10.1021/acs.jmedchem.7b00315. 28850227

[ref10] aLusi R. F.; Sennari G.; Sarpong R. Total synthesis of nine longiborneol sesquiterpenoids using a functionalized camphor strategy. Nat. Chem. 2022, 14, 450–456. 10.1038/s41557-021-00870-4. 35165424 PMC9117171

[ref11] aVicens L.; Olivo G.; Costas M. Rational Design of Bioinspired Catalysts for Selective Oxidations. ACS Catal. 2020, 10, 8611–8631. 10.1021/acscatal.0c02073.

[ref12] aVicens L.; Bietti M.; Costas M. General Access to Modified α-Amino Acids by Bioinspired Stereoselective γ-C–H Bond Lactonization. Angew. Chem., Int. Ed. 2021, 60, 4740–4746. 10.1002/anie.202007899. 33210804

[ref13] Ottenbacher R. V.; Samsonenko D. G.; Talsi E. P.; Bryliakov K. P. Highly Efficient, Regioselective, and Stereospecific Oxidation of Aliphatic C-H Groups with H_2_O_2_, Catalyzed by Aminopyridine Manganese Complexes. Org. Lett. 2012, 14, 4310–4313. 10.1021/ol3015122. 22747086

[ref14] aChen M. S.; White M. C. A Predictably Selective Aliphatic C–H Oxidation Reaction for Complex Molecule Synthesis. Science 2007, 318, 783–787. 10.1126/science.1148597. 17975062

[ref15] Nikishin G. I.; Svitanko I. V.; Troyansky E. I. Direct oxidation of alkanoic acids and their amides to γ-lactones by peroxydisulphate-containing systems. J. Chem. Soc., Perkin Trans. 1983, 2, 595–601. 10.1039/P29830000595.

[ref16] aHuang X.; Groves J. T. Oxygen Activation and Radical Transformations in Heme Proteins and Metalloporphyrins. Chem. Rev. 2018, 118, 2491–2553. 10.1021/acs.chemrev.7b00373. 29286645 PMC5855008

[ref17] aKal S.; Xu S.; Que L. Jr. Bio-inspired Nonheme Iron Oxidation Catalysis: Involvement of Oxoiron(V) Oxidants in Cleaving Strong C-H Bonds. Angew. Chem., Int. Ed. 2020, 59, 7332–7349. 10.1002/anie.201906551. 31373120

[ref18] Pichette Drapeau M.; Goossen L. J. Carboxylic Acids as Directing Groups for C-H Bond Functionalization. Chem. Eur. J. 2016, 22, 18654–18677. 10.1002/chem.201603263. 27730686

[ref19] Dantignana V.; Milan M.; Cussó O.; Company A.; Bietti M.; Costas M. Chemoselective Aliphatic C-H Bond Oxidation Enabled by Polarity Reversal. ACS Cent. Sci. 2017, 3, 1350–1358. 10.1021/acscentsci.7b00532. 29296677 PMC5746866

[ref20] aDeLomba W. C.; Stone E. A.; Alley K. A.; Iannarone V.; Tarsis E.; Ovaska S.; Ovaska T. V. Utilization of the Thorpe-Ingold Effect in the Synthesis of Cyclooctanoid Ring Systems via Anionic 6-*exo-dig* Cyclization/Claisen Rearrangement Sequence. J. Org. Chem. 2020, 85, 9464–9474. 10.1021/acs.joc.0c01132. 32687712 PMC8588887

[ref21] Luo Y.-R. Comprehensive Handbook of Chemical Bond Energies; CRC Press: Boca Raton, 2007.

[ref22] Bietti M. Activation and Deactivation Strategies Promoted by Medium Effects for Selective Aliphatic C-H Bond Functionalization. Angew. Chem., Int. Ed. 2018, 57, 16618–16637. 10.1002/anie.201804929. 29873935

[ref23] aOttenbacher R. V.; Bryliakova A. A.; Shashkov M. V.; Talsi E. P.; Bryliakov K. P. To Rebound or···Rebound? Evidence for the “Alternative Rebound” Mechanism in C–H Oxidations by the Systems Nonheme Mn Complex/H_2_O_2_/Carboxylic Acid. ACS Catal. 2021, 11, 5517–5524. 10.1021/acscatal.1c00811.

[ref24] aFeng A.; Liu Y.; Yang Y.; Zhu R.; Zhang D. Theoretical Insight into the Mechanism and Selectivity in Manganese-Catalyzed Oxidative C(sp^3^)–H Methylation. ACS Catal. 2022, 12, 2290–2301. 10.1021/acscatal.1c05025.

[ref25] aAlabugin I. V.; Kuhn L.; Medvedev M. G.; Krivoshchapov N. V.; Vil’ V. A.; Yaremenko I. A.; Mehaffy P.; Yarie M.; Terent’ev A. O.; Zolfigol M. A. Stereoelectronic power of oxygen in control of chemical reactivity: the anomeric effect is not alone. Chem. Soc. Rev. 2021, 50, 10253–10345. 10.1039/D1CS00386K. 34263287

[ref26] aPostils V.; Delgado-Alonso C.; Luis J. M.; Salvador P. An Objective Alternative to IUPAC’s Approach To Assign Oxidation States. Angew. Chem., Int. Ed. 2018, 57, 10525–10529. 10.1002/anie.201802745. 29787636

[ref27] Li X. X.; Guo M.; Qiu B.; Cho K. B.; Sun W.; Nam W. High-Spin Mn(V)-Oxo Intermediate in Nonheme Manganese Complex-Catalyzed Alkane Hydroxylation Reaction: Experimental and Theoretical Approach. Inorg. Chem. 2019, 58, 14842–14852. 10.1021/acs.inorgchem.9b02543. 31621303

[ref28] Kuhn L.; Vil V. A.; Barsegyan Y. A.; Terent’ev A. O.; Alabugin I. V. Carboxylate as a Non-innocent L-Ligand: Computational and Experimental Search for Metal-Bound Carboxylate Radicals. Org. Lett. 2022, 24, 3817–3822. 10.1021/acs.orglett.2c01356. 35609004

[ref29] Fan R.; Serrano-Plana J.; Oloo W. N.; Draksharapu A.; Delgado-Pinar E.; Company A.; Martin-Diaconescu V.; Borrell M.; Lloret-Fillol J.; Garcia-Espana E.; Guo Y.; Bominaar E. L.; Que L. Jr.; Costas M.; Munck E. Spectroscopic and DFT Characterization of a Highly Reactive Nonheme Fe(V)-Oxo Intermediate. J. Am. Chem. Soc. 2018, 140, 3916–3928. 10.1021/jacs.7b11400. 29463085

[ref30] aSarkar S.; Cheung K. P. S.; Gevorgyan V. C–H functionalization reactions enabled by hydrogen atom transfer to carbon-centered radicals. Chem. Sci. 2020, 11, 12974–12993. 10.1039/D0SC04881J. 34123240 PMC8163321

[ref31] ade Visser S. P. Trends in Substrate Hydroxylation Reactions by Heme and Nonheme Iron(IV)-Oxo Oxidants Give Correlations between Intrinsic Properties of the Oxidant with Barrier Height. J. Am. Chem. Soc. 2010, 132, 1087–1097. 10.1021/ja908340j. 20041691

[ref32] aSerrano-Plana J.; Oloo W. N.; Acosta-Rueda L.; Meier K. K.; Verdejo B.; Garcia-Espana E.; Basallote M. G.; Munck E.; Que L. Jr; Company A.; Costas M. Trapping a Highly Reactive Nonheme Iron Intermediate That Oxygenates Strong C-H Bonds with Stereoretention. J. Am. Chem. Soc. 2015, 137, 15833–15842. 10.1021/jacs.5b09904. 26599834

[ref33] aKopinke F. D.; Georgi A. What Controls Selectivity of Hydroxyl Radicals in Aqueous Solution? Indications for a Cage Effect. J. Phys. Chem. A 2017, 121, 7947–7955. 10.1021/acs.jpca.7b05782. 28956919

[ref34] Bigi M. A.; Reed S. A.; White M. C. Directed metal (oxo) aliphatic C-H hydroxylations: overriding substrate bias. J. Am. Chem. Soc. 2012, 134, 9721–9726. 10.1021/ja301685r. 22607637

[ref35] See Y. Y.; Herrmann A. T.; Aihara Y.; Baran P. S. Scalable C-H Oxidation with Copper: Synthesis of Polyoxypregnanes. J. Am. Chem. Soc. 2015, 137, 13776–13779. 10.1021/jacs.5b09463. 26466196 PMC5287264

[ref36] aFont D.; Canta M.; Milan M.; Cussó O.; Ribas X.; Gebbink R. J. M. K.; Costas M. Readily Accessible Bulky Iron Catalysts exhibiting Site Selectivity in the Oxidation of Steroidal Substrates. Angew. Chem., Int. Ed. 2016, 55, 5776–5779. 10.1002/anie.201600785. 27059402

[ref37] aBreslow R.; Huang Y.; Zhang X.; Yang J. An artificial cytochrome P450 that hydroxylates unactivated carbons with regio- and stereoselectivity and useful catalytic turnovers. Proc. Natl. Acad. Sci. U.S.A. 1997, 94, 11156–11158. 10.1073/pnas.94.21.11156. 9326577 PMC23399

[ref38] Shokova E. A.; Kim J. K.; Kovalev V. V. Camphor and its derivatives. Unusual transformations and biological activity. Russ. J. Org. Chem. 2016, 52, 459–488. 10.1134/S1070428016040011.

[ref39] aGormisky P. E.; White M. C. Catalyst-controlled aliphatic C-H oxidations with a predictive model for site-selectivity. J. Am. Chem. Soc. 2013, 135, 14052–14055. 10.1021/ja407388y. 24020940

[ref40] aJohnson E. R.; Keinan S.; Mori-Sánchez P.; Contreras-García J.; Cohen A. J.; Yang W. Revealing Noncovalent Interactions. J. Am. Chem. Soc. 2010, 132, 6498–6506. 10.1021/ja100936w. 20394428 PMC2864795

[ref41] aParyzek Z.; Skiera I. Synthesis and cleavage of lactones and tiolactones. Applications in organic synthesis. A review. Org. Prep. Proced. Int. 2007, 39, 203–296. 10.1080/00304940709356015.

[ref42] Ouyang Y.; Peng Y.; Li W.-D. Z. Nickel-mediated reductive coupling of neopentyl bromides with activated alkenes at room temperature and its synthetic application. Tetrahedron 2019, 75, 4486–4496. 10.1016/j.tet.2019.06.034.

[ref43] Nonoptimized oxidation of pivalic acid using the Mn(^TIPS^pdp) catalyst under standard conditions delivers 5% of the corresponding β-lactone.

