# Supplementary material for: Carboxylic Acid Directed γ-Lactonization of Unactivated Primary C–H Bonds Catalyzed by Mn Complexes: Application to Stereoselective Natural Product Diversification
Source: J Am Chem Soc. 2022 Oct 13;144(42):19542–58. doi: 10.1021/jacs.2c08620 (PMC9619407; doi:10.1021/jacs.2c08620)
Supplement: Supplementary file 1 — ja2c08620_si_001.pdf [file ja2c08620_si_001.pdf]

Supporting Information for

Carboxylic Acid Directed γ-Lactonization of Unactivated Primary C-H Bonds Catalyzed by Mn Complexes. Application to Stereoselective Natural Product Diversification

Arnau Call,[a] Marco Cianfanelli,[a] Pau Besalú-Sala,[a] Giorgio Olivo,[a,b] Andrea Palone,[a] Laia Vicens,[a] Xavi Ribas,[a] Josep M. Luis,[a,] Massimo Bietti,[c,]* Miquel Costas [a,]**

[a]*Institut de Química Computacional i Catàlisi (IQCC) and Departament de Química, Universitat de Girona, Campus Montilivi, Girona E-17071, Catalonia, Spain.*

[b]*Current address: Dipartimento di Chimica, Università "La Sapienza", Piazzale Aldo Moro 5, 00185 Rome, Italy.*

[c]*Dipartimento di Scienze e Tecnologie Chimiche, Università "Tor Vergata", Via della Ricerca Scientifica, 1 I-00133 Rome, Italy.*

e-mail: miquel.costas@udg.edu, bietti@uniroma2.it, josepm.luis@udg.edu

Contents

1. Experimental section……........………………………..…..........….........4
1.1. Materials………………………………………………………………..4
1.2. Instrumentation…………………………………………………………4

2. Synthesis of the complexes…………………………………………………...5

3. Synthesis of the substrates…………………………………………………...5
3.1. General alkylation procedure……………………………………………...5
3.2. Synthesis of substrate **11**………………………………………..……......7
3.3. Synthesis of substrate **12**……………………………………………….…..8
3.4. Synthesis of substrate **13**…………………………………………………9
3.5. Synthesis of substrate **15**………………………………………………....11
3.6. Synthesis of substrate **16**…………………………………………..…..12
3.7. Synthesis of deuterated substrates………………………………...…...12
3.8. Synthesis of the ^{18}O-labelled substrates……………………………..……..14

4. Oxidation reactions…………………………………………………………15
4.1. General oxidation protocol A ……………………………………………....15
4.2. General oxidation protocol B ……………………………………………....15
4.3. General oxidation protocol for product isolation……………………..……..15
4.4. Reaction optimization……..16
4.5. Catalyst screening……………………………………………………..18

5. Isotopic labelling experiments………………………………………………...22

6. DFT calculations……………………………………………………………24
6.1. Reaction mechanism……………………………………………………..24
6.1.1. Effective atomic orbitals involved in the O-O formation……………………......24
6.1.2. Spin densities for the relevant atoms ....……………………......25

6.1.3. Energy barriers for the 1,5-HAT and 1,7-HAT for several substrates……….25
6.1.4. Computational determination of KIE…………………………………..……26
6.2. Origin of the site-selectivity…………………………………………..…27
6.3. Origin of the diastereoselectivity……………………………………......29
6.3.1. Scan generation and key distances………………………………………......29
6.3.2. Activation barrier for the HAT on (-)-Camphanic acid with Mn(pdp) catalyst..30
6.4. Computational details for the electronic structure determination……........31
6.5. Cartesian coordinates………………………………………………….....31

7. Characterization of the oxidized products…………………………………...32

8. Lactone derivatization……………………………………………………….......43

9. Characterization of the compounds………………………………………......46
9.1. Solid state structures by X-ray diffraction analisis……………………….....46
9.1.1. Solid state structure of ***12b****………………………………………………............46*
9.1.2. Solid state structure of ***14a****……………………………………………....……......48*
9.1.3. Solid state structure ***15a****……………………………………………………….........50*
9.1.4. Solid state structure of ***16a****……………………………………………………........52*
9.1.5. Solid state structure of ***16b****……………………………………………………........54*
9.1.6. Solid state structure of ***16c****…………………………………………………............56*
9.1.7. Solid state structure of ***17b****…………………………………………..………............58*
9.1.8. Solid state structure of ***18a****…………………………………………..……….........60*
9.1.9. Solid state structure of ***18b****…………………………………………………............62*
9.1.10. Solid state structure of ***19a****…………………………………….……............64*
9.1.11. Solid state structure of ***19b****………………………………..……….......……66*
9.1.12. Solid state structure of ***19b-I****……………………………………………….........68*
9.2. NMR spectra………………………………………………………….……........70
10. References………………………………………………………….……....174

1. Experimental Section

1.1. Materials

Reagents and solvents used were of commercially available reagent quality unless stated otherwise. 2,2,2-Trifluoroethanol (TFE) and 1,1,1,3,3,3-Hexafluoro-2-propanol (HFIP) were purchased from Fluorochem while the other solvents were purchased from SDS and Scharlab. Anhydrous solvents were purified and dried by passing through an activated alumina purification system (M-Braun SPS-800**).** Hydrogen peroxide solutions employed in the oxidation reactions were prepared by diluting commercially available hydrogen peroxide (50% H_2O_2 solution in water, Aldrich). Commercially available trifluoromethanesulfonic acid (TfOH, 99%) was purchased from Fluorochem and used as received. $H_2{}^{18}O$ (97% ^{18}O) was obtained from Fluorochem.

1.2. Instrumentation

Oxidation products were identified by comparison of their GC retention times and GC/MS with those of authentic compounds, and by ^1H and ^{13}C-NMR analyses. X-ray diffraction analysis were carried out on a BRUKER SMART APEX CCD diffractometer using graphite-monochromated MoKα radiation (λ = 0.71073 Å) from an X-ray Tube. NMR spectra were taken on BrukerDPX300 and DPX400 spectrometers using standard conditions. Electrospray ionization mass spectrometry (ESI-MS) experiments were performed on a Bruker Daltonics Esquire 3000 Spectrometer using a 1 mM solution of the analyzed compound. High resolution mass spectra (HRMS) were recorded on a Bruker MicroTOF-Q^{II} (Q-TOF) instrument with a ESI source at Serveis Tècnics of the University of Girona. Samples were introduced into the mass spectrometer ion source by direct infusion through a syringe pump and were externally calibrated using sodium formate. Chromatographic analyses were performed on an AgilentGC-7820-A chromatograph using a HP5 column (30 m).

2. Synthesis of the complexes

The complexes [(*S*,*S*)-Fe(OTf)$_2$(pdp)],[1] [(*S*,*S*)-Mn(OTf)$_2$(pdp)],[2] [(*S*,*S*)-Mn(OTf)$_2$(^{DMM}pdp)],[3] [(*S*,*S*)-Mn(OTf)$_2$(^{TMS}pdp)],[4] [(*S*,*S*)-Mn(OTf)$_2$(^{TIPS}pdp)],[5] [(*S*,*S*)-Mn(OTf)$_2$(^{TIBS}pdp)],[4] [(*S*,*S*)-Mn(OTf)$_2$(^{TIPS}mcp)],[5] were prepared according to the reported procedures.

3. Synthesis of the substrates

The following substrates were commercially available and purchased by Fluorochem and Sigma-Aldrich and used as received.

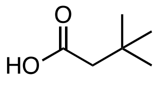

1

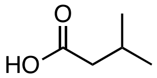

2

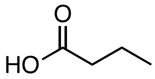

3

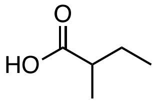

4

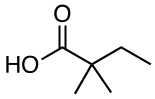

5

3.1. General alkylation procedure

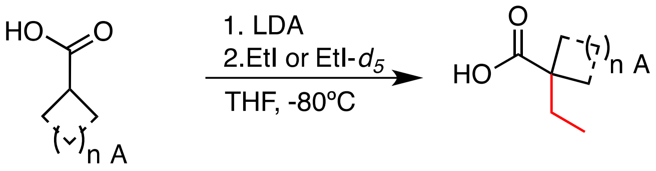

A solution of the corresponding carboxylic acid (5.0 mmol, 1.0 equiv) in THF (2 mL) was added dropwise to a lithium diisopropylamide (1 M in THF, 11 mmol, 2.2 equiv) at –80 °C (AcOEt/N_2 bath). The resulting solution was allowed to warm to room temperature and stirred for 1 h. After the addition of iodoethane or iodoethane-d_5 (99.5% atom D, Sigma-Aldrich) in the case of deuterated substrates (5.0 mmol, 1.0 equiv) in THF (2 mL) at -80 °C, the reaction mixture was stirred for 12 h at room temperature, then poured into 2 N HCl (10 mL) and extracted with EtOAc. The combined organic layers were washed with brine, dried over anhydrous $MgSO_4$, filtered and concentrated. Purification by silica gel flash chromatography with ethyl acetate:hexane eluent mixture provided the pure carboxylic acid.

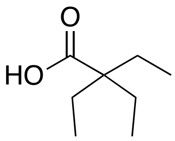

6: Following the *general alkylation procedure*, the crude mixture was purified by column chromatography (SiO_2, hexane:EtOAc 20:1) affording the desired product as a white solid (58% yield).

^{1}H-NMR (400 MHz, $CDCl_3$) δ, ppm: 11.37 (bs, 1H), 1.60 (q, *J* = 7.5 Hz, 6H), 0.81 (t, *J* = 7.5 Hz, 9H).

^{13}C-NMR (101 MHz, $CDCl_3$) δ, ppm: 183.4, 49.8, 26.0, 8.28.

HRMS (ESI-) *m/z* calculated for $C_8H_{16}O_2$ $[M-H]^-$ 143.1072, found 143.1085.

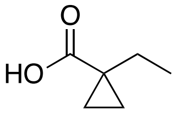

7 Following the *general alkylation procedure*, the crude mixture was purified by column chromatography (SiO_2, hexane:EtOAc 20:1) affording the desired product as a yellowish semi solid (16% yield).

^{1}H-NMR (400 MHz, $CDCl_3$) δ, ppm: 11.86 (bs, 1H), 1.56 (q, *J* = 7.3 Hz, 2H), 1.26 (q, *J* = 3.9 Hz, 2H), 1.01 (t, *J* = 7.3 Hz, 3H), 0.78 – 0.72 (q, 2H).

^{13}C-NMR (101 MHz, $CDCl_3$) δ, ppm: 182.1, 26.5, 24.3, 16.2, 11.7.

HRMS (ESI-) *m/z* calculated for $C_6H_{10}O_2$ $[M-H]^-$ 113.0603, found 113.0611.

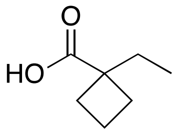

8: Following the *general alkylation procedure*, the crude mixture was purified by column chromatography (SiO_2, hexane:EtOAc 20:1) affording the desired product as a yellowish semi solid (32% yield).

^{1}H-NMR (400 MHz, $CDCl_3$) δ, ppm: 11.39 (bs, 1H), 2.54 – 2.35 (m, 2H), 1.97 – 1.86 (m, 4H), 1.83 (q, *J* = 7.4 Hz, 2H), 0.88 (t, *J* = 7.4 Hz, 3H).

^{13}C-NMR (101 MHz, $CDCl_3$) δ, ppm: 183.84, 48.12, 30.54, 29.51, 15.41, 9.04.

HRMS (ESI-) *m/z* calculated for $C_7H_{12}O_2$ $[M-H]^-$ 127.0759, found 127.0764.

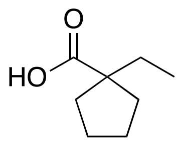

9: Following the *general alkylation procedure*, the crude mixture was purified by column chromatography (SiO_2, hexane:EtOAc 20:1) affording the desired product was obtained as a white solid (51% yield).

^{1}H-NMR (400 MHz, $CDCl_3$) δ, ppm: 11.71 (bs, 1H), 2.20 – 2.05 (m, 2H), 1.74 – 1.58 (m, 6H), 1.50 (m, 2H), 0.90 (t, J = 7.4 Hz, 3H).

^{13}C-NMR (101 MHz, $CDCl_3$) δ, ppm: 184.67, 184.61, 54.46, 35.61, 31.74, 25.11, 10.20.

HRMS (ESI-) *m/z* calculated for $C_8H_{14}O_2$ $[M-H]^-$ 141.0916, found 141.0919.

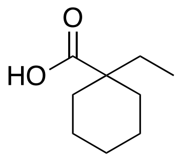

10: Following the *general alkylation procedure*, the crude mixture was purified by column chromatography (SiO_2, hexane:EtOAc 20:1) affording the desired product was obtained as a white solid (68% yield).

^{1}H-NMR (400 MHz, $CDCl_3$) δ, ppm: 11.26 (b, 1H), 2.12 – 2.00 (m, 2H), 1.58 (m, 5H), 1.41 (m, 2H), 1.30 – 1.15 (m, 3H), 0.86 (t, J = 7.5 Hz, 3H).

^{13}C-NMR (101 MHz, $CDCl_3$) δ, ppm: 183.05, 47.18, 33.52, 33.04, 25.97, 23.22, 8.49.

HRMS (ESI-) *m/z* calculated for $C_9H_{16}O_2$ $[M-H]^-$ 155.1072, found 155.1077.

3.2. Synthesis of substrate 11

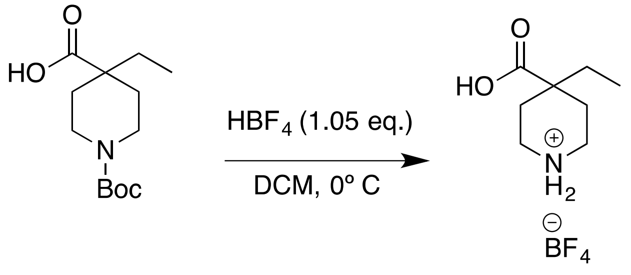

11

1-(*tert*-butoxycarbonyl)-4-ethylpiperidine-4-carboxylic acid (103 mg, 0.4 mmol) was dissolved in anhydrous CH_2Cl_2 (4 mL) under nitrogen atmosphere at 0 ºC. Then, a solution of $HBF_4{\cdot}Et_2O$ (0.42 mmol, 1.05 eq.) in anhydrous CH_2Cl_2 (1 mL) was added dropwise. After 5 minutes, the solvent was evacuated under reduced pressure. The off-white solid was washed with Et_2O (4 mL) multiple times and then dried under high

vacuum for 2 hours. Recrystallization from hot MeCN afforded **11** as white solid (95% yield), which was used directly in the oxidation reaction.

^{1}H-NMR (400 MHz, CD_3CN) δ, ppm: 6.46 (bs, 1H), 3.34-3.28 (m, 2H), 3.00-2.94 (m, 2H), 2.24-2.20 (m, 2H), 1.65-1.46 (m, 4H), 0.85 (t, *J* = 7.5 Hz, 3H).

^{13}C-NMR (101 MHz, CD_3CN) δ, ppm: 175.78, 44.84, 43.25, 32.97, 30.12, 8.42.

HRMS (ESI+) *m/z* calculated for $C_8H_{16}NO_2$ $[M]^+$ 158.1176, found 158.1181.

3.3. Synthesis of substrate 12

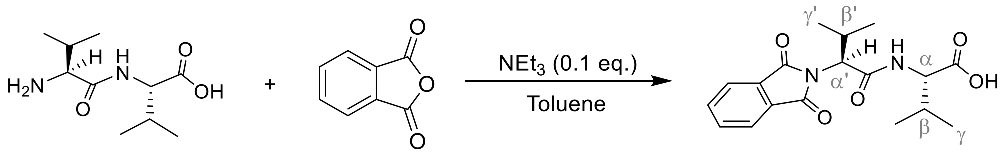

12

Synthesis of **12** was performed according to a reported procedure[6] using 522.1 mg of H-Val-Val-OH (2.38 mmol, 1 equiv.), 357.6 mg of phthalic anhydride (2.38 mmol, 1 equiv.) and 35 μL of Et_3N (0.238 mmol, 0.1 equiv.). The product was purified by column chromatography (hexane:EtOAc, 1:1) and obtained as a white solid (315.0 mg, 38% yield).

^{1}H-NMR (400 MHz, $CDCl_3$), δ: 7.87 (dd, *J* = 5.5, 3.0 Hz, $2H_{Phth}$), 7.75 (dd, *J* = 5.5, 3.0 Hz, $2H_{Phth}$), 7.51 (d, *J* = 8.5 Hz, $1H_{NH}$), 4.49 (dd, *J* = 8.5, 4.6 Hz, $1H_{\alpha}$), 4.45 (d, *J* = 11.4 Hz, $1H_{\alpha'}$), 2.95 – 2.74 (m, $1H_{\beta'}$), 2.35 – 2.14 (m, $1H_{\beta}$), 1.10 (d, *J* = 6.6 Hz, $1H_{\gamma'}$), 0.99 (d, *J* = 7.1 Hz, $1H_{\gamma}$), 0.96 (d, *J* = 7.1 Hz, $1H_{\gamma}$), 0.86 (d, *J* = 6.6 Hz, $1H_{\gamma'}$).

^{13}C-NMR (100 MHz, $CDCl_3$), δ: 175.5 (C_{COOH}), 169.6 ($C_{C=O}$), 168.7 ($C_{C=O}$), 134.62 ($2C_{Phth}$), 131.4 ($2C_{Phth(q)}$), 123.9 ($2C_{Phth}$), 63.4 ($C_{\alpha'}$), 57.4 (C_{α}), 30.7 (C_{β}), 28.0 ($C_{\beta'}$), 19.9 ($C_{\gamma'}$), 19.6 ($C_{\gamma'}$), 19.3 (C_{γ}), 17.7 (C_{γ}).

HRMS (ESI+) *m/z* calculated for $C_{18}H_{22}N_2O_5$ $[M+Na]^+$ 369.1421, found 369.1436.

3.4. Synthesis of substrate 13

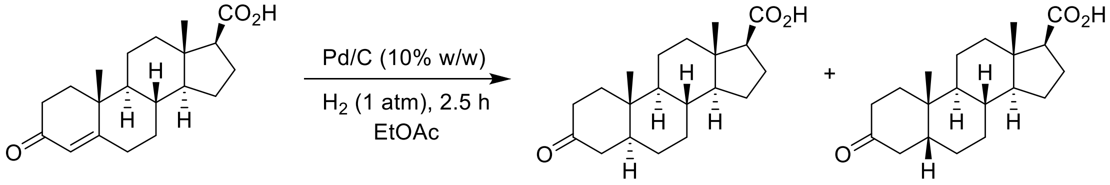

3-keto-4-etiocholenic acid (**14**) **13** **13'**

Mixture of diastereoisomers

3-keto-4-etiocholenic acid (**14**) (400 mg, 1.26 mmol) was added to a suspension of Pd(0) on charcoal (10% w/w, 40 mg) in ethyl acetate (150 mL) under a nitrogen atmosphere. The flask was subsequently purged with hydrogen and the reaction mixture was stirred at room temperature under hydrogen atmosphere (1 atm) for 2.5 h. After that, the reaction mixture was filtered through Celite and the solvent was removed under reduced pressure. Purification by flash chromatography over silica gel (hexane:EtOAc:MeOH 8:1:1) affords the product as a non-separable mixture of diastereoisomers (**13** + **13'**) as a white solid (0.35 g. 1.10 mmol, 87% yield).

13 and **13'**: ^{13}C-NMR (101 MHz, $CDCl_3$) δ, ppm: 213.55, 212.32, 179.99, 179.93, 56.01, 55.84, 55.30, 55.26, 53.74, 46.67, 44.63, 44.37, 44.24, 42.30, 40.81, 38.55, 38.31, 38.13, 37.17, 37.02, 35.73, 35.56, 34.97, 31.69, 28.86, 26.56, 25.83, 24.49, 23.49, 23.43, 22.65, 21.30, 21.06, 13.40, 11.49.

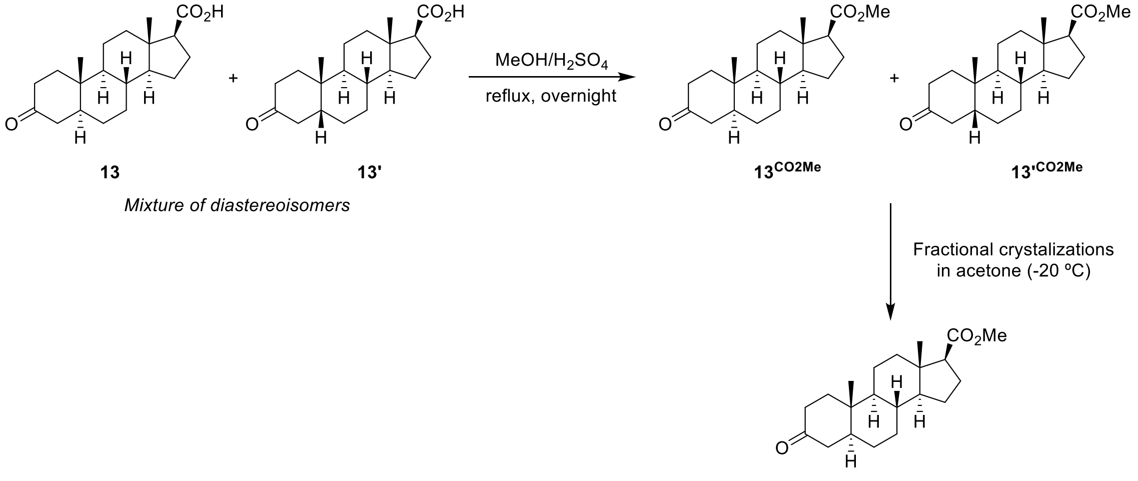

13^{CO2Me}

Diasteroisomers **13** and **13'** (0.35 g, 1.10 mmol) obtained in the previous hydrogenation reaction were dissolved in MeOH (200 mL) and H_2SO_4 (1 mL) was added at room temperature. The mixture was refluxed overnight. After this time, the solvent was removed under reduced pressure. The residue was dissolved in CH_2Cl_2 (20 mL), washed with brine and extracted with CH_2Cl_2 (2 x 20 mL). The combined organic layers were then dried over $MgSO_4$ and concentrated to give the mixture of the estified compounds

13^{CO2Me} and **13'^{CO2Me}** (0.35 g, 1.05 mmol, 96% yield). Fractional crystalizations of the solid residue from acetone at -20 ºC furnished 30 mg of the isomer **13^{CO2Me}** in a pure form.

^{1}H-NMR (400 MHz, $CDCl_3$) δ, ppm: 3.68 (s, 3H), 2.43-2.24 (m, 4H), 2.18-2.00 (m, 4H), 1.86-1.51 (m, 5H), 1.45-1.24 (m, 7H), 1.16-0.95 (m, 5H), 0.82-0.75 (m 1H), 0.69 (s, 3H).

^{13}C-NMR (101 MHz, $CDCl_3$) δ, ppm: 212.00, 174.64, 55.82, 55.40, 53.88, 51.35, 46.81, 44.79, 44.26, 38.68, 38.43, 38.26, 35.84, 35.66, 31.82, 28.98, 24.62, 23.77, 21.41, 13.68, 11.59.

HRMS (ESI+) *m/z* calculated for $C_{21}H_{32}O_3$ $[M+Na]^+$ 355.2244, found 355.2253.

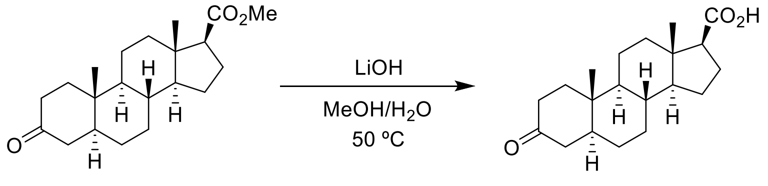

To a solution of **13^{CO2Me}** (26 mg, 0.078 mmol) in MeOH:H_2O (6:3 mL), lithium hydroxide monohydrate (20 mg, 0.48 mmol, 6.1 eq.) was added and the mixture was stirred at 50 ºC for 2 weeks. The reaction was monitored by TLC. The mixture was concentrated to remove the organic solvent, and then water (20 mL) was added to the mixture. The aqueous layer was washed with AcOEt (3 x 20 mL). HCl was then added to the resulting aqueous layer to adjust to pH = 3, and then the mixture was extracted with AcOEt (3 x 20 mL). The combined organic layers were dried over $MgSO_4$ and concentrated to give the desired product **13** as a single diastereoisomer (21.5 mg, 0.067 mmol, 87% yield).

^{1}H-NMR (400 MHz, $CDCl_3$) δ, ppm: 2.43-2.23 (m, 4H), 2.13-1.99 (m, 4H), 1.86-1.77 (m, 1H), 1.75-1.66 (m, 2H), 1.64-1.46 (m, 2H), 1.45-1.22 (m, 8H), 1.16-1.06 (m, 1H), 1.01 (s, 3H), 1.00-0.85 (m, 1H), 0.81-0.71 (m, 4H).

^{13}C-NMR (101 MHz, $CDCl_3$) δ, ppm: 212.17, 179.77, 55.97, 55.28, 53.87, 46.80, 44.78, 44.41, 38.68, 38.25, 35.85, 35.68, 31.81, 28.98, 24.63, 24.60, 23.56, 21.41, 13.52, 11.61.

HRMS (ESI-) *m/z* calculated for $C_{20}H_{30}O_3$ $[M-H]^-$ 317.2111, found 317.2099.

3.5. Synthesis of substrate 15

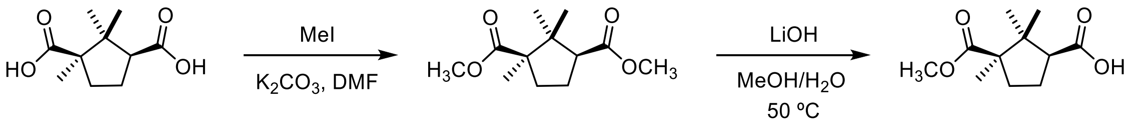

(+)-Camphoric acid

15

To a stirred suspension of (+)-Camphoric acid (4.8 g, 24.3 mmol) and K_2CO_3 (10 g, 73 mmol) in DMF (50 mL) iodomethane (12.6 g, 89 mmol) was added at 15 ºC. Then, the mixture was stirred at room temperature overnight. AcOEt (100 mL) was added to the reaction mixture, and it was washed with water and brine. The organic layer was then dried over $MgSO_4$ and concentrated to give the esterified compound dimethyl (1*R*,3*S*)-1,2,2-trimethylcyclopentane-1,3-dicarboxylate (90 % yield).

^{1}H-NMR (400 MHz, $CDCl_3$) δ, ppm: 3.68 (s, 3H), 3.67 (s, 3H), 2.79 (t, *J* = 8.8 Hz, 1H), 2.62-2.54 (m, 1H), 2.24-2.14 (m, 1H), 1.87-1.76 (m, 1H), 1.54-1.47 (m, 1H), 1.24 (s, 3H), 1.20 (s, 3H)

^{13}C-NMR (101 MHz, $CDCl_3$) δ, ppm: 176.33, 174.60, 56.40, 52.94, 51.68, 51.62, 46.95, 32.68, 23.02, 22.79, 21.79, 21.51.

To a solution of dimethyl (1*R*,3*S*)-1,2,2-trimethylcyclopentane-1,3-dicarboxylate (0.92 g, 3.92 mmol) in MeOH:H_2O (4:2 mL), lithium hydroxide monohydrate (0.167 g, 3.92 mmol) was added and the mixture was stirred at 50 ºC for 3 days. The reaction was monitored by TLC. The mixture was concentrated to remove the solvent, and then water (20 mL) was added to the mixture. The aqueous layer was washed with AcOEt (3 x 20 mL). HCl was then added to the resulting aqueous layer to adjust to pH = 3, and then the mixture was extracted with AcOEt (3 x 20 mL). The combined organic layers were dried over $MgSO_4$ and concentrated to give the desired product (*1S,3R*)-3-(methoxycarbonyl)-1,2,2-trimethylcyclopentane-1-carboxylic acid (**15**) (0.675 g, 3.15 mmol, 80% yield).

^{1}H-NMR (400 MHz, $CDCl_3$) δ, ppm: 11.31 (br, 1H), 3.68 (s, 3H), 2.83 (t, *J* = 8.8 Hz, 1H), 2.63-2.54 (m, 1H), 2.20 – 2.10 (m, 1H), 1.87 – 1.81 (m, 1H), 1.56 – 1.49 (m, 1H), 1.29 (s, 3H), 1.21 (s, 3H), 0.84 (s, 3H).

^{13}C-NMR (101 MHz, $CDCl_3$) δ, ppm: 179.95, 176.25, 56.47, 52.83, 51.74, 47.07, 32.61, 22.88, 22.55, 21.76, 21.30.

HRMS (ESI+) *m/z* calculated for $C_{11}H_{18}O_4$ $[M+Na]^+$ 237.1097, found 237.1102.

3.6. Synthesis of substrate 16

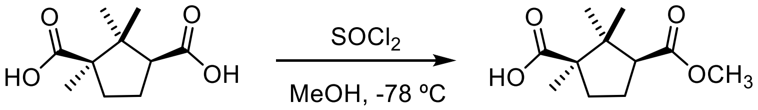

(+)-Camphoric acid **16**

To a solution of (+)-Camphoric acid (1g, 5 mmol) in MeOH (13 mL) at -78 ºC was added $SOCl_2$ (0.4 mL, 5.5 mmol). After stirring at -78 ºC for 30 minutes, the reaction was allowed to warm to room temperature. Stirring was continued for 14 hours. The reaction mixture was concentrated under reduced pressure, yielding a yellow oil. The oil was taken into water. The solution was adjusted to pH 12 with 1 M NaOH and was washed with AcOEt. The basic aqueous layer was acidified with 1 M HCl, and was extracted with AcOEt (3 x 20 mL). The combined organic layers were dried with anhydrous $MgSO_4$, filtered and concentrated under reduced pressure, yieding the desired product (*1R*,*3S*)-3-(methoxycarbonyl)-1,2,2-trimethylcyclopentane-1-carboxylic acid (**16**) as white solid (0.93 g, 4.3 mmol, 87% yield).

^{1}H-NMR (400 MHz, $CDCl_3$) δ, ppm: 10.76 (br, 1H), 3.69 (s, 3H), 2.82 (t, J = 8.8 Hz, 1H), 2.59-2.51 (m, 1H), 2.26-2.16 (m, 1H), 1.88-1.78 (m, 1H), 1.56-1.50 (m, 1H), 1.27 (s, 3H), 1.26 (s, 3H), 0.86 (s, 3H).

^{13}C-NMR (101 MHz, $CDCl_3$) δ, ppm: 181.21, 174.51, 56.18, 52.93, 51.68, 46.86, 32.44, 22.86, 22.70, 21.77, 21.43.

HRMS (ESI+) *m/z* calculated for $C_{11}H_{18}O_4$ $[M+Na]^+$ 237.1097, found 237.1100.

3.7. Synthesis of deuterated substrates

Deuterated **6-d$_{10}$** and **6-d$_5$** were synthetized by alkylation reaction with iodoethane-d_5 (99.5% atom D, Sigma-Aldrich). Deuterium incorporation was evaluated by HRMS analysis.

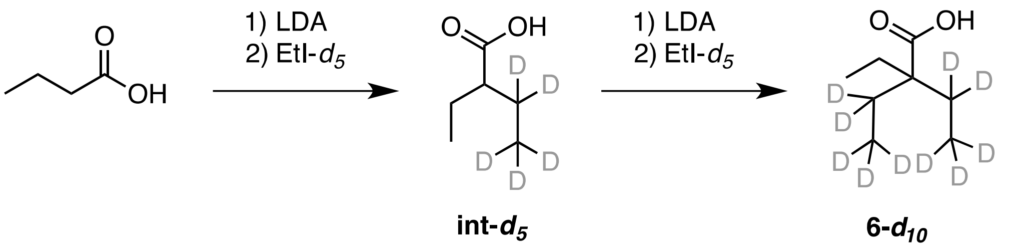

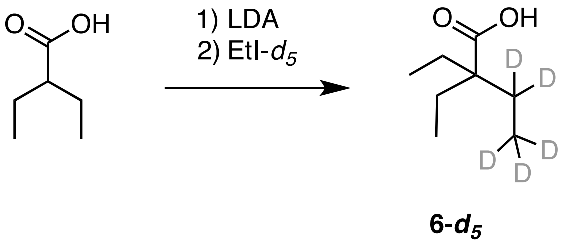

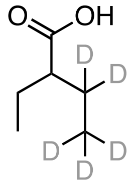

int-*d*$_5$: Following the *general alkylation procedure* the desired compound was obtained from butanoic acid and isolated after column chromatography (SiO_2, hexane:EtOAc 20:1) as a pale yellow oil (43% yield, $d_5 : d_4$ = 98:2).

^{1}H-NMR (400 MHz, $CDCl_3$) δ, ppm: 2.22 (dd, J = 8.4, 5.7 Hz, 1H), 1.72 – 1.50 (m, 2H), 0.94 (t, J = 7.4 Hz, 3H).

^{13}C-NMR (101 MHz, $CDCl_3$) δ, ppm: 182.5, 48.4, 24.7, 11.7.

HRMS (ESI-) *m/z* calculated for $C_6H_7D_5O_2$ $[M-H]^-$ 120.1067, found 120.1072.

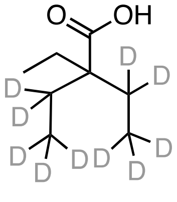

6-*d*$_{10}$: Following the *general alkylation procedure* the desired compound was obtained from **int-*d*$_5$** and isolated after column chromatography (SiO_2, hexane:EtOAc 20:1) as a colorless solid (64% yield, $d_{10} : d_9$ = 97:3).

^{1}H-NMR (400 MHz, $CDCl_3$) δ, ppm: 1.60 (q, J = 7.5 Hz, 2H), 0.81 (t, J = 7.5 Hz, 3H).

^{13}C-NMR (101 MHz, $CDCl_3$) δ, ppm: 183.5, 49.4, 26.0, 8.3.

HRMS (ESI-) *m/z* calculated for $C_8H_6D_{10}O_2$ $[M-H]^-$ 153.1694, found 153.1693.

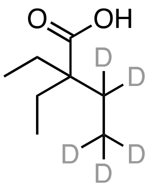

6-d_5: Following the *general alkylation procedure* the desired compound was obtained from 2-ethylbutyric and isolated after column chromatography (SiO_2, hexane:EtOAc 20:1) as a colorless solid (78% yield, $d_5 : d_4$ = 98:2).

^{1}H-NMR (400 MHz, $CDCl_3$) δ, ppm: 1.62 (q, J = 7.5 Hz, 4H), 0.92 – 0.78 (t, J = 7.5 Hz, 6H).

^{13}C-NMR (101 MHz, $CDCl_3$) δ, ppm: 184.1, 49.6, 26.0, 8.3.

HRMS (ESI-) *m/z* calculated for $C_8H_{11}D_5O_2$ $[M-H]^-$ 148.1380, found 148.1385.

3.8. Synthesis of the ^{18}O-labelled substrates

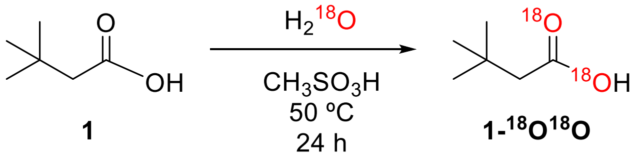

^{18}O-Labelled water (5 eq.) was added dropwise to a stirred suspension of the corresponding carboxylic acid (6 mmol) in MsOH (2 mL) at 50 °C. After 24 h, the clear solution was cooled, treated with ice (3 g), followed by saturated aqueous $(NH_4)_2SO_4$ solution (3 mL). The resulting mixture was extracted with CH_2Cl_2 (3 x 4 mL), the combined extracts were washed with saturated aqueous $(NH_4)_2SO_4$ (3 x 4 mL), dried over Na_2SO_4, and concentrated by rotary evaporation. The isotopically enriched carboxylic acid **1-^{18}O$_2$** was obtained as a colorless oil for which ^{18}O-incorporation was obtained by HRMS analysis.

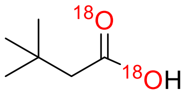

1-^{18}O^{18}O

51% doubly labelled ^{18}O
41% singly labelled ^{18}O
8% unlabelled

4. Oxidation reactions

4.1. General oxidation protocol A

Substrate (100 µmol, 1 molar equivalent) and catalyst (2.0 µmol, 2 mol%) were dissolved in 4.0 mL of TFE ([substrate] = 25 mM) inside a 12-mL vial equipped with a magnetic stirring bar in a water/ice bath. A 0.9 M solution of H_2O_2 in TFE (1.5 molar equivalent) diluted from commercially available H_2O_2 water solution (50% w/w, Sigma Aldrich) was delivered over 30 minutes by syringe pump into the solution under air. At the end of the addition, the mixture was left under stirring for additional 15 minutes to ensure complete lactonization. *Workup for GC analysis:* after quenching the reaction with isopropanol (0.2 mL), 50 µmol of the internal standard (biphenyl) were added and the mixture was quickly filtered through a short plug of silica gel, which was subsequently rinsed with EtOAc. GC analysis of the filtrate provided substrate conversion and product yields relative to the internal standard integration.

4.2. General oxidation protocol B

Substrate (100 µmol, 1 molar equivalent) and catalyst (5.0 µmol, 5 mol%) were dissolved in 4.0 mL of HFIP ([substrate] = 25 mM) inside a 12-mL vial equipped with a magnetic stirring bar in a water/ice bath. A 0.9 M solution of H_2O_2 in HFIP (2.0 molar equivalent) diluted from commercially available H_2O_2 water solution (50% w/w, Sigma Aldrich) was delivered over 30 minutes by syringe pump into the solution under air. At the end of the addition, the mixture was left under stirring for additional 15 minutes to ensure complete lactonization. *Workup for GC analysis:* after quenching the reaction with isopropanol (0.2 mL), 50 µmol of the internal standard (biphenyl) were added and the mixture was quickly filtered through a short plug of silica gel, which was subsequently rinsed with EtOAc. GC analysis of the filtrate provided substrate conversion and product yields relative to the internal standard integration.

4.3. General oxidation protocol for product isolation

Catalysis were performed following the general protocol A or B but in a 0.3-0.5 mmol scale, but without the addition of internal standard. After quenching the reaction with isopropanol, the solvent was removed under reduced pressure and the resultant crude was purified by column chromatography. 1D- and 2D-NMR analyses, HRMS and X-ray diffraction analyses were employed for the characterization of the lactones.

4.4. Reaction optimization

Table S1. Optimization of cat. loading and H_2O_2 equiv. for the oxidation of **1**.

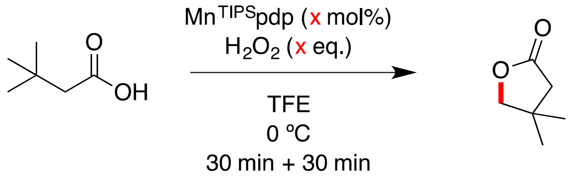

Entry	H_2O_2 (eq.)	Cat. Loading (mol %)	Conversion (%)	Lactone Yield (%)
1	1	1	75	44
2	1.5	1	94	55
**3**	**1.5**	**2**	**96**	**66**
4	2	2	96	61
5	1.5	3	96	65
6	1.5	4	96	65

[a] Reaction conditions and workup as described in General oxidation protocol A. Conversions and yields determined by GC analysis.

Table S2. Optimization of the oxidation of **16**.

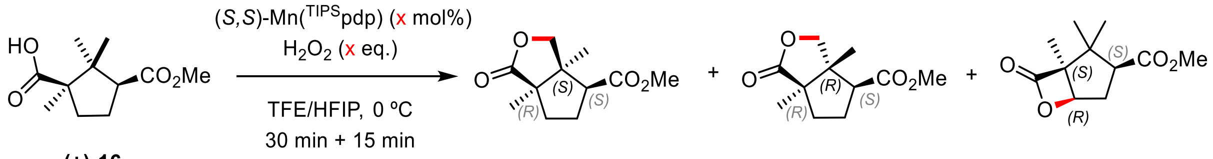

Entry[a]	Cat. loading (mol %)	H_2O_2 (eq.)	Solvent	Conv. (%)	Yield 16a (%)	Yield 16b (%)	Yield 16c (%)	d.r.[b]	r.r.[c]
1	2	1.5	TFE	46	19	<1	5	19 : 1	3.7 : 1
2	3	1.5	TFE	54	19	<1	5	19 : 1	3.7 : 1
3	4	1.5	TFE	57	20	<1	6	20 : 1	3.7 : 1
4	5	2	TFE	70	25	<1	7	25 : 1	3.6 : 1
5	2	2	TFE	60	21	<1	6	21 : 1	3.8 : 1
6[d]	5 x 2	2 x 2	TFE	79	31	<1	9	31 : 1	3.6 : 1
7[d]	5 x 2	1 x 2	TFE	69	23	<1	6	23 : 1	3.7 : 1
8[d,e]	5 x 2	1 x 2	TFE	83	28	<1	7	28 : 1	3.9 : 1
9[f]	5	2	TFE	62	24	<1	6	24 : 1	4.2 : 1
10	2	1.5	HFIP	67	30	<1	7	30 : 1	4.2 : 1
11[f]	2	1.5	HFIP	39	14	<1	4	14 : 1	3.9 : 1
12	5	1.5	HFIP	96	50	<2	12	25: 1	4.3 : 1
13	4	2	HFIP	96	46	<2	11	23 : 1	4.2 : 1
**14**	**5**	**2**	**HFIP**	**100**	**52**	**<2**	**13**	**26 : 1**	**4.3 : 1**

[a] Reaction conditions and workup as described in General oxidation protocol A and B. Conversions and yields determined by GC analysis of crude reaction mixtures. [b] Diastereoisomeric ratio (d.r.) corresponds to **16a**:**16b** ratio. [c] Regioisomeric ratio (r.r.) refers to the ratio of primary over secondary C-H oxidation products (**16a** + **16b**)/(**16c**). [d] Two sequential additions of catalyst and H_2O_2. [e] Reaction performed using 12.5 mM substrate. [f] In the presence of 0.1 eq. TfOH.

4.5. Catalyst screening

Table S3. Catalyst screening for oxidation of **16**.

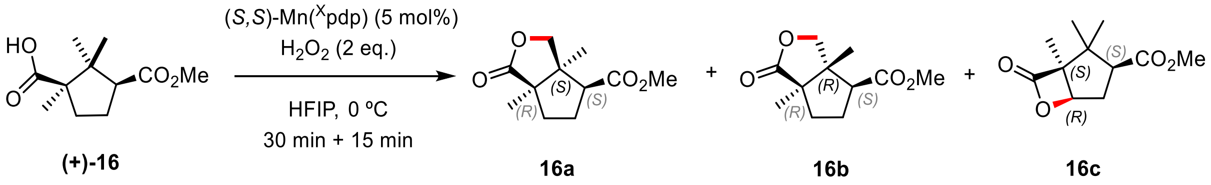

Entry[a]	Cat.	Conv. (%)	Yield 16a (%)	Yield 16b (%)	Yield 16c (%)	d.r.[b]	r.r.[c]
1	(*S*,*S*)-Mn(^{TIPS}pdp)	100	52	<2	13	26 : 1	4.3 : 1
2	(*S*,*S*)-Mn(^{TIBS}pdp)	100	34	1	17	34 : 1	2 : 1
3	(*S*,*S*)-Mn(^{TMS}pdp)	100	32	1	19	32 : 1	1.7 : 1
4	(*S*,*S*)-Mn(^{DMM}pdp)	100	16	1	27	16 : 1	0.6 : 1
5	(*S*,*S*)-Mn(pdp)	100	21	1	24	21 : 1	1 : 1
6	(*S*,*S*)-Mn(^{TIPS}mcp)	90	42	1	7	42 : 1	5.9 : 1

[a] Reaction conditions and workup as described in General oxidation protocol B. Conversions and yields determined by GC analysis of crude reaction mixtures. [b] Diastereoisomeric ratio (d.r.) corresponds to **16a**:**16b** ratio. [c] Regioisomeric ratio (r.r.) refers to the ratio of primary over secondary C-H oxidation products (**16a** + **16b**)/(**16c**).

Table S4. Catalyst screening for oxidation of **16**.

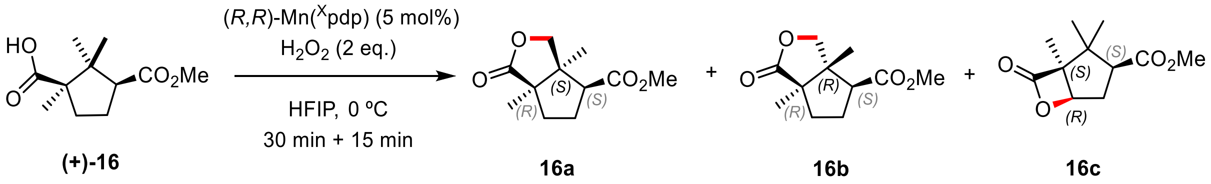

Entry[a]	Cat.	Conv. (%)	Yield 16a (%)	Yield 16b (%)	Yield 16c (%)	d.r.[b]	r.r.[c]
1	(*R*,*R*)-Mn(^{TIPS}pdp)	100	47	39	1	1.2 : 1	86 : 1
2	(*R*,*R*)-Mn(^{TIBS}pdp)	100	42	48	1	0.9 : 1	90 : 1
3	(*R*,*R*)-Mn(^{DMM}pdp)	100	15	67	1	0.2 : 1	82 : 1
5	(*R*,*R*)-Mn(pdp)	100	23	65	16c)1	0.4 : 1	88 : 1
6	(*R*,*R*)-Mn(^{TIPS}mcp)	65	30	17	1	1.7 : 1	47 : 1

[a] Reaction conditions and workup as described in General oxidation protocol B. Conversions and yields determined by GC analysis of crude reaction mixtures. [b] Diastereoisomeric ratio (d.r.) corresponds to **16a**:**16b** ratio. [c] Regioisomeric ratio (r.r.) refers to the ratio of primary over secondary C-H oxidation products (**16a** + **16b**)/(**16c**).

Table S5. Catalyst screening for oxidation of **17**.

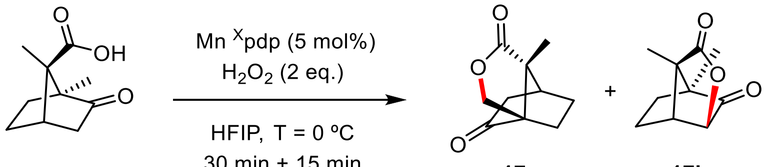

Entry[a]	Cat	Conv (%)	Yield 17a (%)	Yield 17b (%)	r.r.[b]
**1**	**(*S*,*S*)-Mn(^{TIPS}pdp)**	**100**	**1**	**86**	**1 : 86**
**2**	**(*R*,*R*)-Mn(^{TIPS}pdp)**	**100**	**16**	**28**	**1 : 1.7**
3	(*R*,*R*)-Mn(pdp)	100	21	64	1 : 3.1
4	(*R*,*R*)-Mn(^{DMM}pdp)	100	7	66	1 : 9.3
5	(*R*,*R*)-Mn(^{TIPS}mcp)	100	10	17.4	1 : 1.8
5	(*R*,*R*)-Mn(mcp)	100	23	54	1 : 2.4

[a] Reaction conditions and workup as described in General oxidation protocol B. Conversions and yields determined by GC analysis of crude reaction mixtures. [b] [c]regioisomeric ratio (r.r.) refers to the ratio of primary over secondary C-H oxidation products **17a**:**17b**.

Table S6. Catalyst screening for oxidation of **18**.

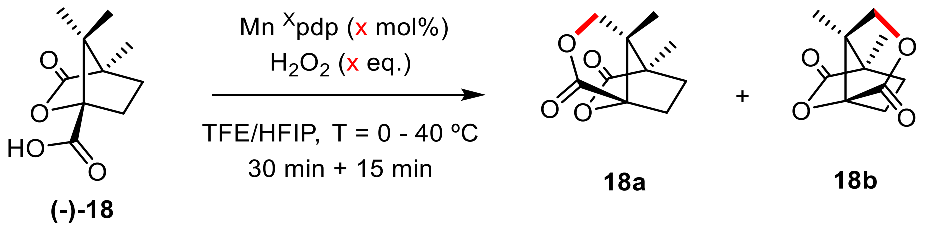

**Entry**[a]	**Cat**	**Cat. loading (mol %)**	**H_2O_2 (eq.)**	**Solvent (T, ºC)**	**Yield 18a (%)**	**Yield 18b (%)**	**d.r.**[b]
1	(*R*,*R*)-Mn(^{TIPS}pdp)	5	2	TFE (0)	<2	n.d.	>100 : 1
2	(*R*,*R*)-Mn(^{TIPS}pdp)	5	2	HFIP (0)	11	n.d.	>100 : 1
3	(*R*,*R*)-Mn(^{TIPS}pdp)	5	4	HFIP (0)	7	n.d.	>100 : 1
3	(*R*,*R*)-Mn(^{TIPS}pdp)	5	6	HFIP (0)	7	n.d.	>100 : 1
4[c]	(*R*,*R*)-Mn(^{TIPS}pdp)	5x2	2x2	HFIP (0)	16	n.d.	>100 : 1
5[c]	(*R*,*R*)-Mn(^{TIPS}pdp)	5x3	2x3	HFIP (0)	21	n.d.	>100 : 1
6	(*R*,*R*)-Mn(^{TIPS}pdp)	5	2	HFIP (20)	22	n.d.	>100 : 1
7	(*R*,*R*)-Mn(^{TIPS}pdp)	5	2	HFIP (30)	30	n.d.	>100 : 1
8	(*R*,*R*)-Mn(^{TIPS}pdp)	5	2	HFIP (40)	37	n.d.	>100 : 1
**9**[c]	**(*R*,*R*)-Mn(^{TIPS}pdp)**	**5x3**	**2x3**	**HFIP (40)**	**56**	**n.d.**	**>100 : 1**
**10**[c]	**(*S*,*S*)-Mn(^{TIPS}pdp)**	**5x3**	**2x3**	**HFIP (40)**	**6**	**54**	**1 : 8.7**
11	(*R*,*R*)-Mn(pdp)	5	2	HFIP (0)	5	n.d.	>100
12	(*S*,*S*)-Mn(pdp)	5	2	HFIP (0)	n.d.	39	1 : >100
**13**	**(*R*,*R*)-Mn(pdp)**	**5x3**	**2x3**	**HFIP (40)**	**50**	**n.d.**	**>100 : 1**
**14**	**(*S*,*S*)-Mn(pdp)**	**5x3**	**2x3**	**HFIP (40)**	**2**	**74**	**1 : 32**

[a]Reaction conditions and workup as described in General oxidation protocol B. Conversions and yields determined by GC analysis of crude reaction mixtures. [b] Diastereoisomeric ratio (d.r.) corresponds to **18a**:**18b** ratio. [c] Sequential additions of catalyst and H_2O_2. n.d.: not detected

Table S7. Catalyst sreeening for oxidation of **19**.

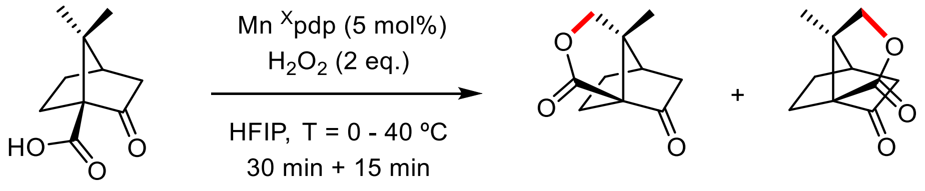

Entry[a]	Cat	Solvent	T (ºC)	Conv (%)	Yield 19a (%)	Yield 19b (%)	d.r.[b]
1	(*R*,*R*)-Mn(^{TIPS}pdp)	HFIP	0	91	5	<1	10 : 1
2	(*R*,*R*)-Mn(^{DMM}pdp)	HFIP	0	76	11	1	9 : 1
**3**	**(*R*,*R*)-Mn(pdp)**	**HFIP**	**0**	**91**	**35**	**3**	**12 : 1**
4	(*R*,*R*)-Mn(^{DMM}pdp)	HFIP	40	100	33	3	10 : 1
5	(*R*,*R*)-Mn(pdp)	HFIP	40	100	36	5	7.4 : 1
6	(*S*,*S*)-Mn(^{TIPS}pdp)	HFIP	0	100	<1	50	1 : >100
**7**	**(*S*,*S*)-Mn(pdp)**	**HFIP**	**0**	**100**	**<1**	**84**	**1 : >100**
8	(*S*,*S*)-Mn(pdp)	HFIP	30	100	<1	82	1 : >100

[a]Reaction conditions and workup as described in General oxidation protocol B. Conversions and yields determined by GC analysis of crude reaction mixtures. [b] Diastereoisomeric ratio (d.r.) corresponds to **19a**:**19b** ratio.

5. Isotopic labelling experiments

Table S8. Intramolecular deuterium Kinetic Isotope Effects (k_H/k_D) in the oxidations of deuterated **6-*d*$_{10}$** (at different reaction times and temperatures) and **6-*d*$_5$**.

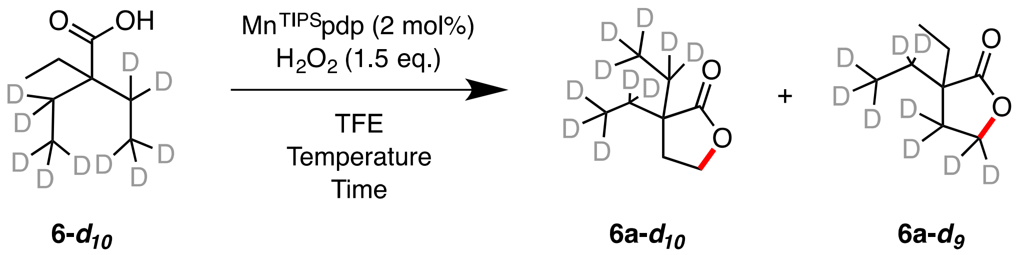

Entry	Time (min)	Temperature (ºC)	Corrected 6a-*d*$_{10}$: 6a-*d*$_9$	k_H/k_D
**1**	30	0	84 :16	10.5
**2**	30	-35	83: 17	9.8
**3**	1	0	82 : 18	9.1
**4**	2	0	83 : 17	9.8
**5**	5	0	83 : 17	9.8
**6**	10	0	82 : 18	9.1
**7**	20	0	83.5 : 16.5	10.1
**8**	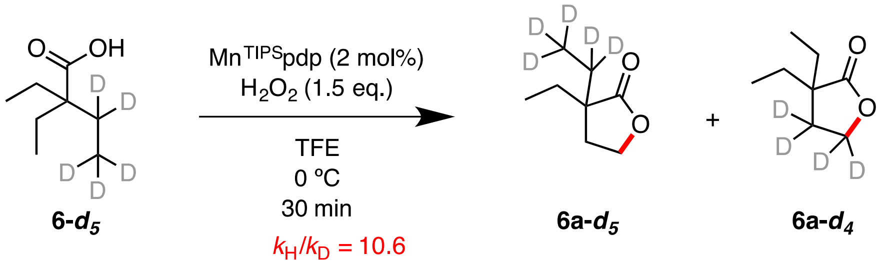			

KIE (k_H/k_D) values were obtained from the d_5/d_4 or d_{10}/d_9 lactone ratios, which in turn come from HRMS or GC-MS (CI source) and are corrected for the isotopic purity of the deuterated acid substrate.

Table S9. The effect of catalyst, solvent and TfOH on the rebound step to yield **1a**.

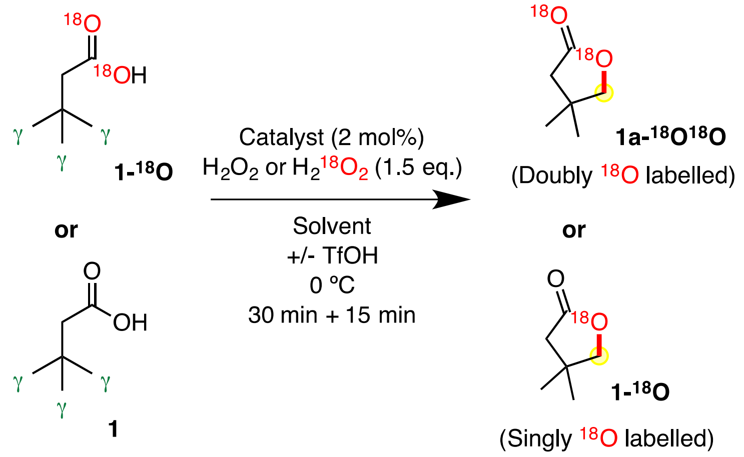

Entry	Catalyst	Solvent	TfOH (eq.)	Lactone Yield (%)	$^{18}O^{18}O$ incorporation (%)
**1**	Mn(pdp)	TFE	-	54	>99
**2**	Mn(^{TIBS}pdp)	“	-	54	>99
**3**	Mn(^{TIPS} pdp)	“	-	65	>99
**4**	“ (with $H_2{}^{18}O_2$)		-	64	0 (0% singly ^{18}O)
**5**	“	MeCN	-	11	>99
**6**	“	HFIP	-	64	>99
**7**	“	TFE	0.1	62	>99
**8**	Mn(^{TIPS}mcp)	“	-	41	>99
**9**	Mn(^{DMM}mcp)	“	-	29	>99
**10**	Mn(^{NMe2}mcp)	“	-	17	98
**11**	Fe(pdp)	“	-	25	73
**12**	Fe(^{TIPS}pdp)	“	-	6	86
**13**	“ (with $H_2{}^{18}O_2$)	“	-	10	0 (15% singly ^{18}O)
**14**	“	“	0.1	6	72

^{18}O-atom incorporated into the lactones was calculated on the basis of GC−MS analysis (via chemical ionization with NH_3/CH_4). The percentage of carboxylate rebound corresponds to the percentage of the doubly ^{18}O-labelled lactone. The reported ^{18}O incorporations are obtained after correction for the isotopic purity of the labelled reactants. In the experiments with labelled $H_2{}^{18}O_2$, non-labelled **1** was used.

6. DFT calculations

6.1. Reaction mechanism

6.1.1. Effective atomic orbitals involved in the O-O formation

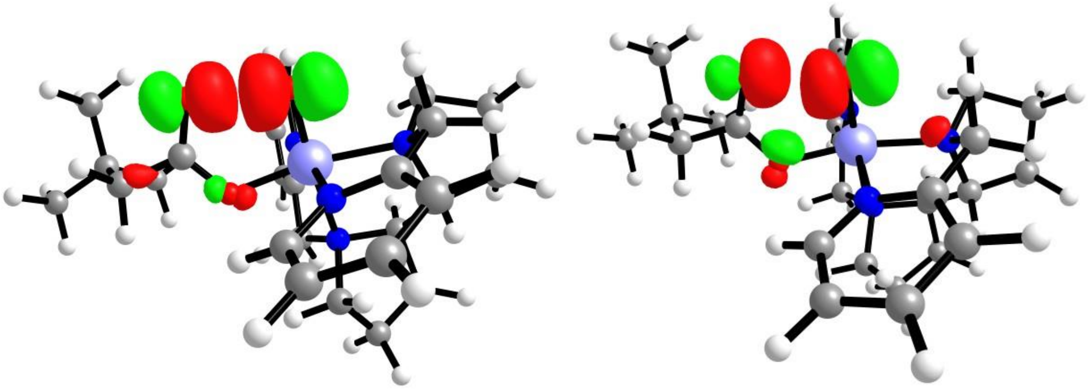

Figure S1. Positive and negative isocontours of the alpha (left) and beta (right) effective atomic orbitals involved in the O-O interaction between the oxyl and the oxygen of the carbonyl.

The occupancies for the alpha effective atomic spinorbitals depicted in Figure S1 are 0.65 and 0.36 electrons for the carbonyl and oxyl oxygens respectively, and therefore their linear combination will generate an occupied σ and an unoccupied σ* spinorbitals. On the other hand, the occupations for the beta effective atomic spinorbitals are 0.95 and 0.78 for the carbonyl and oxyl oxygens respectively, and therefore, in this case, their linear combination will generate an occupied σ and an occupied σ* spinorbitals. The addition of the alpha and beta analysis characterizes this bond as a single bond formed by the alpha electron, in perfect agreement with the -0.33 and -0.23 spin density found in the O-O contact, and its bond order of 0.5.

6.1.2. Spin densities for the relevant atoms

In Table S10 we report the spin densities of the key atoms involved in the 1,7-HAT reactivity of all intermediates along the reaction profile of Figure 3B of the manuscript.

Table S10. Spin densities of Mn, O_{oxyl}, O_1, O_2 and C_1 atoms along the 1,7-HAT reaction coordinate.

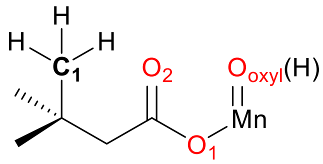

Intermediate	Mn	O_{oxyl}(H)	O_1	O_2	C_1
**II_q**	3.993	0.011	0.031	0.012	0.001
**III_t**	2.840	-0.378	-0.034	-0.239	0.001
**IV_t**	3.052	0.134	-0.010	0.102	-1.059
**VI_t^{carb}**	2.054	0.091	-0.008	0.001	0.002
**VII_t^{carb}**	2.051	0.083	-0.013	0.001	0.000

6.1.3. Energy barriers for the 1,5-HAT and 1,7-HAT for several substrates

Table S11. Gibbs free energy barriers for the 1,5- and 1,7-HAT reactions for 3,3-dimethylbutanoic acid, 1-hexanoic acid and 2,2-diethylbutanoic acid computed from **III**. Energy values given in kcal·mol^{-1}.

Substrate	Attacked position	$\Delta G^{\ddagger}$	$\Delta\Delta G^{\ddagger}$
3,3-dimethylbutanoic acid	1,5-HAT<br>1,7-HAT	20.9<br>10.2	10.7
1-hexanoic acid	1,5-HAT<br>1,7-HAT	19.2<br>7.3	11.9
2,2-diethylbutanoic acid	1,5-HAT<br>1,7-HAT	14.7<br>4.9	9.8

For all three substrates, we have focused only in the γ-C-H activation, therefore the 1,5-HAT corresponds to the carbonyl-mediated activation and the 1,7-HAT corresponds to the Mn^{IV}-oxyl activation. In all three cases, the 1,7-HAT is favourable over the 1,5-HAT, consistently indicating that the reaction proceeds directly through the Mn^{IV}-oxyl for both methylenic and primary γ-C-H bond oxidations.

6.1.4. Computational determination of KIE

We have computed the kinetic isotope effect (KIE) for the HAT of the γ-C-H of the 2,2-diethylbutanoic acid (**6**) by computing the chemical barriers for both the non-deuterated (**6**) and decadeuterated (**6-*d*$_{10}$**) substrate, considering both the direct and the carbonyl-assisted activation HAT pathways. We added tunneling corrections to the KIE through the Skodje-Truhlar equation,[7] however such effects were negligible compared with the non-corrected KIE value, and had no effect in the final conclusions.

All the DFT calculations of this study includes the effect of the solvent through the SMD implicit solvent corrections. However, in order to describe more accurately the effect of the solvent in the computation of the KIE, we have also performed calculations adding to our computational model two explicit TFE molecules. Among all different conformations considered, the minimum in the PES is given by the geometry where the acidic proton of both solvent molecules interact with the oxygen of the Mn^{IV}-oxyl moiety (Figure S2). The conformation where the acidic proton of one TFE is forming a hydrogen bond with the oxygen of the Mn^{IV}-oxyl, while the proton for the other TFE is interacting with the oxygen of the carbonyl of the substrate, is not a stationary point of the PES and it relaxes barrierless to the aforementioned minima.

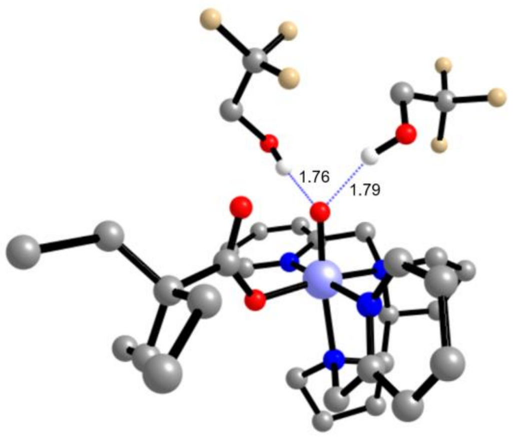

Figure S2. Equilibrium geometry of 2,2-dimethylbutanoate coordinated to Mn(^{TIPS}pdp) including two explicit solvent molecules. TIPS ligand and hydrogens bonded to carbons hidden for clarity.

When the explicit TFE molecules are included in the computational model, the Mn-O bond is slightly enlarged by 0.03Å, and the absolute value of the spin density on oxyl

decreases by 0.02 electrons (from -0.38 to -0.36), which induces a light decrease in the HAT barrier (ΔΔG = 1.4 kcal·mol$^{-1)}$.

Table S12. HAT barriers and KIE for the 2,2-diethylbutanoic acid (**6**) substrate.

**HAT reaction**	**ΔG^{a} (kcal·mol^{-1})**		**ΔG^{b} (kcal·mol^{-1})**	
	**H**	**D**	**H**	**D**
Direct	6.6	7.4	4.9	6.3
Carbonyl-assisted	14.4	15.6	14.5	15.7
KIE direct	3.9		11.6	
KIE Carbonyl	7.6		7.6	
Experimental KIE	10.6			

[a] Reaction modelled including a SMD model potential to simulate the 2,2,2-trifluoroethanol as solvent. [b] Reaction modelled including a SMD model potential to simulate the 2,2,2-trifluoroethanol as solvent, plus two molecules of explicit solvent interacting with the Mn^{IV}-oxyl moiety.

The KIE of the assisted-HAT pathway is computed to be 7.6 and does not change (within two significant digits) upon the addition of the two explicit TFE molecules to the computational model, since none of the solvent molecules are interacting directly with the carbonyl. On the contrary, for the direct-HAT pathway, the inclusion of the two explicit TFE molecules is required to simulate the KIE, since the KIE value increases from 3.9 to 11.6 upon their addition. The latter DFT KIE value is the closest to the experimental value of 10.6, thus increasing the number of evidences indicating that the reaction proceeds through a direct-HAT mechanism.

6.2. Origin of the site-selectivity

The C-H bond dissociation energies (BDE) for the β-, γ-, and δ-methylene units of the hexanoic acid were computed to be 97.2, 96.7 and 96.5 kcal·mol^{-1}, respectively. For their computation, we used the same methodology stated in the *Computational details for the electronic structure determination* section (see below), this is a UB3LYP-D3BJ/Def2TZVPP/SMD //UB3LYP-D3BJ/Def2SVP/SMD model for all the systems, except for the energy of the dissociated hydrogen which was considered as -0.5 Hartree.[8] The BDE values indicate that none of the three C-H cleavages are clearly thermodynamically favored. Therefore, the stereoselectivity is ruled by the kinetics, which have been studied by computing the Gibbs energy barrier for the β- γ- and δ- C-H

bond oxidation. Both pathways (*i.e.* the C-H cleavage by the oxyl of the catalyst core and by the carbonyl of the substrate) have been considered (See Table S13), although consistently with the results presented in the manuscript for the γ-C-H bond cleavage HAT Gibbs energy barriers are always lower for the *oxo* pathway. Furthermore, as it is shown in Figure S3, the cleavage of the γ-C-H is always the most favored one. For the less energetically demanding oxo pathway, the order of the barriers is 7.3 (γ), 10.2 (δ) and 15.7 kcal/mol (β).

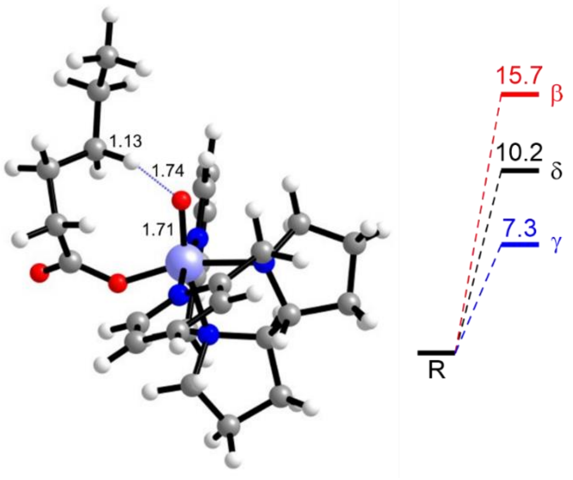

Figure S3. DFT equilibrium geometry of the TS for the *oxo* γ-C-H claevage (left) and the three reaction Gibbs energy barriers for the oxo β- γ- and δ- C-H bond cleavage.

Table S13. Energy barriers for both HAT mechanisms.

HAT mechanism (site)	ΔG (kcal·mol^{-1})
oxo (β)	15.74
oxo (γ)	7.26
oxo (δ)	10.19
carbonyl (β)	27.96
carbonyl (γ)	19.20
carbonyl (δ)	20.07

6.3. Origin of the diastereoselectivity

6.3.1. Scan generation and key distances

The rotational scan was generated at the UB3LYP-D3BJ/Def2SVP level of theory, via a constrained minimization every 2° over the 360° conformational scan, this is a total of 180 constrained optimizations per system. Although the step-size was relatively small, in order to save computational time and obtain a reasonable geometrical guess of the four key structures, a much less dense grid of 72 optimizations (step-size 5°) is enough. The non-covalent interaction presented in the article were obtained with the NCIPLOT package[9] with the standard parameters and removing all the non-covalent interactions other than the ones involving the substrate.

The distance of methyl groups with respect to the closest carbon of the TIPS groups is in clear correspondence with the energy trends observed in the rotational scans (Figure S4). Thus, the most stable conformer appears at larger TIPS-Me distances. Such fact implies that the relative position of the TIPS groups, which depend on the absolute configuration of the catalyst, will determine the catalytic poses of the substrate. Given an enantio-specific most stable conformation, the methyl pointing towards the active center is more likely to be activated since it has unhindered access to the Mn-oxo moiety. On the contrary, the methyl pointing towards the ligand is hindered by the ^{TIPS}pdp, and is more difficult to activate, as the Newman projection models presented in the manuscript also illustrate.

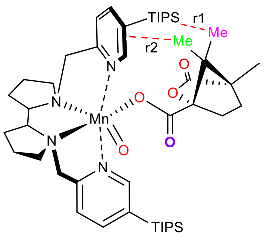

***S,S*-18**^{s} 0.0 kcal/mol
r1=3.96 A
r2=4.20 A
r(Me-O)=3.91 A
r(Me-O)=**3.39** A

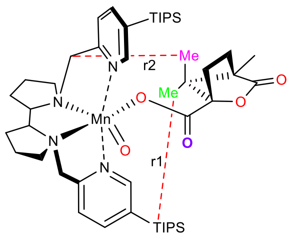

***S,S*-18'** 7.6 kcal/mol
r1=3.77 A
r2=3.77 A
r(Me-O)=**4.26** A
r(Me-O)=4.57 A

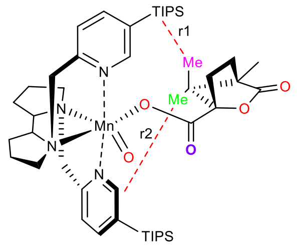

***R,R*-18**^{s} 2.1 kcal/mol
r1=3.98 A
r2=3.75 A
r(Me-O)=**4.18** A
r(Me-O)=4.56 A

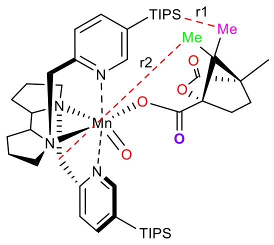

***R,R*-18'** 6.0 kcal/mol
r1=3.80 A
r2=3.69 A
r(Me-O)=4.49 A
r(Me-O)=**3.35** A

Figure S4. Key TIPS-Me distances (in Å) for the ***S,S*-18**^{s}, ***S,S*-18'**, ***R,R*-18**^{s} and ***R,R*-18'** structures.

6.3.2. Activation barrier for the HAT on (-)-Camphanic acid with Mn(pdp) catalyst.

Table S14. Activation barriers (kcal·mol^{-1}) for the oxidation of the (-)-Camphanic acid (**18**) methyl groups by the Mn(pdp) catalyst.

Methyl	(*S*,*S*)-Mn(pdp) barrier (kcal·mol^{-1})	(*R*,*R*)-Mn(pdp) barrier (kcal·mol^{-1})
Generating **18a**	12.6	7.3
Generating **18b**	8.8	12.0

The (*S*,*S*)-Mn(pdp) catalyst favors the formation of **18b** by 3.8 kcal/mol, while the (*R*,*R*)-Mn(pdp) catalyst operates in the opposite direction, thus favoring the formation of **18b** by 4.7 kcal/mol. The reactant complex and TS geometries and energies have been summarized in Figure S5.

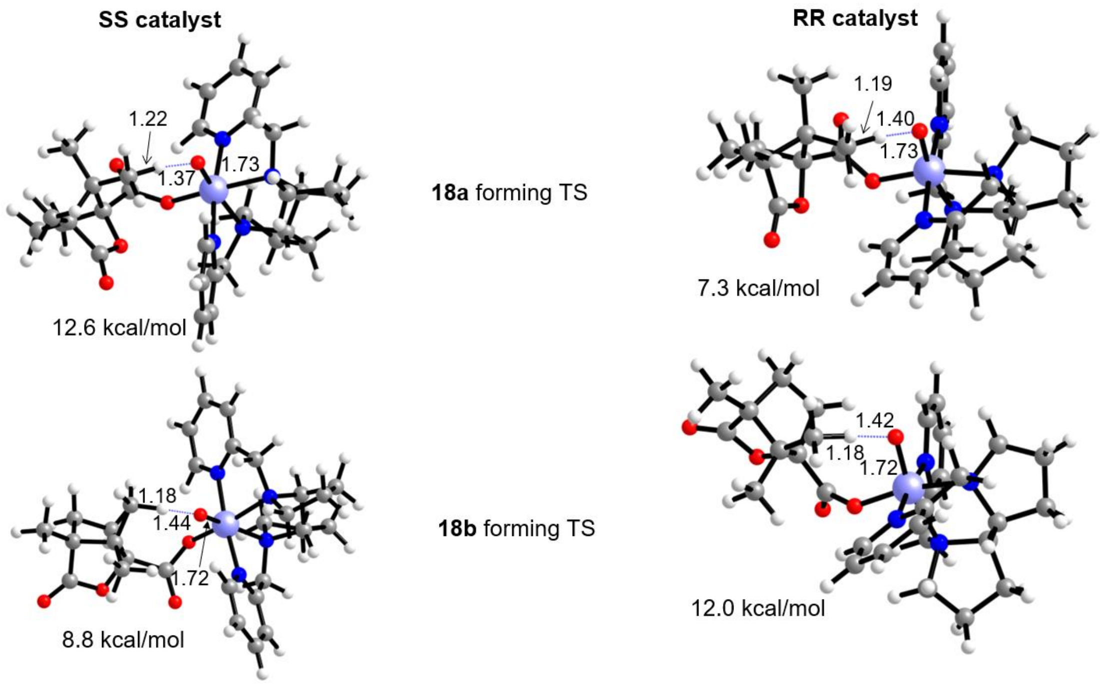

Figure S5. Key geometrical parameters of the TS forming either **18a** or **18b** products for both (*S*,*S*)-Mn(pdp) and (*R*,*R*)-Mn(pdp) catalyst.

6.4. Computational details for the electronic structure determination

All DFT calculations were performed by using the Gaussian16 program.[10] The UB3LYP density functional[11] together with dispersion corrections developed by Grimme and coworkers with the Becke-Jonhson damping[12] and adding solvent (2,2,2-trifluoroethanol) corrections through the Solvation Model based on Density (SMD)[13] was used for geometrical optimizations, expanding the molecular orbitals over a double-ζ basis-set of Ahlrichs and co-workers, Def2SVP.[14] All stationary points in the PES were characterized by means of analytical vibrational frequency calculations and connected through IRC calculations. Single-point electronic energy corrections of reaction intermediates and transition states (TS) were performed by increasing the basis set quality to triple-ζ Def2TZVPP.[15] Therefore, the whole methodology of the study can be denoted as UB3LYP-D3BJ/Def2TZVPP/SMD//UB3LYP-D3BJ/Def2SVP/SMD.

6.5. Cartesian coordinates

All optimized geometries can be accessed at https://iochem.udg.edu:8443/browse/review-collection/100/3673/6ccd87ffa8507cd67c7d6112 onto the IOCHEM-BD platform (www.iochem-bd.org) to facilitate data exchange and dissemination, according to the FAIR principles of OpenData sharing.

7. Characterization of the oxidized products

Oxidation of 1

General oxidation protocol A. The desired product **1a** was obtained after column chromatography (SiO_2, hexane:EtOAc 15:1) as a colorless oil (64% yield).

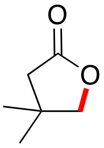

1a: ^{1}H-NMR (400 MHz, $CDCl_3$) δ, ppm: 3.98 (s, 2H), 2.33 (s, 2H), 1.20 (s, 6H).

^{13}C-NMR (101 MHz, $CDCl_3$) δ, ppm: 177.1, 79.6, 43.1, 36.7, 25.8.

HRMS (ESI+) *m/z* calculated for $C_6H_{10}O_2$ $[M+Na]^+$ 137.0578, found 137.0574.

Oxidation of 5

General oxidation protocol A. The desired product **5a** was obtained after column chromatography (SiO_2, hexane:EtOAc 15:1) as a colorless oil (59% yield).

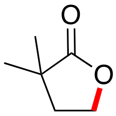

5a: ^{1}H-NMR (400 MHz, $CDCl_3$) δ, ppm: 4.26 (t, *J* = 7.2 Hz, 2 H), 2.11 (t, *J* = 7.2 Hz, 2 H), 1.26 (s, 6 H).

^{13}C-NMR (101 MHz, $CDCl_3$) δ, ppm: 182.3, 64.7, 37.1, 24.2.

HRMS (ESI+) *m/z* calculated for $C_6H_{10}O_2$ $[M+Na]^+$ 137.0578, found 137.0577.

Oxidation of 6

General oxidation protocol A. Pure sample of the desired product **6a** was obtained after column chromatography (SiO_2, hexane:EtOAc 5:1) as a colorless oil (91% yield).

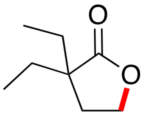

6a: ^{1}H-NMR (400 MHz, $CDCl_3$) δ, ppm: 4.26 (t, *J* = 7.4 Hz, 2H), 2.14 (t, *J* = 7.4 Hz, 2H), 1.66 (q, *J* = 7.5, 1.5 Hz, 4H), 0.96 (t, *J* = 7.5 Hz, 6H).

^{13}C-NMR (101 MHz, $CDCl_3$) δ, ppm: 181.3, 65.2, 46.8, 31.2, 28.5, 8.7.

HRMS(ESI+) *m/z* calculated for $C_8H_{14}O_2$ $[M+Na]^+$ 165.0891, found 165.0881.

Oxidation of 7

General oxidation protocol A. Pure sample of the desired product **7a** was obtained after column chromatography (SiO_2, hexane:EtOAc 15:1) as a pale yellow oil (56% yield).

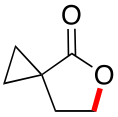

7a: ^{1}H-NMR (400 MHz, $CDCl_3$) δ, ppm: 4.43 (t, *J* = 7.4 Hz, 2H), 2.32 (t, *J* = 7.5 Hz, 2H), 1.26 (m, 2H), 1.01 – 0.96 (m, 2H).

^{13}C-NMR (101 MHz, $CDCl_3$) δ, ppm: 180.2, 65.7, 29.7, 19.7, 15.1.

HRMS (ESI+) *m/z* calculated for $C_6H_8O_2$ $[M+Na]^+$ 135.0422, found 135.0418.

Oxidation of 8

General oxidation protocol A. The desired product **8a** was obtained after column chromatography (SiO_2, hexane:EtOAc 15:1) as a colorless oil (54% yield).

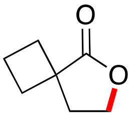

8a: ^{1}H-NMR (400 MHz, $CDCl_3$) δ, ppm: 4.20 (t, *J* = 6.9 Hz, 2H), 2.56 – 2.44 (m, 2H), 2.35 (t, *J* = 6.9 Hz, 2H), 2.20 – 1.99 (m, 4H).

^{13}C-NMR (101 MHz, $CDCl_3$) δ, ppm: 181.3, 65.1, 43.1, 36.0, 30.1, 16.4

HRMS (ESI+) *m/z* calculated for $C_7H_{10}O_2$ $[M+Na]^+$ 149.0578, found 149.0571.

Oxidation of 9

General oxidation protocol A. The lactone products were obtained, after column chromatography (SiO_2, hexane:EtOAc 10:1), as a mixture of isomers. Colorless oil (53% yield).

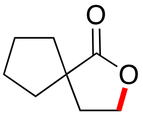

9a

minor product

9a: ^{1}H-NMR (400 MHz, $CDCl_3$) δ, ppm: 4.25 (t, *J* = 6.9 Hz, 2H), 2.14 (t, *J* = 6.9 Hz, 2H), 1.95-2.10 (m, 2H), 1.8-1.9 (m, 2H), 1.65-1.72 (m, 4H).

^{13}C-NMR (101 MHz, $CDCl_3$) δ, ppm: 182.9, 65.5, 48.6, 36.9, 36.6, 25.5.

HRMS (ESI+) *m/z* calculated for $C_8H_{12}O_2$ $[M+Na]^+$ 163.0735, found 163.0730.

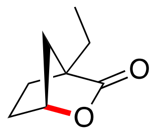

9b

major product **9b:** ^{1}H-NMR (400 MHz, $CDCl_3$) δ, ppm: 4.86 (d, *J* = 2.4 Hz, 1H), 2.06 (dd, *J* = 10.2, 2.3 Hz, 1H), 1.98 – 1.92 (m, 1H), 1.94-1.85 (m, 1H), 1.80 – 1.67 (m, 1H), 1.03 (t, *J* = 7.5 Hz, 3H). 1.81 (ddd, *J* = 12.6, 9.5, 6.4 Hz, 2H), 1.65 – 1.53 (m, 2H).

^{13}C-NMR (101 MHz, $CDCl_3$) δ, ppm: 179.6, 79.2, 52.3, 42.6, 29.5, 27.8, 21.6, 9.8.

HRMS (ESI+) *m/z* calculated for $C_8H_{12}O_2$ $[M+Na]^+$ 163.0735, found 163.0730.

Oxidation of 10

General oxidation protocol A. The lactone products were obtained, after column chromatography (SiO_2, hexane:EtOAc 10:1), as a mixture of isomers. Colorless semisolid (62% yield).

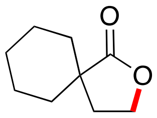

10a

minor product **10a:** ^{1}H-NMR (400 MHz, $CDCl_3$) δ, ppm: 4.27 (t, *J* = 7.1 Hz, 2H), 2.17 (d, *J* = 7.1 Hz, 2H), 1.79 - 1.64 (m, 5H), 1.54 - 1.53 (m, 2H), 1.44 – 1.31 (m, 3H).

^{13}C-NMR (101 MHz, $CDCl_3$) δ, ppm: 182.0, 65.1, 43.1, 33.1, 32.4, 25.3, 22.2.

HRMS (ESI+) *m/z* calculated for $C_9H_{14}O_2$ $[M+Na]^+$ 177.0891, found 177.0893.

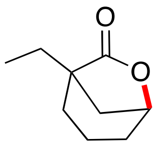

10b

major product **10b:** ^{1}H-NMR (400 MHz, $CDCl_3$) δ, ppm: 4.78 (m, 1H), 2.31 (m, 1H), 2.06 – 1.95 (m, 1H), 1.87 – 1.72 (m, 2H), 1.67 – 1.50 (m, 6H), 0.92 (t, *J* = 7.5 Hz, 3H).

^{13}C-NMR (101 MHz, $CDCl_3$) δ, ppm: 180.1, 75.7, 46.7, 40.2, 32.5, 27.8, 27.0, 18.7, 8.6.

HRMS (ESI+) *m/z* calculated for $C_9H_{14}O_2$ $[M+Na]^+$ 177.0891, found 177.0893.

Oxidation of 11

General oxidation protocol A. The desired product was obtained as the acetyl derivate **11aAc**. After column chromatography (SiO_2, hexane:EtOAc 5:1), the product was obtained as a white solid (46% yield).

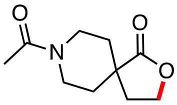

11aAc: ^{1}H-NMR (400 MHz, $CDCl_3$) δ, ppm: δ 4.33 (t, *J* = 7.3 Hz, 2H), 4.09 (m, 1H), 3.84 (m, 1H), 3.35 (m, 2H), 2.28 – 2.17 (m, 2H), 2.11 (s, 3H), 2.00 – 1.83 (m, 2H), 1.65 – 1.54 (m, 2H).

^{13}C-NMR (101 MHz, $CDCl_3$) δ, ppm: 179.9, 168.9, 64.9, 42.8, 41.0, 37.8, 33.9, 32.8, 31.7, 21.4.

HRMS (ESI+) *m/z* calculated for $C_{10}H_{15}NO_3$ $[M+Na]^+$ 220.0950, found 220.0948.

Oxidation of 12

Lactone **12a** was obtained following oxidation protocol A at 25 ºC, with (*S*,*S*)-Mn(^{TIPS}pdp) (2 mol% x 2) and H_2O_2 (3 eq. x 2) and isolated in 21% yield as a white solid after column chromatography (SiO_2, hexane:EtOAc 7:3).

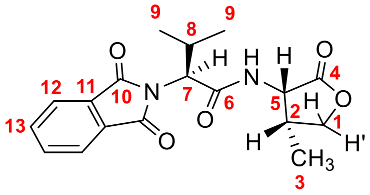

12a

^{1}H-NMR (400 MHz, $CDCl_3$) δ, ppm: 7.91-7.87 (m, 2H, H_{12}), 7.79-7.75 (m, 2H, H_{13}), 7.42 (d, $^3J_{NH\text{-}5}$ = 5.9 Hz, 1H, NH), 4.69 (dd, $^3J_{52}$ = 7.2 Hz, $^3J_{5\text{-}NH}$ = 5.9 Hz, 1H, H_5), 4.50 (d, $^3J_{78}$ = 11.1 Hz, 1H, H_7), 4.44-4.34 (m, 1H, H_1), 4.09 (d, $^2J_{1'1}$ = 9.3 Hz, 1H, $H_{1'}$), 3.04-2.96 (m, 1H, H_2), 2.87 (dq, $^3J_{87}$ = 11.1 Hz, $^3J_{89}$ = 6.7 Hz, 1H, H_8), 1.14 (d, $^3J_{98}$ = 6.7 Hz, 1H, H_9), 0.99 (d, $^3J_{32}$ = 7.2 Hz, 1H, H_3), 0.88 (d, $^3J_{98}$ = 6.7 Hz, 1H, H_9).

^{13}C-NMR (101 MHz, $CDCl_3$) δ, ppm: 174.42 (C_4), 169.62 (C_6), 168.43 (C_{10}), 134.56 (C_{13}), 131.28 (C_{11}), 123.88 (C_{12}), 72.61 (C_1), 62.52 (C_7), 53.27 (C_5), 33.93 (C_2), 27.84 (C_8), 19.97 (C_9), 19.46 (C_9), 12.89 (C_3).

HRMS (ESI+) *m/z* calculated for $C_{18}H_{20}N_2O_5$ $[M+Na]^+$ 367.1264, found 367.1271.

Lactone **12b** was obtained following oxidation protocol A at 25 ºC, with (*S*,*S*)-Mn(^{TIPS}pdp) (2 mol% x 2) and H_2O_2 (3 eq. x 2) and isolated in 25% yield as a white solid after column chromatography (SiO_2, hexane:EtOAc 7:3).

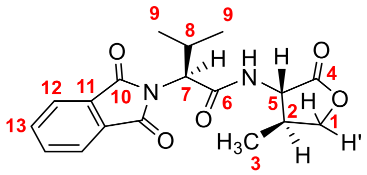

12b

^{1}H-NMR (400 MHz, $CDCl_3$) δ, ppm: 7.89-7.87 (m, 2H, H_{12}), 7.77-7.75 (m, 2H, H_{13}), 7.50 (d, , $^3J_{NH\text{-}5}$ = 7.9 Hz, 1H, NH), 4.51 (d, $^3J_{78}$ = 11.2 Hz, 1H, H_7), 4.43 (dd, $^3J_{1'1}$ = 9.1 Hz, $^3J_{1'2}$ = 7.8 Hz, 1H, $H_{1'}$), 4.38-4.33 (m, 1H, H_5), 3.83 (dd, $^3J_{1'2}$ = 10.6 Hz, $^3J_{11'}$ = 9.1 Hz, 1H, H_1), 2.87-2.78 (m, 1H, H_8), 2.61-2.52 (m, 1H, H_2), 1.21 (d, $^3J_{32}$ = 6.6 Hz, 3H, H_3), 1.14 (d, $^3J_{98}$ = 6.5 Hz, 3H, H_9), 0.87 (d, $^3J_{98}$ = 6.8 Hz, 3H, H_9).

^{13}C-NMR (101 MHz, $CDCl_3$) δ, ppm: 174.48 (C_4), 169.76 (C_6), 168.67 (C_{10}), 134.72 (C_{13}), 131.42 (C_{11}), 124.00 (C_{12}), 71.50 (C_1), 62.93 (C_7), 55.47 (C_5), 38.30 (C_2), 28.10 (C_8), 19.88 (C_3), 19.60 (C_9), 15.06 (C_9).

HRMS (ESI+) *m/z* calculated for $C_{18}H_{20}N_2O_5$ $[M+Na]^+$ 367.1264, found 367.1267.

X-ray quality crystals were obtained by slow eaporation of a solution of **12b** in $CHCl_3$.

Lactone **12c** was obtained following oxidation protocol A at 25 ºC, with (*S*,*S*)-Mn(^{DMM}pdp) (2 mol% x 2) and H_2O_2 (3 eq. x 2) and isolated in 20% yield as a white solid after column chromatography (SiO_2, hexane:EtOAc 7:3).

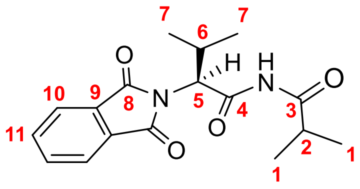

12c

^{1}H-NMR (400 MHz, $CDCl_3$) δ, ppm: 9.08 (br, 1H, NH), 7.91-7.89 (m, 2H, H_{10}), 7.79-7.77 (m, 2H, H_{11}), 4.54 (d, $^3J_{56}$ = 10.7 Hz, 1H, H_5), 3.16 (hept, $^3J_{12}$ = 6.8 Hz, 1H, H_2), 2.84 (m, 1H, H_6), 1.15 (d, $^3J_{12}$ = 6.8 Hz, 6H, H_1), 1.13 (d, $^3J_{67}$ = 6.6 Hz, 3H, H_7), 0.88 (d, $^3J_{67}$ = 6.6 Hz, 3H, H_7).

^{13}C-NMR (101 MHz, $CDCl_3$) δ, ppm: 178.40 (C_3), 168.36 (C_8), 167.22 (C_4), 134.87 (C_{11}), 131.31 (C_9), 124.11 (C_{10}), 63.30 (C_5), 35.76 (C_2), 27.61 (C_6), 20.03 (C_1), 19.34 (C_1), 18.86 (C_7), 18.61 (C_7).

HRMS (ESI+) *m/z* calculated for $C_{17}H_{20}N_2O_4$ $[M+Na]^+$ 339.1315, found 339.1331.

Oxidation of 13

Lactone **13a** was obtained following oxidation protocol B with (*R*,*R*)-Mn(^{TIPS}pdp) and isolated in 10% yield as a white solid after column chromatography (SiO_2, DCM:MeCN 97:3).

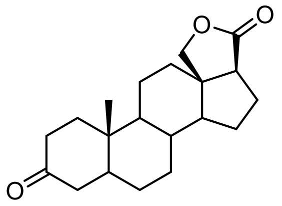

13a

^{1}H-NMR (400 MHz, $CDCl_3$) δ, ppm: 4.09 (d, *J* = 9.6 Hz, 1H), 3.98 (d, *J* = 9.6 Hz, 1H), 2.66 (dd, *J* = 15.3, 13.3 Hz, 1H), 2.51 (dd, *J* = 11.9, 3.4 Hz, 1H), 2.33 (td, *J* = 14.6, 5.4 Hz, 1H), 2.25–1.97 (m, 6H), 1.95–1.80 (m, 2H), 1.75 (dq, *J* = 13.9, 3.6 Hz, 1H), 1.66–1.52 (m, 2H), 1.44 (dd, *J* = 19.7, 9.3 Hz, 4H), 1.34–1.19 (m, 5H), 0.98 (s, 3H).

ESI-MS *m/z* calculated for $C_{20}H_{28}O_3$ $[M+K]^+$ 355.2, found 355.3.

The spectroscopic data matched those reported in the literature.[16]

Oxidation of 14

Lactone **14a** was obtained following oxidation protocol B with (*R*,*R*)-Mn(^{TIPS}pdp) and isolated in 12% yield as a white solid after column chromatography (SiO_2, DCM:MeCN 97:3).

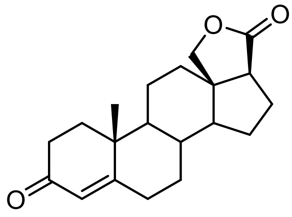

14a

^{1}H-NMR (400 MHz, $CDCl_3$) δ, ppm: 5.75 (s, 1H), 4.12 (d, *J* = 9.6 Hz, 1H), 4.01 (dd, *J* = 9.6 Hz, *J'* = 1.6 Hz, 1H), 2.52-2.26 (m, 5H), 2.17-1.64 (m, 9H), 1.45-1.24 (m, 4H), 1.20-1.09 (m, 4H), 1.05-0.99 (m, 1H).

^{13}C-NMR (101 MHz, $CDCl_3$) δ, ppm: 199.24, 180.49, 169.80, 124.37, 72.13, 54.77, 52.68, 51.24, 48.64, 38.56, 36.49, 35.82, 34.46, 33.97, 32.47, 32.09, 29.87, 26.67, 21.77, 17.53.

HRMS (ESI+) *m/z* calculated for $C_{20}H_{26}O_3$ $[M+Na]^+$ 337.1774, found 337.1776.

X-ray quality crystals were obtained by slow eaporation of a solution of **14a** in $CHCl_3$.

Oxidation of 15

Lactone **15a** was obtained following oxidation protocol B with (*S*,*S*)-Mn(^{TIPS}pdp) and isolated in 52% yield as a white solid after column chromatography (SiO_2, hexane:EtOAc 8:2).

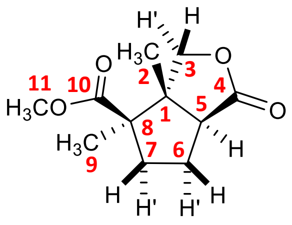

15a

^{1}H-NMR (400 MHz, $CDCl_3$) δ, ppm: 4.26 (d, $^2J_{3'3}$ = 8.3 Hz, 1H, $H_{3'}$), 3.99 (d, $^2J_{33'}$ = 8.3 Hz, 1H, H_3), 3.68 (s, 3H, H_{11}), 3.16 (dd, $^3J_{56}$ = 11.9 Hz, $^3J_{56'}$ = 7.2 Hz, 1H, H_5), 2.96 (ddd, $^3J_{76}$ = 13.9 Hz, $^3J_{76'}$ = 10.7 Hz, $^3J_{77'}$ = 2.8 Hz, 1H, H_7), 1.93-1.86 (m, 1H, $H_{7'}$), 1.83-1.64 (m, 2H, H_6 + $H_{6'}$), 1.35 (s, 3H, H_9), 1.08 (s, 3H, H_2).

^{13}C-NMR (101 MHz, $CDCl_3$) δ, ppm: 175.55 (C_{10}), 172.63 (C_4), 74.52 (C_3), 53.88 (C_1), 52.21 (C_{11}), 51.88 (C_5), 50.77 (C_8), 38.67 (C_7), 22.64 (C_9), 17.40 (C_2), 17.34 (C_6).

HRMS (ESI+) *m/z* calculated for $C_{11}H_{16}O_4$ $[M+Na]^+$ 235.0941, found 235.0946.

X-ray quality crystals were obtained by slow eaporation of a solution of **15a** in EtOAc.

Oxidation of 16

Lactone **16a** was obtained following oxidation protocol B with (*S*,*S*)-Mn(^{TMS}pdp) and isolated in 28% yield as a white solid after column chromatography (SiO_2, hexane:EtOAc 10:2).

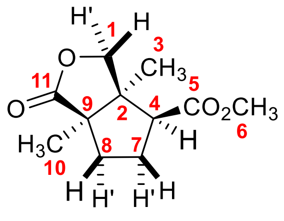

16a

^{1}H-NMR (400 MHz, $CDCl_3$) δ, ppm: 4.24 (d, $^2J_{11'}$ = 10.2 Hz, 1H, H_1), 3.91 (d, $^2J_{1'1}$ = 10.2 Hz, 1H, $H_{1'}$), 3.71 (s, 3H, H_6), 2.73 (dd, , $^3J_{47}$ = 11.6 Hz, $^3J_{47'}$ = 7.5 Hz, 1H, H_4), 2.27 (ddd, $^3J_{87}$ = 13.7 Hz, $^3J_{87}$ = 8.3 Hz, $^3J_{87'}$ = 2.6 Hz, 1H, H_8), 2.06-1.98 (m, 1H, $H_{7'}$), 1.94-1.83 (m, 1H, H_7), 1.68-1.60 (m, 1H, $H_{8'}$), 1.25 (s, 3H, H_3), 1.19 (s, 3H, H_{10}).

^{13}C-NMR (101 MHz, $CDCl_3$) δ, ppm: 182.04 (C_{11}), 173.13 (C_5), 74.26 (C_1), 54.49 (C_4), 52.53 (C_9), 52.01 (C_6), 51.28 (C_2), 37.79 (C_8), 26.99 (C_7), 21.51 (C_3), 18.93 (C_{10}).

HRMS (ESI+) *m/z* calculated for $C_{11}H_{16}O_4$ $[M+Na]^+$ 235.0941, found 235.0944.

X-ray quality crystals were obtained by slow eaporation of a solution of **16a** in EtOAc.

Lactone **16b** was obtained following oxidation protocol B with (*R*,*R*)-Mn(^{DMM}pdp) and isolated in 61% yield as a white solid after column chromatography (SiO_2, hexane:EtOAc 10:1).

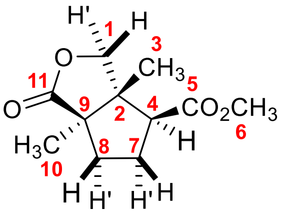

16b

^{1}H-NMR (400 MHz, $CDCl_3$) δ, ppm: 4.48 (dd, $^{2}J_{1'1}$ = 8.6 Hz, $^{3}J_{1'10}$ = 1.1 Hz, 1H, $H_{1'}$), 4.01 (d, $^{2}J_{11'}$ = 8.6 Hz, 1H, H_1), 3.70 (s, 3H, H_6), 3.02 (dd, $^{3}J_{47'}$ = 9.7 Hz, $^{3}J_{47}$ = 7.6 Hz, 1H, H_4), 2.69-2.60 (m, 1H, H_7), 2.46-2.36 (m, 1H, $H_{7'}$), 1.98-1.90 (m, 1H, H_8), 1.48-1.42 (m, 1H, $H_{8'}$), 1.36 (d, $^{3}J_{101'}$ = 1.1 Hz, 3H, H_{10}), 1.15 (s, 3H, H_3).

^{13}C-NMR (101 MHz, $CDCl_3$) δ, ppm: 175.69 (C_{11}), 173.09 (C_5), 75.42 (C_1), 57.76 (C_9), 53.65 (C_2), 52.17 (C_6), 43.31 (C_4), 29.23 (C_7), 25.45 (C_8), 20.16 (C_{10}), 19.93 (C_3).

HRMS (ESI+) *m/z* calculated for $C_{11}H_{16}O_4$ $[M+Na]^+$ 235.0941, found 235.0942.

X-ray quality crystals were obtained by slow eaporation of a solution of **16b** in EtOAc.

Lactone **16c** was obtained following oxidation protocol B with (*S*,*S*)-Mn(^{TMS}pdp) and isolated in 19% yield as a white solid after column chromatography (SiO_2, hexane:EtOAc 10:2).

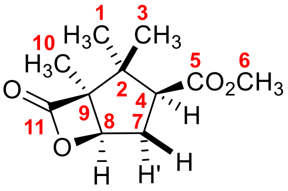

16c

^{1}H-NMR (400 MHz, $CDCl_3$) δ, ppm: 4.67 (d, $^{3}J_{87'}$ = 4.8 Hz, 1H, H_8), 3.67 (s, 3H, H_6), 2.88 (dd, $^{3}J_{47'}$ = 9.2 Hz, $^{3}J_{47}$ = 1.5 Hz, 1H, H_4), 2.49 (dd, $^{2}J_{77'}$ = 15.9 Hz, $^{3}J_{74}$ = 1.5 Hz, 1H, H_7), 2.23 (ddd, $^{2}J_{7'7}$ = 15.9 Hz, $^{3}J_{7'4}$ = 9.2 Hz, $^{3}J_{7'8}$ = 4.8 Hz, 1H, $H_{7'}$), 1.36 (s, 3H, H_{10}), 1.22 (s, 3H, H_3), 1.00 (s, 3H, H_1).

^{13}C-NMR (101 MHz, $CDCl_3$) δ, ppm: 173.64, 173.62, 84.06 (C_8), 69.37 (C_9), 55.58 (C_4), 51.86 (C_6), 45.24 (C_2), 31.88 (C_7), 26.91 (C_1), 19.55 (C_3), 12.97 (C_{10}).

HRMS (ESI+) *m/z* calculated for $C_{11}H_{16}O_4$ $[M+Na]^+$ 235.0941, found 235.0943.

X-ray quality crystals were obtained by slow eaporation of a solution of **16c** in EtOAc.

Oxidation of 17

Lactone **17a** was obtained following oxidation protocol B with (*R*,*R*)-Mn(pdp) and isolated in 28% yield (+54% yield **17b**) as a white solid after column chromatography (SiO_2, hexane:EtOAc 9:1).

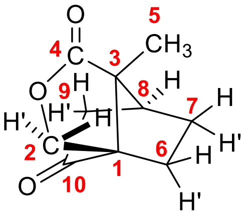

17a

^{1}H-NMR (400 MHz, $CDCl_3$) δ, ppm: 4.69 (d, $^{2}J_{2'2}$ = 8.8 Hz, 1H, $H_{2'}$), 4.25 (d, $^{2}J_{22'}$ = 8.8 Hz, 1H, H_2), 2.61 (t, $^{3}J_{8}$ = 4.2 Hz, 1H, H_8), 2.57-2.48 (m, 1H, H_7), 2.40 (ddd, $^{2}J_{99'}$ = 18.4 Hz, $^{3}J_{98}$ = 3.6 Hz, $^{4}J_{97}$ = 0.9 Hz, 1H, H_9), 2.21 (d, $^{2}J_{99'}$ = 18.4 Hz, 1H, $H_{9'}$), 1.91-1.80 (m, 2H, H_6 + $H_{7'}$), 1.45-1.41 (m, 1H, $H_{6'}$), 1.39 (s, 3H, H_5).

^{13}C-NMR (101 MHz, $CDCl_3$) δ, ppm: 215.45 (C_{10}), 175.49 (C_4), 67.61 (C_2), 65.56 (C_3), 58.95 (C_1), 44.74 (C_9), 36.81 (C_8), 33.99 (C_7), 19.10 (C_6), 13.92 (C_5).

HRMS (ESI+) *m/z* calculated for $C_{10}H_{12}O_3$ $[M+Na]^+$ 203.0679, found 219.0681.

Lactone **17b** was obtained following oxidation protocol B with (*S*,*S*)-Mn(^{TIPS}pdp) and isolated in 90% yield as a white solid after column chromatography (SiO_2, hexane:EtOAc 9:1).

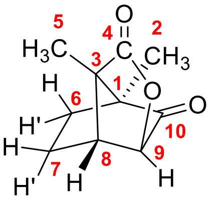

17b

^{1}H-NMR (400 MHz, $CDCl_3$) δ, ppm: 4.52 (d, $^{3}J_{89}$ = 2.7 Hz, 1H, H_9), 2.94 (dd, , $^{3}J_{87}$ = 5.0 Hz, $^{3}J_{89}$ = 2.7 Hz, 1H, H_8), 2.02-1.94 (m, 1H, H_6), 1.90-1.74 (m, 2H, $H_{6'}$ + H_7), 1.70-1.63 (m, 1H, $H_{7'}$), 1.24 (s, 3H, H_5), 1.09 (s, 3H, H_2).

^{13}C-NMR (101 MHz, $CDCl_3$) δ, ppm: 207.56 (C_{10}), 176.54 (C_4), 83.08 (C_9), 56.73 (C_3), 56.55 (C_8), 55.37 (C_1), 34.79 (C_6), 18.88 (C_7), 9.59 (C_2), 8.99 (C_5).

HRMS (ESI+) *m/z* calculated for $C_{10}H_{12}O_3$ $[M+Na]^+$ 203.0679, found 219.0684.

X-ray quality crystals were obtained by slow eaporation of a solution of **17b** in EtOAc.

Oxidation of 18

Lactone **18a** was obtained following oxidation protocol B at 40 ºC with (*R*,*R*)-Mn(^{TIPS}pdp) (5 mol% x 3) and H_2O_2 (2 eq. x 3) and isolated in 57% yield as a white solid after column chromatography (SiO_2, hexane:EtOAc 8:2). It has also been isolated in 62% yield under the same conditions using (*R*,*R*)-Mn(pdp).

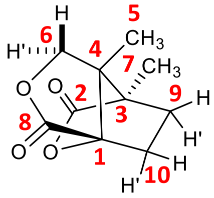

18a

^{1}H-NMR (400 MHz, $CDCl_3$) δ, ppm: 4.33 (d, $^2J_{6'6}$ = 9.3 Hz, 1H, $H_{6'}$), 3.99 (d, $^2J_{66'}$ = 9.4 Hz, 1H, H_6), 2.40-2.34 (m, 1H, H_{10}), 2.15-2.04 (m, 3H, $H_{10'}$, H_9 + $H_{9'}$), 1.29 (s, 3H, H_5), 1.24 (s, 3H, H_7).

^{13}C-NMR (101 MHz, $CDCl_3$) δ, ppm: 175.94 (C_2), 166.27 (C_8), 93.87 (C_1), 73.72 (C_6), 58.68 (C_3), 50.47 (C_4), 36.19 (C_{10}), 23.49 (C_9), 14.23 (C_5), 10.63 (C_7).

HRMS (ESI+) *m/z* calculated for $C_{11}H_{12}O_4$ $[M+Na]^+$ 219.0628, found 219.0628.

X-ray quality crystals were obtained by slow eaporation of a solution of **18a** in EtOAc.

Lactone **18b** was obtained following oxidation protocol B at 40 ºC with (*S*,*S*)-Mn(^{TIPS}pdp) (5 mol% x 3) and H_2O_2 (2 eq. x 3) and isolated in 60% yield (**18a** + **18b** dr: 1:8.7) as a white solid after column chromatography (SiO_2, hexane:EtOAc 8:2).

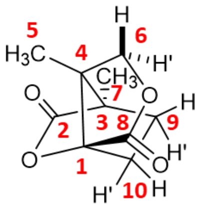

18b

^{1}H-NMR (400 MHz, $CDCl_3$) δ, ppm: 4.34 (d, $^2J_{6'6}$ = 8.9 Hz, 1H, $H_{6'}$), 4.09 (d, $^2J_{66'}$ = 8.9 Hz, 1H, H_6), 2.42-2.35 (m, 1H, H_{10}), 2.30-2.22 (m, 1H, $H_{10'}$), 2.09-2.01 (m, 1H, H_9), 1.91-1.84 (m, 1H, $H_{9'}$), 1.35 (s, 3H, H_7), 1.24 (s, 3H, H_5).

^{13}C-NMR (101 MHz, $CDCl_3$) δ, ppm: 178.90 (C_2), 167.69 (C_8), 86.23 (C_1), 72.70 (C_6), 58.91, 58.78, 32.52 (C_{10}), 27.51 (C_9), 15.27 (C_5), 11.68 (C_7).

HRMS (ESI+) *m/z* calculated for $C_{11}H_{12}O_4$ $[M+Na]^+$ 219.0628, found 219.0628.

X-ray quality crystals were obtained by slow eaporation of a solution of **18b** in EtOAc.

Oxidation of 19

Lactone **19a** was obtained following oxidation protocol B with (*R*,*R*)-Mn(pdp) and isolated in 31% yield as a white solid after column chromatography (SiO_2, hexane:EtOAc 8:2).

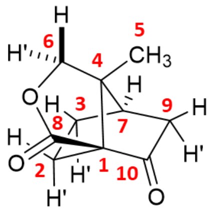

19a

^{1}H-NMR (400 MHz, $CDCl_3$) δ, ppm: 4.42 (dq, $^2J_{6'6}$ = 9.2 Hz, $^4J_{6'5}$ = 1.1 Hz, 1H, $H_{6'}$), 3.93 (d, $^2J_{66'}$ = 9.2 Hz, 1H, H_6), 2.89-2.79 (m, 1H, H_9), 2.57-2.54 (m, 1H, H_7), 2.51-2.42 (m, 1H, H_2), 2.27-2.12 (m, 3H, $H_{9'}$, $H_{2'}$ and H_3), 1.89-1.79 (m, 1H, $H_{3'}$), 1.35 (d, $^4J_{56'}$ = 1.1 Hz, 3H, H_5).

^{13}C-NMR (101 MHz, $CDCl_3$) δ, ppm: 200.30 (C_{10}), 169.02 (C_8), 72.19 (C_6), 63.30 (C_1), 57.87 (C_4), 49.14 (C_9), 41.25 (C_7), 29.98 (C_2), 27.91 (C_3), 17.53 (C_5).

HRMS (ESI+) *m/z* calculated for $C_{11}H_{12}O_3$ $[M+Na]^+$ 203.0679, found 203.0681.

X-ray quality crystals were obtained by slow eaporation of a solution of **19a** in EtOAc.

Lactone **19b** was obtained following oxidation protocol B with (*S*,*S*)-Mn(^{TIPS}pdp) and isolated in 46% yield as a white solid after column chromatography (SiO_2, hexane:EtOAc 8:2). It has also been isolated in 80% yield under the same conditions using (*S*,*S*)-Mn(pdp).

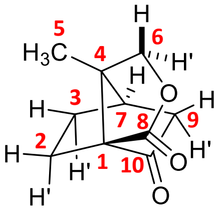

19b

^{1}H-NMR (400 MHz, $CDCl_3$) δ, ppm: 4.16 (dq, $^2J_{6'6}$ = 9.9 Hz, $^4J_{6'5}$ = 1.1 Hz, 1H, $H_{6'}$), 4.02 (d, $^2J_{6'6}$ = 9.9 Hz, 1H, H_6), 2.64 – 2.51 (m, 2H, H_3 and H_9), 2.49-2.47 (m, 1H, H_7), 2.26 (d, $^2J_{9'9}$= 18.3 Hz, 1H, $H_{9'}$), 2.03 – 1.93 (m, 1H, H_2), 1.90-1.82 (m, 1H, $H_{3'}$), 1.60 (ddd, $^2J_{22'}$ = 12.8, $^3J_{2'3'}$= 8.6, $^3J_{2'3}$ = 3.8 Hz, 1H, $H_{2'}$), 1.37 (d, $^4J_{56'}$ = 1.1 Hz, 3H, H_5).

^{13}C-NMR (101 MHz, $CDCl_3$) δ, ppm: 209.87 (C_{10}), 169.74 (C_8), 72.87 (C_6), 70.93 (C_1), 58.38 (C_4), 43.75 (C_9), 37.36 (C_7), 35.11 (C_3), 17.44 (C_2), 17.06 (C_5).

HRMS (ESI+) *m/z* calculated for $C_{11}H_{12}O_3$ $[M+Na]^+$ 203.0679, found 203.0681.

X-ray quality crystals were obtained by slow eaporation of a solution of **19b** in EtOAc.

8. Lactone derivatization

The synthesis of the iodo compounds **19a-I**, **19b-I** and **19b-I^{COOH}** was carried out following the general procedure reported in the literature with slight modifications.[17]

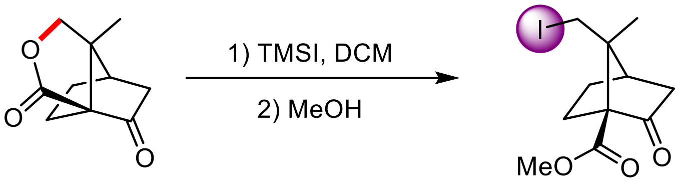

19a **19a-I**

To stirred solution of lactone **19a** (0.175 mmol, 1.0 eq.) under nitrogen atmosphere in anhydrous CH_2Cl_2 (5 mL), protected from light, was added iodotrimethylsilane (0.140 mL, 0.7 mmol, 4 eq.) dropwise. The resulting orange solution was left stirred at room temperature until the reaction was complete as judged by ^{1}H-NMR spectroscopy. Anhydrous methanol (0.07 mL, 10 eq.) was added and the reaction was monitored by ^{1}H-NMR spectroscopy until ester formation was complete. After that, $Na_2S_2O_3$ (10%, 5 mL) was added. The aqueous layer was extracted with CH_2Cl_2 (2 x 5 mL) and the combined organic layers were dried over $MgSO_4$ and evaporated under reduced pressure. The residual pale solid was purified by flash column chromatography (SiO_2, hexane:EtOAc 95:5) to leave the desired iodo ester compound **19a-I** as a pale yellow solid (0.034 g, 0.106 mmol, 60% yield).

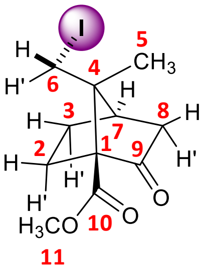

19a-I

^{1}H-NMR (400 MHz, $CDCl_3$) δ, ppm: 3.77 (s, 3H, H_{11}), 3.40 (dd, $^{2}J_{6'6}$ = 10.2 Hz, $^{4}J_{6'5}$ = 1.3 Hz, 1H, $H_{6'}$), 3.23 (d, $^{2}J_{66'}$ = 10.2 Hz, 1H, H_6), 2.54-2.48 (m, 1H, H_8), 2.40-2.31 (m, 2H, H_3 + H_7), 2.03 (d, $^{2}J_{8'8}$ = 18.5 Hz, 1H, $H_{8'}$), 1.89-1.82 (m, 2H, H_2 + $H_{3'}$), 1.51-1.45 (m, 1H, $H_{2'}$), 1.28 (s, 3H, H_5).

^{13}C-NMR (101 MHz, $CDCl_3$) δ, ppm: 209.62 (C_9), 169.58 (C_{10}), 67.13 (C_1), 52.34 (C_4), 52.17 (C_{11}), 44.21 (C_7), 43.53 (C_8), 26.46 (C_3), 25.99 (C_2), 19.48 (C_5), 13.69 (C_6).

HRMS (ESI+) *m/z* calculated for $C_{11}H_{15}IO_3$ $[M+Na]^+$ 344.9958, found 344.9961.

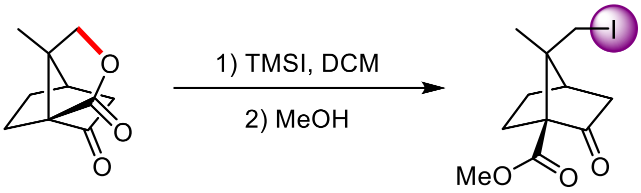

19b **19b-I**

To stirred solution of lactone **19b** (0.4 mmol, 1.0 eq.) under nitrogen atmosphere in anhydrous CH_2Cl_2 (5 mL), protected from light, was added iodotrimethylsilane (0.23 mL, 1.6 mmol, 4 eq.) dropwise. The resulting orange solution was left stirred at room temperature until the reaction was complete as judged by ^{1}H-NMR spectroscopy. Anhydrous methanol (0.16 mL, 10 eq.) was added and the reaction was monitored by ^{1}H-NMR spectroscopy until ester formation was complete. After that, $Na_2S_2O_3$ (10%, 5 mL) was added. The aqueous layer was extracted with CH_2Cl_2 (2 x 5 mL) and the combined organic layers were dried over $MgSO_4$ and evaporated under reduced pressure. The residual pale solid was purified by flash column chromatography (SiO_2, hexane:EtOAc 95:5) to leave the desired iodo ester compound **19b-I** as a pale yellow solid (0.090 g, 0.280 mmol, 70% yield).

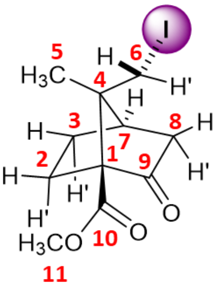

19b-I

^{1}H-NMR (400 MHz, $CDCl_3$) δ, ppm: 3.76 (s, 3H, H_{11}), 3.52 (d, $^{2}J_{66'}$ = 11.2 Hz, 1H, H_6), 3.18 (dd, $^{2}J_{6'6}$ = 11.2 Hz, $^{4}J_{6'5}$ = 1.3 Hz, 1H, $H_{6'}$), 2.55-2.41 (m, 3H, H_3, H_7 + H_8), 2.09-1.98 (m, 3H, H_2 + $H_{3'}$ + $H_{8'}$), 1.48-1.41 (m, 1H, $H_{2'}$), 1.22 (d, $^{4}J_{56'}$ = 1.3 Hz, 1H, H_5). ^{13}C-NMR (101 MHz, $CDCl_3$) δ, ppm: 209.56 (C_9), 169.61 (C_{10}), 66.93 (C_1), 52.47 (C_4), 52.39 (C_{11}), 45.28 (C_7), 43.14 (C_8), 29.32 (C_3), 25.37 (C_2), 17.33 (C_5), 14.93 (C_6). HRMS (ESI+) *m/z* calculated for $C_{11}H_{15}IO_3$ $[M+Na]^+$ 344.9958, found 344.9958. X-ray quality crystals were obtained by slow eaporation of a solution of **19b-I** in $CHCl_3$.

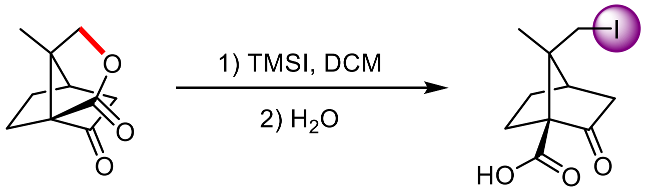

19b **19b-I^{COOH}**

To stirred solution of lactone **19b** (0.8 mmol, 1.0 eq.) under nitrogen atmosphere in anhydrous CH_2Cl_2 (1 mL), protected from light, was added iodotrimethylsilane (0.46 mL, 3.2 mmol, 4 eq.) dropwise. The resulting orange solution was left stirred at room temperature until the reaction was complete as judged by ^{1}H-NMR spectroscopy. After that, $Na_2S_2O_3$ (10%, 10 mL) was added, and the mixture was stirred for 4 hours. The aqueous layer was extracted with CH_2Cl_2 (2 x 10 mL) and the combined organic layers were dried over $MgSO_4$ and evaporated under reduced pressure. The residual pale solid was purified by flash column chromatography (SiO_2, DCM:MeOH 99:1) to leave the desired iodo acid compound **19b-I^{COOH}** as a white solid (0.130 g, 0.422 mmol, 53% yield).

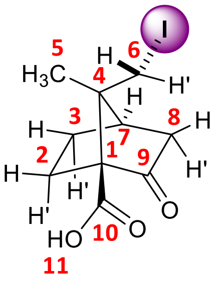

19b-I^{COOH}

^{1}H-NMR (400 MHz, $CDCl_3$) δ, ppm: 8.55 (br, 1H, H_{11}), 3.48 (d, $^2J_{66'}$ = 11.1 Hz, 1H, H_6), 3.12 (dd, $^2J_{6'6}$ = 11.1 Hz, $^4J_{6'5}$ = 1.3 Hz, 1H, $H_{6'}$), 2.59-2.45 (m, 3H, H_3, H_7 and H_8), 2.13-2.02 (m, 3H, H_2, $H_{3'}$ and $H_{8'}$), 1.55-1.44 (m, 1H, $H_{2'}$), 1.31 (d, $^4J_{56'}$ = 1.3 Hz, 1H, H_5).

^{13}C-NMR (101 MHz, $CDCl_3$) δ, ppm: 210.69 (C_9), 174.08 (C_{10}), 65.96 (C_1), 52.87 (C_4), 45.16 (C_7), 42.99 (C_8), 29.87 (C_3), 25.56 (C_2), 17.46 (C_5), 13.74 (C_6).

HRMS (ESI+) *m/z* calculated for $C_{11}H_{13}IO_3$ $[M+Na]^+$ 330.9802, found 330.9792.

9. Characterization of the compounds

9.1. Solid state structures by X-ray diffraction analisis

9.1.1. Solid state structure of **12b**.

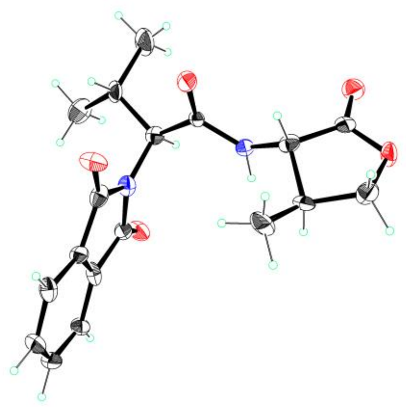

Table S15. Crystal data for **12b**.

**Chemical formula**	$C_{18}H_{20}N_2O_5$	
**Formula weight**	344.36 g/mol	
**Temperature**	100(2) K	
**Wavelength**	0.71076 Å	
**Crystal size**	0.010 x 0.030 x 0.230 mm	
**Crystal habit**	colorless needle	
**Crystal system**	orthorhombic	
**Space group**	P 21 21 21	
**Unit cell dimensions**	a = 5.078(6) Å	α = 90°
	b = 10.566(15) Å	β = 90°
	c = 31.40(3) Å	γ = 90°
**Volume**	1685.(4) $Å^3$	
**Z**	4	
**Density (calculated)**	1.358 g/cm^3	
**Absorption coefficient**	0.100 mm^{-1}	
**F(000)**	728	
**Diffractometer**	Bruker D8 QUEST ECO three-circle diffractometer	
**Radiation source**	Ceramic x-ray tube (Mo Kα, λ = 0.71076 Å)	
**Theta range for data collection**	3.23 to 23.34°	
**Index ranges**	-5<=h<=5, -11<=k<=11, -33<=l<=34	
**Reflections collected**	7899	
**Independent reflections**	2431 [R(int) = 0.0754]	
**Coverage of independent reflections**	99.4%	
**Absorption correction**	Multi-Scan	

**Max. and min. transmission**	0.7449 and 0.5669
**Structure solution technique**	direct methods
**Structure solution program**	SHELXT 2014/5 (Sheldrick, 2014)
**Refinement method**	Full-matrix least-squares on F2
**Refinement program**	SHELXL-2017/1 (Sheldrick, 2017)
**Function minimized**	Σ w(Fo2 - Fc2)2
**Data / restraints / parameters**	2431 / 0 / 233
**Goodness-of-fit on F^2**	1.197
**Final R indices**	2043 data; I>2σ(I) R1 = 0.0728, wR2 = 0.1635
	all data R1 = 0.0883, wR2 = 0.1693
**Weighting scheme**	$w=1/[\sigma^2(F_o^2)+(0.0383P)^2+3.4666P]$<br>where $P=(F_o^2+2F_c^2)/3$
**Absolute structure parameter**	-0.4(10)
**Largest diff. peak and hole**	0.297 and -0.302 eÅ^{-3}
**R.M.S. deviation from mean**	0.073 eÅ^{-3}

9.1.2. Solid state structure of **14a**.

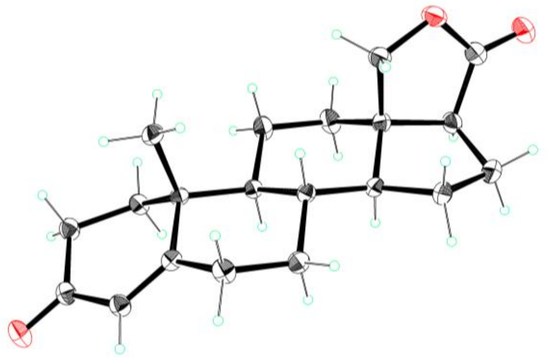

Table S16. Crystal data for **14a**.

**Chemical formula**	$C_{20}H_{26}O_3$	
**Formula weight**	314.41 g/mol	
**Temperature**	100(2) K	
**Wavelength**	0.71076 Å	
**Crystal size**	0.150 x 0.200 x 0.280 mm	
**Crystal habit**	colorless block	
**Crystal system**	orthorhombic	
**Space group**	P 21 21 21	
**Unit cell dimensions**	a = 7.249(5) Å	α = 90°
	b = 12.873(8) Å	β = 90°
	c = 17.456(10) Å	γ = 90°
**Volume**	1628.9(17) Å^3	
**Z**	4	
**Density (calculated)**	1.282 g/cm^3	
**Absorption coefficient**	0.084 mm^{-1}	
**F(000)**	680	
**Diffractometer**	Bruker D8 QUEST ECO three-circle diffractometer	
**Radiation source**	Ceramic x-ray tube (Mo Kα, λ = 0.71076 Å)	
**Theta range for data collection**	3.04 to 30.60°	
**Index ranges**	-10<=h<=10, -18<=k<=18, -24<=l<=24	
**Reflections collected**	90283	
**Independent reflections**	4997 [R(int) = 0.0491]	
**Coverage of independent reflections**	99.5%	
**Absorption correction**	Multi-Scan	
**Max. and min. transmission**	0.7461 and 0.6920	
**Structure solution technique**	direct methods	

**Structure solution program**	SHELXT 2014/5 (Sheldrick, 2014)
**Refinement method**	Full-matrix least-squares on F^2
**Refinement program**	SHELXL-2017/1 (Sheldrick, 2017)
**Function minimized**	$\Sigma\ w(F_o^2 - F_c^2)^2$
**Data / restraints / parameters**	4997 / 0 / 209
**Goodness-of-fit on F^2**	1.108
**Final R indices**	4713 data; I>2σ(I) R1 = 0.0331, wR2 = 0.0893
	all data R1 = 0.0370, wR2 = 0.0931
**Weighting scheme**	$w=1/[\sigma^2(F_o^2)+(0.0554P)^2+0.2093P]$ where $P=(F_o^2+2F_c^2)/3$
**Absolute structure parameter**	0.1(2)
**Largest diff. peak and hole**	0.291 and -0.179 eÅ^{-3}
**R.M.S. deviation from mean**	0.040 eÅ^{-3}

9.1.3. Solid state structure **15a**.

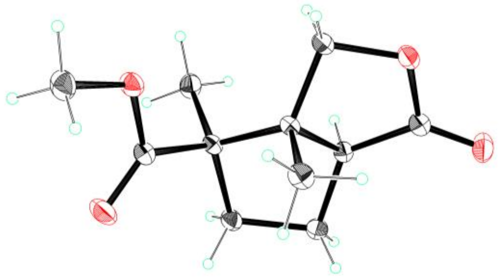

Table S17. Crystal data for **15a**.

**Chemical formula**	$C_{11}H_{16}O_4$	
**Formula weight**	212.24 g/mol	
**Temperature**	100(2) K	
**Wavelength**	0.71076 Å	
**Crystal size**	0.100 x 0.160 x 0.300 mm	
**Crystal habit**	colorless prism	
**Crystal system**	monoclinic	
**Space group**	P 1 21 1	
**Unit cell dimensions**	a = 7.183(3) Å	α = 90°
	b = 7.623(3) Å	β = 100.618(18)°
	c = 9.938(5) Å	γ = 90°
**Volume**	534.8(4) Å^3	
**Z**	2	
**Density (calculated)**	1.318 g/cm^3	
**Absorption coefficient**	0.100 mm^{-1}	
**F(000)**	228	
**Diffractometer**	Bruker D8 QUEST ECO three-circle diffractometer	
**Radiation source**	Ceramic x-ray tube (Mo Kα, λ = 0.71076 Å)	
**Theta range for data collection**	3.23 to 33.14°	
**Index ranges**	-11<=h<=11, -11<=k<=11, -15<=l<=15	
**Reflections collected**	29751	
**Independent reflections**	4047 [R(int) = 0.0368]	
**Coverage of independent reflections**	99.6%	
**Absorption correction**	Multi-Scan	
**Max. and min. transmission**	0.7465 and 0.7117	
**Structure solution technique**	direct methods	
**Structure solution program**	SHELXT 2014/5 (Sheldrick, 2014)	

**Refinement method**	Full-matrix least-squares on F^2
**Refinement program**	SHELXL-2017/1 (Sheldrick, 2017)
**Function minimized**	$\Sigma\, w(F_o^2 - F_c^2)^2$
**Data / restraints / parameters**	4047 / 1 / 200
**Goodness-of-fit on F^2**	1.103
**Final R indices**	3632 data; $I>2\sigma(I)$ R1 = 0.0342, wR2 = 0.0780
	all data R1 = 0.0451, wR2 = 0.0865
**Weighting scheme**	$w=1/[\sigma^2(F_o^2)+(0.0395P)^2+0.0912P]$<br>where $P=(F_o^2+2F_c^2)/3$
**Absolute structure parameter**	0.1(2)
**Largest diff. peak and hole**	0.301 and -0.231 eÅ^{-3}
**R.M.S. deviation from mean**	0.045 eÅ^{-3}

9.1.4. Solid state structure of **16a**.

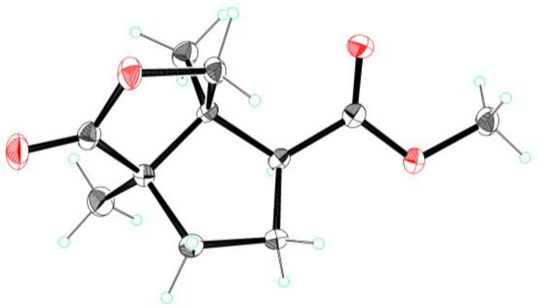

Table S18. Crystal data for **16a**.

**Chemical formula**	$C_{11}H_{16}O_4$	
**Formula weight**	212.24 g/mol	
**Temperature**	100(2) K	
**Wavelength**	0.71076 Å	
**Crystal size**	0.020 x 0.100 x 0.130 mm	
**Crystal habit**	colorless plate	
**Crystal system**	monoclinic	
**Space group**	P 1 21 1	
**Unit cell dimensions**	a = 6.894(6) Å	α = 90°
	b = 7.409(7) Å	β = 106.89(5)°
	c = 10.829(11) Å	γ = 90°
**Volume**	529.3(9) $Å^3$	
**Z**	2	
**Density (calculated)**	1.332 g/cm^3	
**Absorption coefficient**	0.101 mm^{-1}	
**F(000)**	228	
**Diffractometer**	Bruker D8 QUEST ECO three-circle diffractometer	
**Radiation source**	Ceramic x-ray tube (Mo Kα, λ = 0.71076 Å)	
**Theta range for data collection**	3.09 to 29.66°	
**Index ranges**	-9<=h<=9, -10<=k<=10, -15<=l<=15	
**Reflections collected**	12389	
**Independent reflections**	2977 [R(int) = 0.0558]	
**Coverage of independent reflections**	99.2%	
**Absorption correction**	Multi-Scan	
**Max. and min. transmission**	0.7459 and 0.6589	
**Structure solution technique**	direct methods	
**Structure solution program**	SHELXT 2014/5 (Sheldrick, 2014)	
**Refinement method**	Full-matrix least-squares on F^2	

**Refinement program**	SHELXL-2017/1 (Sheldrick, 2017)
**Function minimized**	Σ w(F_o^2 - F_c^2)^2
**Data / restraints / parameters**	2977 / 1 / 139
**Goodness-of-fit on F^2**	1.026
**Final R indices**	2480 data; I>2σ(I) R1 = 0.0417, wR2 = 0.0871
	all data R1 = 0.0579, wR2 = 0.0934
**Weighting scheme**	w=1/[σ^2(F_o^2)+(0.0428P)^2+0.0809P]<br>where P=(F_o^2+2F_c^2)/3
**Absolute structure parameter**	0.0(6)
**Largest diff. peak and hole**	0.318 and -0.215 eÅ^{-3}
**R.M.S. deviation from mean**	0.044 eÅ^{-3}

9.1.5. Solid state structure of **16b**.

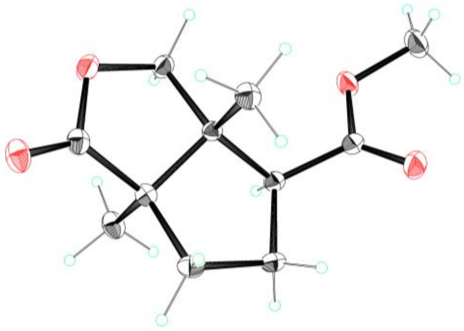

Table S19. Crystal data for **16b**.

**Chemical formula**	$C_{11}H_{16}O_4$	
**Formula weight**	212.24 g/mol	
**Temperature**	100(2) K	
**Wavelength**	0.71073 Å	
**Crystal size**	0.030 x 0.060 x 0.300 mm	
**Crystal habit**	colorless needle	
**Crystal system**	orthorhombic	
**Space group**	P 21 21 21	
**Unit cell dimensions**	a = 6.7478(4) Å	α = 90°
	b = 11.3960(6) Å	β = 90°
	c = 14.2371(7) Å	γ = 90°
**Volume**	1094.80(10) $Å^3$	
**Z**	4	
**Density (calculated)**	1.288 g/cm^3	
**Absorption coefficient**	0.097 mm^{-1}	
**F(000)**	456	
**Diffractometer**	Bruker D8 QUEST ECO three-circle diffractometer	
**Radiation source**	Ceramic x-ray tube (Mo Kα, λ = 0.71073 Å)	
**Theta range for data collection**	3.34 to 30.45°	
**Index ranges**	-9<=h<=9, -16<=k<=16, -20<=l<=20	
**Reflections collected**	62617	
**Independent reflections**	3330 [R(int) = 0.0503]	
**Coverage of independent reflections**	99.8%	
**Absorption correction**	Multi-Scan	
**Max. and min. transmission**	0.9970 and 0.9710	
**Structure solution technique**	direct methods	
**Structure solution program**	SHELXT 2014/5 (Sheldrick, 2014)	

**Refinement method**	Full-matrix least-squares on F^2
**Refinement program**	SHELXL-2017/1 (Sheldrick, 2017)
**Function minimized**	$\Sigma w(F_o^2 - F_c^2)^2$
**Data / restraints / parameters**	3330 / 0 / 139
**Goodness-of-fit on F^2**	1.091
**Final R indices**	3114 data; $I>2\sigma(I)$ R1 = 0.0345, wR2 = 0.0845
	all data R1 = 0.0385, wR2 = 0.0864
**Weighting scheme**	$w=1/[\sigma^2(F_o^2)+(0.0498P)^2+0.1417P]$<br>where $P=(F_o^2+2F_c^2)/3$
**Bijvoet Pairs**	1410
**Coverage (Bijvoet pairs)**	100
**Flack x**	0.1(3)
**Parsons z**	0.1(2)
**Hooft y**	0.1(2)
**P2(true)**	1.0
**P3(true)**	0.818
**P3(rac-twin)**	0.182
**P3(false)**	0.3E-03
**G**	0.8012
**G (su)**	0.4479
**Largest diff. peak and hole**	0.327 and -0.143 eÅ^{-3}
**R.M.S. deviation from mean**	0.040 eÅ^{-3}

9.1.6. Solid state structure of **16c**.

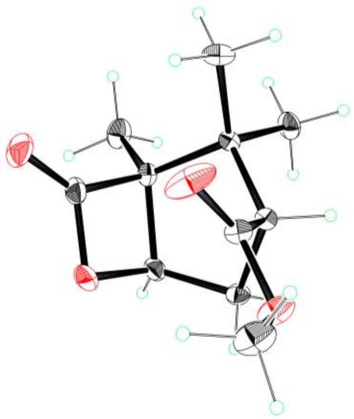

Table S20. Crystal data for **16c**.

**Chemical formula**	$C_{11}H_{16}O_4$	
**Formula weight**	212.24 g/mol	
**Temperature**	100(2) K	
**Wavelength**	0.71076 Å	
**Crystal size**	0.080 x 0.160 x 0.300 mm	
**Crystal habit**	colorless block	
**Crystal system**	orthorhombic	
**Space group**	P 21 21 21	
**Unit cell dimensions**	a = 7.938(10) Å	α = 90°
	b = 11.421(14) Å	β = 90°
	c = 11.949(16) Å	γ = 90°
**Volume**	1083.(2) $Å^3$	
**Z**	4	
**Density (calculated)**	1.301 g/cm^3	
**Absorption coefficient**	0.098 mm^{-1}	
**F(000)**	456	
**Diffractometer**	Bruker D8 QUEST ECO three-circle diffractometer	
**Radiation source**	Ceramic x-ray tube (Mo Kα, λ = 0.71076 Å)	
**Theta range for data collection**	2.47 to 33.74°	
**Index ranges**	-12<=h<=12, -17<=k<=17, -18<=l<=18	
**Reflections collected**	91249	
**Independent reflections**	4329 [R(int) = 0.0523]	
**Coverage of independent reflections**	99.8%	
**Absorption correction**	Multi-Scan	
**Max. and min. transmission**	0.9920 and 0.9710	
**Structure solution technique**	direct methods	

**Structure solution program**	SHELXT 2014/5 (Sheldrick, 2014)
**Refinement method**	Full-matrix least-squares on F^2
**Refinement program**	SHELXL-2017/1 (Sheldrick, 2017)
**Function minimized**	$\Sigma\, w(F_o^2 - F_c^2)^2$
**Data / restraints / parameters**	4329 / 0 / 140
**Goodness-of-fit on F^2**	1.078
**Final R indices**	3945 data; $I>2\sigma(I)$ R1 = 0.0367, wR2 = 0.0903
	all data R1 = 0.0428, wR2 = 0.0941
**Weighting scheme**	$w=1/[\sigma^2(F_o^2)+(0.0527P)^2+0.1502P]$ where $P=(F_o^2+2F_c^2)/3$
**Flack x**	-0.03(19)
**Parsons z**	0.02(16)
**Bijvoet Pairs**	1870
**Coverage (Bijvoet Pairs)**	100
**P2(true)**	1.00
**P3(true)**	0.993
**P3(rac-twin)**	0.007
**P3(false)**	0.9E-08
**G**	1.0777
**G (su)**	0.3411
**Hooft y**	-0.04(17)
**Largest diff. peak and hole**	0.313 and -0.251 eÅ^{-3}
**R.M.S. deviation from mean**	0.060 eÅ^{-3}

9.1.7. Solid state structure of **17b**.

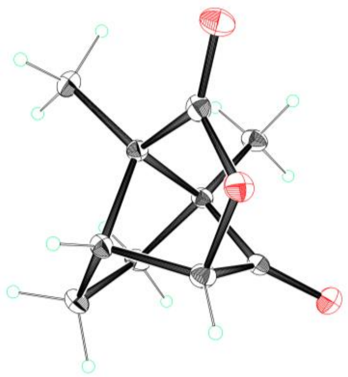

Table S21. Crystal data for **17b**.

**Chemical formula**	$C_{10}H_{12}O_3$	
**Formula weight**	180.20 g/mol	
**Temperature**	100(2) K	
**Wavelength**	0.71073 Å	
**Crystal size**	0.020 x 0.070 x 0.160 mm	
**Crystal habit**	colorless block	
**Crystal system**	tetragonal	
**Space group**	P 41 21 2	
**Unit cell dimensions**	a = 8.01990(10) Å	α = 90°
	b = 8.01990(10) Å	β = 90°
	c = 26.7822(5) Å	γ = 90°
**Volume**	1722.60(5) $Å^3$	
**Z**	8	
**Density (calculated)**	1.390 g/cm^3	
**Absorption coefficient**	0.102 mm^{-1}	
**F(000)**	768	
**Diffractometer**	Bruker D8 QUEST ECO three-circle diffractometer	
**Radiation source**	Ceramic x-ray tube (Mo Kα, λ = 0.71073 Å)	
**Theta range for data collection**	2.96 to 30.51°	
**Index ranges**	-11<=h<=11, -11<=k<=11, -38<=l<=37	
**Reflections collected**	58774	
**Independent reflections**	2637 [R(int) = 0.0379]	
**Coverage of independent reflections**	99.8%	
**Absorption correction**	Multi-Scan	
**Max. and min. transmission**	0.9980 and 0.9840	
**Structure solution technique**	direct methods	
**Structure solution program**	SHELXT 2014/5 (Sheldrick, 2014)	

**Refinement method**	Full-matrix least-squares on F^2
**Refinement program**	SHELXL-2017/1 (Sheldrick, 2017)
**Function minimized**	$\Sigma\ w(F_o^2 - F_c^2)^2$
**Data / restraints / parameters**	2637 / 0 / 155
**Goodness-of-fit on F^2**	1.144
**Δ/σ_{max}**	0.001
**Final R indices**	2526 data; $I>2\sigma(I)$ R1 = 0.0339, wR2 = 0.0838
	all data R1 = 0.0360, wR2 = 0.0849
**Weighting scheme**	$w=1/[\sigma^2(F_o^2)+(0.0441P)^2+0.4026P]$<br>where $P=(F_o^2+2F_c^2)/3$
**Absolute structure parameter**	-0.3(2)
**Largest diff. peak and hole**	0.379 and -0.180 eÅ^{-3}
**R.M.S. deviation from mean**	0.045 eÅ^{-3}

9.1.8. Solid state structure of **18a**.

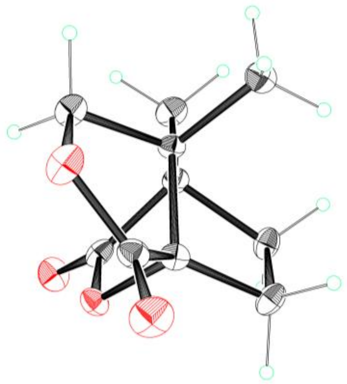

Table S22. Crystal data for **18a**.

**Chemical formula**	$C_{10}H_{12}O_4$	
**Formula weight**	196.20 g/mol	
**Temperature**	100(2) K	
**Wavelength**	0.71073 Å	
**Crystal size**	0.050 x 0.110 x 0.210 mm	
**Crystal habit**	colorless block	
**Crystal system**	orthorhombic	
**Space group**	P 21 21 21	
**Unit cell dimensions**	a = 6.9289(6) Å	α = 90°
	b = 7.2928(7) Å	β = 90°
	c = 18.4132(16) Å	γ = 90°
**Volume**	930.44(14) $Å^3$	
**Z**	4	
**Density (calculated)**	1.401 g/cm^3	
**Absorption coefficient**	0.109 mm^{-1}	
**F(000)**	416	
**Diffractometer**	Bruker D8 QUEST ECO three-circle diffractometer	
**Radiation source**	Ceramic x-ray tube (Mo Kα, λ = 0.71073 Å)	
**Theta range for data collection**	3.56 to 27.58°	
**Index ranges**	-8<=h<=8, -9<=k<=9, -23<=l<=23	
**Reflections collected**	28501	
**Independent reflections**	2139 [R(int) = 0.1014]	
**Coverage of independent reflections**	99.5%	
**Absorption correction**	Multi-Scan	
**Structure solution technique**	direct methods	
**Structure solution program**	SHELXT 2014/5 (Sheldrick, 2014)	

**Refinement method**	Full-matrix least-squares on F^2
**Refinement program**	SHELXL-2017/1 (Sheldrick, 2017)
**Function minimized**	$\Sigma\ w(F_o^2 - F_c^2)^2$
**Data / restraints / parameters**	2139 / 0 / 129
**Goodness-of-fit on F^2**	1.122
**Final R indices**	1811 data; $I>2\sigma(I)$ R1 = 0.0605, wR2 = 0.1081
	all data R1 = 0.0759, wR2 = 0.1130
**Weighting scheme**	$w=1/[\sigma^2(F_o^2)+(0.0279P)^2+1.0451P]$<br>where $P=(F_o^2+2F_c^2)/3$
**Absolute structure parameter**	-0.2(6)
**Largest diff. peak and hole**	0.227 and -0.304 eÅ^{-3}
**R.M.S. deviation from mean**	0.063 eÅ^{-3}

9.1.9. Solid state structure of **18b**.

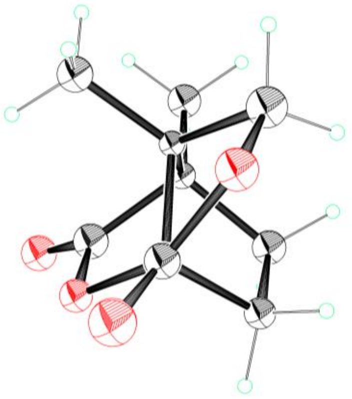

Table S23. Crystal data for **18b**.

**Chemical formula**	$C_{10}H_{12}O_4$	
**Formula weight**	196.20 g/mol	
**Temperature**	100(2) K	
**Wavelength**	0.71073 Å	
**Crystal size**	0.020 x 0.030 x 0.100 mm	
**Crystal habit**	colorless needle	
**Crystal system**	orthorhombic	
**Space group**	P 21 21 21	
**Unit cell dimensions**	a = 6.479(4) Å	α = 90°
	b = 9.450(6) Å	β = 90°
	c = 14.895(8) Å	γ = 90°
**Volume**	912.0(10) $Å^3$	
**Z**	4	
**Density (calculated)**	1.429 g/cm^3	
**Absorption coefficient**	0.111 mm^{-1}	
**F(000)**	416	
**Diffractometer**	Bruker D8 QUEST ECO three-circle diffractometer	
**Radiation source**	Ceramic x-ray tube (Mo Kα, λ = 0.71073 Å)	
**Theta range for data collection**	2.55 to 17.22°	
**Index ranges**	-5<=h<=5, -7<=k<=7, -12<=l<=12	
**Reflections collected**	1692	
**Independent reflections**	555 [R(int) = 0.0835]	
**Coverage of independent reflections**	99.4%	
**Absorption correction**	Multi-Scan	
**Max. and min. transmission**	0.9980 and 0.9890	
**Structure solution technique**	direct methods	

**Structure solution program**	SHELXT 2014/5 (Sheldrick, 2014)
**Refinement method**	Full-matrix least-squares on F^2
**Refinement program**	SHELXL-2017/1 (Sheldrick, 2017)
**Function minimized**	$\Sigma\ w(F_o^2 - F_c^2)^2$
**Data / restraints / parameters**	555 / 0 / 59
**Goodness-of-fit on F^2**	1.093
**Final R indices**	444 data; $I>2\sigma(I)$ R1 = 0.0505, wR2 = 0.0957
	all data R1 = 0.0768, wR2 = 0.1233
**Weighting scheme**	$w=1/[\sigma^2(F_o^2)+(0.0315P)^2+1.9127P]$ where $P=(F_o^2+2F_c^2)/3$
**Absolute structure parameter**	-5.4(10)
**Largest diff. peak and hole**	0.175 and -0.296 eÅ^{-3}
**R.M.S. deviation from mean**	0.054 eÅ^{-3}

9.1.10. Solid state structure of **19a**.

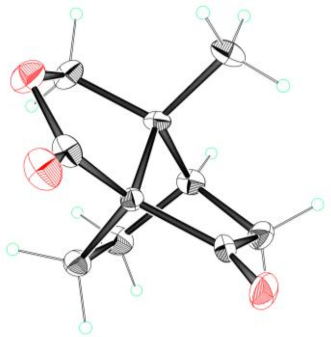

Table S24. Crystal data for of **19a**.

**Chemical formula**	$C_{10}H_{12}O_3$	
**Formula weight**	180.20 g/mol	
**Temperature**	100(2) K	
**Wavelength**	0.71073 Å	
**Crystal size**	0.020 x 0.150 x 0.300 mm	
**Crystal habit**	colorless plate	
**Crystal system**	hexagonal	
**Space group**	P 61	
**Unit cell dimensions**	a = 6.6024(2) Å	α = 90°
	b = 6.6024(2) Å	β = 90°
	c = 34.3244(11) Å	γ = 120°
**Volume**	1295.80(9) $Å^3$	
**Z**	6	
**Density (calculated)**	1.386 g/cm^3	
**Absorption coefficient**	0.102 mm^{-1}	
**F(000)**	576	
**Diffractometer**	Bruker D8 QUEST ECO three-circle diffractometer	
**Radiation source**	Ceramic x-ray tube (Mo Kα, λ = 0.71073 Å)	
**Theta range for data collection**	3.56 to 30.08°	
**Reflections collected**	2548	
**Independent reflections**	2548 [R(int) = 0.0554]	
**Coverage of independent reflections**	99.8%	
**Absorption correction**	Multi-Scan	
**Max. and min. transmission**	0.9980 and 0.9700	
**Structure solution technique**	direct methods	
**Structure solution program**	SHELXT 2014/5 (Sheldrick, 2014)	
**Refinement method**	Full-matrix least-squares on F^2	

**Refinement program**	SHELXL-2017/1 (Sheldrick, 2017)
**Function minimized**	Σ w(F_o^2 - F_c^2)^2
**Data / restraints / parameters**	2548 / 1 / 120
**Goodness-of-fit on F^2**	1.257
**Final R indices**	2546 data; I>2σ(I) R1 = 0.0531, wR2 = 0.1349
	all data R1 = 0.0531, wR2 = 0.1350
**Weighting scheme**	w=1/[σ^2(F_o^2)+(0.0474P)^2+1.0649P]<br>where P=(F_o^2+2F_c^2)/3
**Absolute structure parameter**	-0.2(2)
**Largest diff. peak and hole**	0.336 and -0.264 eÅ^{-3}
**R.M.S. deviation from mean**	0.064 eÅ^{-3}

9.1.11. Solid state structure of **19b**.

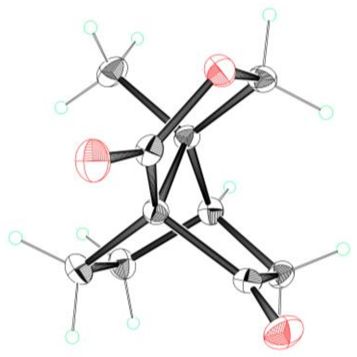

Table S25. Crystal data for of **19b**.

**Chemical formula**	$C_{10}H_{12}O_3$	
**Formula weight**	180.20 g/mol	
**Temperature**	100(2) K	
**Wavelength**	0.71076 Å	
**Crystal size**	0.150 x 0.270 x 0.300 mm	
**Crystal habit**	colorless block	
**Crystal system**	orthorhombic	
**Space group**	P 21 21 21	
**Unit cell dimensions**	a = 7.085(7) Å	α = 90°
	b = 10.299(11) Å	β = 90°
	c = 12.163(10) Å	γ = 90°
**Volume**	887.5(14) Å^3	
**Z**	4	
**Density (calculated)**	1.349 g/cm^3	
**Absorption coefficient**	0.099 mm^{-1}	
**F(000)**	384	
**Diffractometer**	Bruker D8 QUEST ECO three-circle diffractometer	
**Radiation source**	Ceramic x-ray tube (Mo Kα, λ = 0.71076 Å)	
**Theta range for data collection**	3.35 to 30.58°	
**Index ranges**	-10<=h<=10, -14<=k<=14, -17<=l<=17	
**Reflections collected**	48018	
**Independent reflections**	2707 [R(int) = 0.0330]	
**Coverage of independent reflections**	99.5%	
**Absorption correction**	Multi-Scan	
**Max. and min. transmission**	0.7461 and 0.6929	
**Structure solution technique**	direct methods	
**Structure solution program**	SHELXT 2014/5 (Sheldrick, 2014)	

**Refinement method**	Full-matrix least-squares on F^2	
**Refinement program**	SHELXL-2017/1 (Sheldrick, 2017)	
**Function minimized**	$\Sigma\ w(F_o^2 - F_c^2)^2$	
**Data / restraints / parameters**	2707 / 0 / 119	
**Goodness-of-fit on F^2**	1.110	
**Final R indices**	2565 data; $I>2\sigma(I)$	R1 = 0.0298, wR2 = 0.0758
	all data	R1 = 0.0334, wR2 = 0.0794
**Weighting scheme**	$w=1/[\sigma^2(F_o^2)+(0.0403P)^2+0.1784P]$<br>where $P=(F_o^2+2F_c^2)/3$	
**Absolute structure parameter**	-0.16(17)	
**Largest diff. peak and hole**	0.323 and -0.173 eÅ^{-3}	
**R.M.S. deviation from mean**	0.040 eÅ^{-3}	

9.1.12. Solid state structure of **19b-I**.

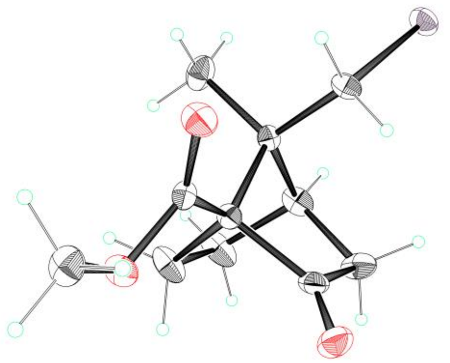

Table S26. Crystal data for of **19b-I**.

**Chemical formula**	$C_{11}H_{15}IO_3$	
**Formula weight**	322.13 g/mol	
**Temperature**	100(2) K	
**Wavelength**	0.71073 Å	
**Crystal size**	0.030 x 0.080 x 0.180 mm	
**Crystal habit**	colorless block	
**Crystal system**	orthorhombic	
**Space group**	P 21 21 21	
**Unit cell dimensions**	a = 7.3230(3) Å	α = 90°
	b = 9.5037(5) Å	β = 90°
	c = 17.0127(8) Å	γ = 90°
**Volume**	1184.01(10) $Å^3$	
**Z**	4	
**Density (calculated)**	1.807 g/cm^3	
**Absorption coefficient**	2.690 mm^{-1}	
**F(000)**	632	
**Diffractometer**	Bruker D8 QUEST ECO three-circle diffractometer	
**Radiation source**	Ceramic x-ray tube (Mo Kα, λ = 0.71073 Å)	
**Theta range for data collection**	3.51 to 27.48°	
**Index ranges**	-9<=h<=8, -12<=k<=12, -22<=l<=21	
**Reflections collected**	13939	
**Independent reflections**	2711 [R(int) = 0.0430]	
**Coverage of independent reflections**	99.7%	
**Absorption correction**	Multi-Scan	
**Max. and min. transmission**	0.9240 and 0.6430	
**Structure solution technique**	direct methods	

**Structure solution program**	SHELXT 2014/5 (Sheldrick, 2014)
**Refinement method**	Full-matrix least-squares on F^2
**Refinement program**	SHELXL-2017/1 (Sheldrick, 2017)
**Function minimized**	$\Sigma\ w(F_o^2 - F_c^2)^2$
**Data / restraints / parameters**	2711 / 0 / 138
**Goodness-of-fit on F^2**	1.092
**Final R indices**	2614 data; $I>2\sigma(I)$ R1 = 0.0193, wR2 = 0.0427
	all data R1 = 0.0215, wR2 = 0.0438
**Weighting scheme**	$w=1/[\sigma^2(F_o^2)+(0.0129P)^2+0.3585P]$<br>where $P=(F_o^2+2F_c^2)/3$
**Absolute structure parameter**	-0.026(16)
**Largest diff. peak and hole**	0.449 and -0.614 eÅ^{-3}
**R.M.S. deviation from mean**	0.073 eÅ^{-3}

9.2. NMR spectra

^{1}H-NMR of **6** in $CDCl_3$

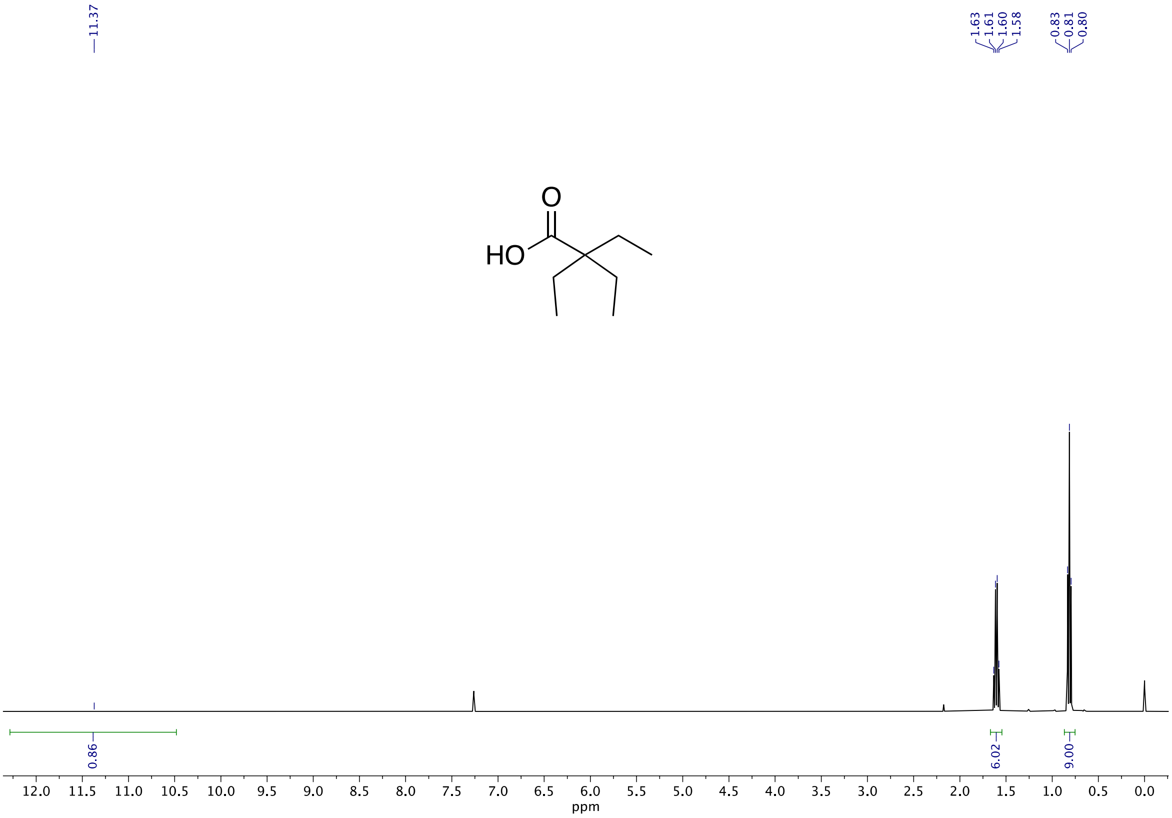

^{13}C-NMR of **6** in $CDCl_3$

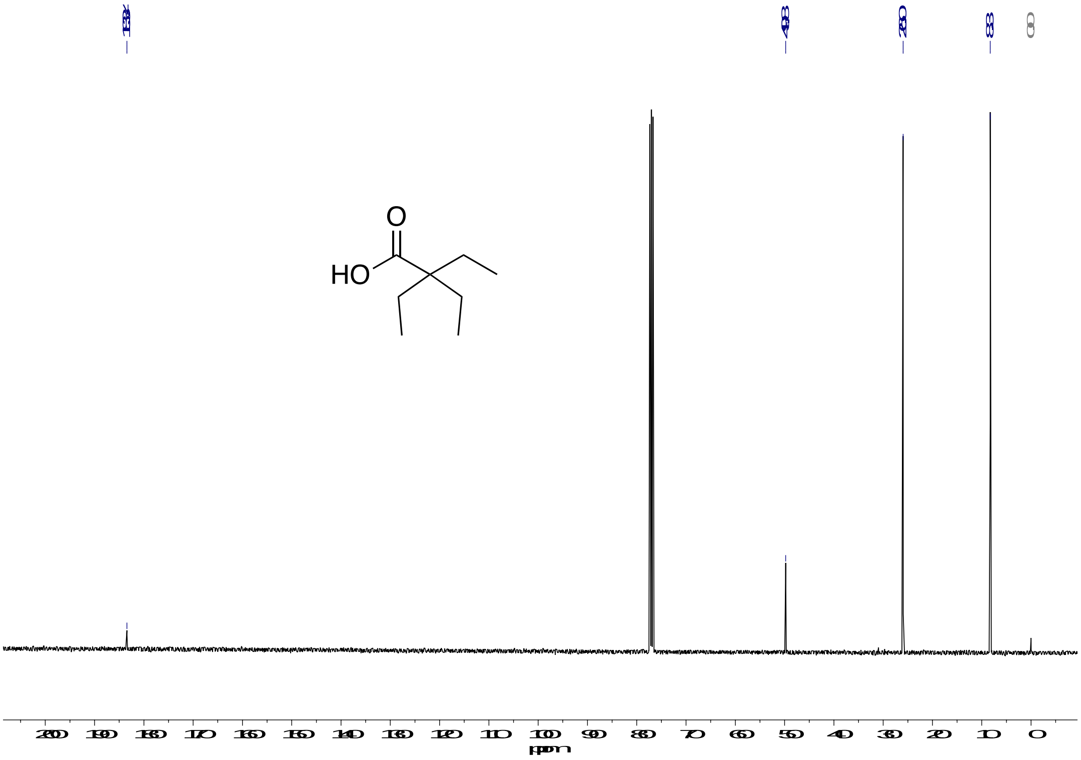

^{1}H-NMR of **int-*d5*** in $CDCl_3$

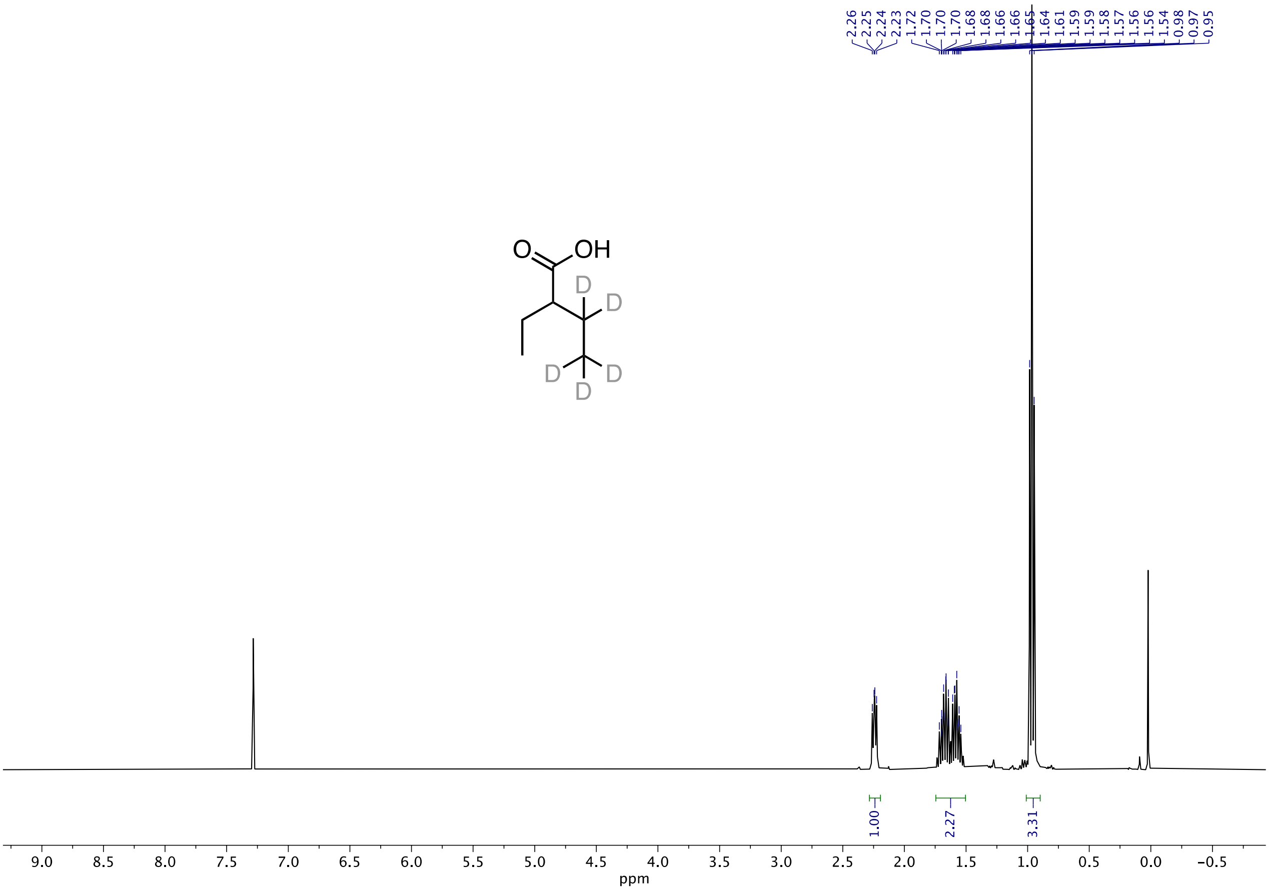

^{13}C-NMR of **int-*d5*** in $CDCl_3$

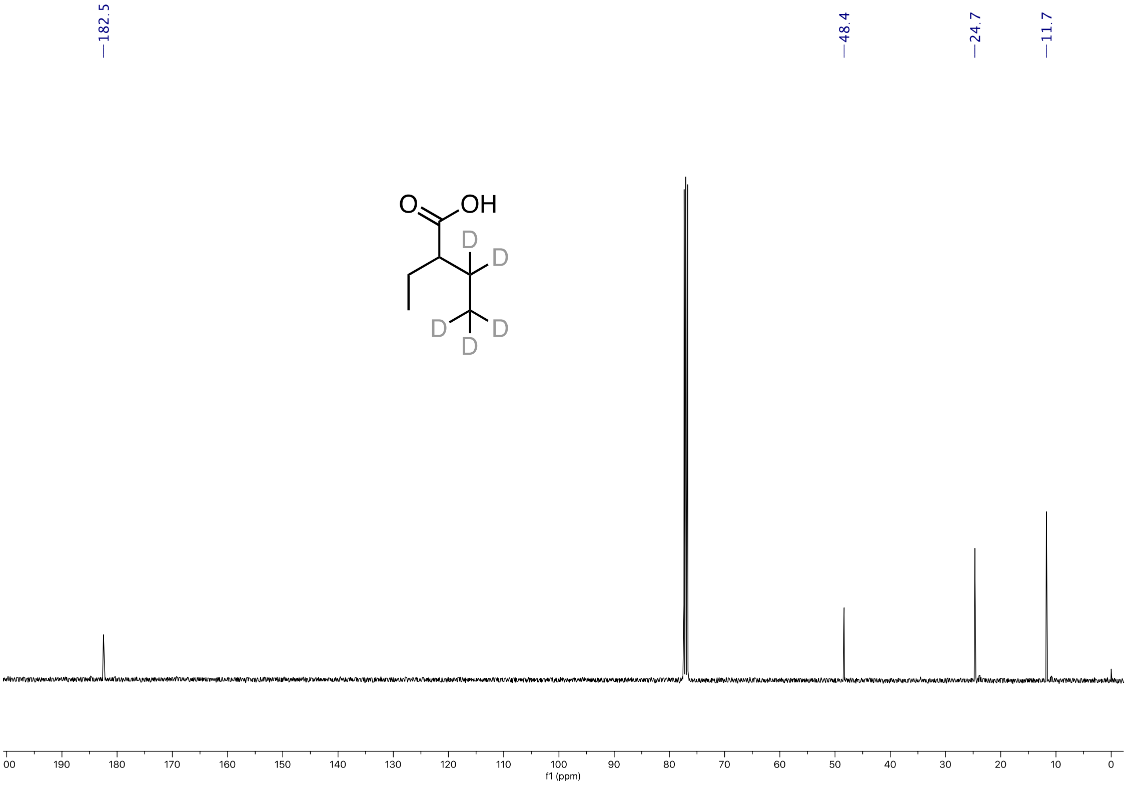

^{1}H-NMR of **6-d_{10}** in $CDCl_3$

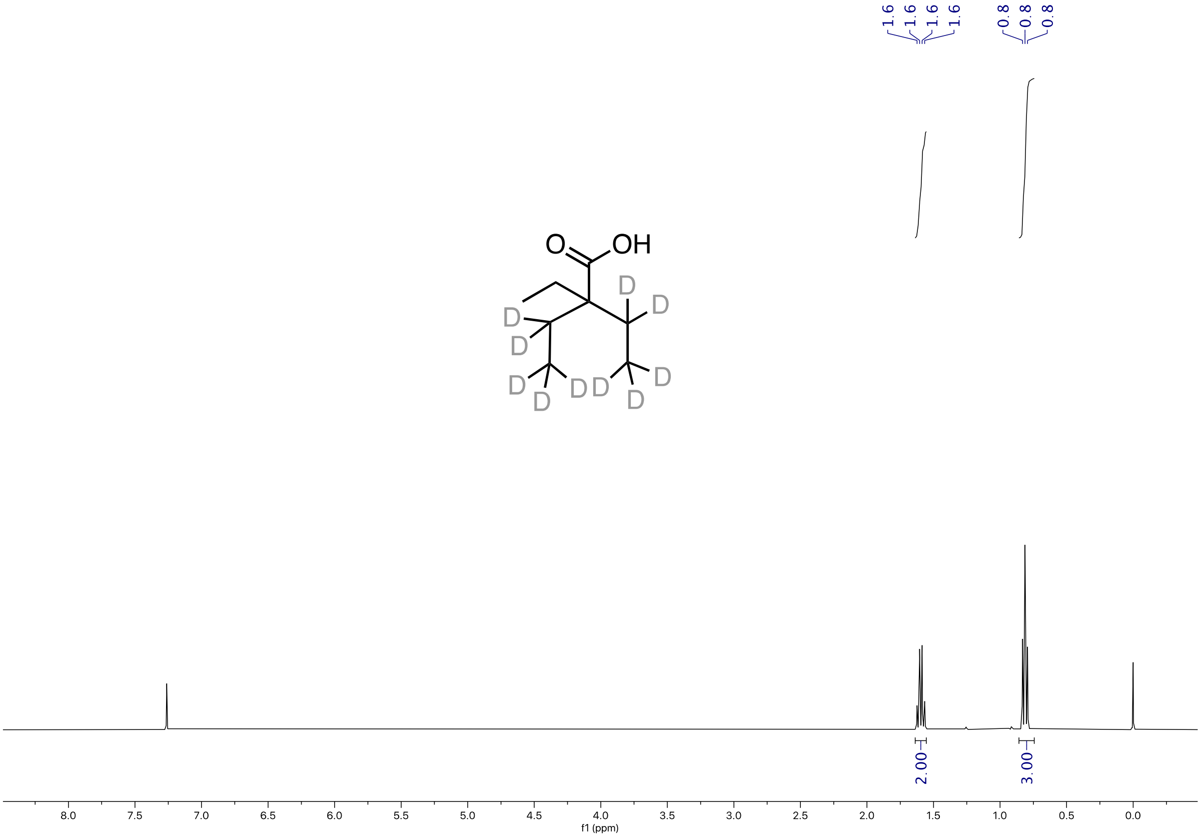

^{13}C-NMR of **6-d_{10}** in $CDCl_3$

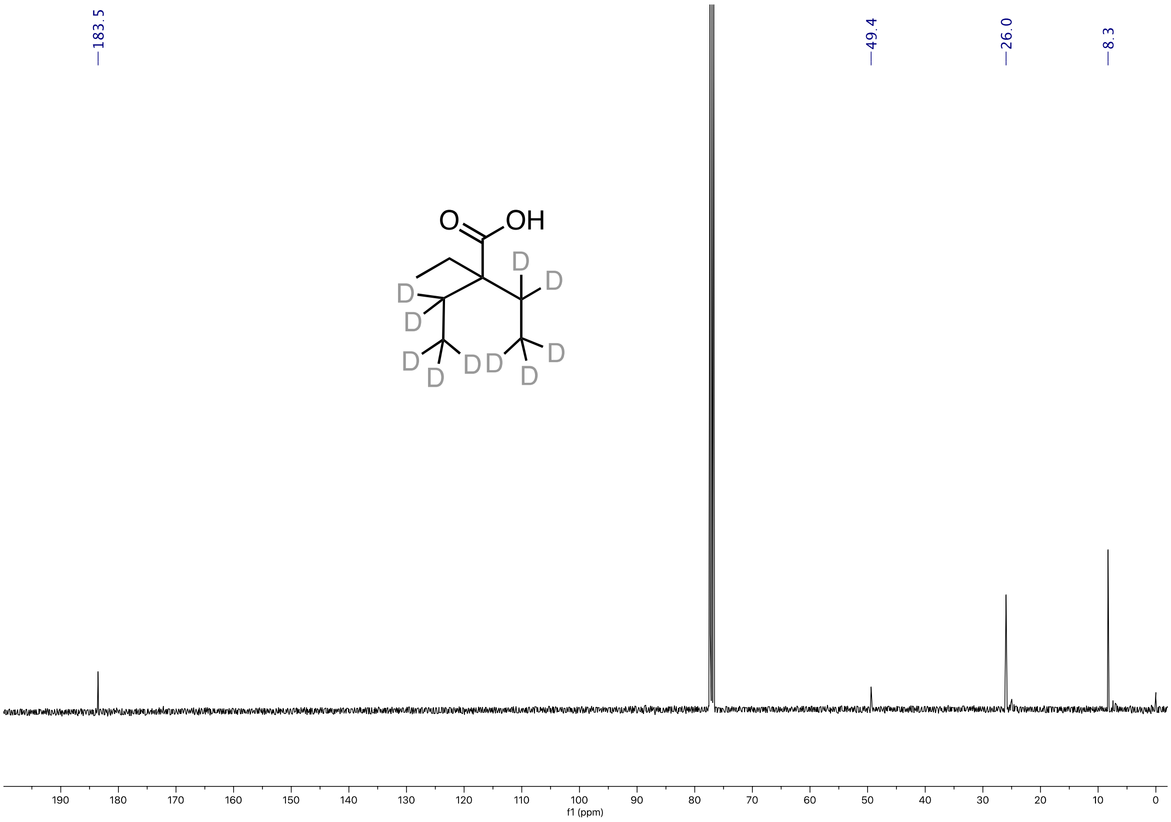

^{1}H-NMR of **6-*d*$_5$** in $CDCl_3$

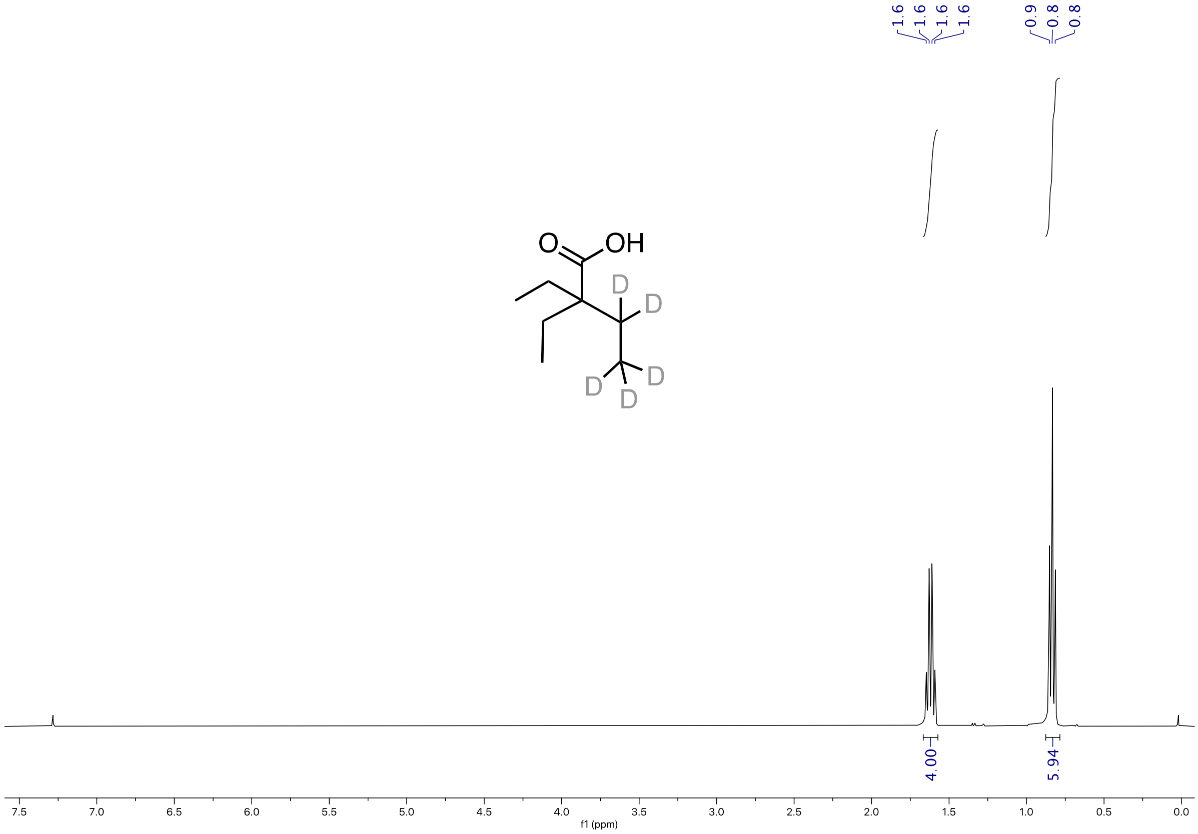

^{13}C-NMR of **6-*d*$_5$** in $CDCl_3$

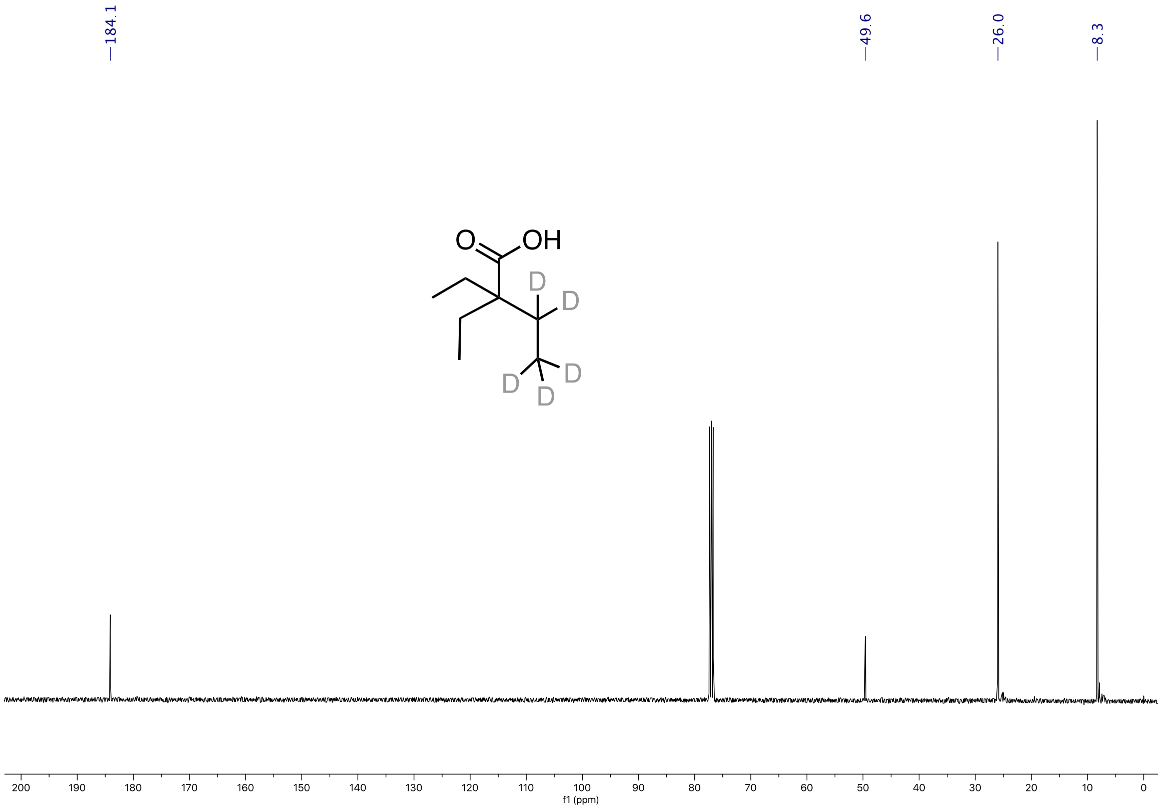

^{1}H-NMR of **7** in $CDCl_3$

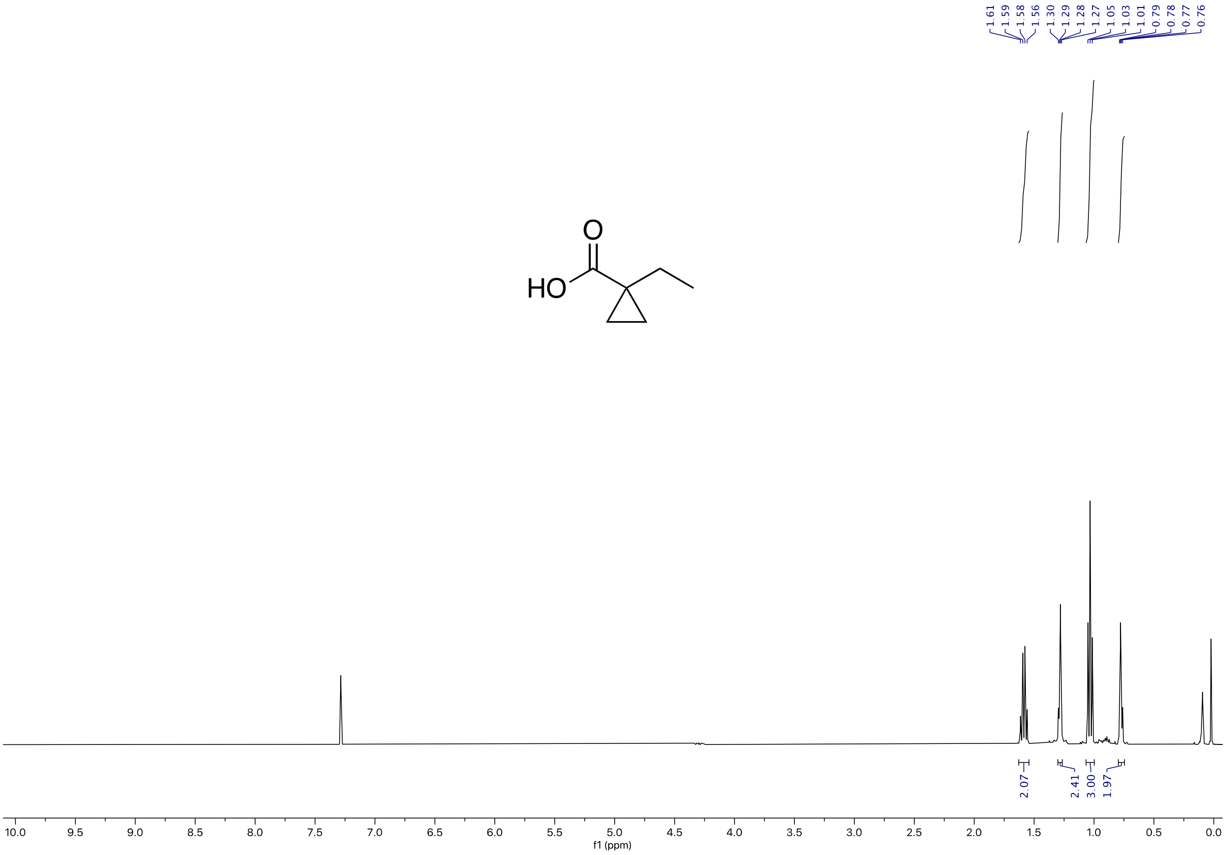

^{13}C-NMR of **7** in $CDCl_3$

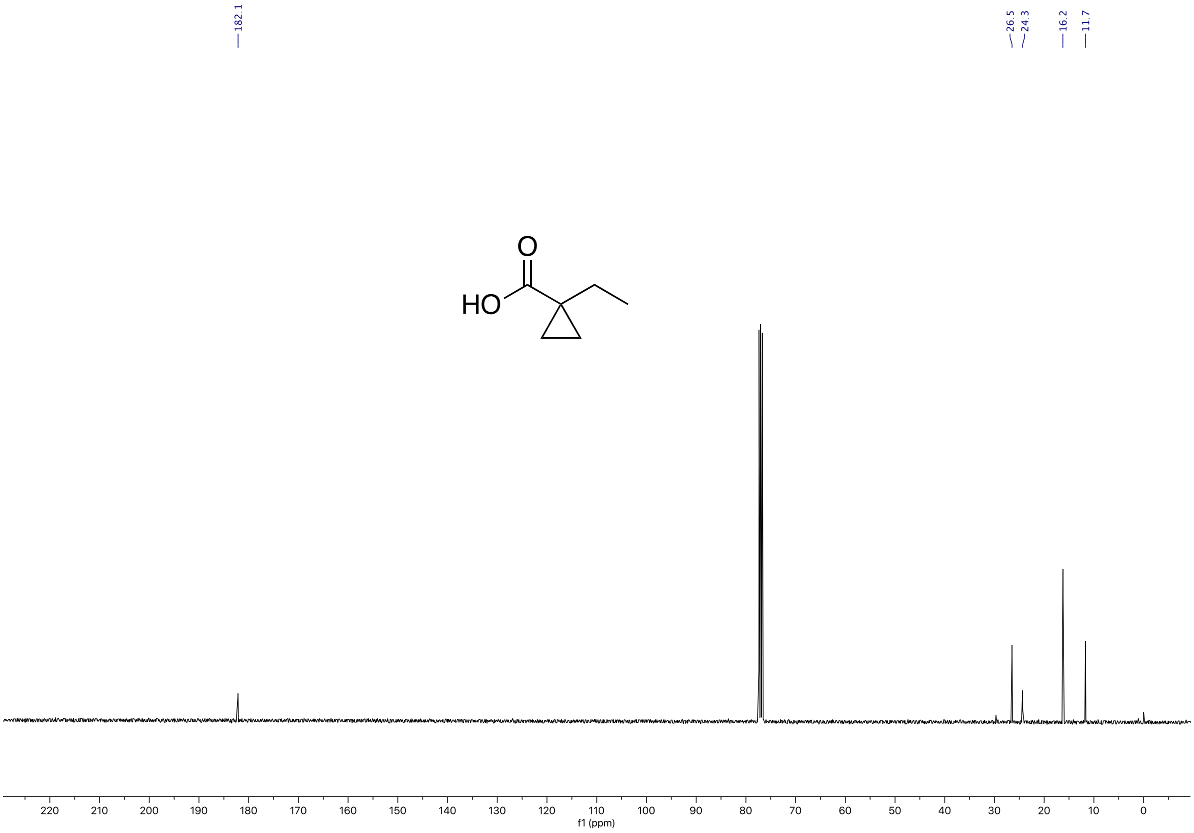

^{1}H-NMR of **8** in $CDCl_3$

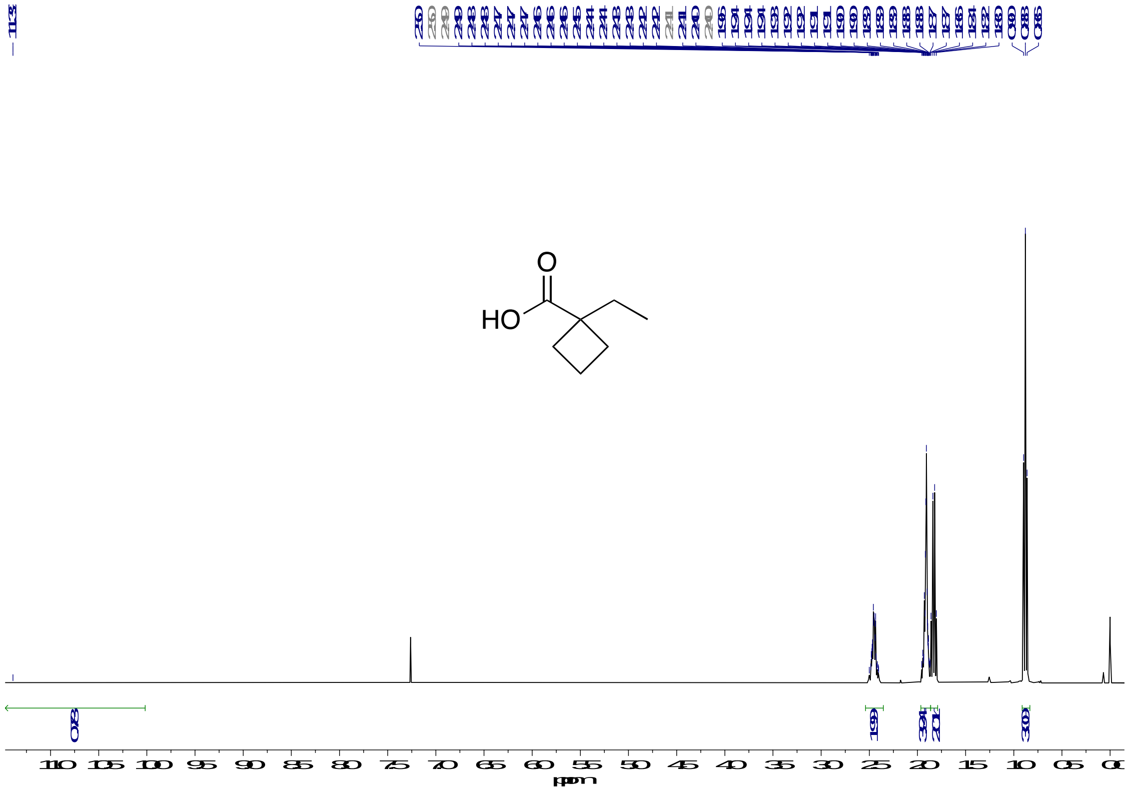

^{13}C-NMR of **8** in $CDCl_3$

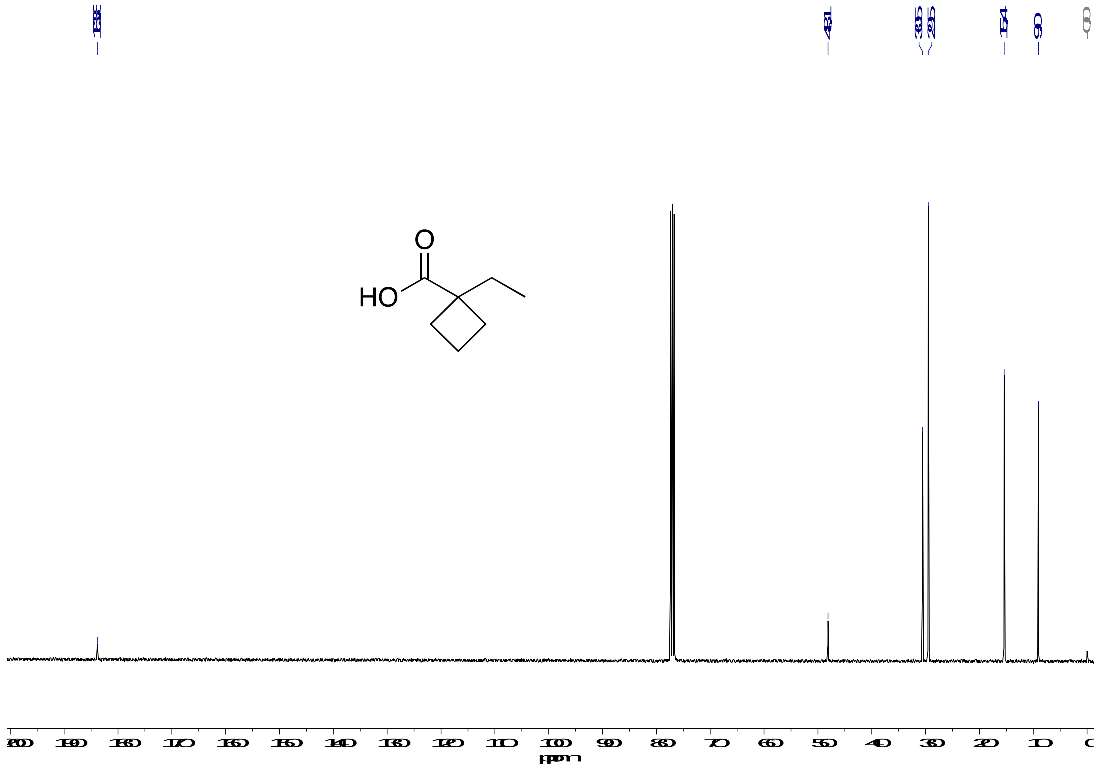

^{1}H-NMR of **9** in $CDCl_3$

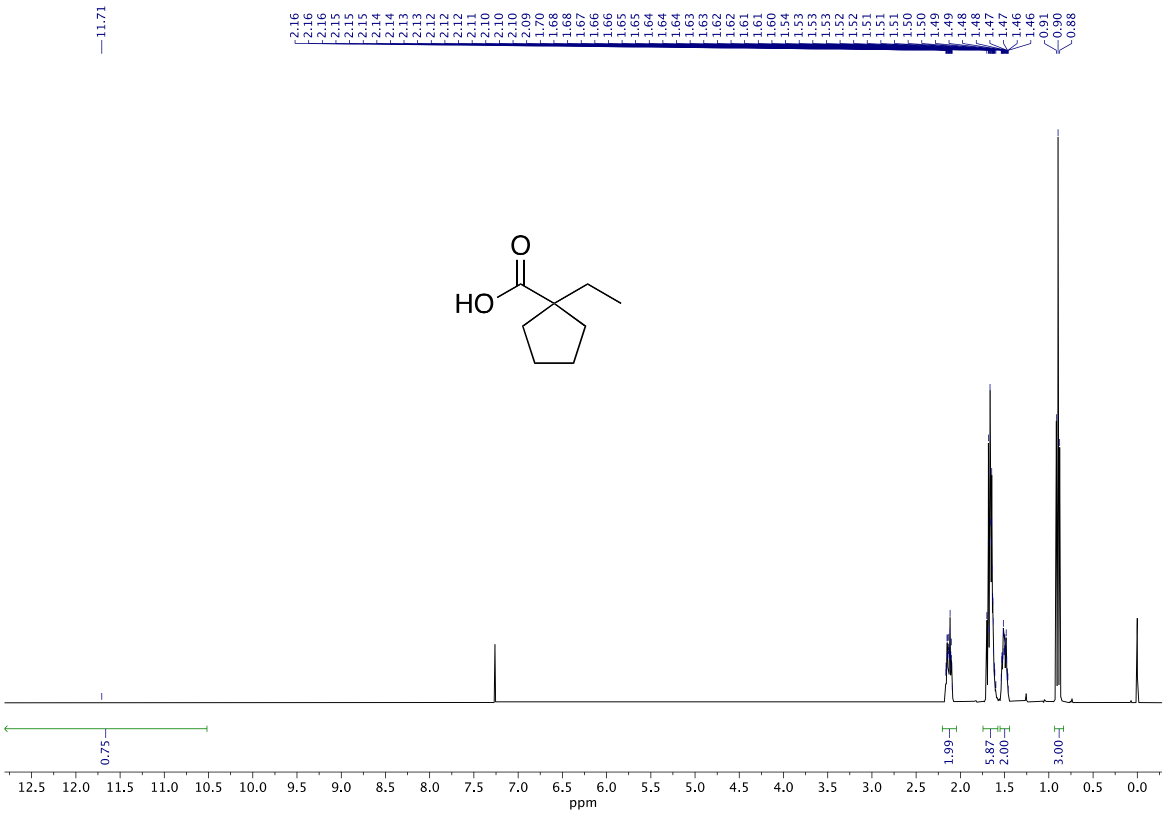

^{13}C-NMR of **9** in $CDCl_3$

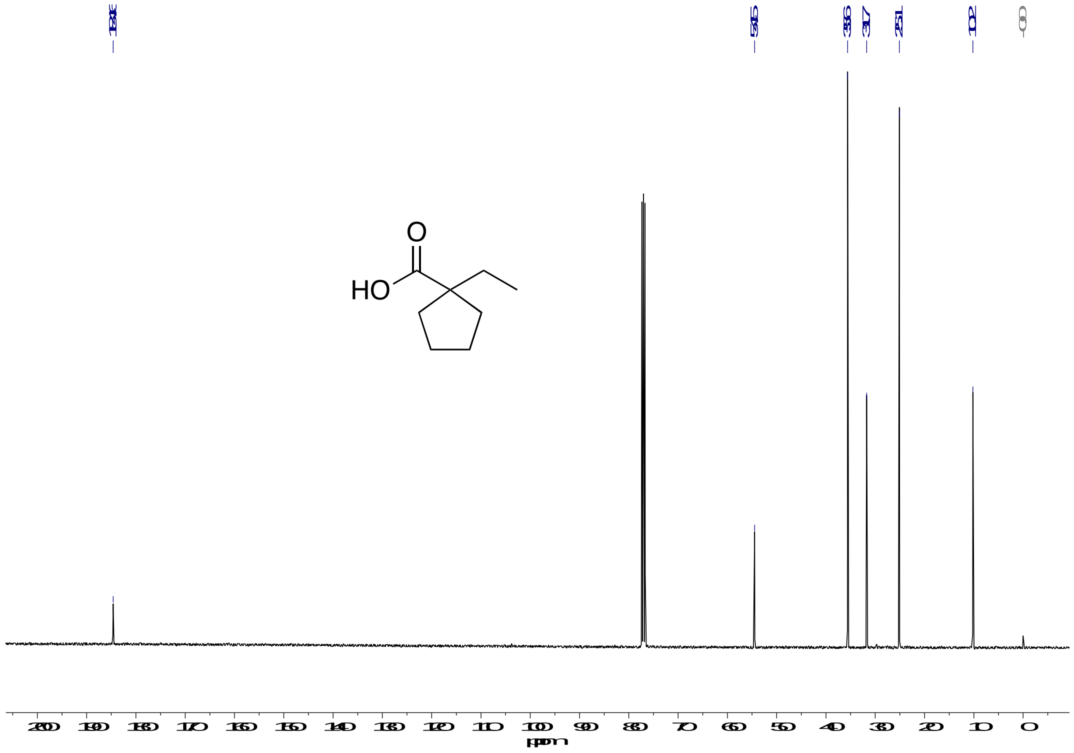

^{1}H-NMR of **10** in $CDCl_3$

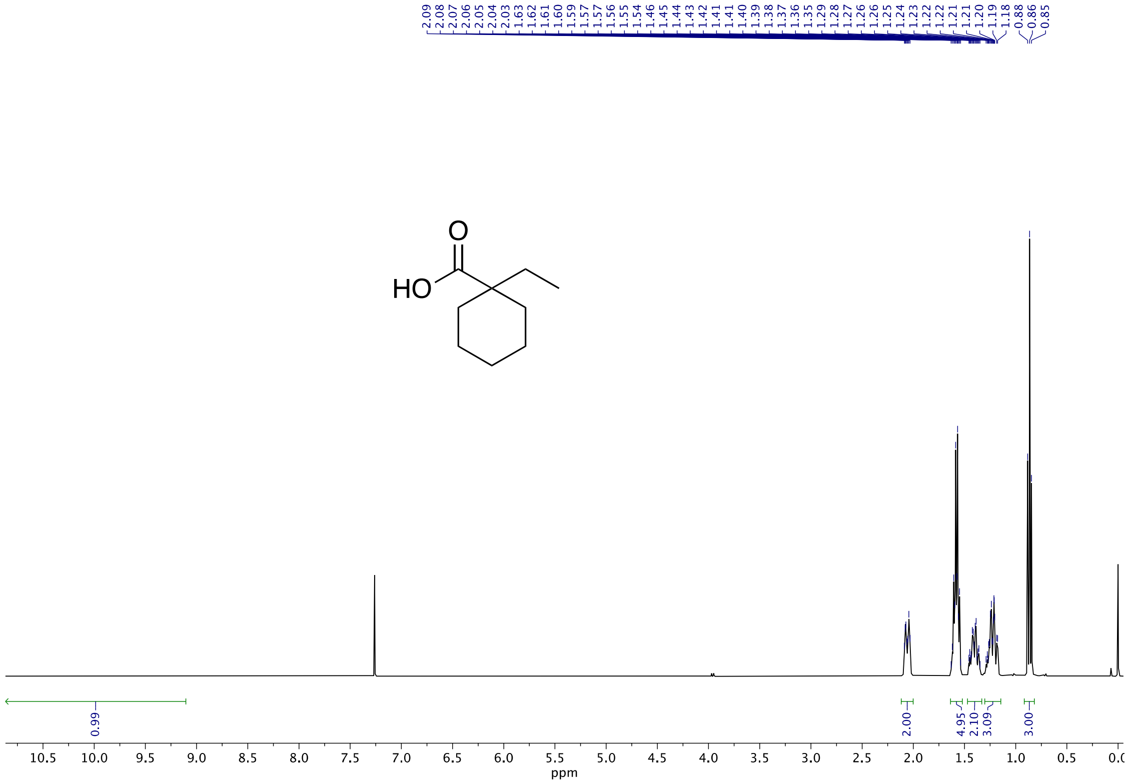

^{13}C-NMR of **10** in $CDCl_3$

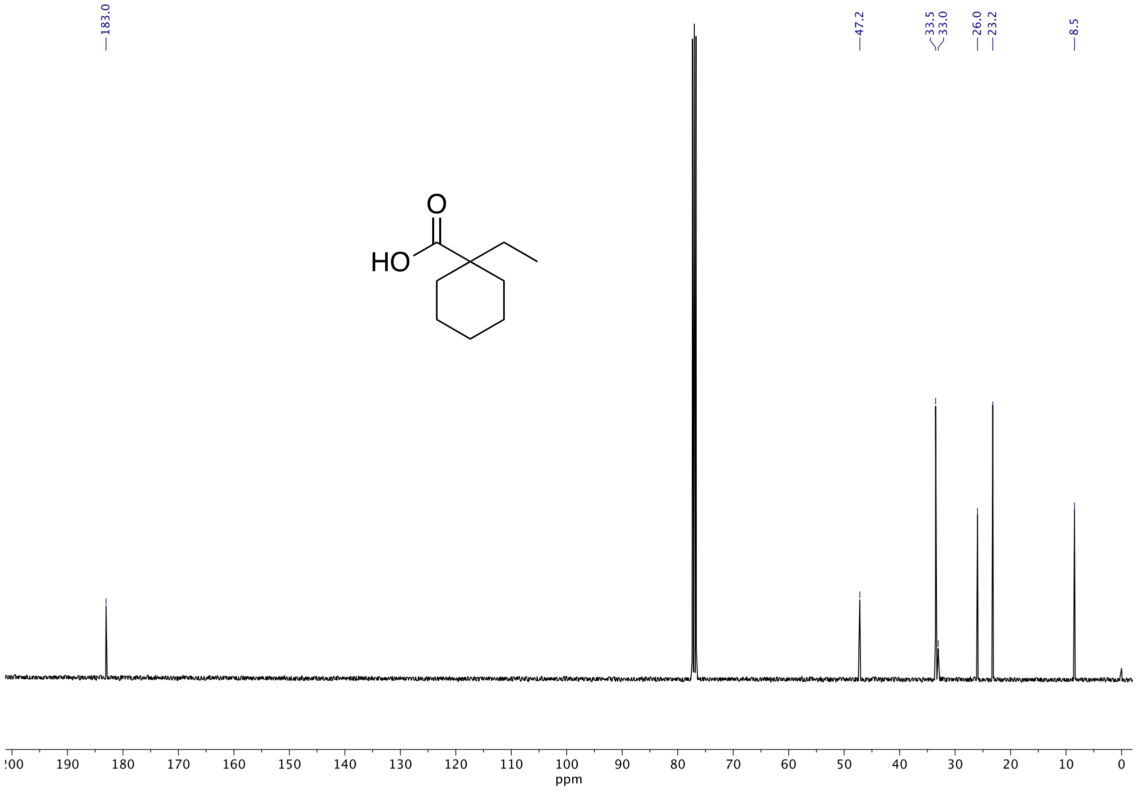

^{1}H-NMR of **12** in $CDCl_3$

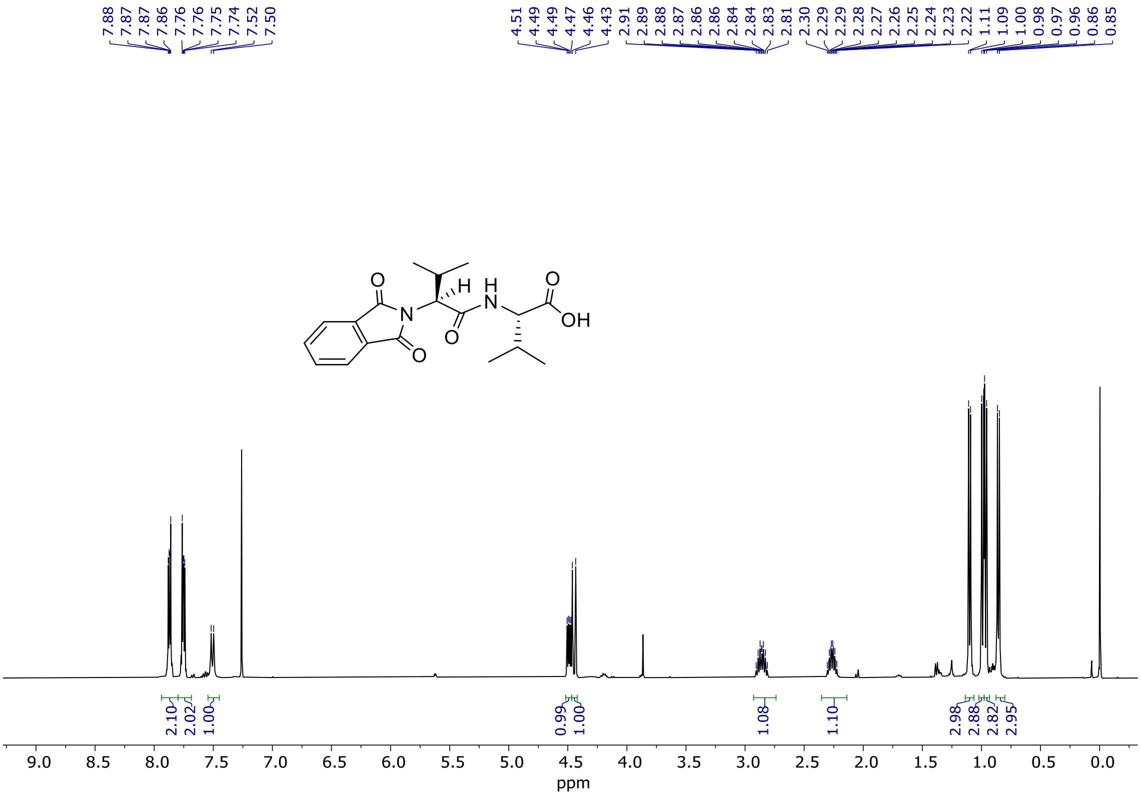

^{13}C-NMR of **12** in $CDCl_3$

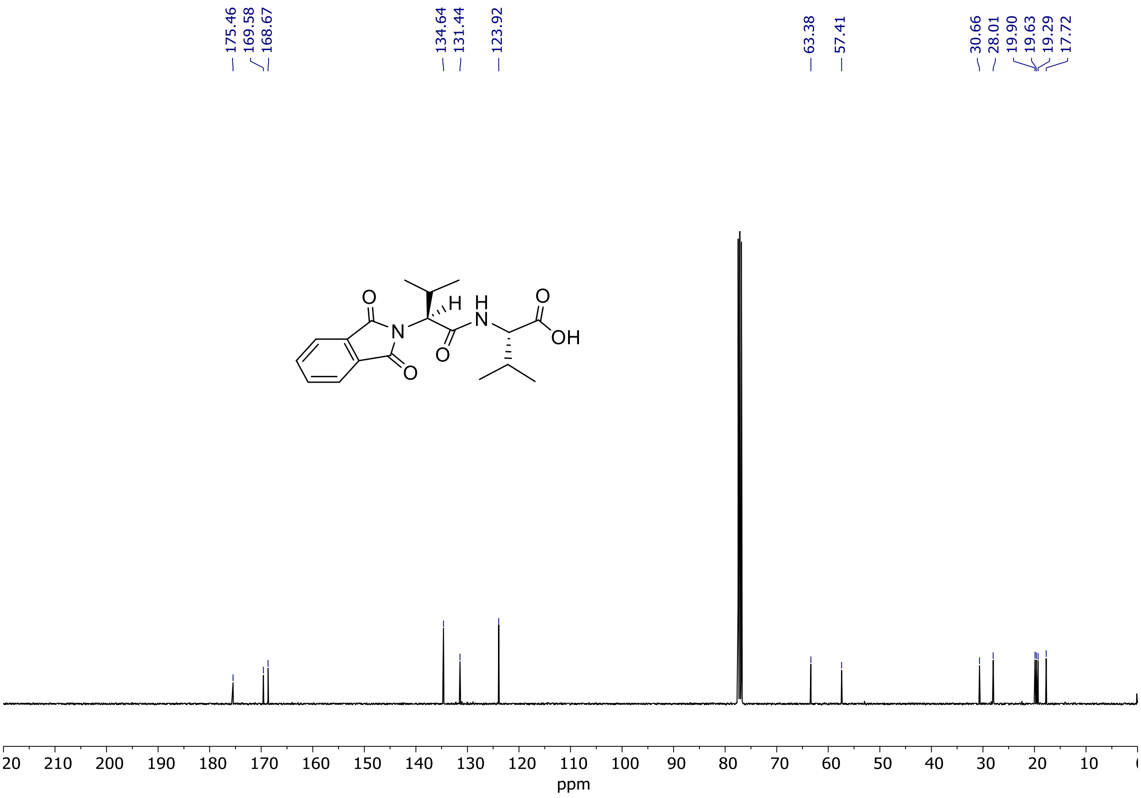

^{1}H-NMR of **13** in $CDCl_3$

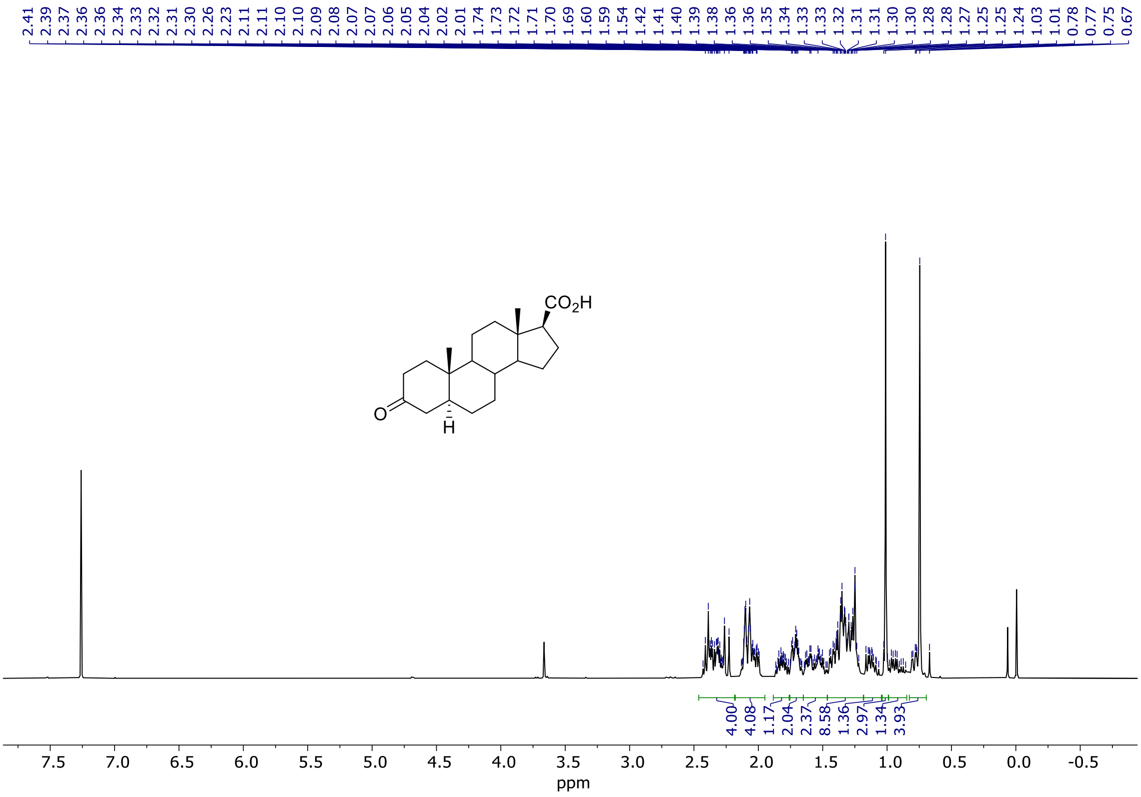

^{13}C-NMR of **13** in $CDCl_3$

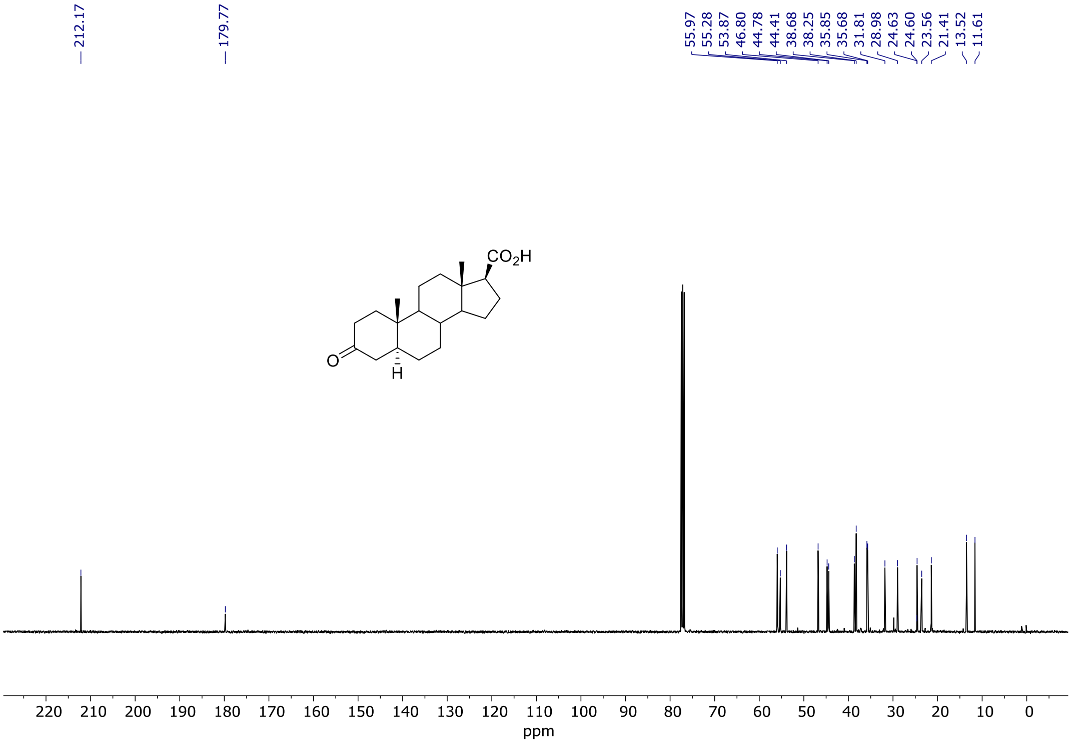

^{1}H-NMR of **15** in CDCl$_3$

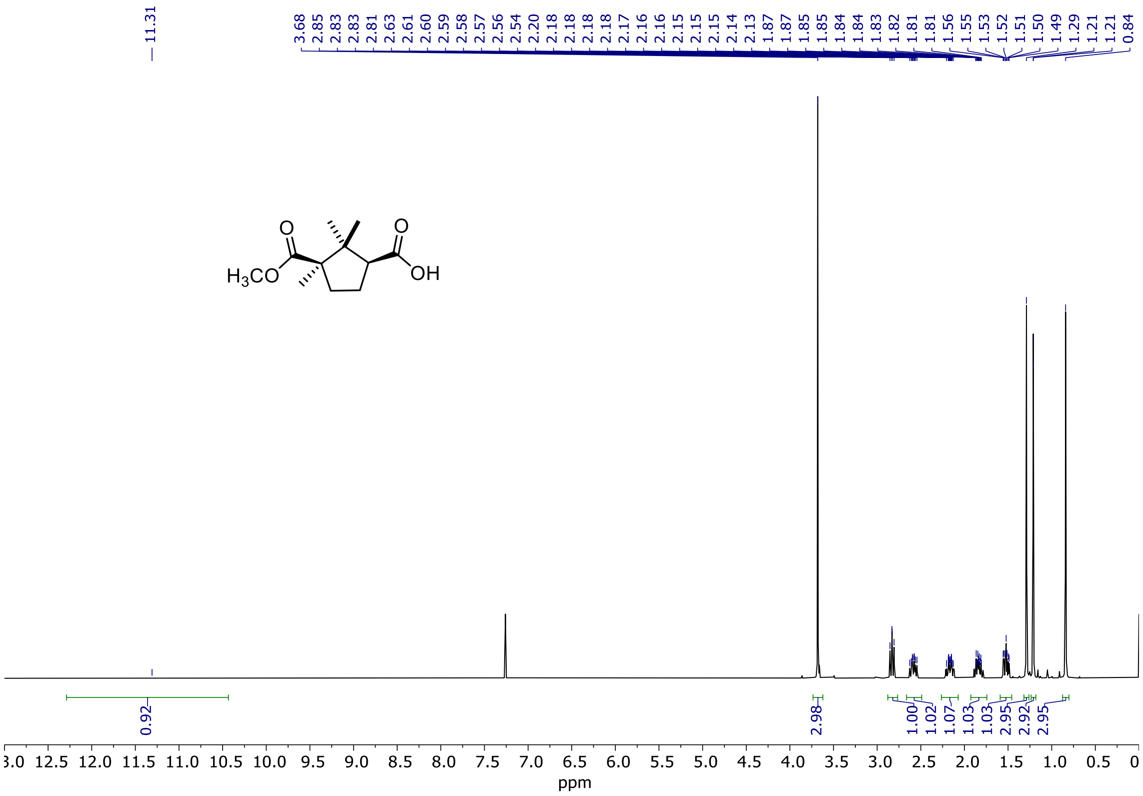

^{13}C-NMR of **15** in CDCl$_3$

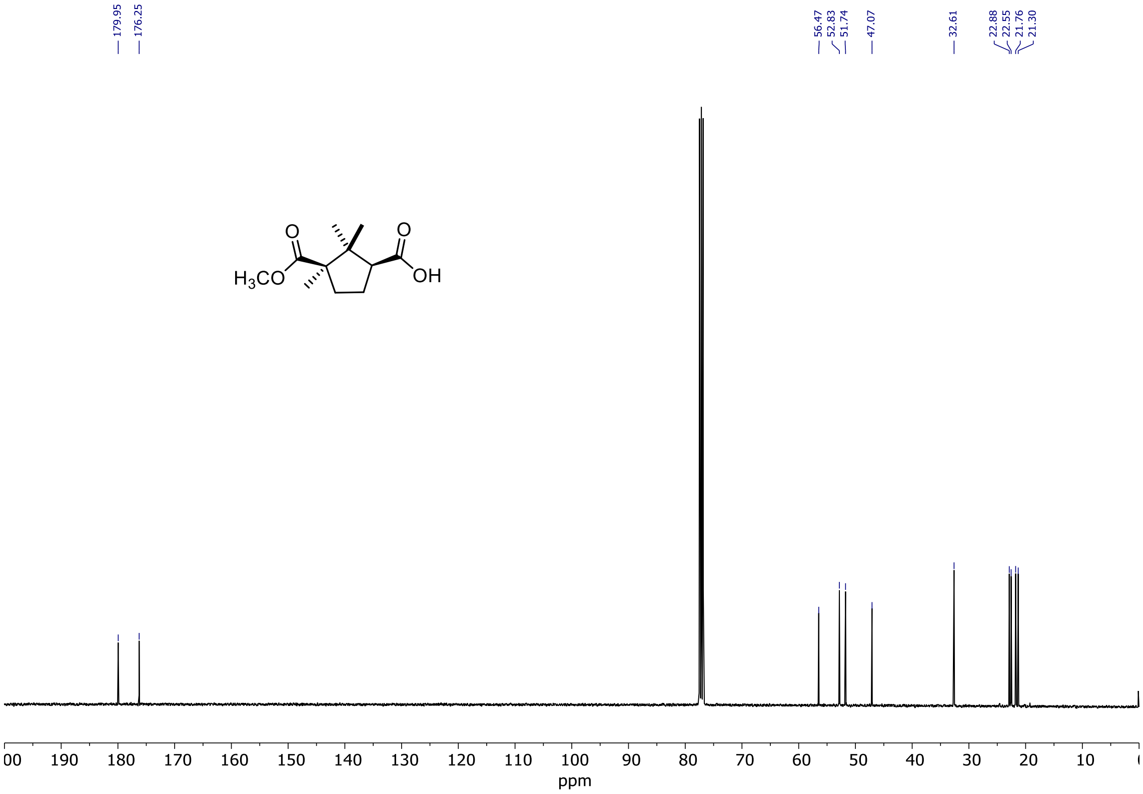

^{1}H-NMR of **16** in $CDCl_3$

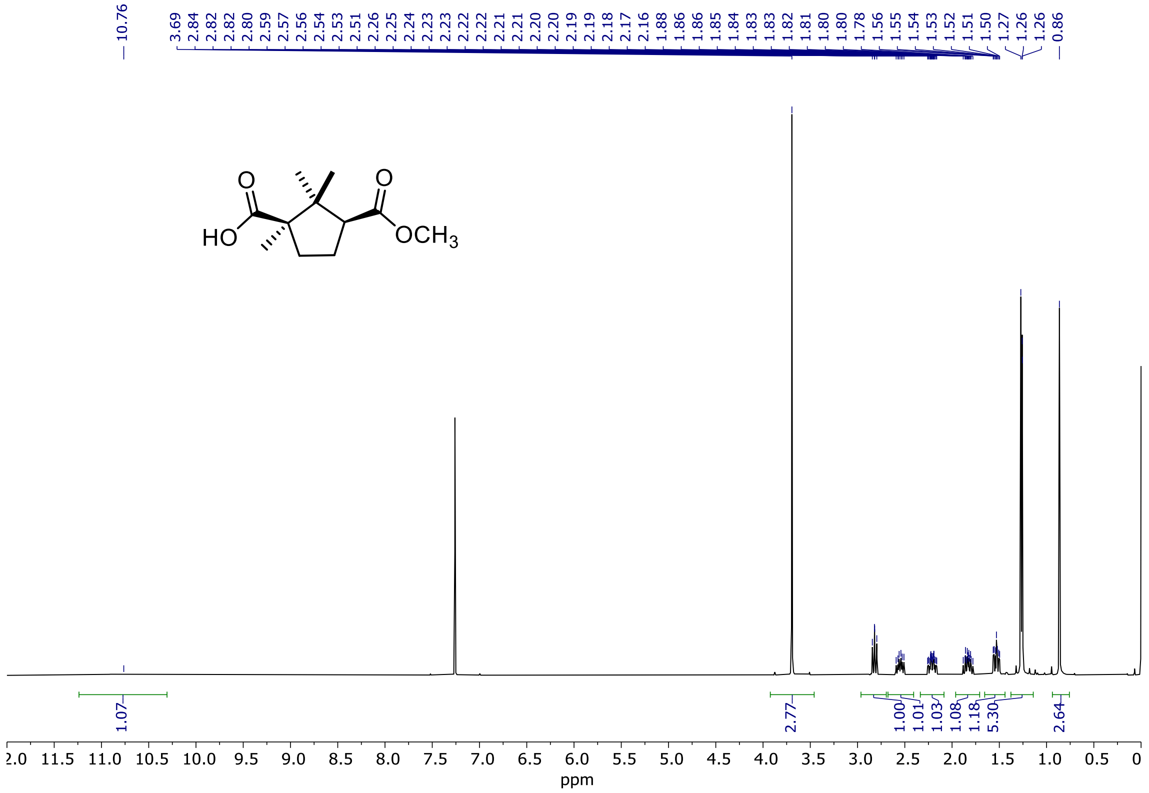

^{13}C-NMR of **16** in $CDCl_3$

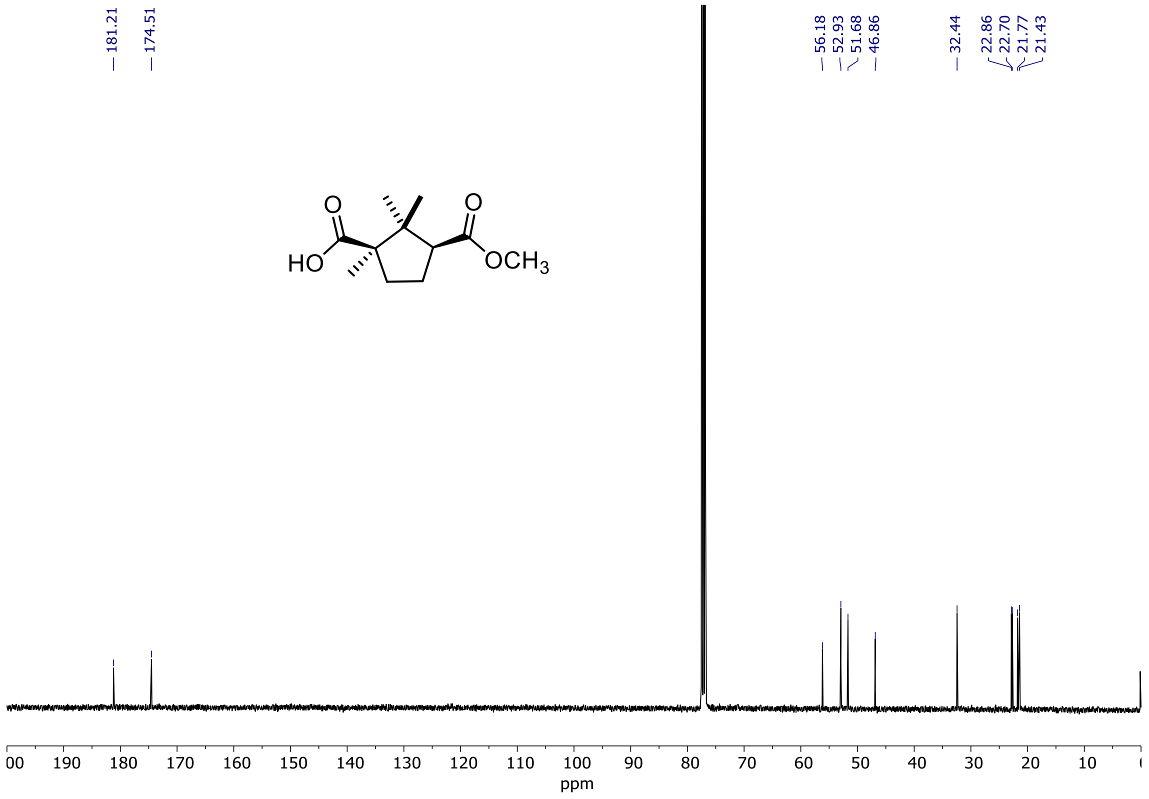

^{1}H-NMR of **1a** in $CDCl_3$

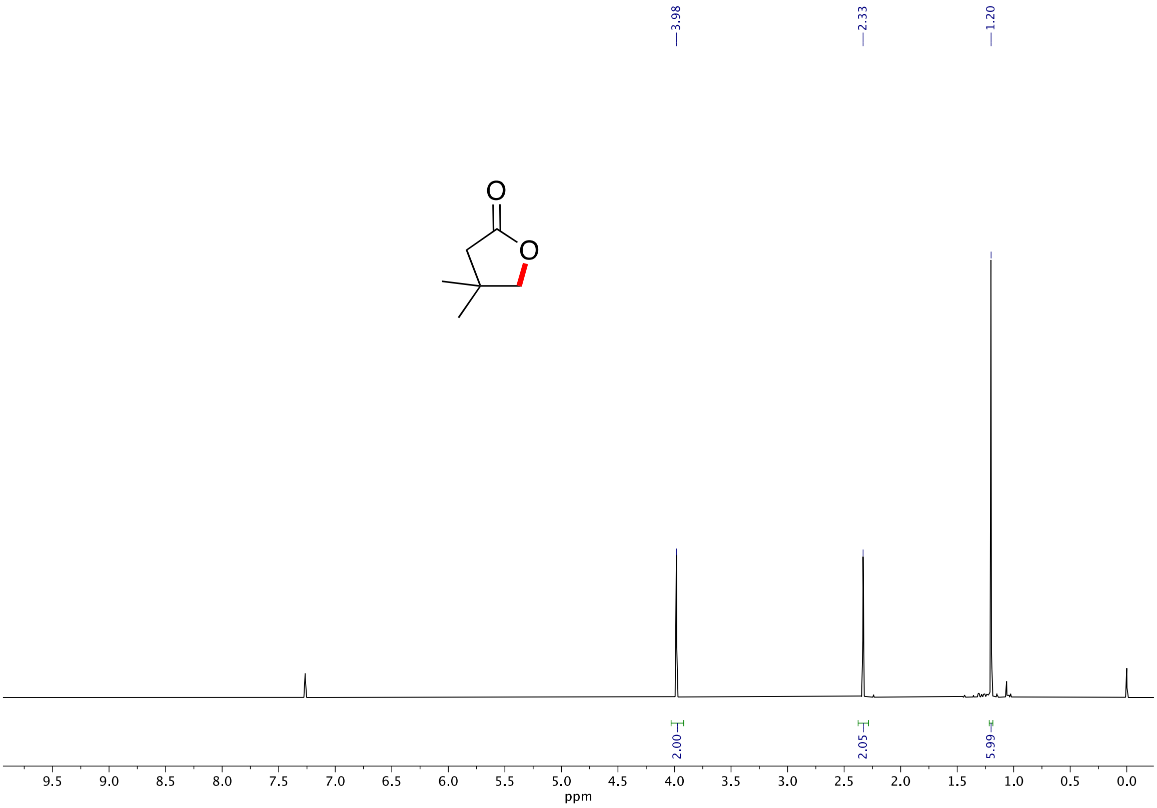

^{13}C-NMR of **1a** in $CDCl_3$

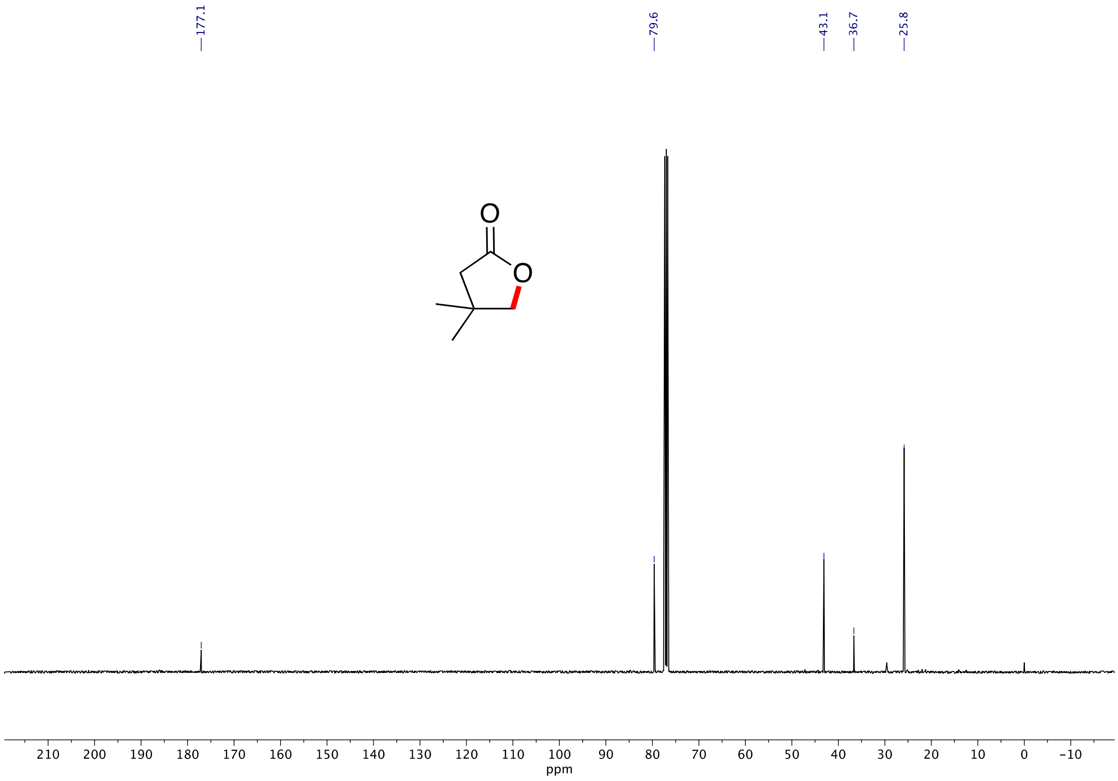

^{1}H-NMR of **5a** in $CDCl_3$

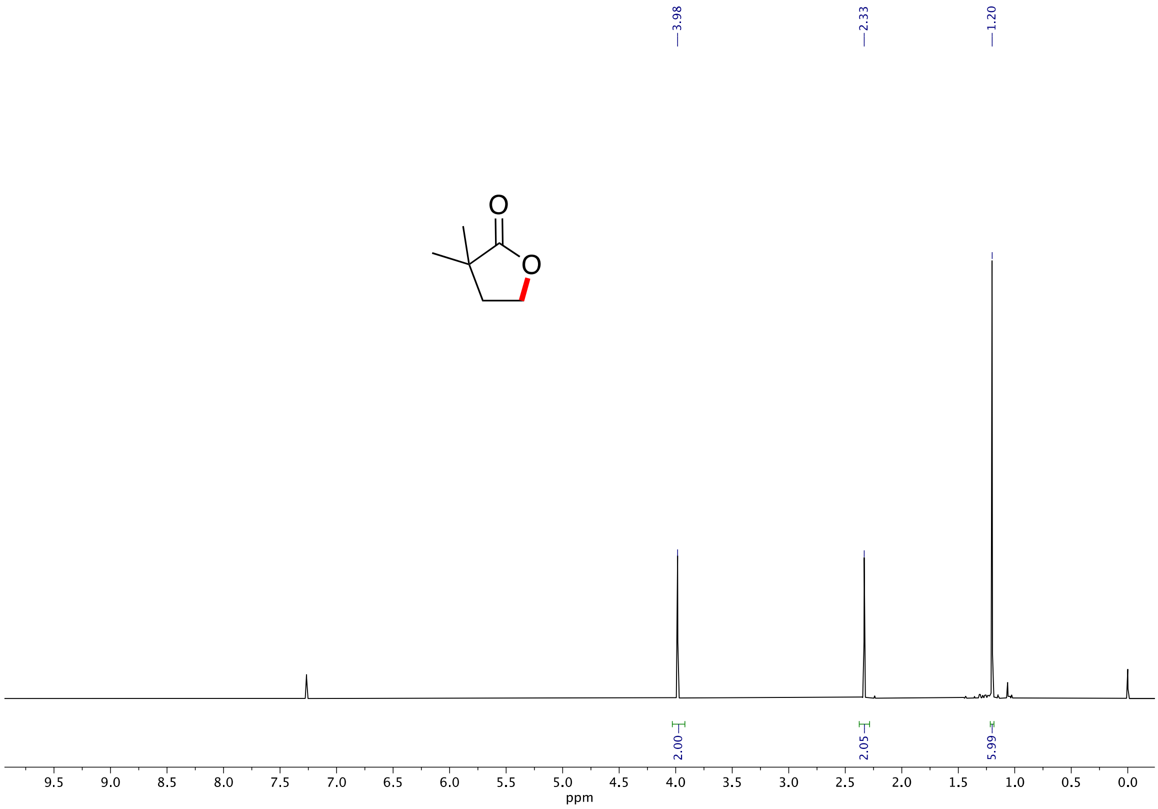

^{13}C-NMR of **5a** in $CDCl_3$

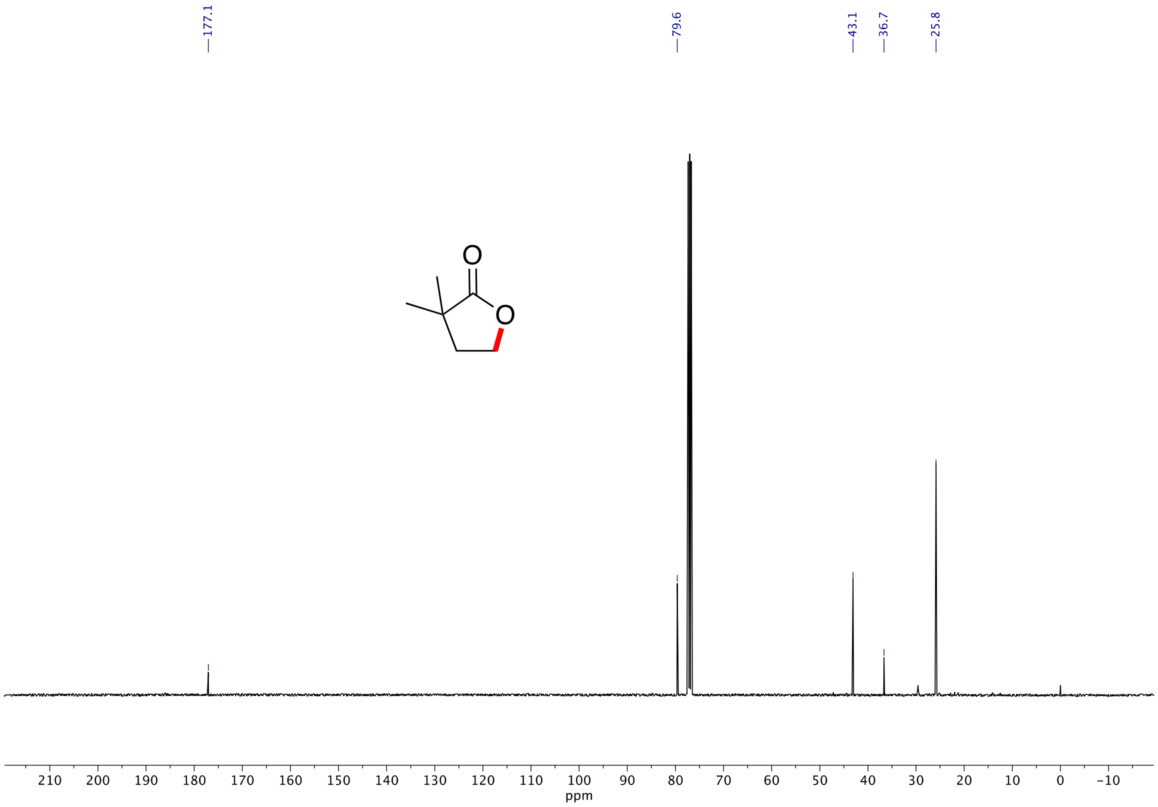

^{1}H-NMR of **6a** in $CDCl_3$

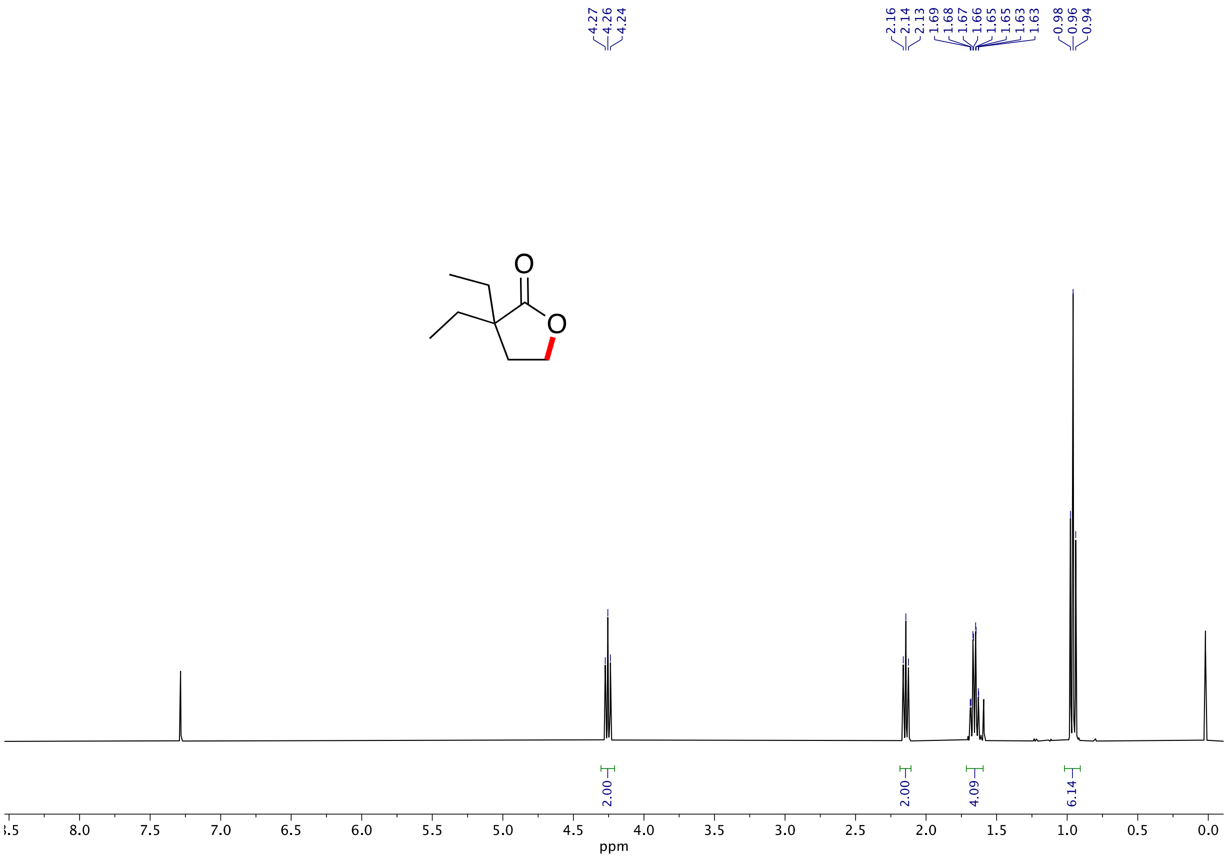

^{13}C-NMR of **6a** in $CDCl_3$

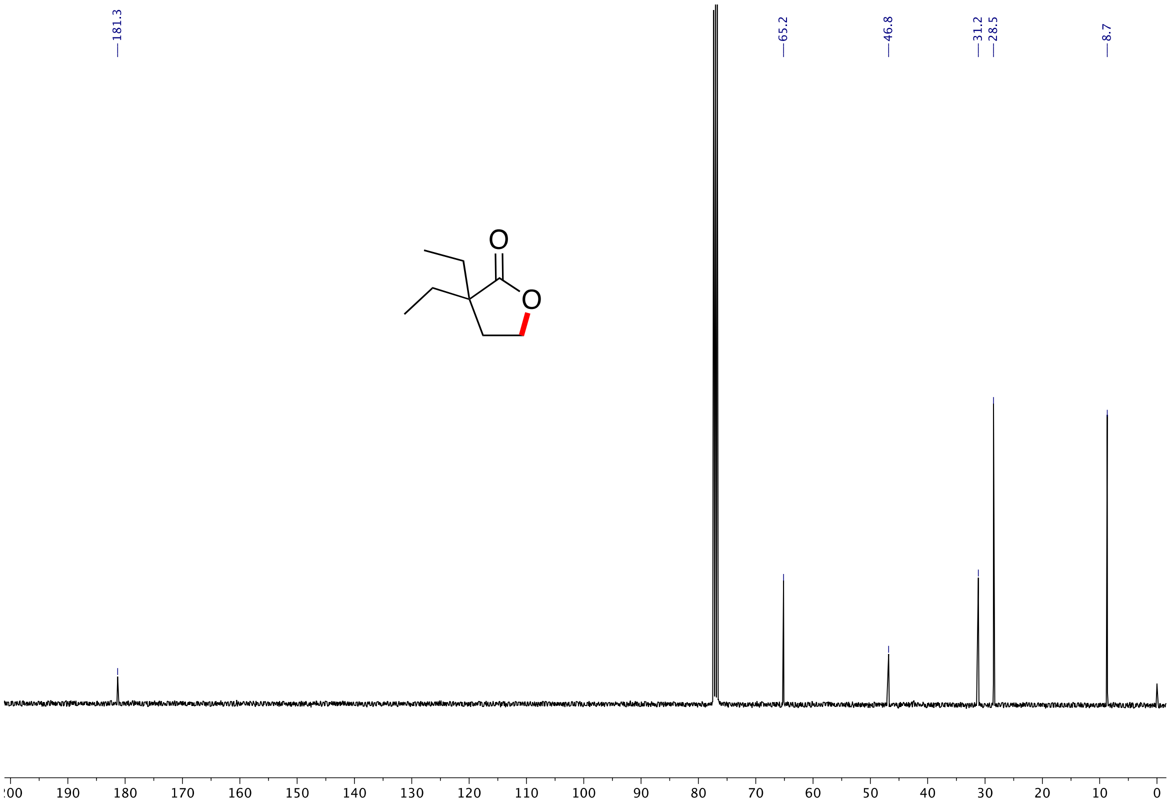

^{1}H-NMR of **7a** in $CDCl_3$

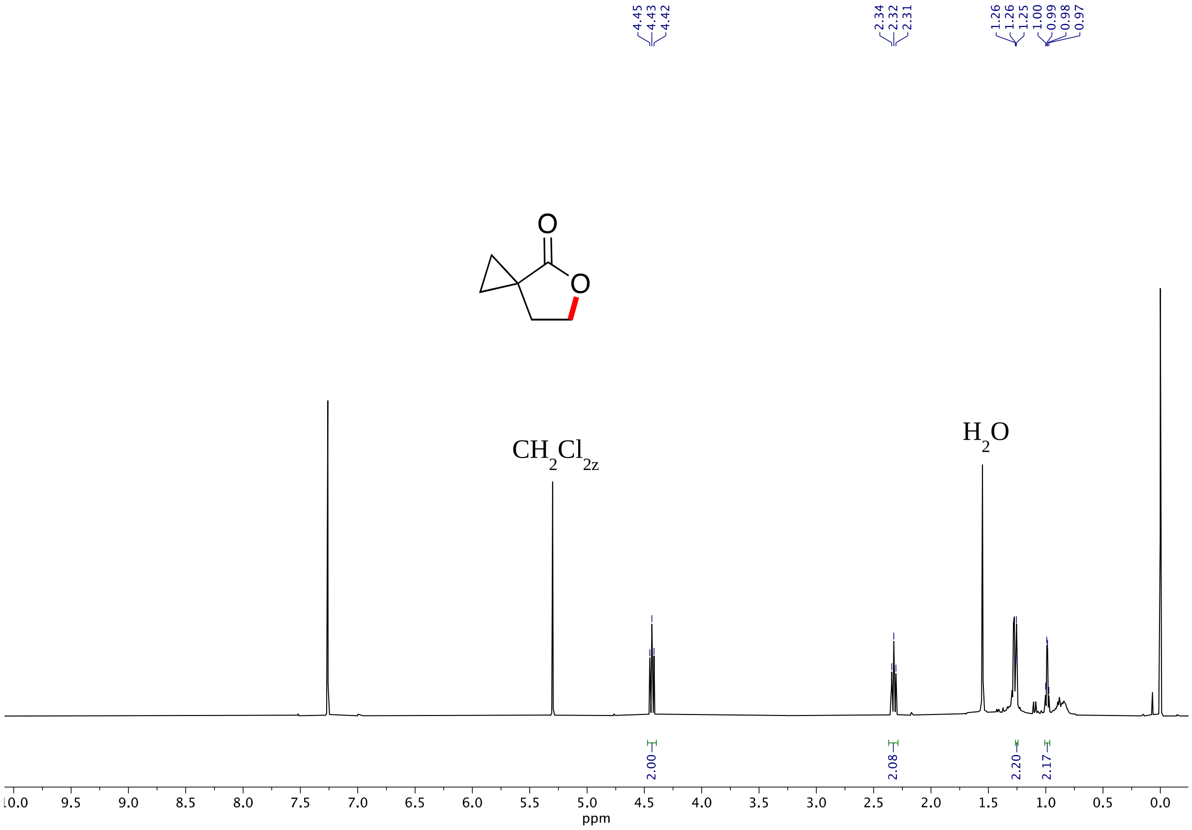

^{13}C-NMR of **7a** in $CDCl_3$

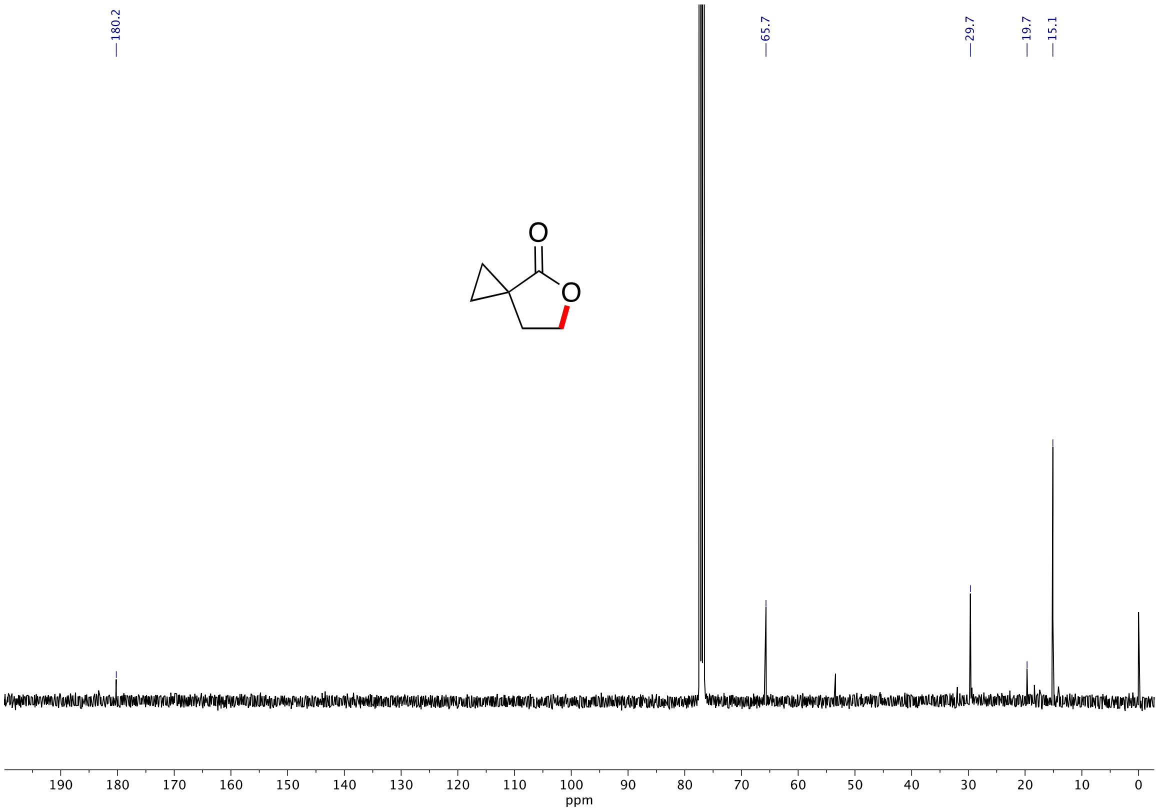

^{1}H-NMR of **8a** in $CDCl_3$

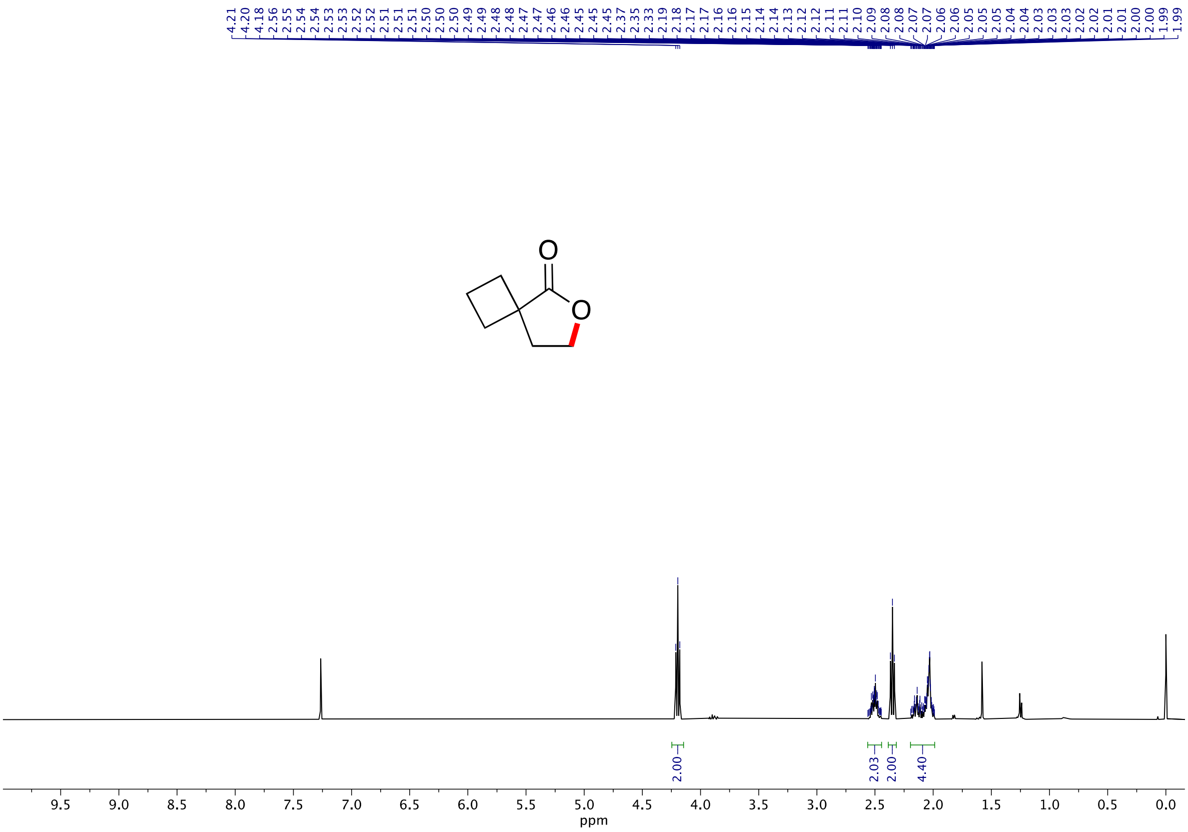

^{13}C-NMR of **8a** in $CDCl_3$

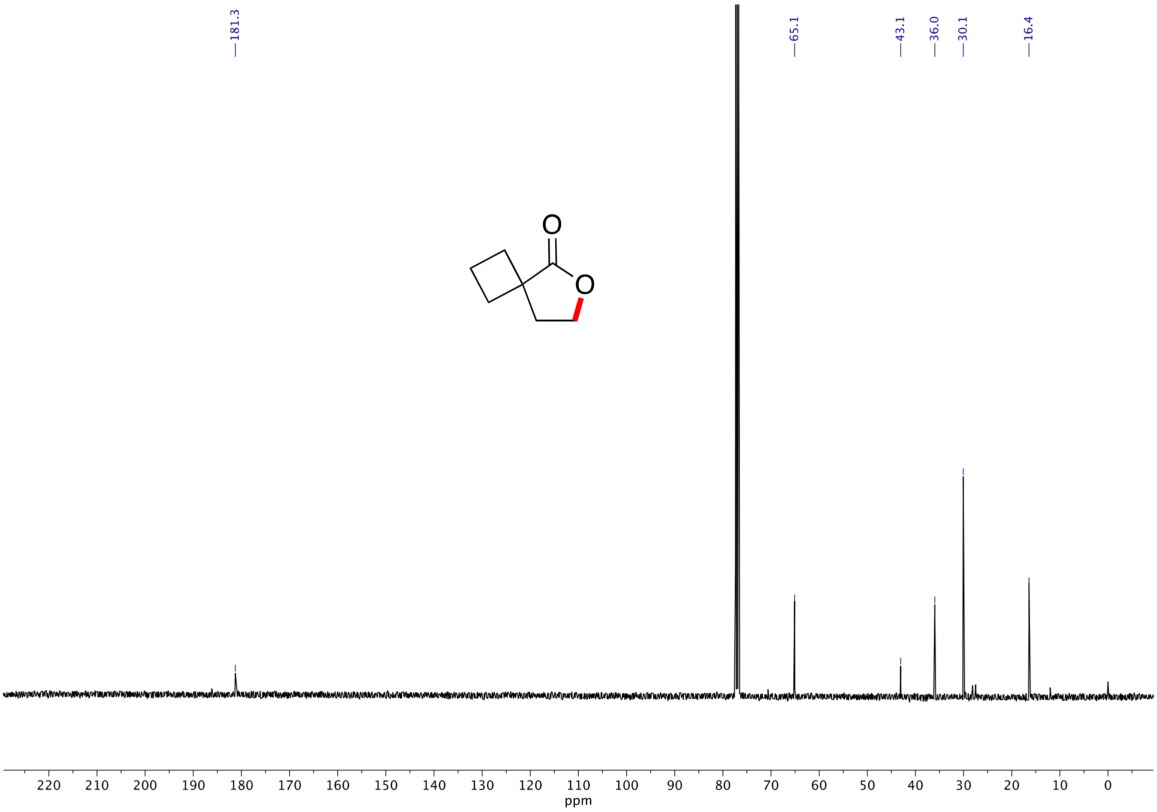

^{1}H-NMR of **9a+9b** in $CDCl_3$

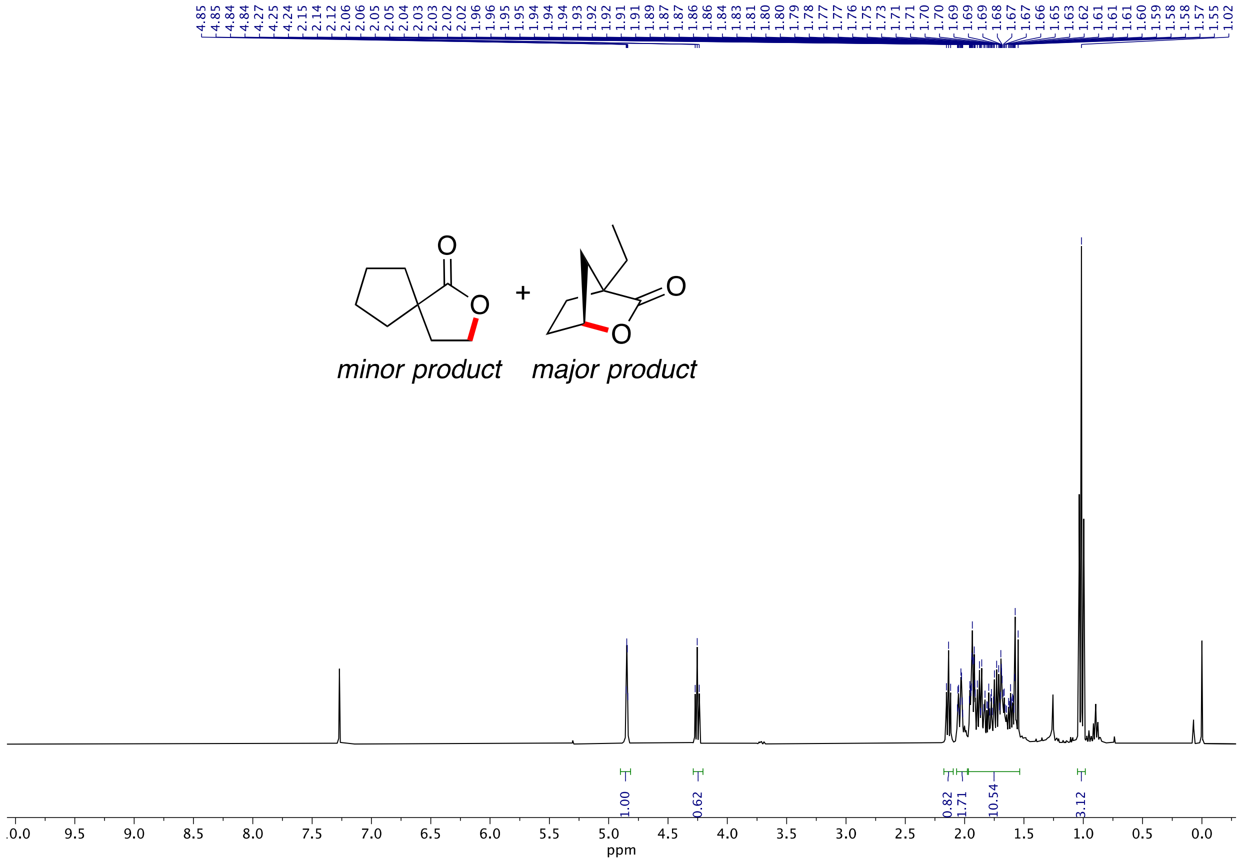

^{13}C-NMR of **9a+9b** in $CDCl_3$ (signal of **9a** labeled)

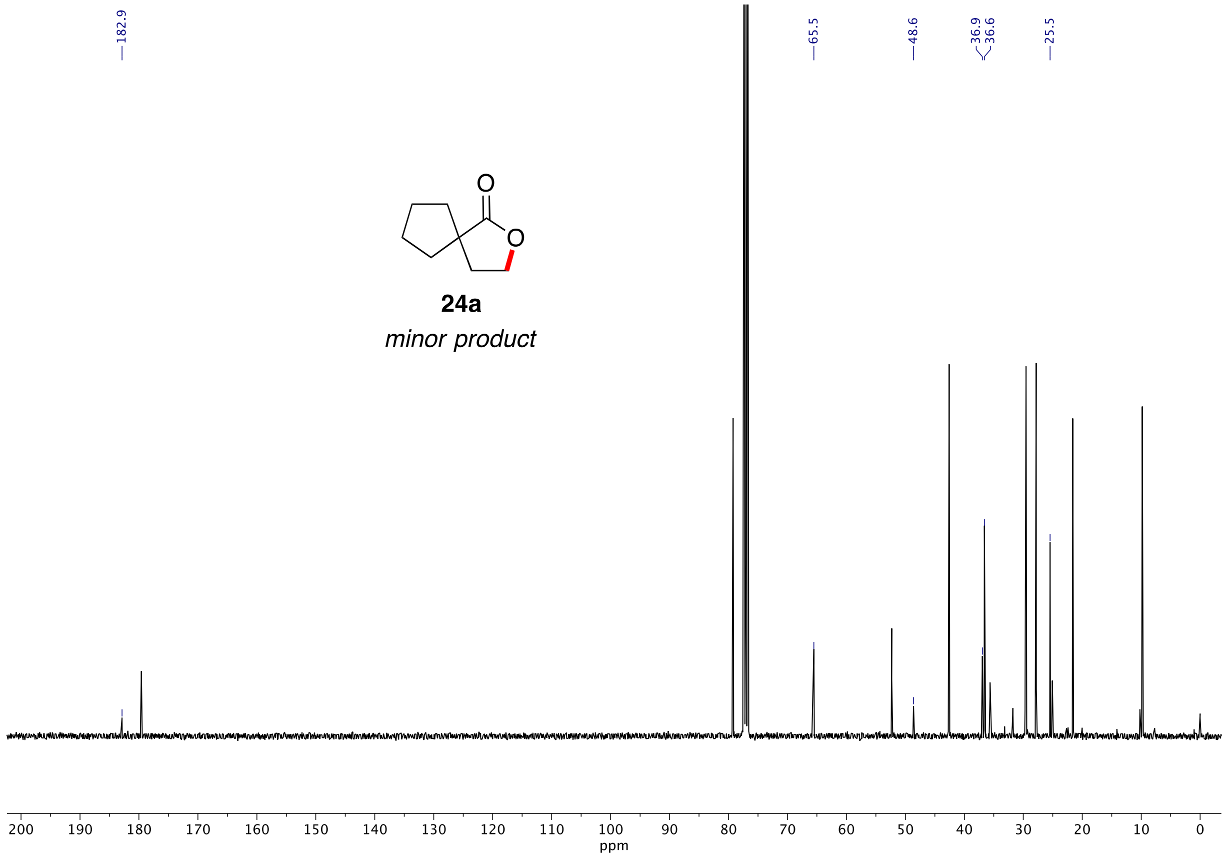

^{13}C-NMR of **9a+9b** in $CDCl_3$ (signal of **9b** labeled)

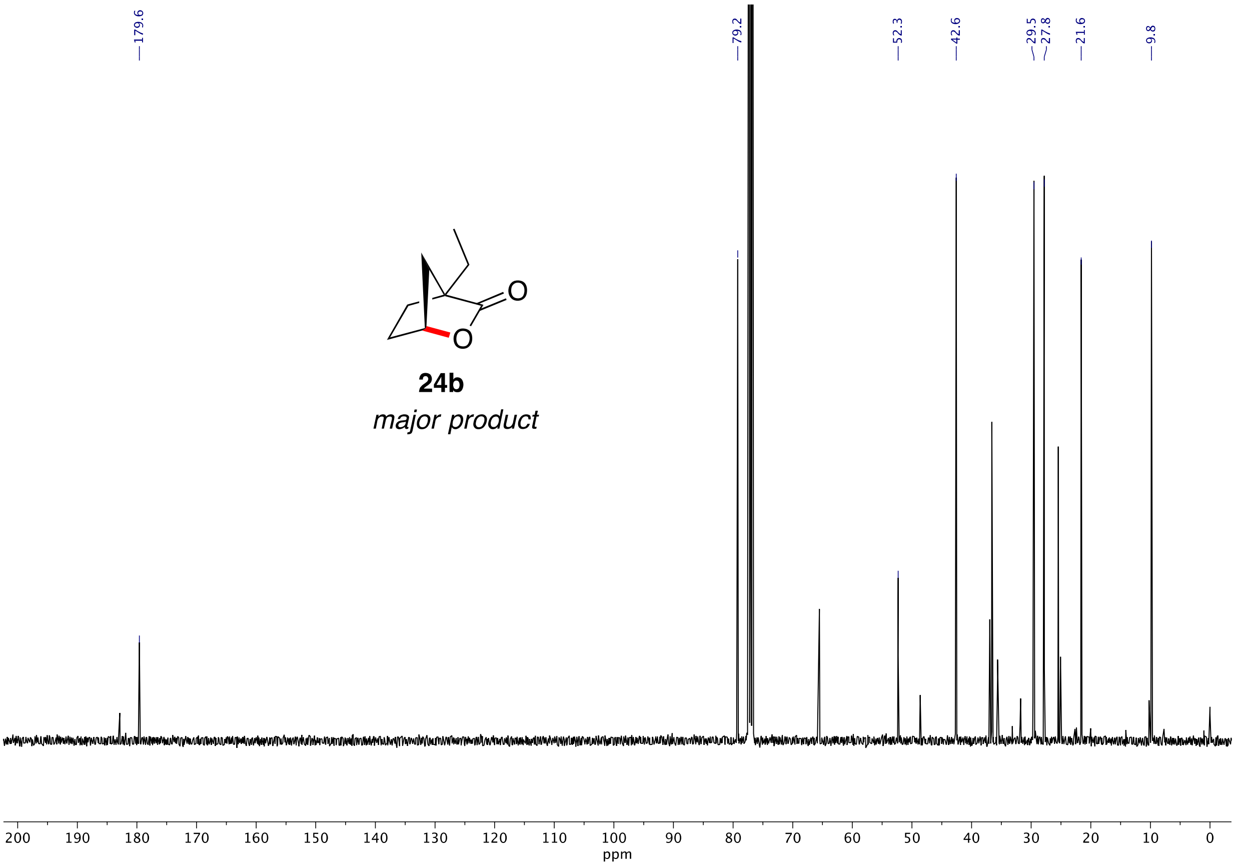

Selective TOCSY experiments of **9a+9b**

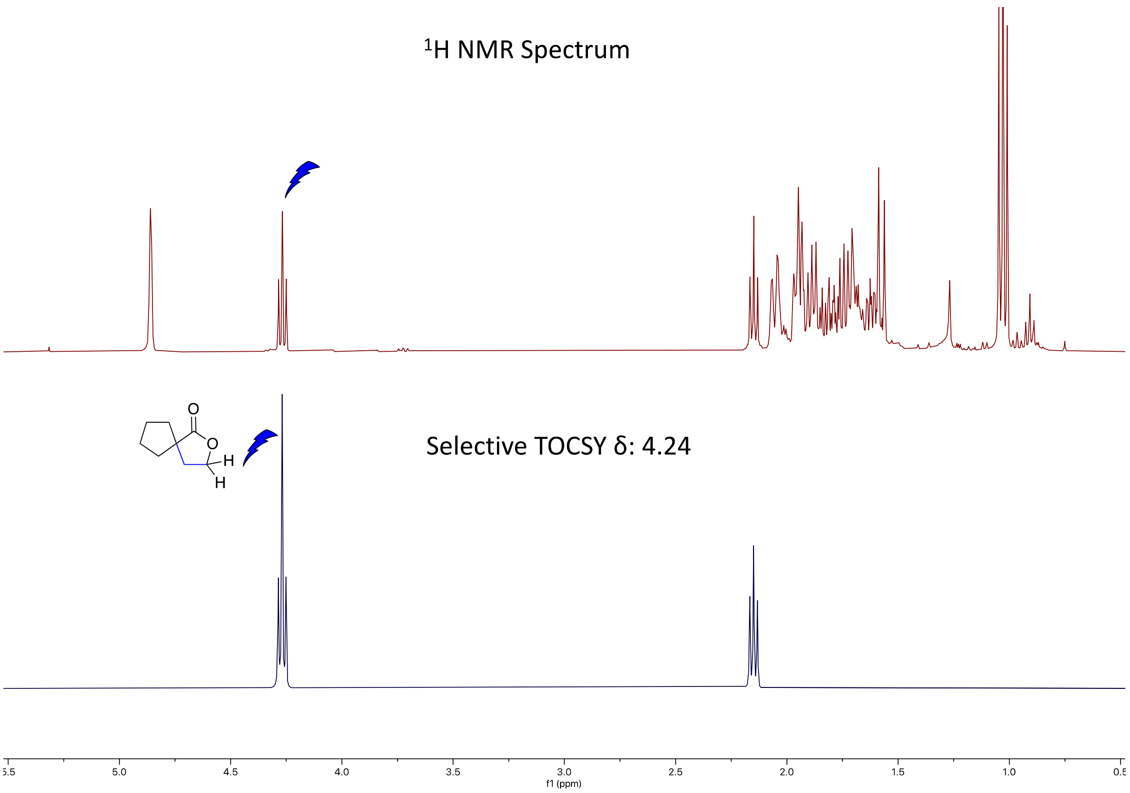

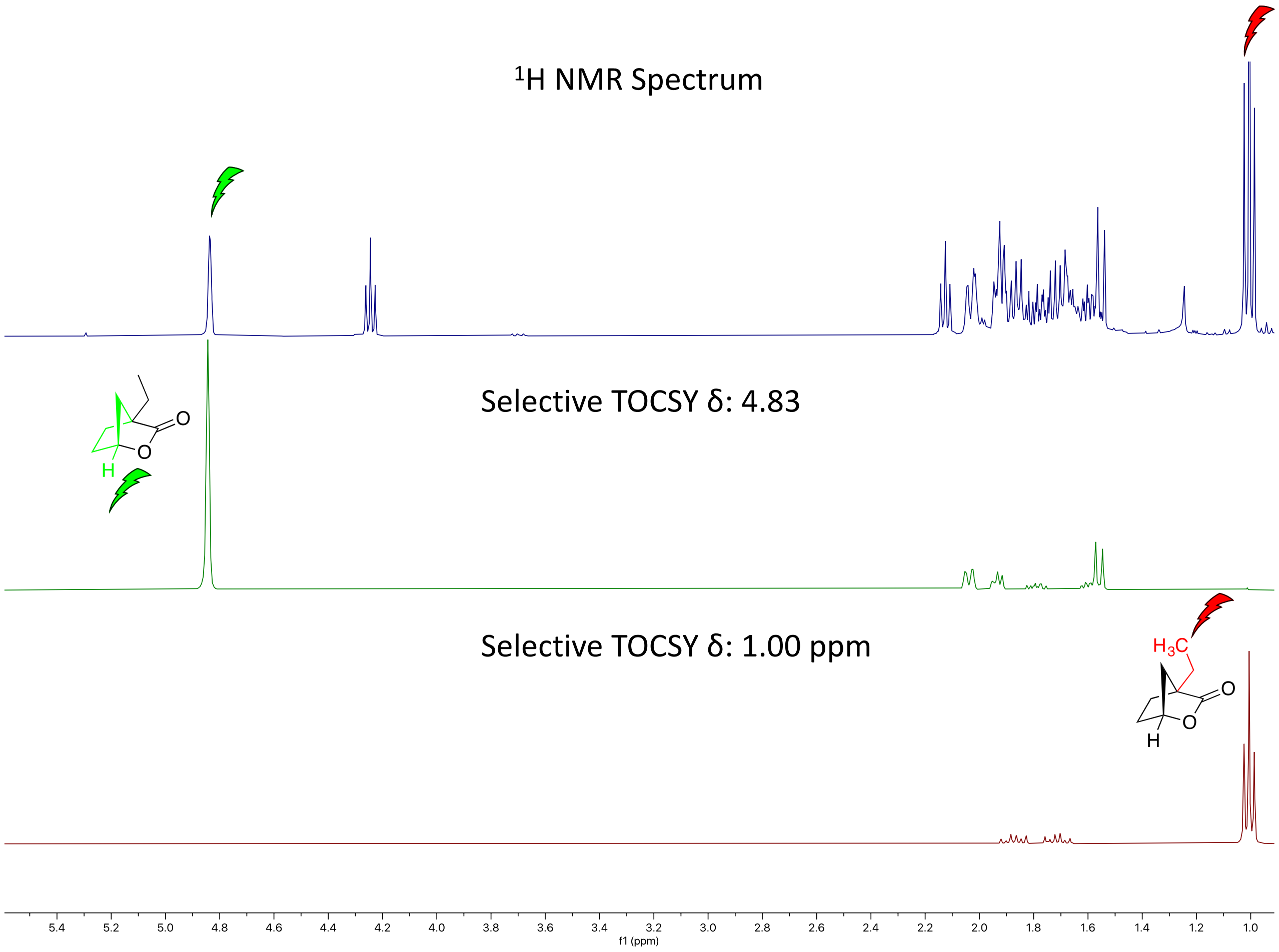

^{1}H NMR Spectrum
Selective TOCSY δ: 4.83
Selective TOCSY δ: 1.00 ppm
H
O
O
H_3C
5.4
5.2
5.0
4.8
4.6
4.4
4.2
4.0
3.8
3.6
3.4
3.2
3.0
2.8
2.6
2.4
2.2
2.0
1.8
1.6
1.4
1.2
1.0
f1 (ppm)

^{1}H-NMR of **10a+10b** in $CDCl_3$

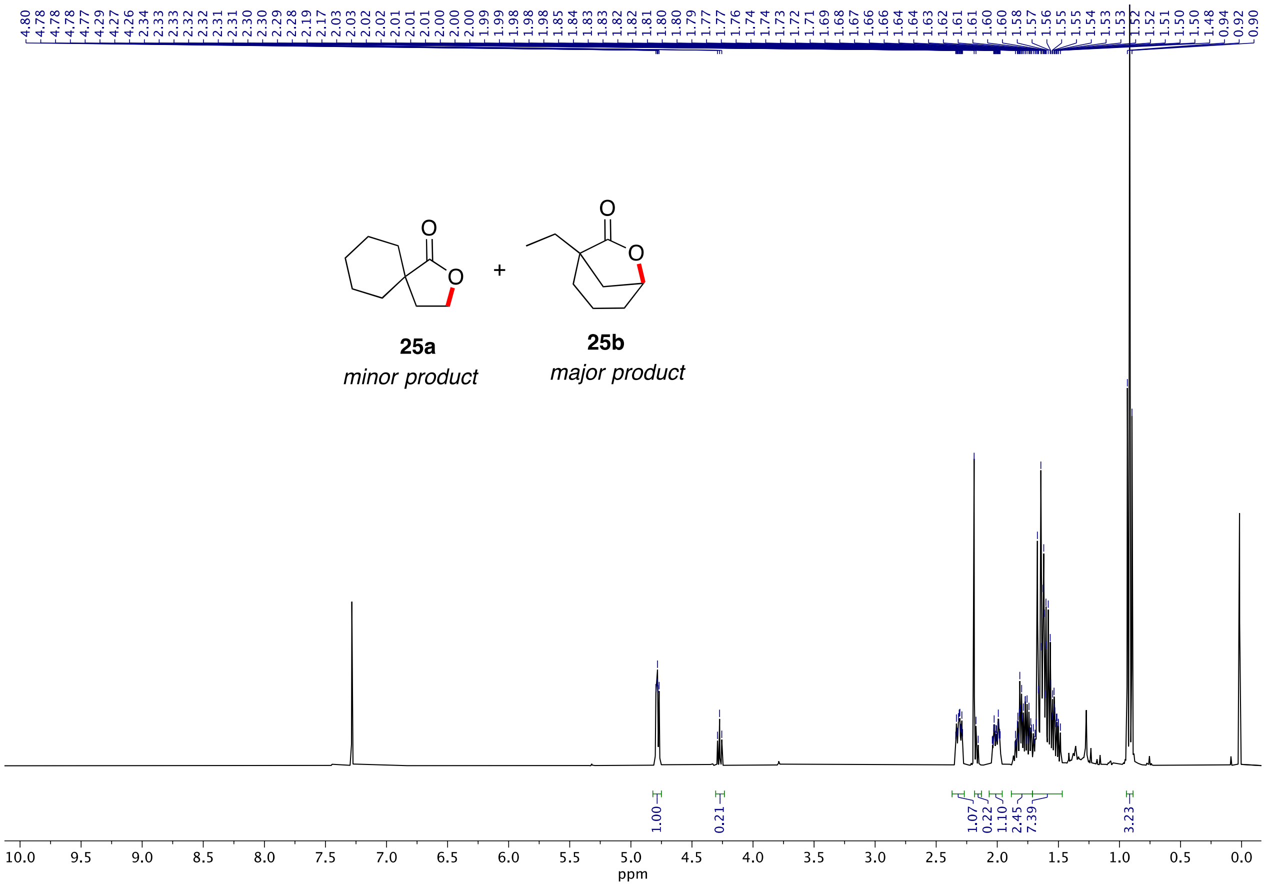

^{13}C-NMR of **10a+10b** in $CDCl_3$ (labeled **10a**)

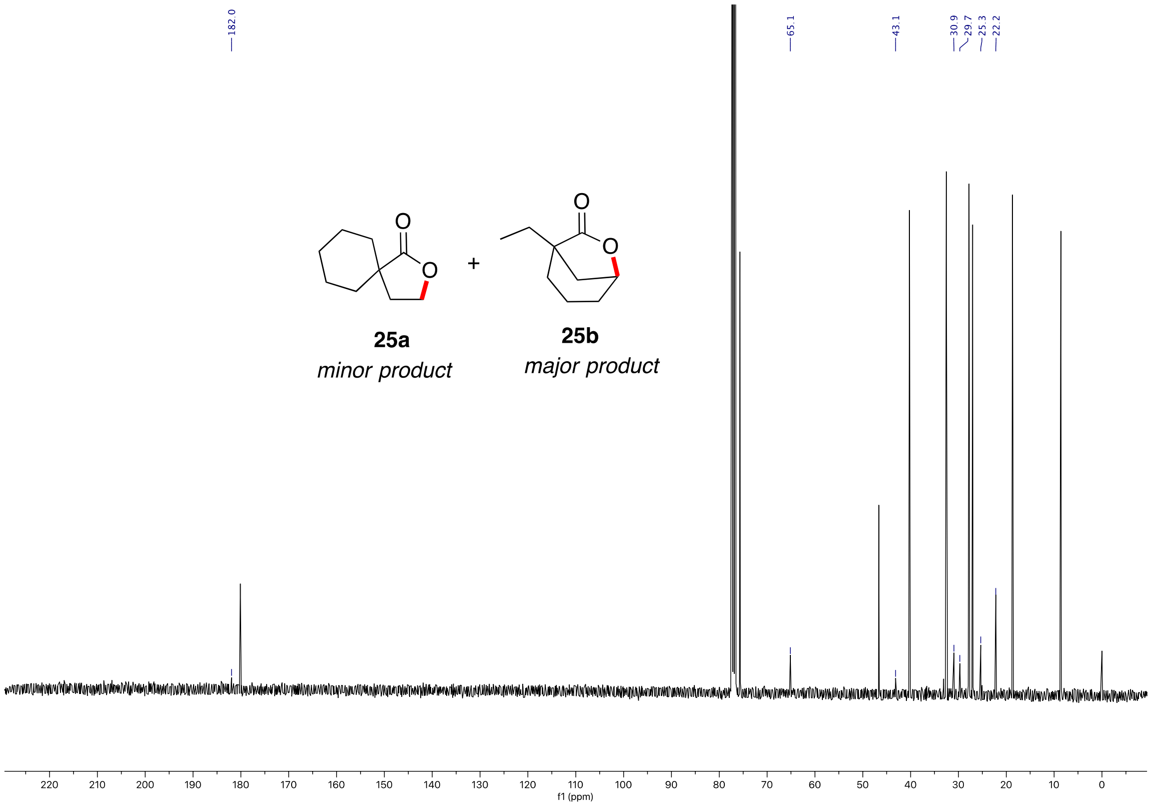

^{13}C-NMR of **10a+10b** in $CDCl_3$ (labeled **10b**)

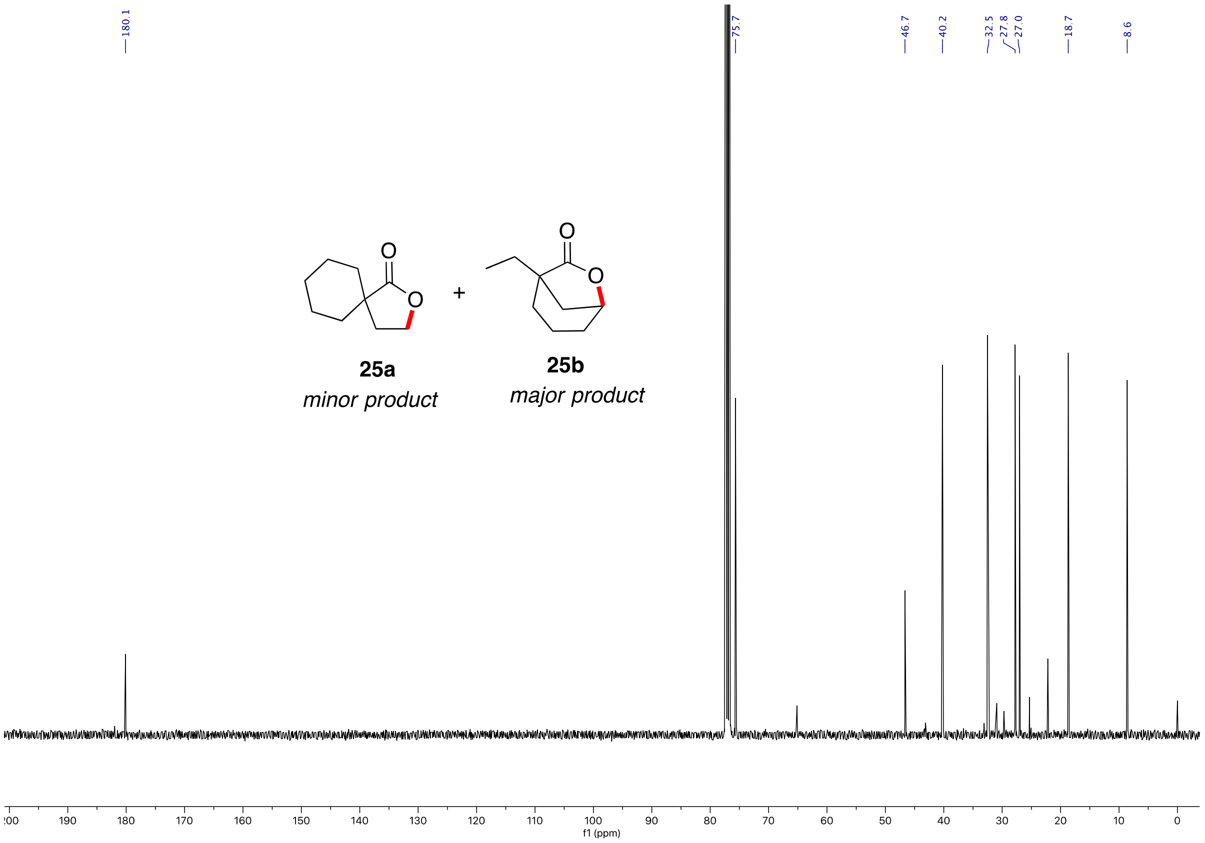

^{1}H-^{1}H TOCSY of **10a+10b** in $CDCl_3$

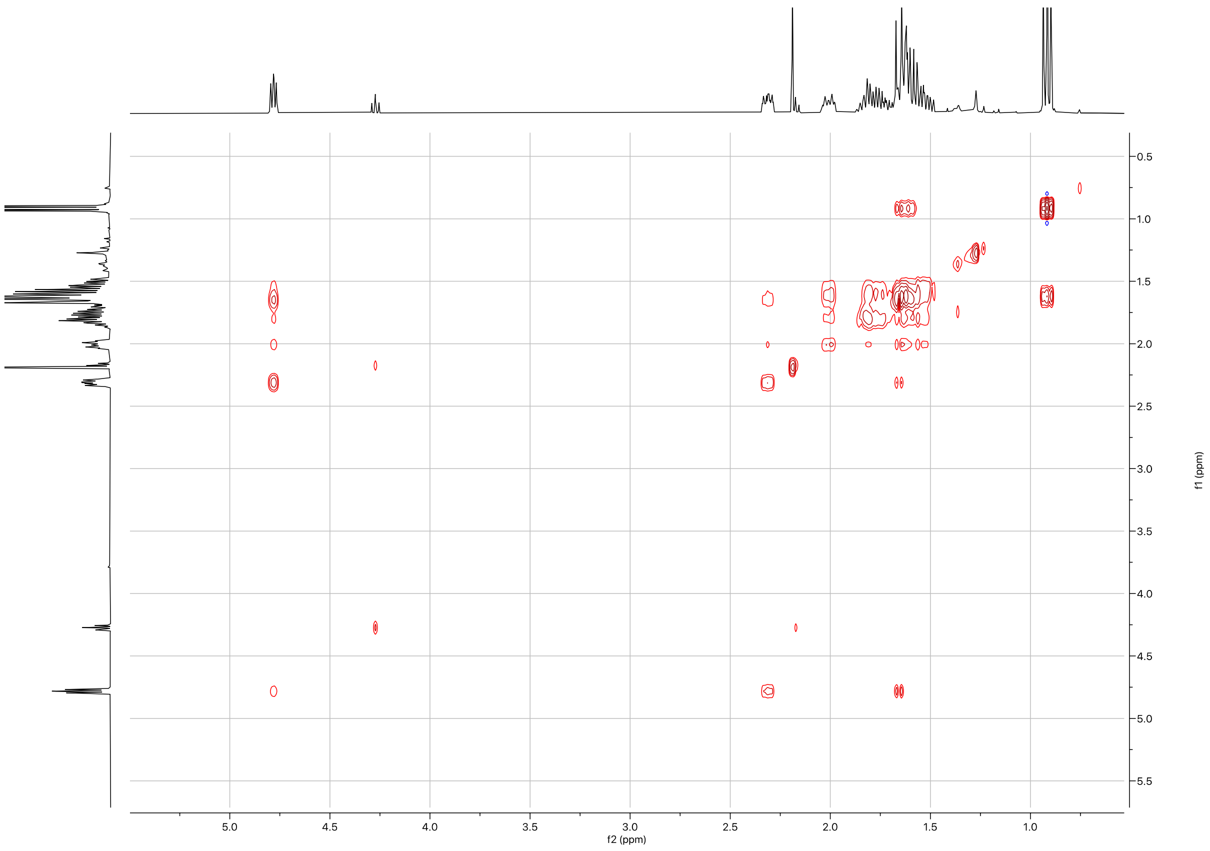

^{1}H-^{13}C HSQC of **10a+10b** in $CDCl_3$

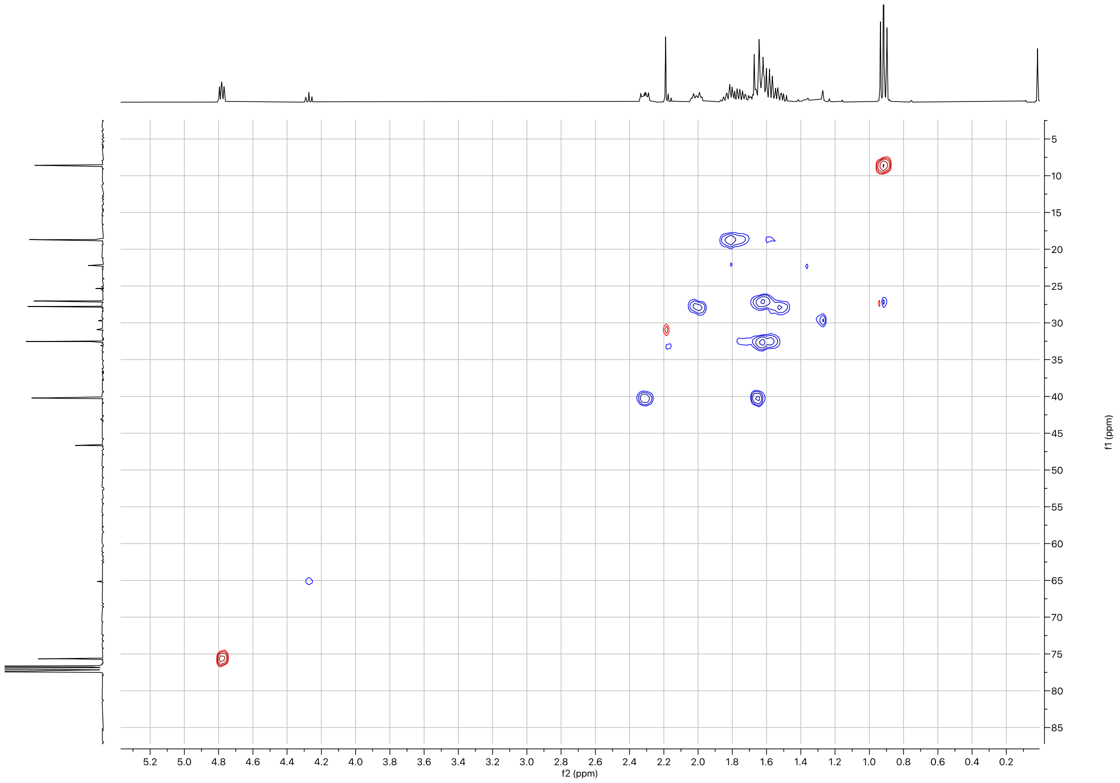

^{1}H-NMR of **11aAc** in $CDCl_3$

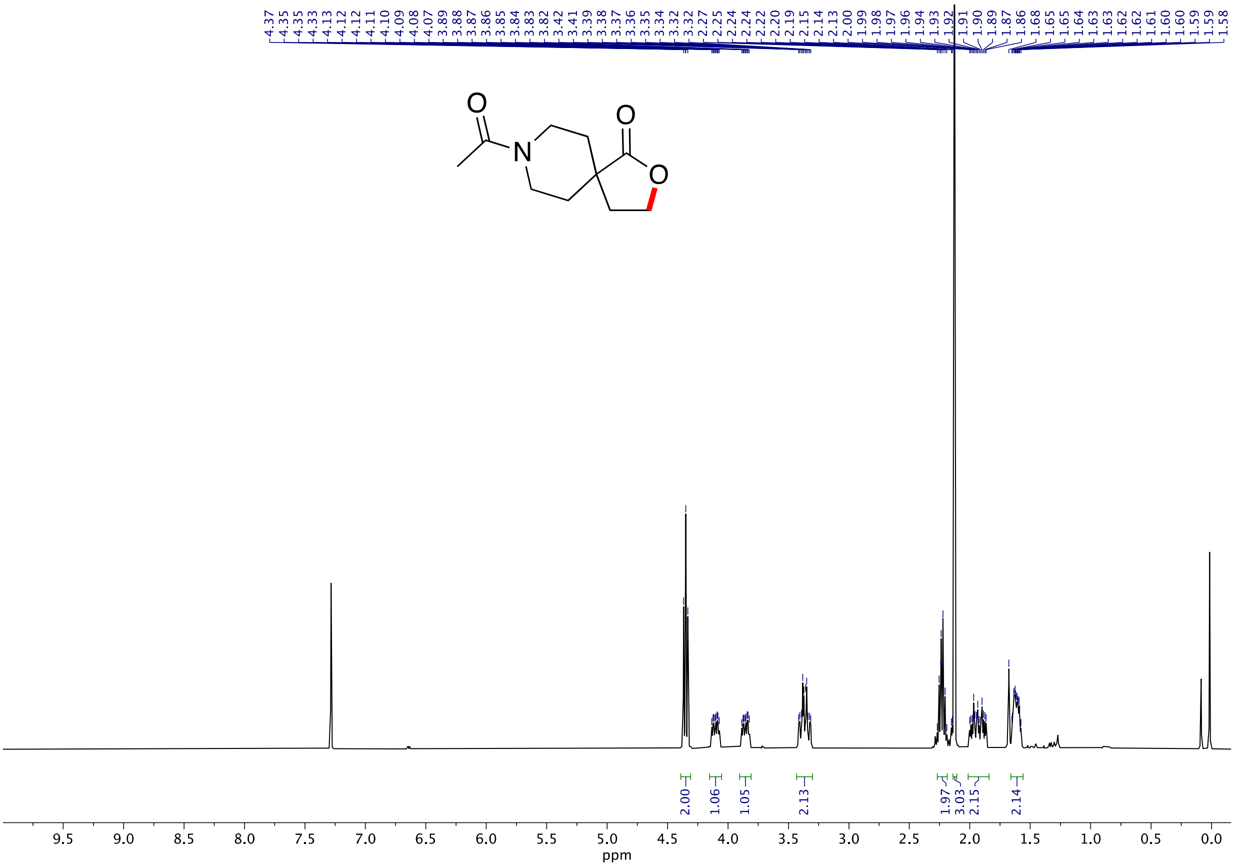

^{13}C-NMR of **11aAc** in $CDCl_3$

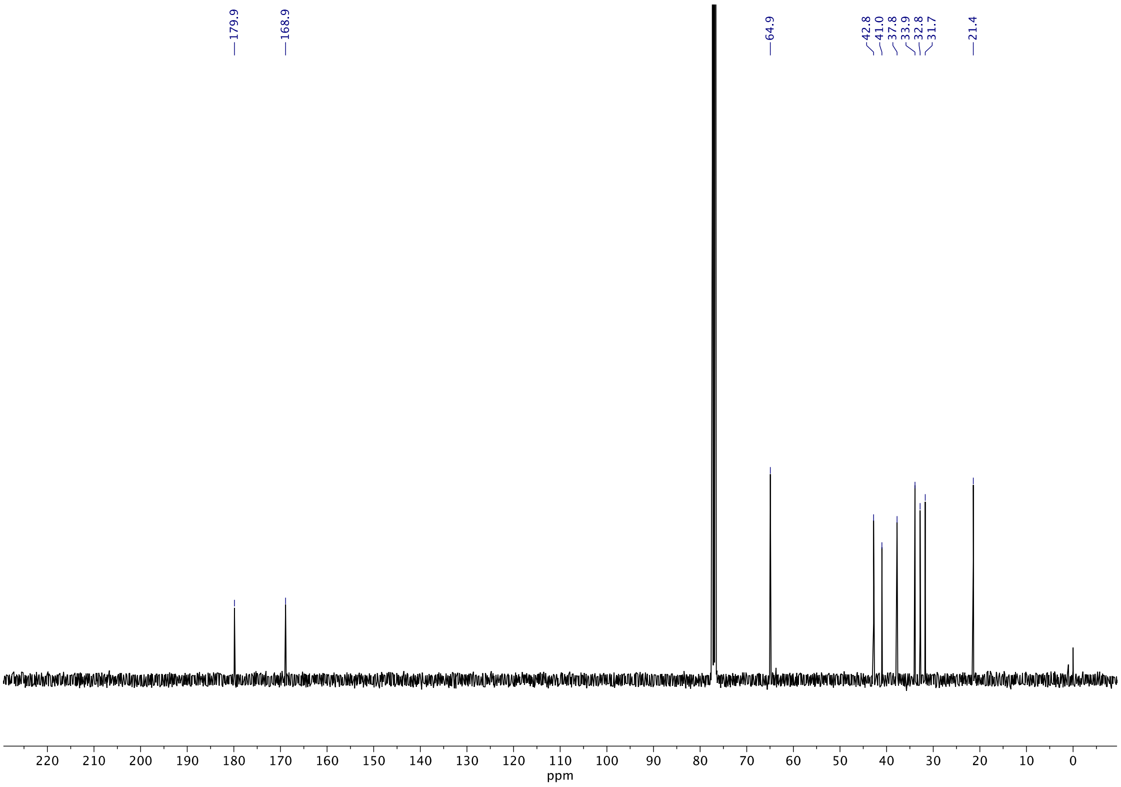

^{1}H-^{13}C HSQC of **11aAc** in $CDCl_3$

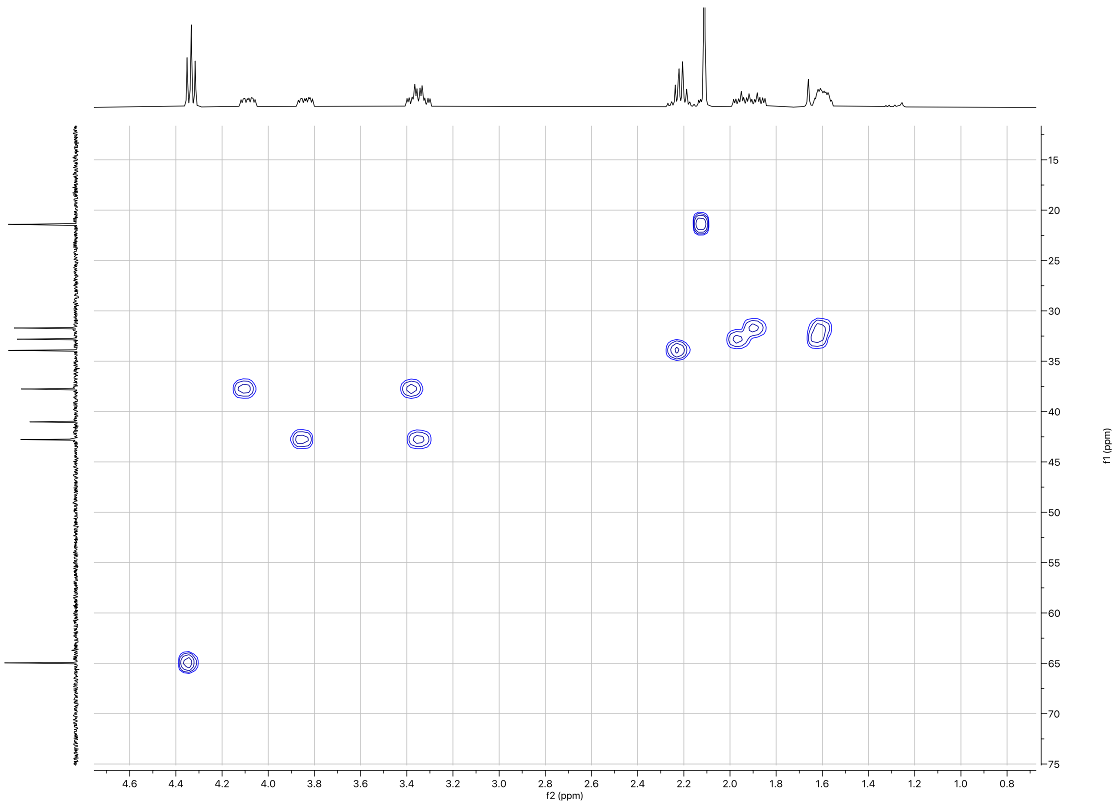

^{1}H-^{1}HCOSY of **11aAc** in $CDCl_3$

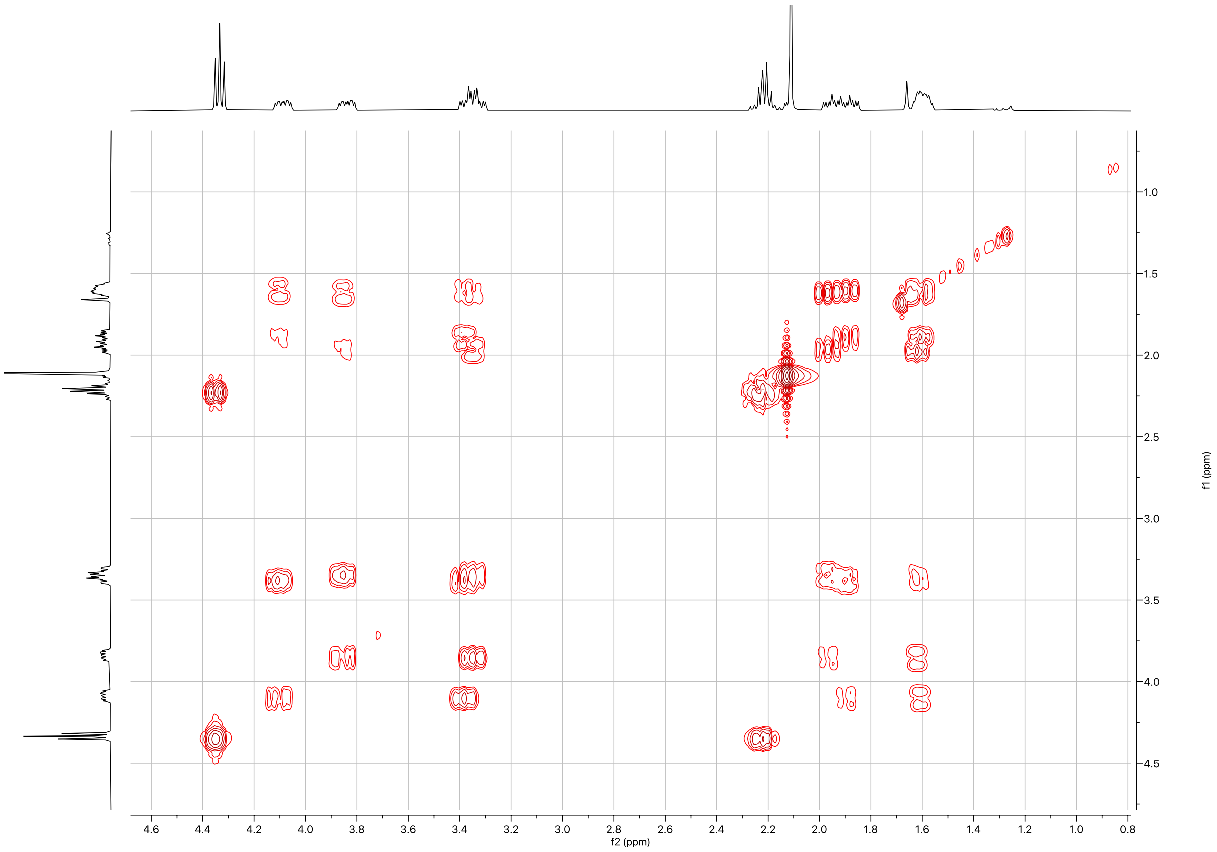

^{1}H-NMR of **12a** in $CDCl_3$

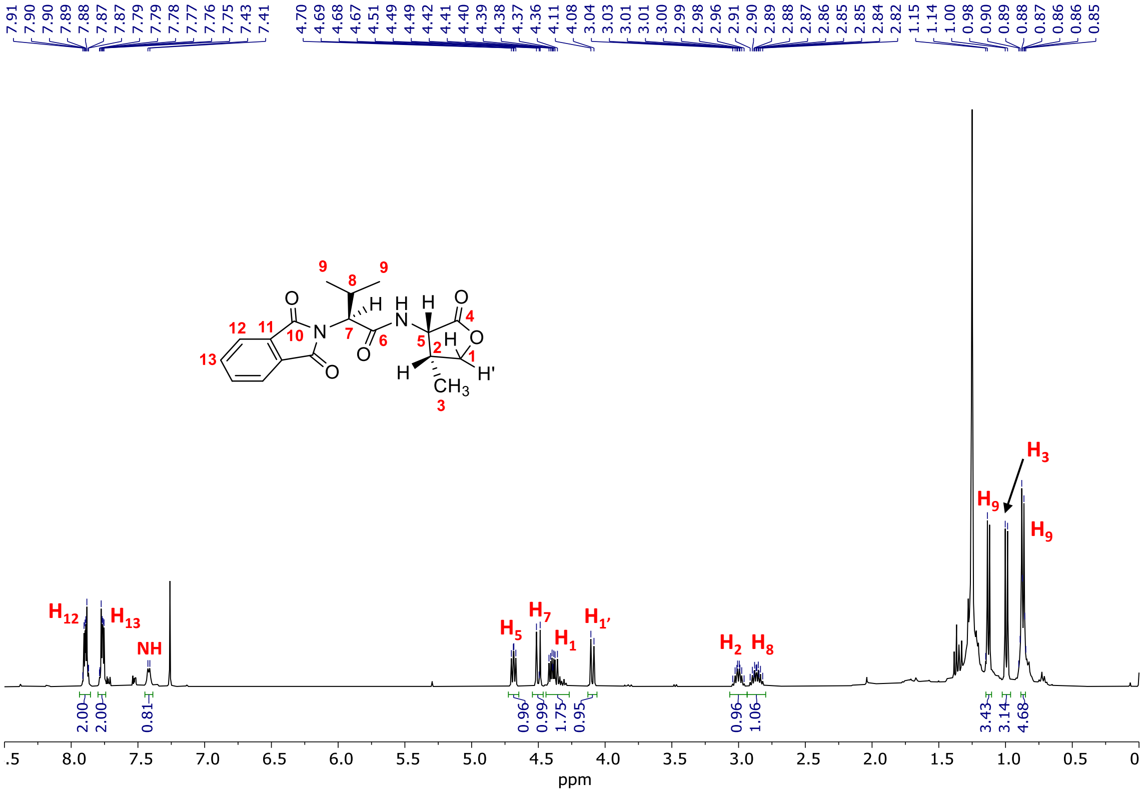

^{13}C-NMR of **12a** in $CDCl_3$

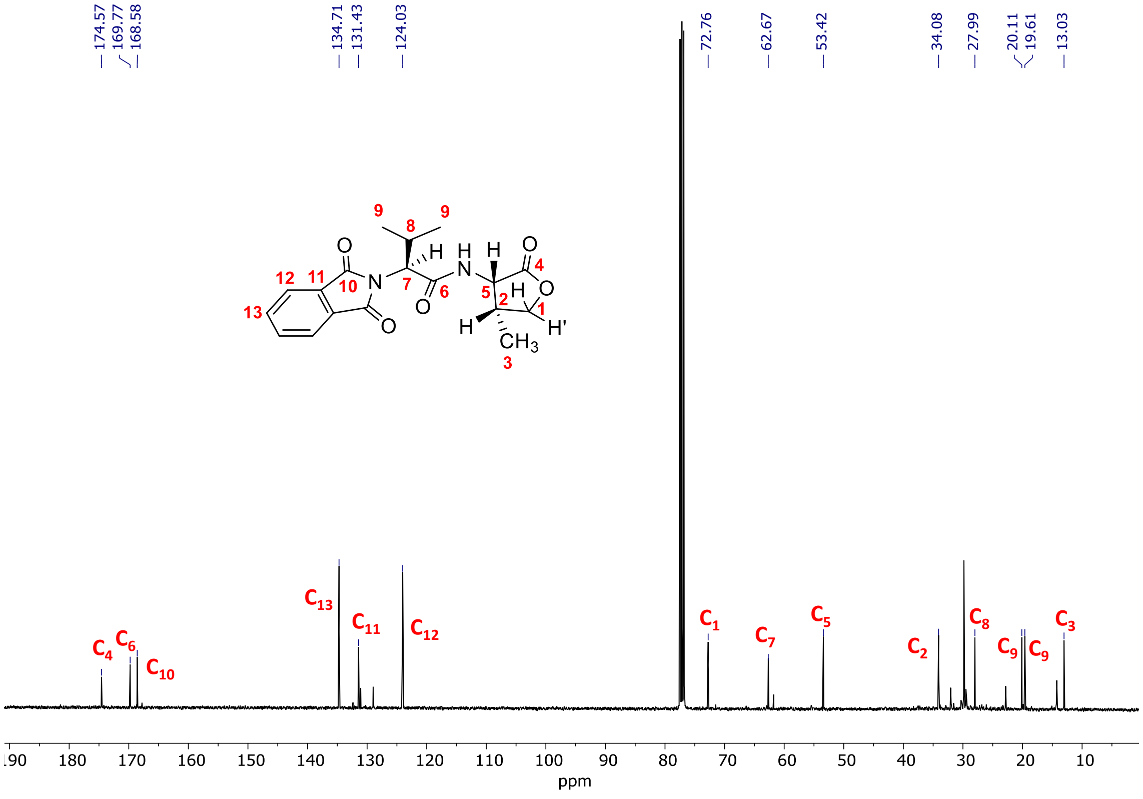

^{1}H-^{1}H COSY of **12a** in $CDCl_3$

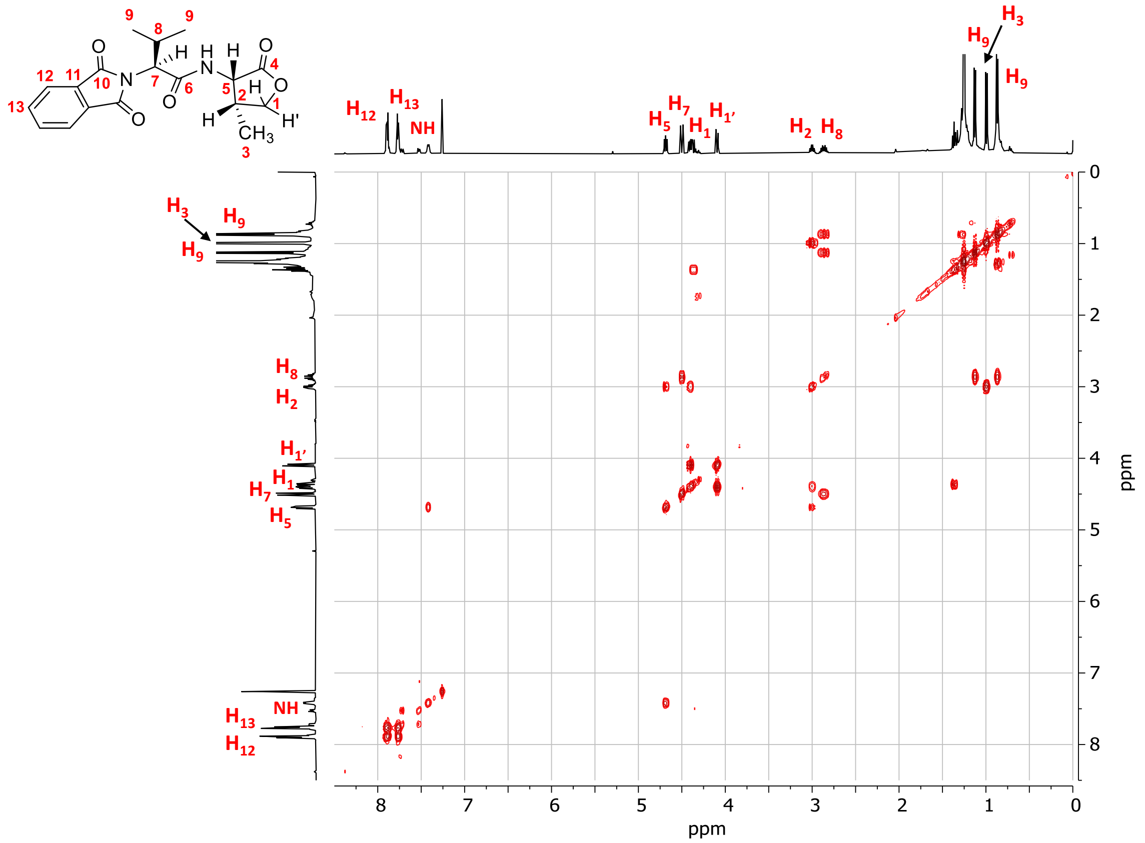

^{1}H-^{13}C HSQCed of **12a** in $CDCl_3$

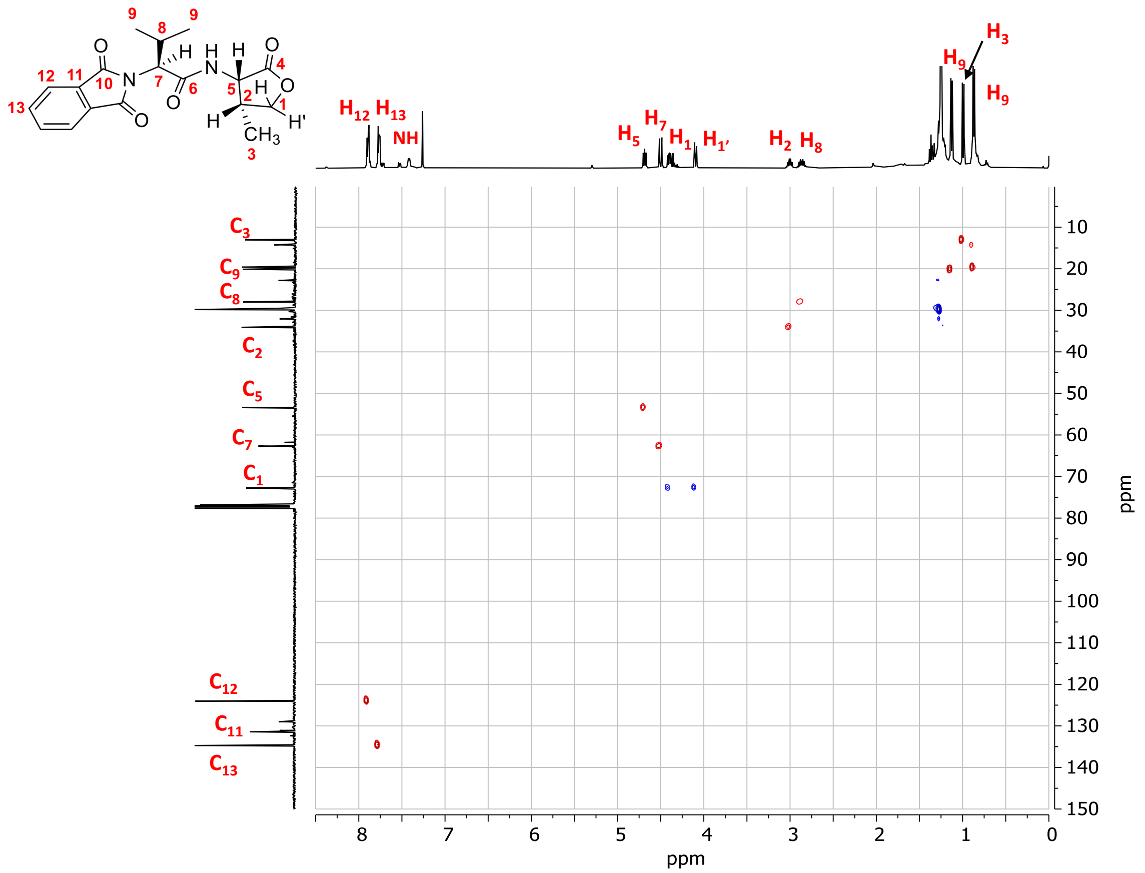

DEPTQ of **12a** in $CDCl_3$

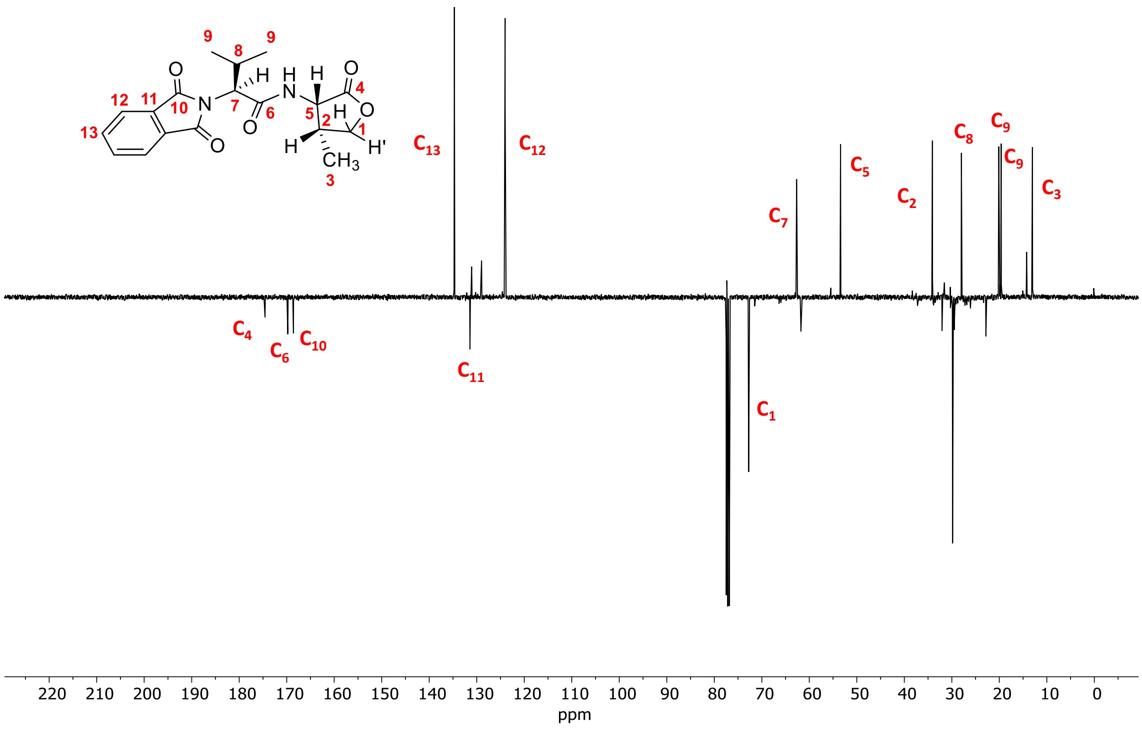

^1H-^{13}C HMBC of **12a** in $CDCl_3$

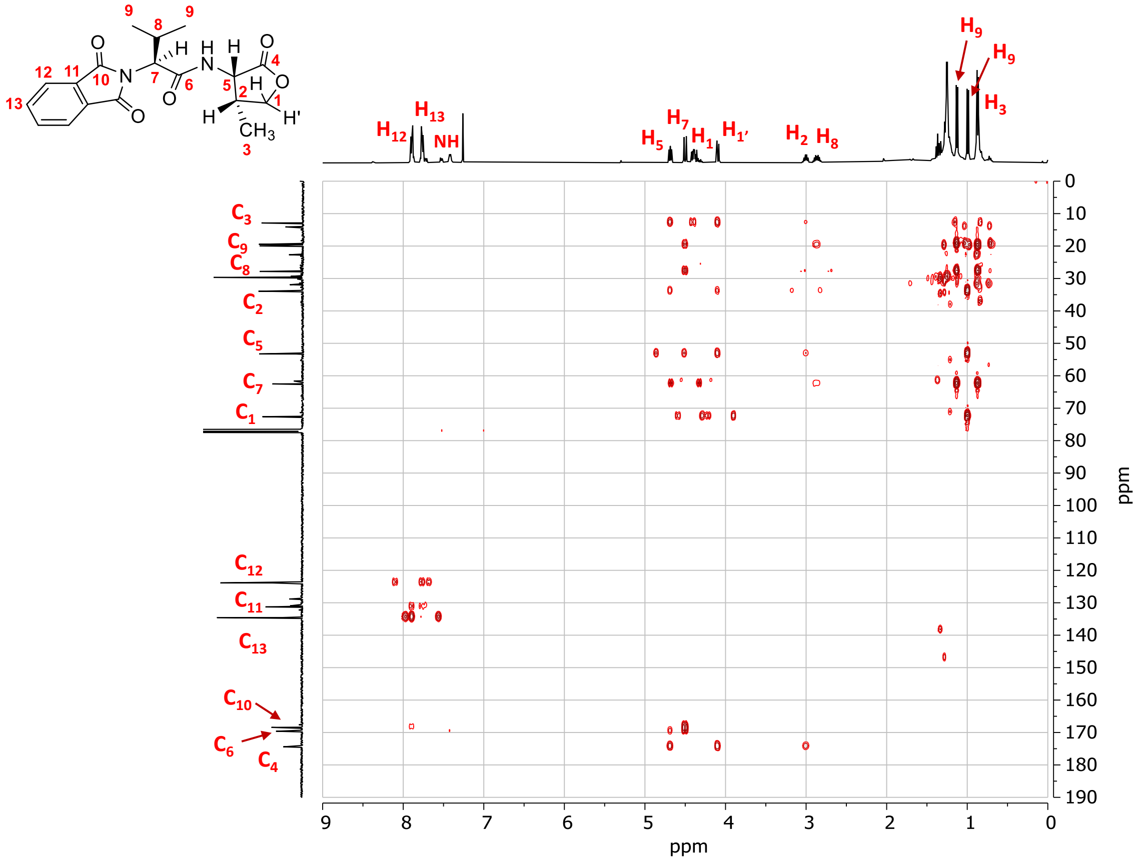

^{1}H-^{1}H TOCSY of **12a** in $CDCl_3$

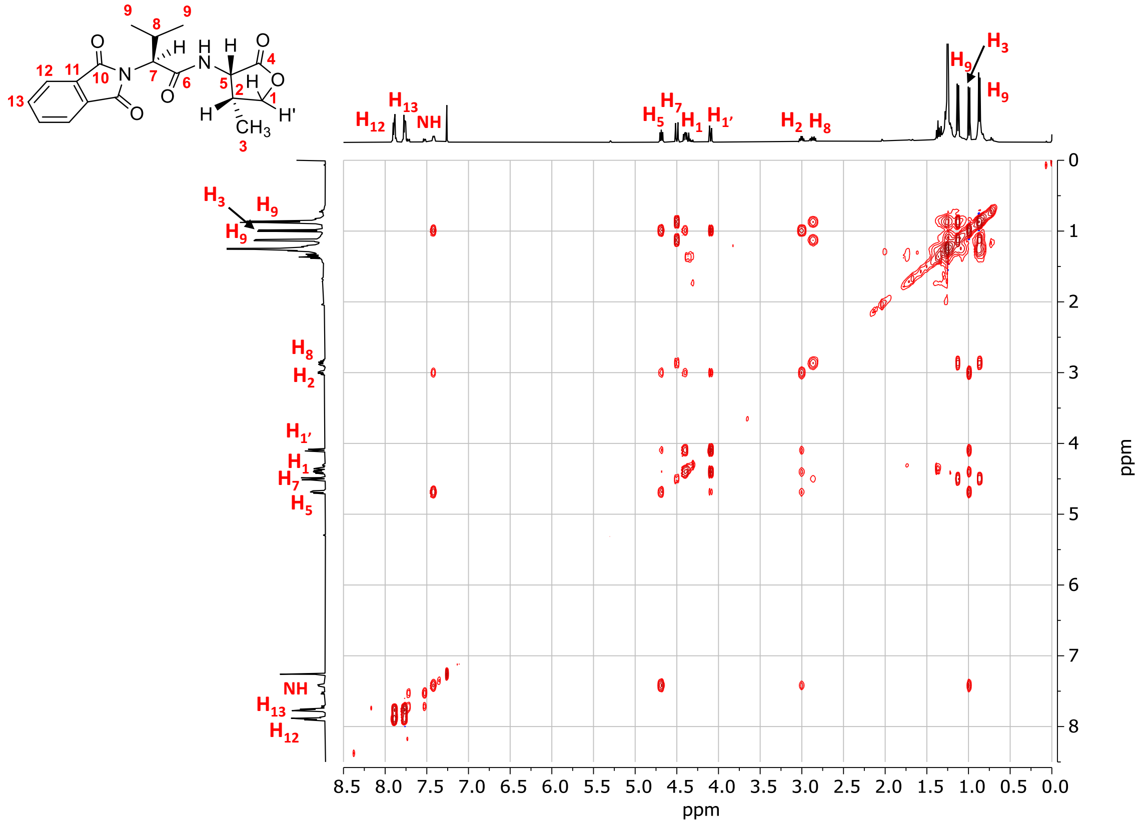

^{1}H-^{1}H NOESY of **12a** in $CDCl_3$

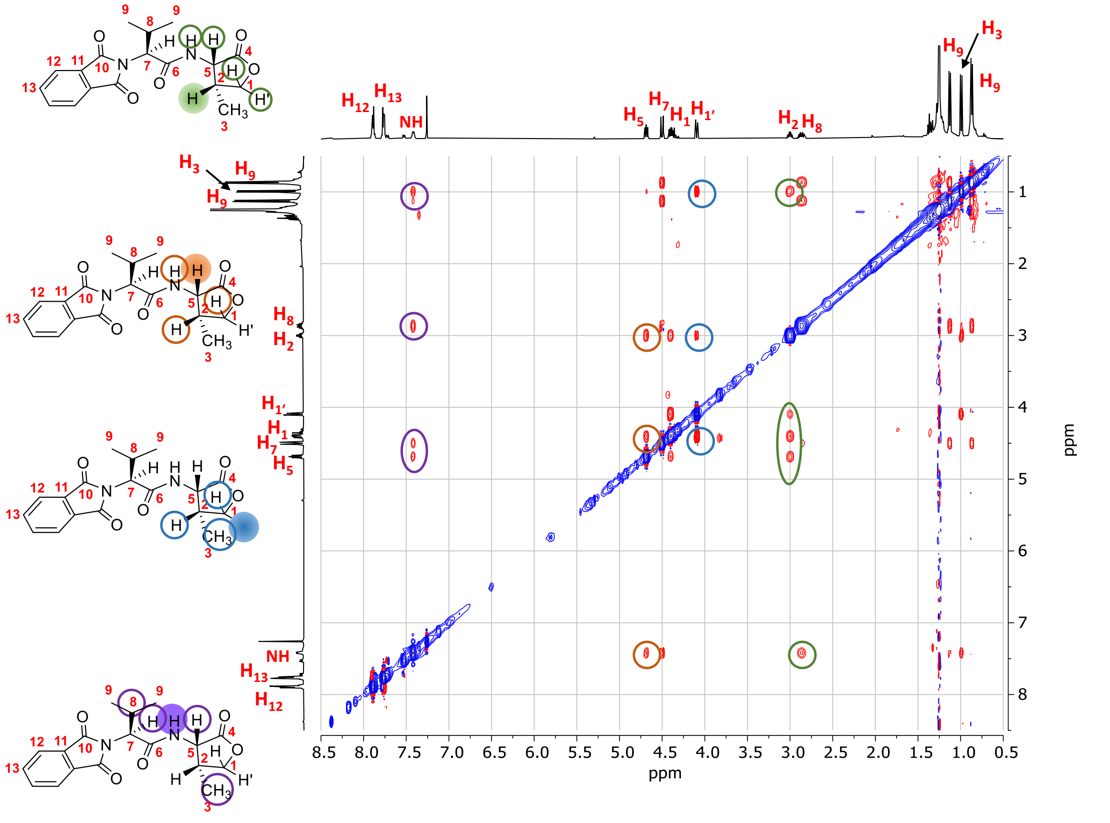

Selective NOESY experiments of **12a** in $CDCl_3$

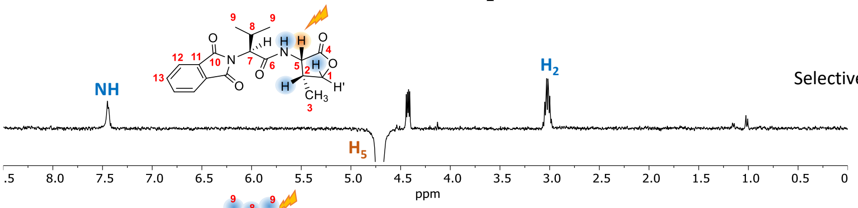

Selective NOESY δ = 4.69 ppm

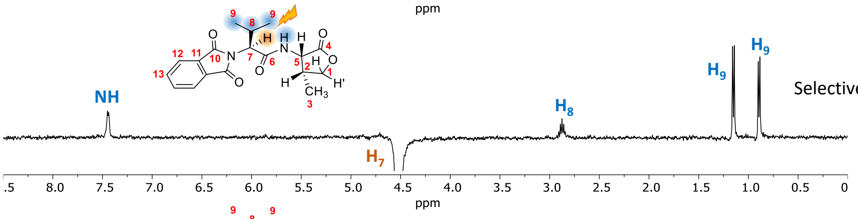

Selective NOESY δ = 4.50 ppm

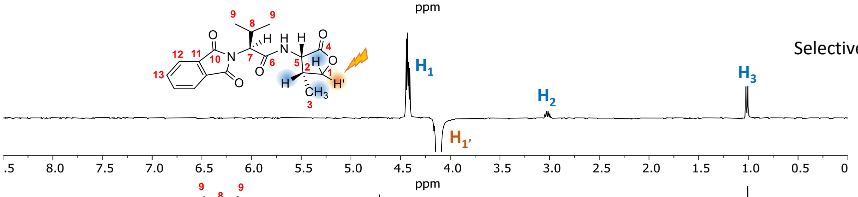

Selective NOESY δ = 4.09 ppm

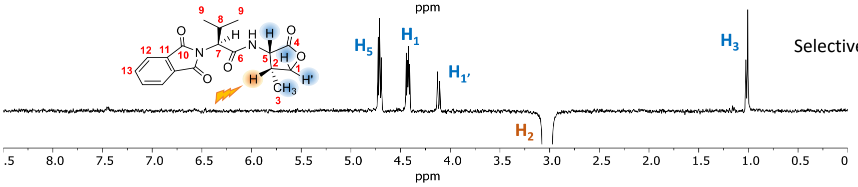

Selective NOESY δ = 3.02 ppm

^{1}H-NMR of **12b** in $CDCl_3$

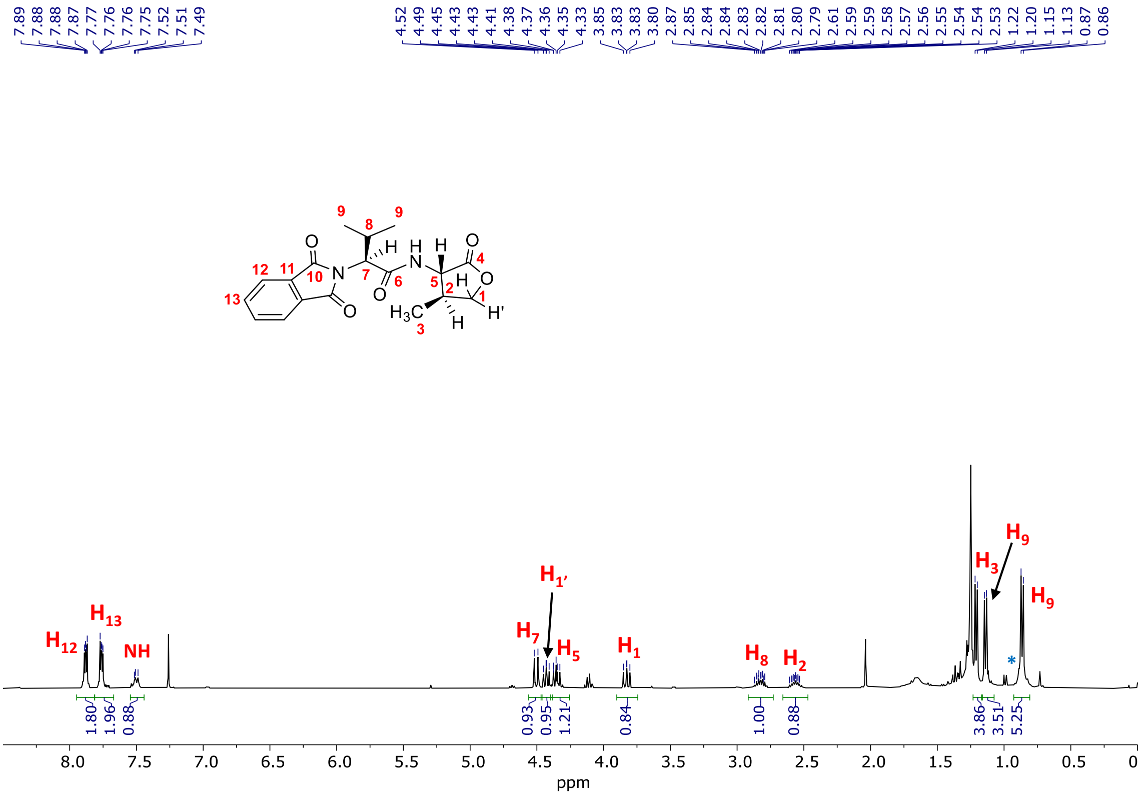

^{13}C-NMR of **12b** in $CDCl_3$

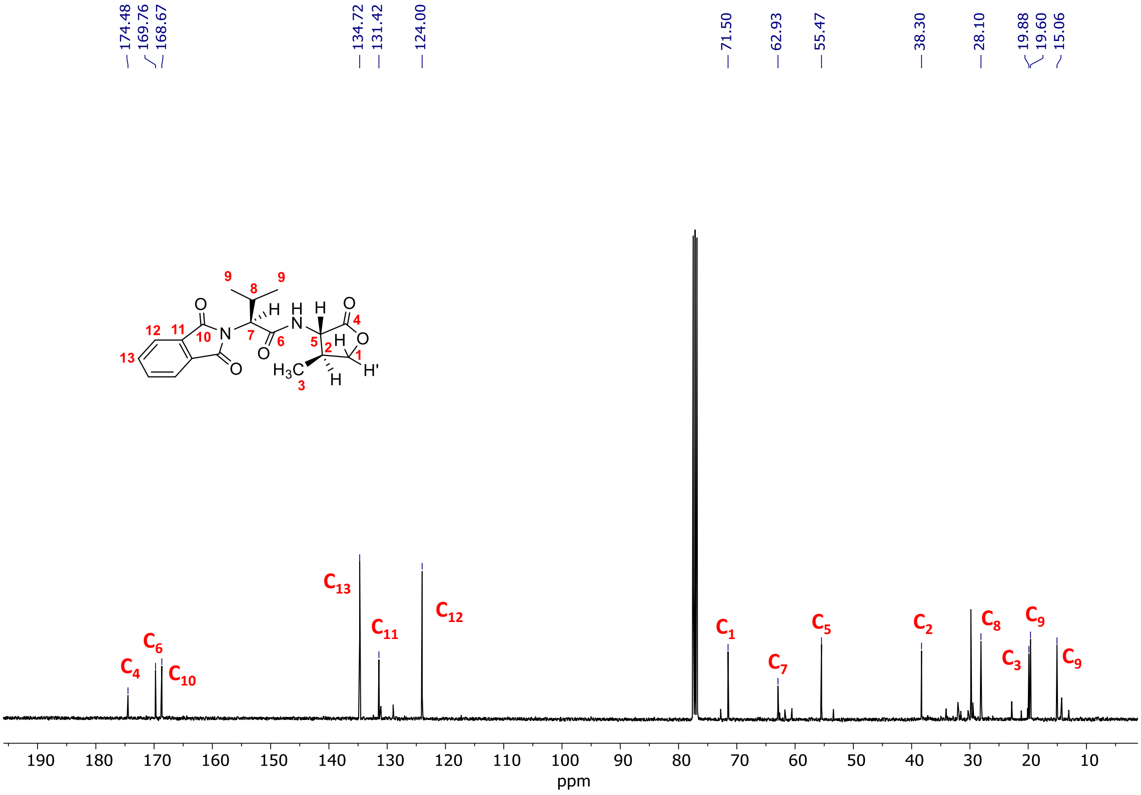

^{1}H-^{1}H COSY of **12b** in $CDCl_3$

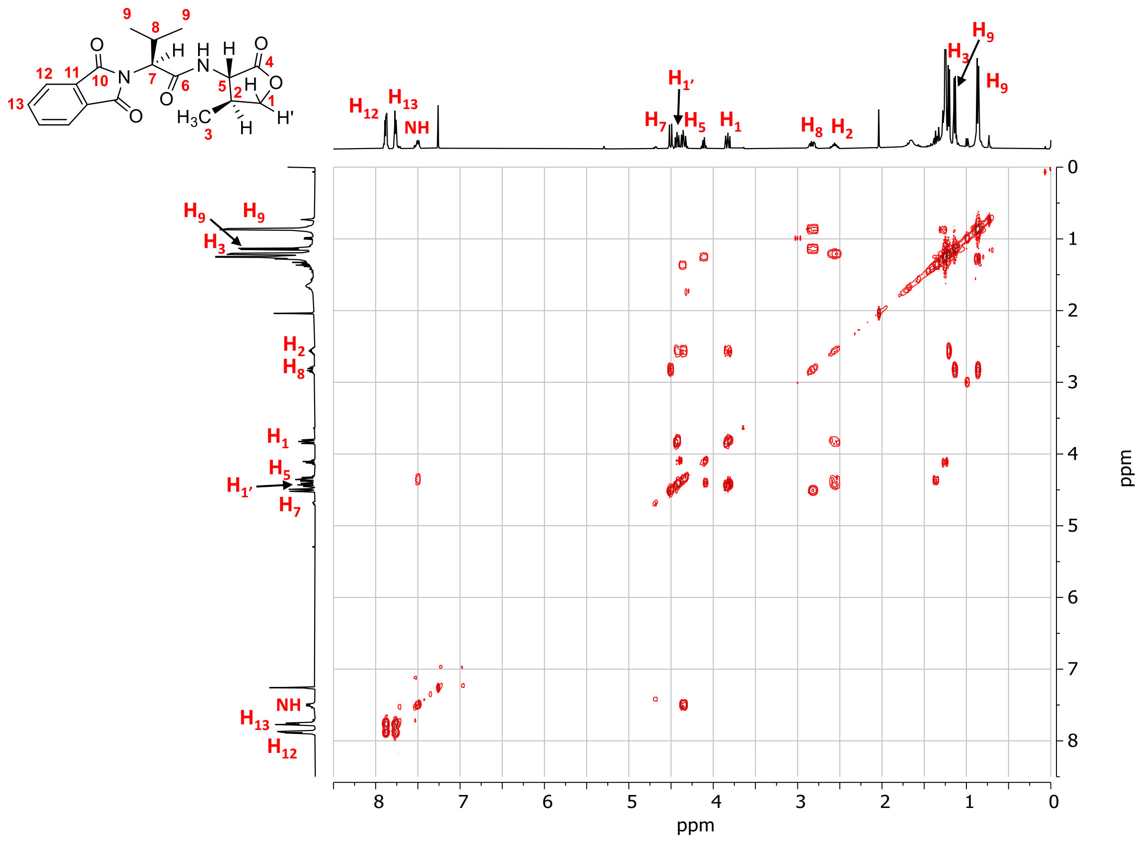

^{1}H-^{13}C HSQCed of **12b** in $CDCl_3$

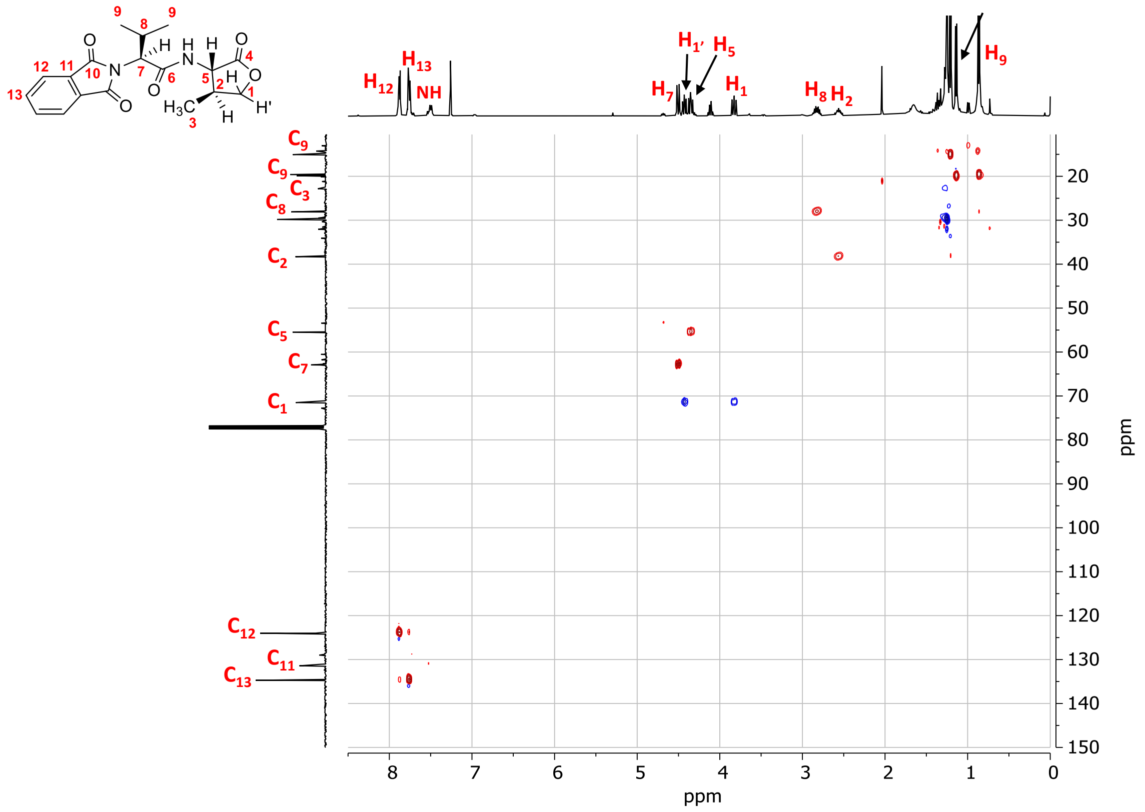

DEPTQ of **12b** in $CDCl_3$

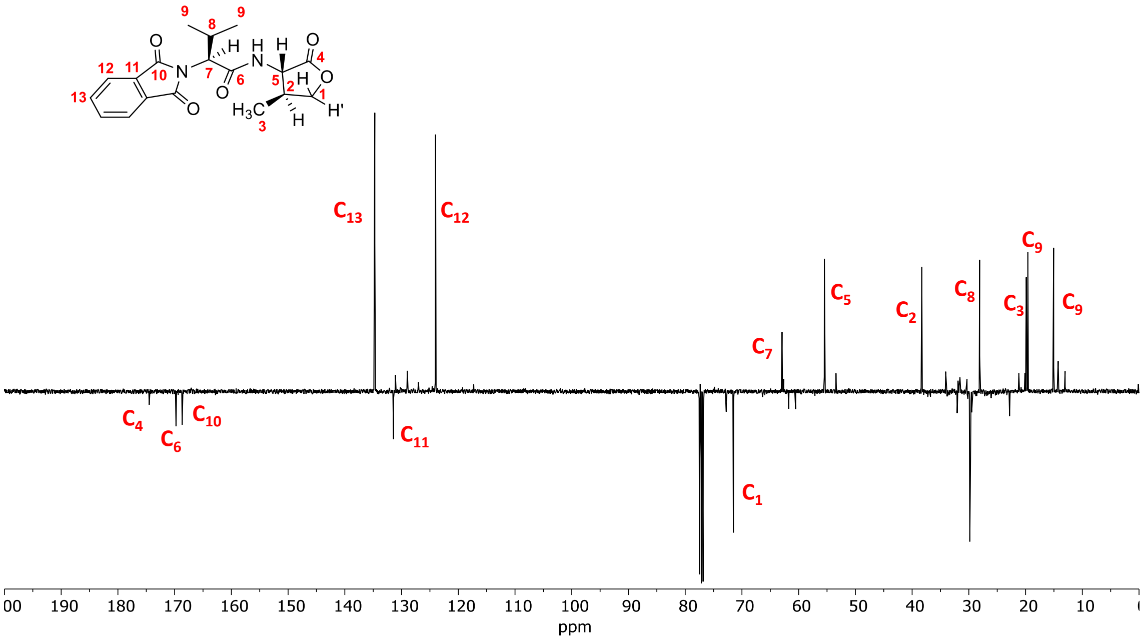

^{1}H-^{13}C HMBC of **12b** in $CDCl_3$

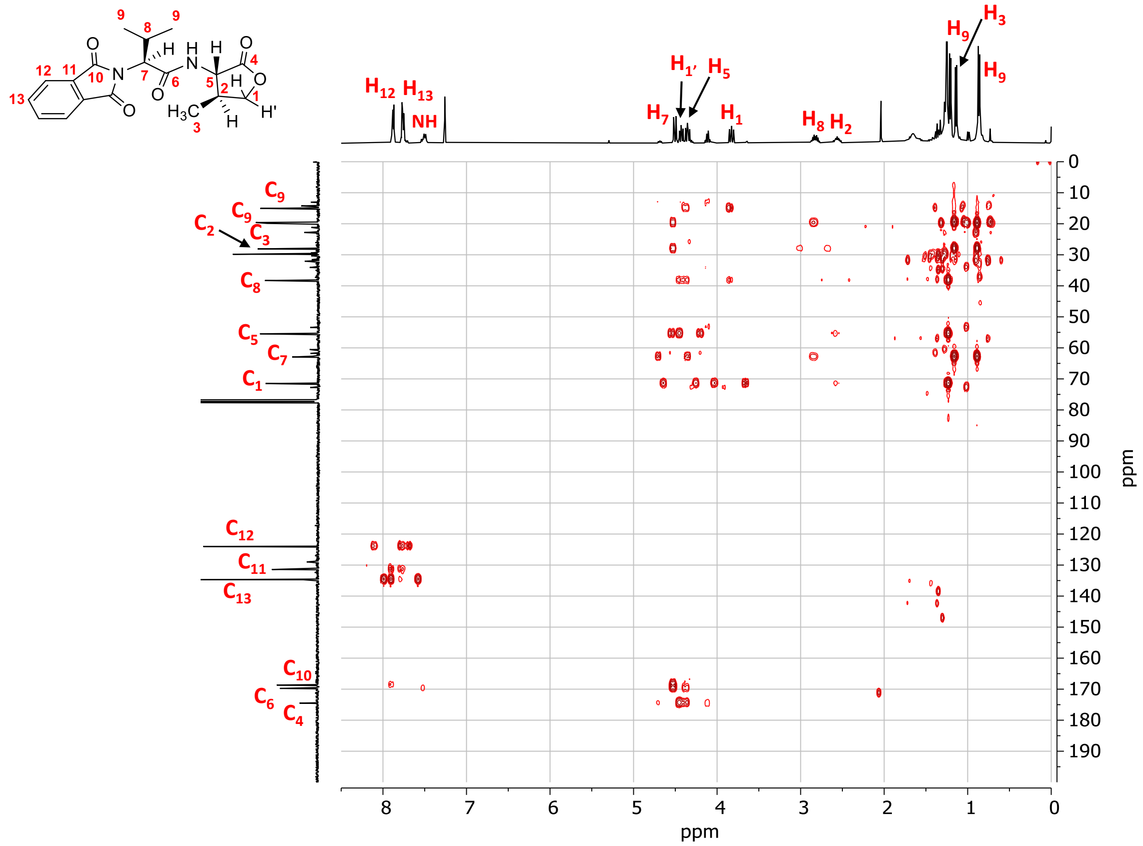

^1H-^1H TOCSY of **12b** in $CDCl_3$

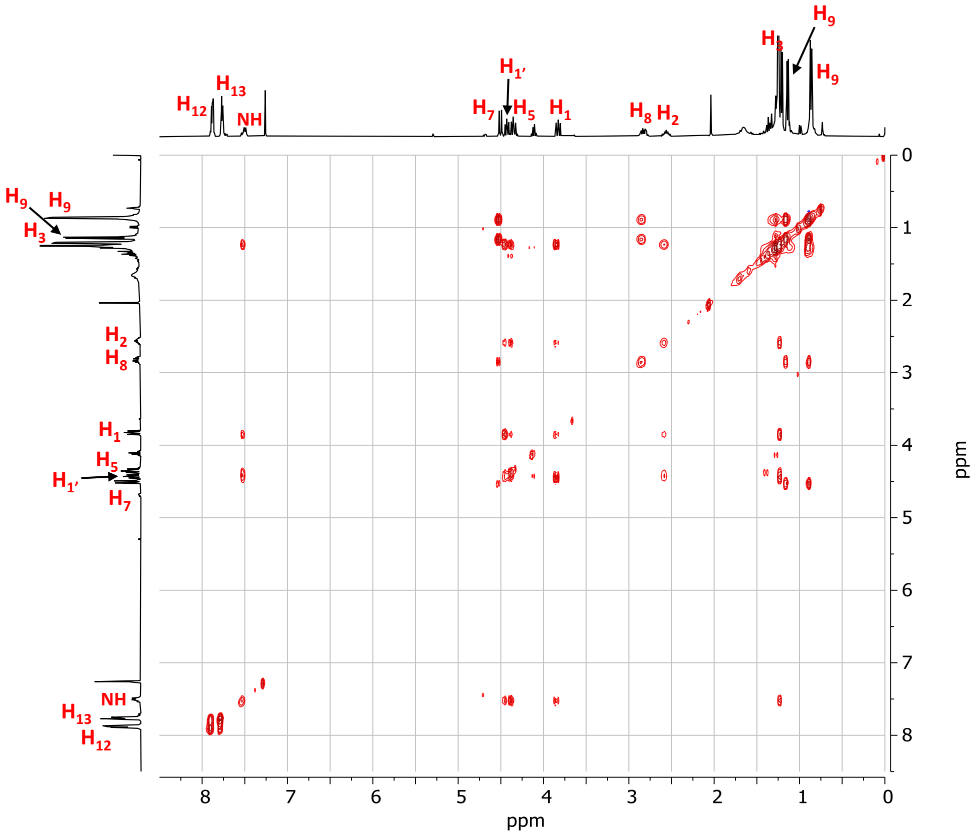

^1H-^1H NOESY of **12b** in $CDCl_3$

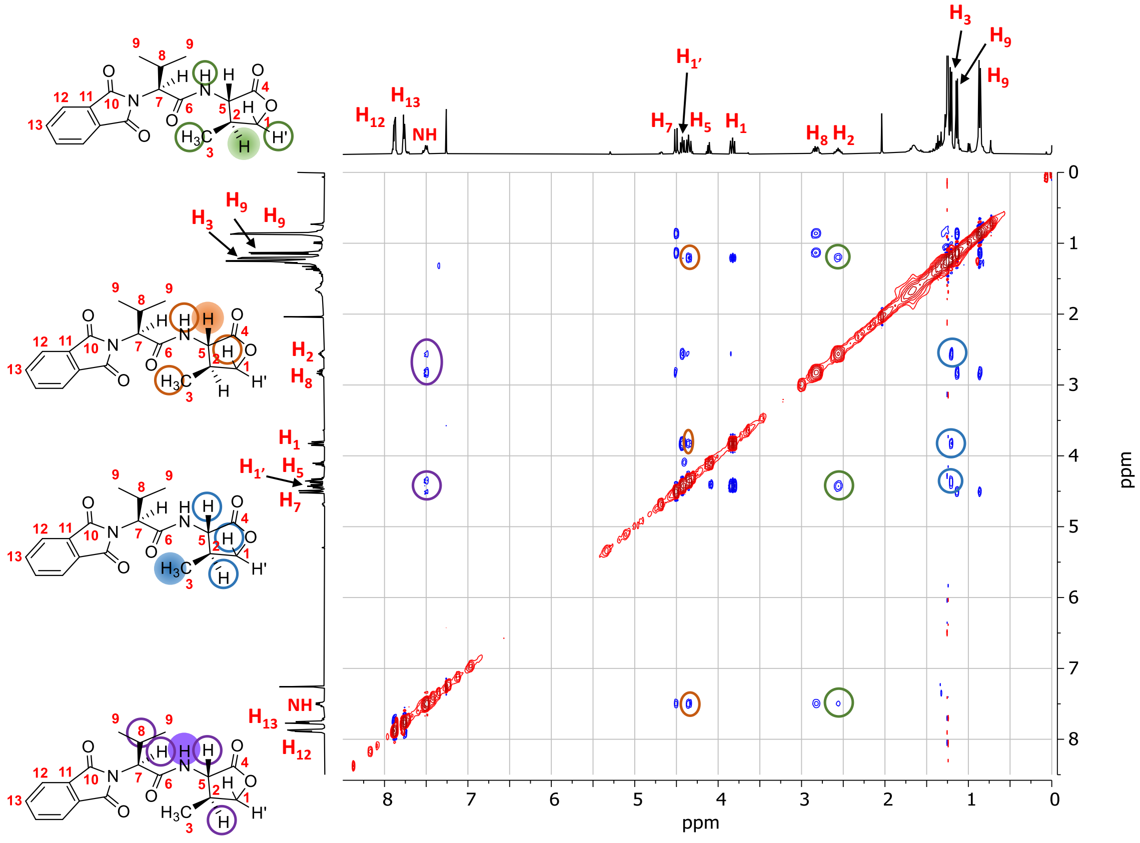

Selective NOESY experiments of **12b** in $CDCl_3$

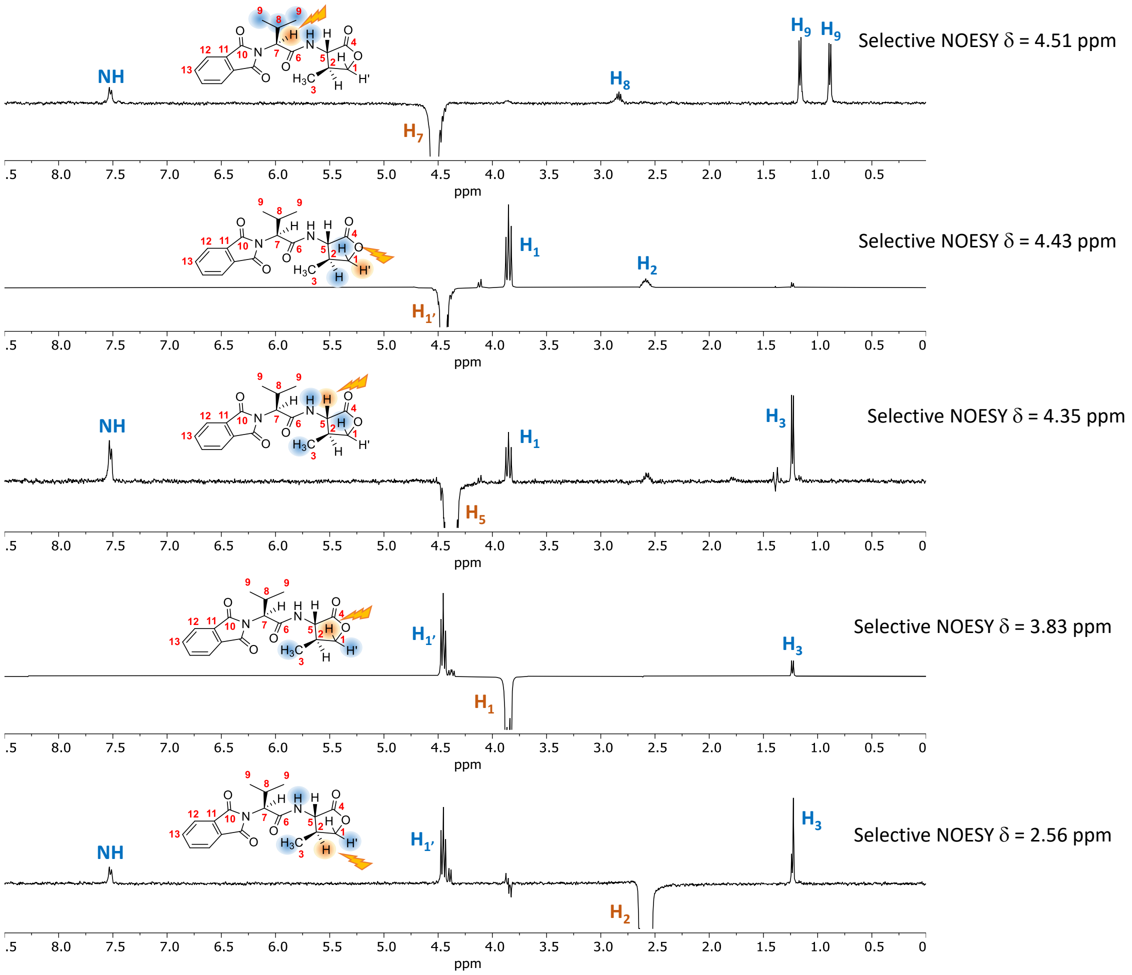

^{1}H-NMR of **12c** in $CDCl_3$

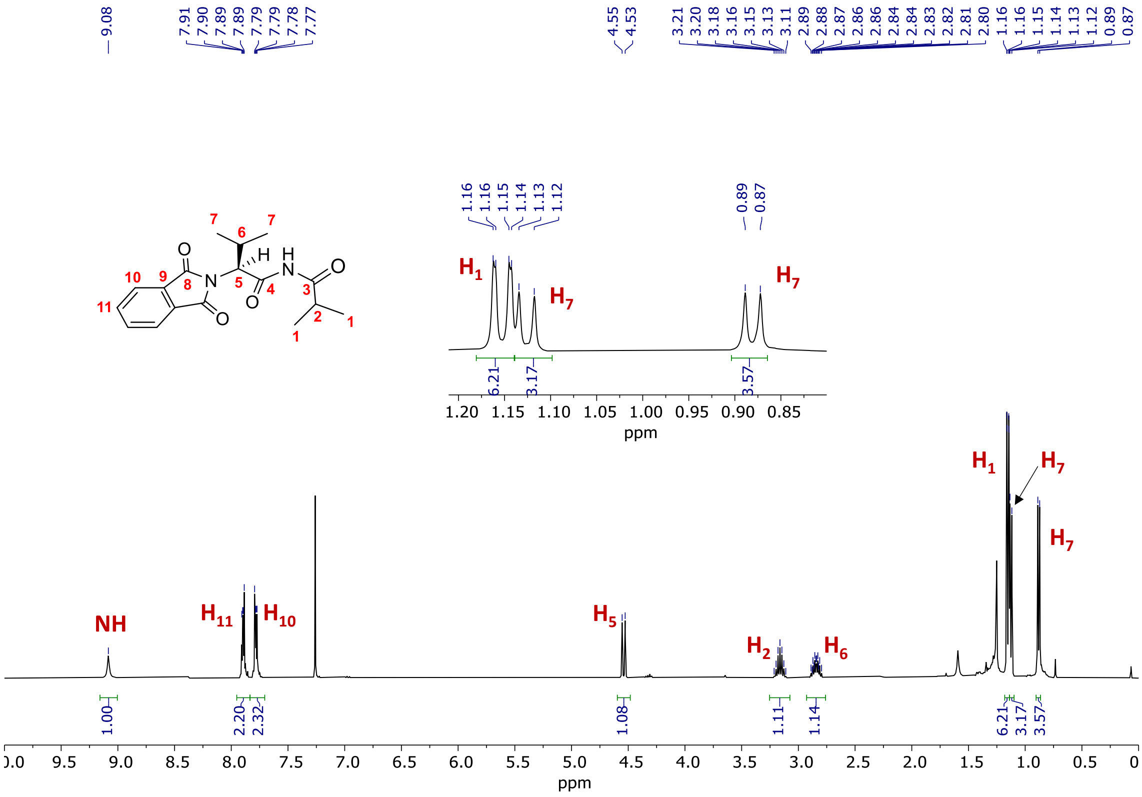

^{13}C-NMR of **12c** in $CDCl_3$

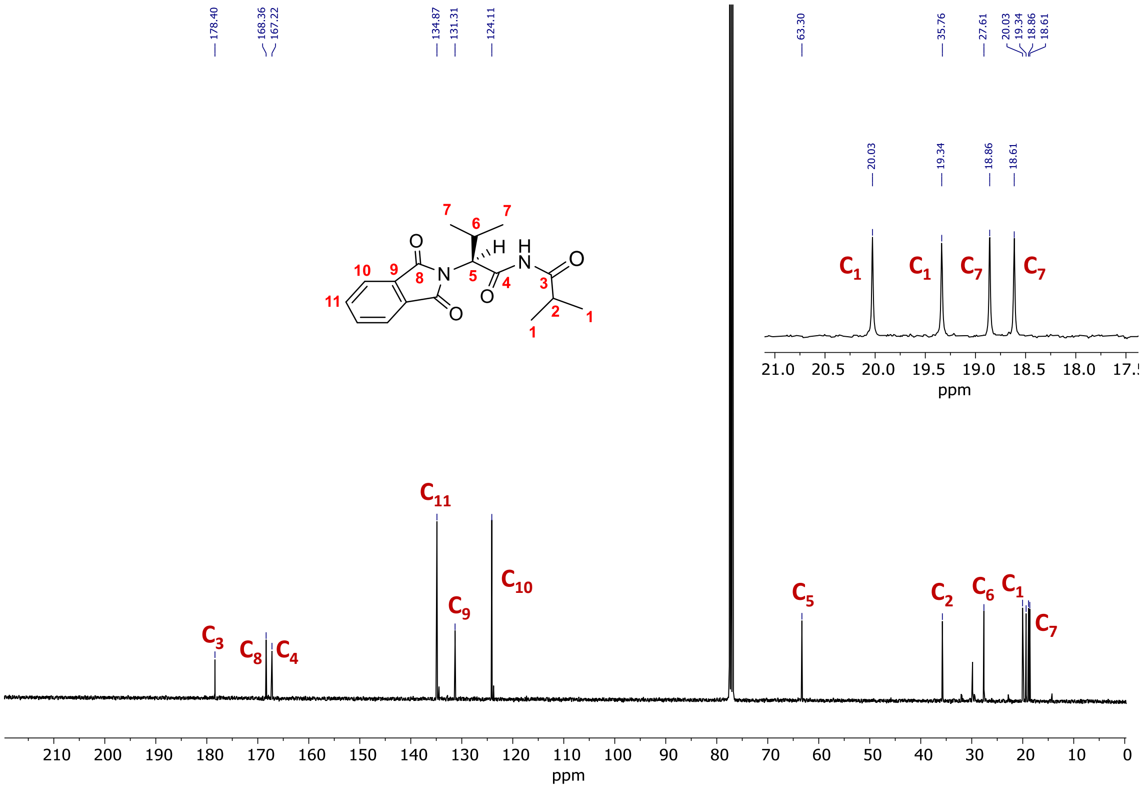

^{1}H-^{1}H COSY of **12c** in $CDCl_3$

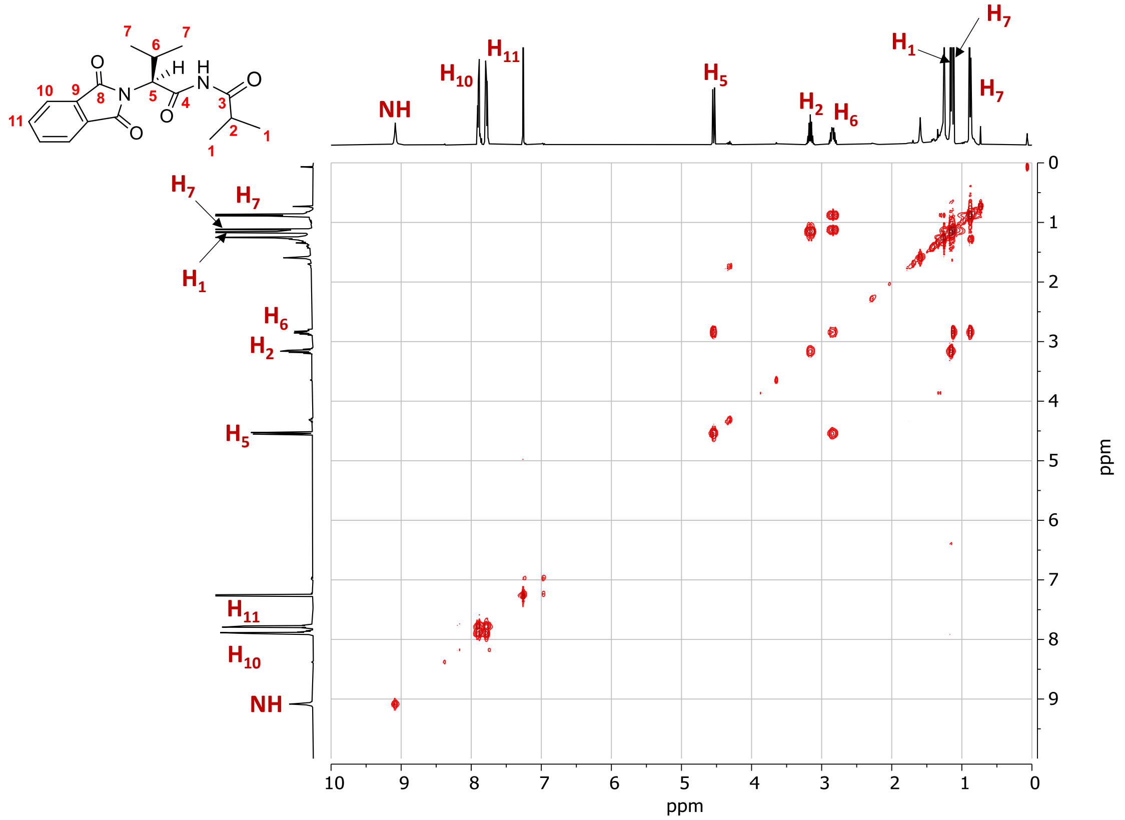

^{1}H-^{13}C HSQCed of **12c** in $CDCl_3$

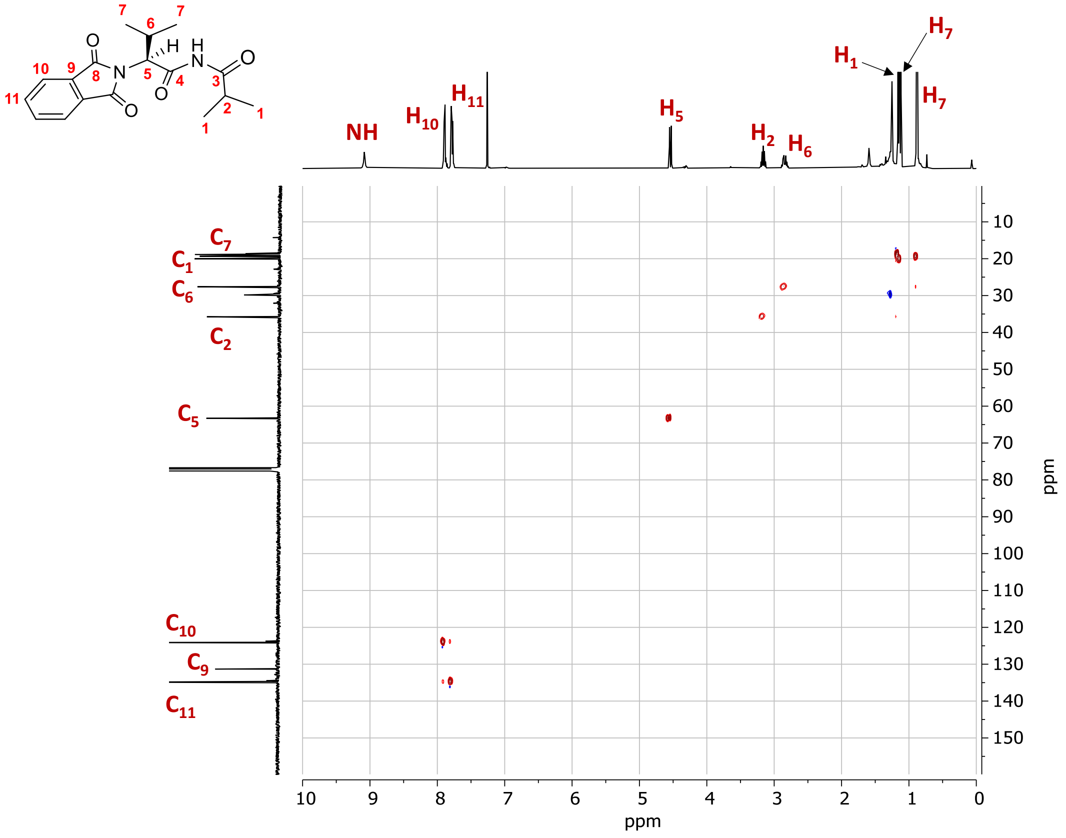

DEPTQ of **12c** in $CDCl_3$

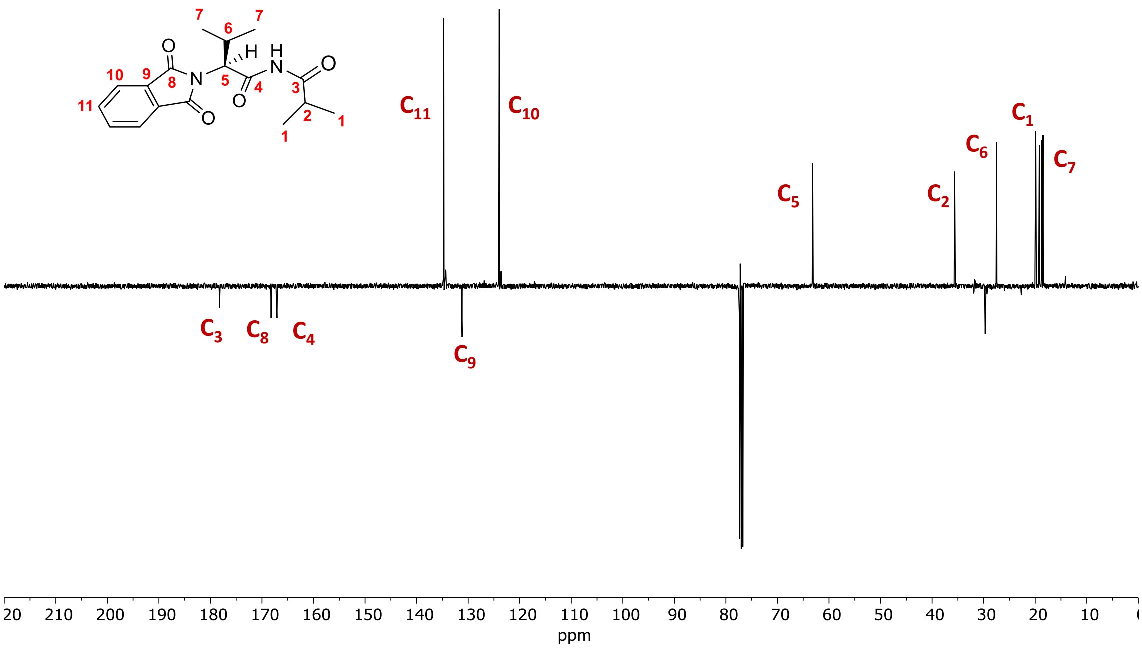

^1H-^{13}C HMBC of **12c** in $CDCl_3$

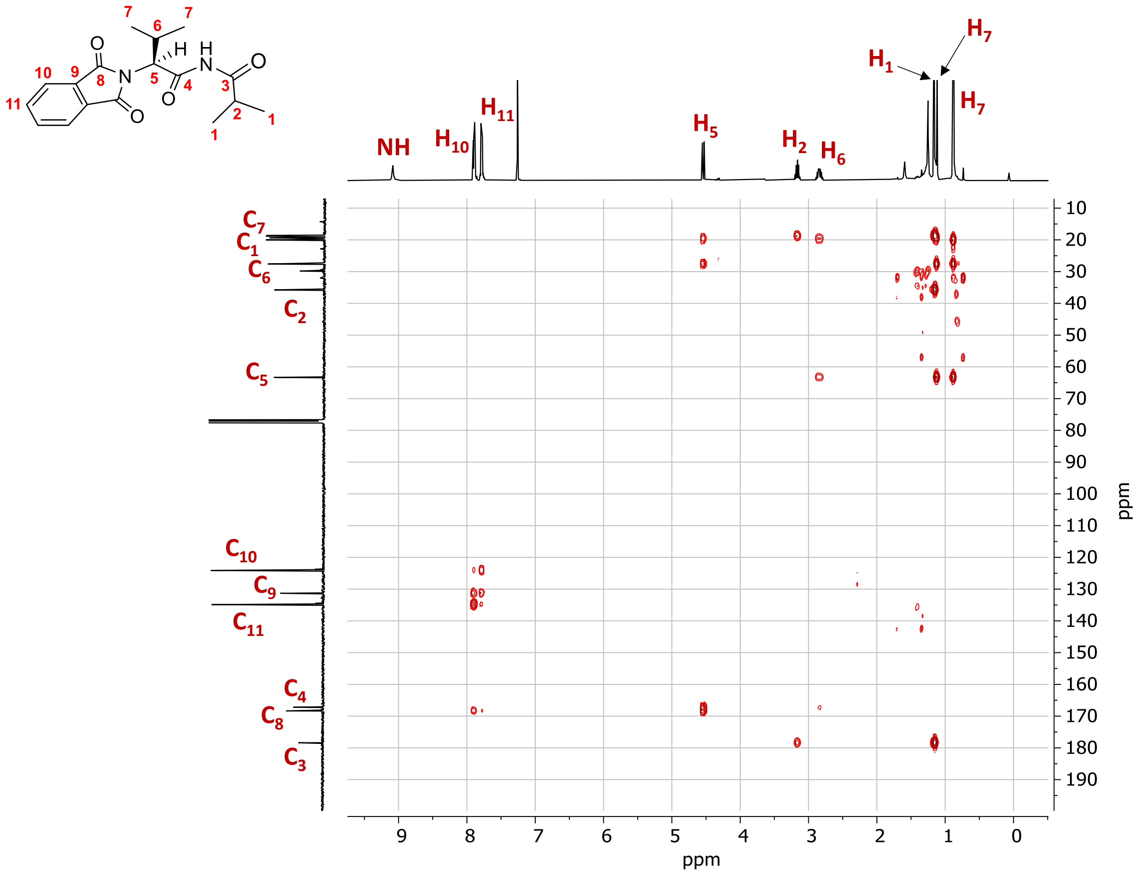

^{1}H-^{1}H TOCSY of **12c** in $CDCl_3$

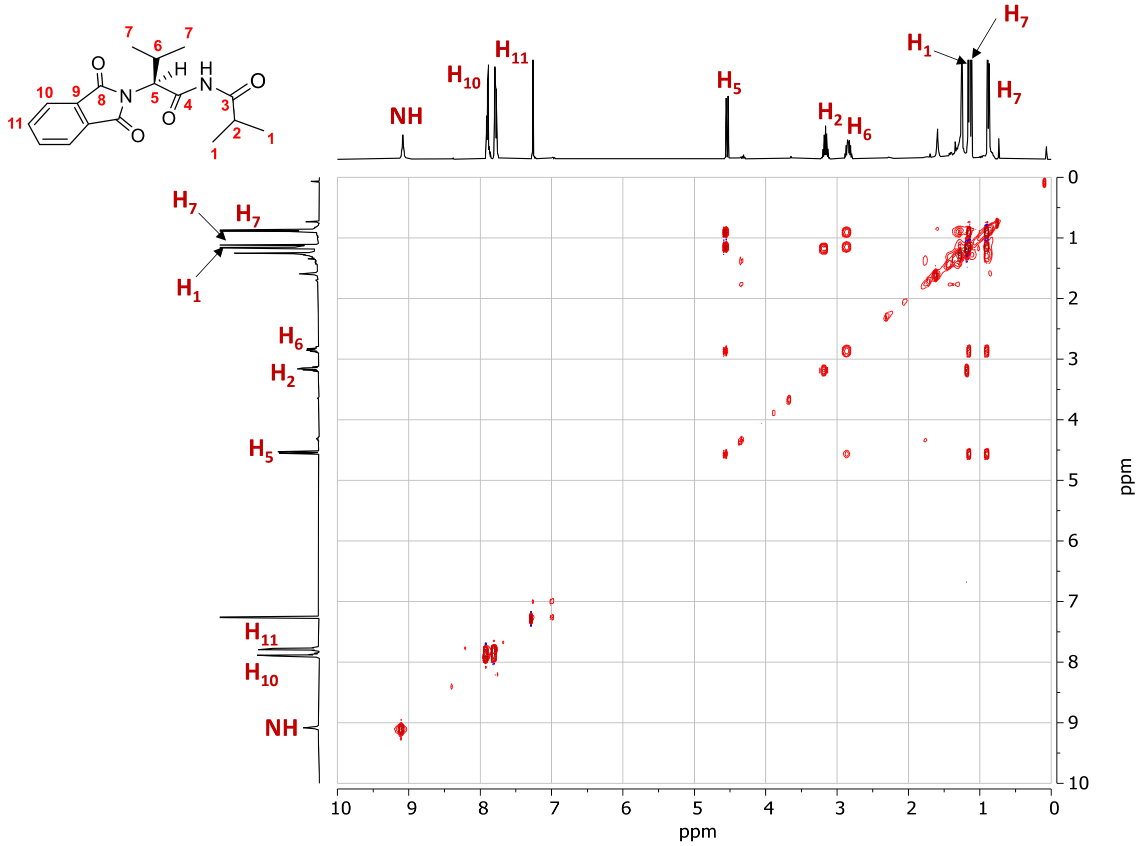

^{1}H-^{1}H NOESY of **12c** in $CDCl_3$

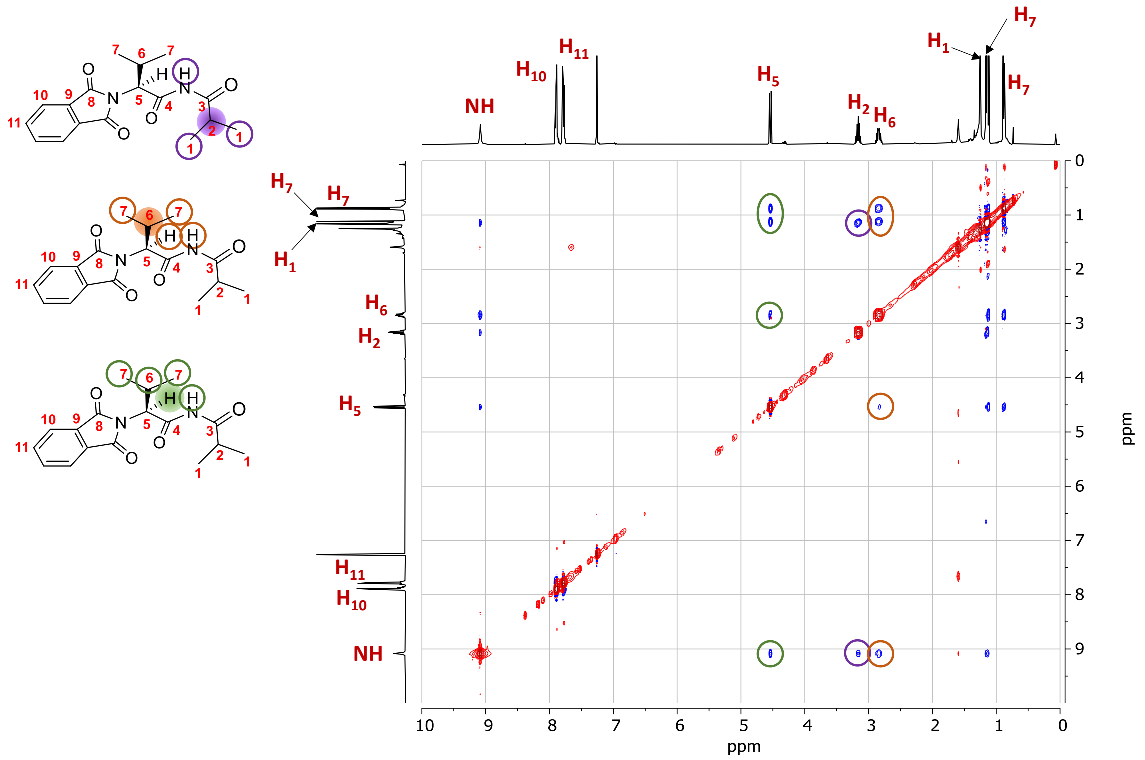

Selective TOCSY experiments of **12c** in $CDCl_3$

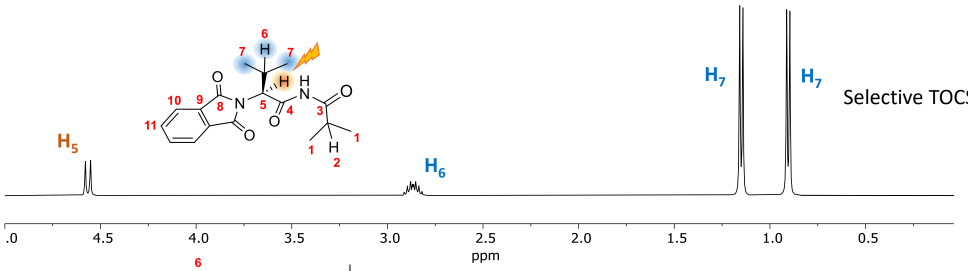

Selective TOCSY δ = 4.54 ppm

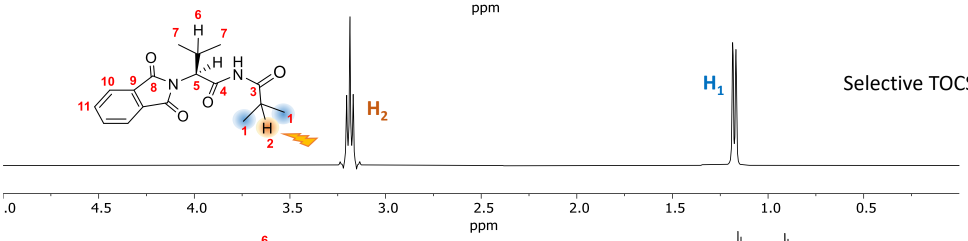

Selective TOCSY δ = 3.16 ppm

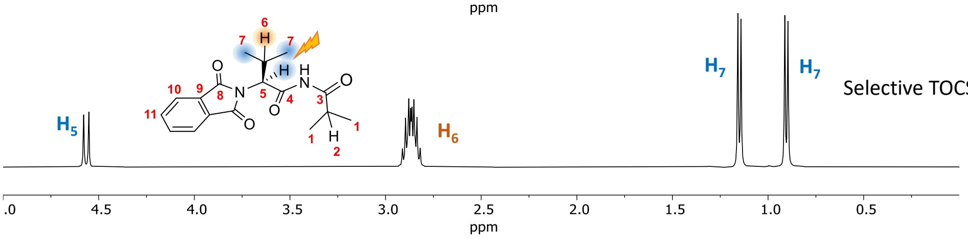

Selective TOCSY δ = 2.84 ppm

^{1}H-NMR of **13a** in $CDCl_3$

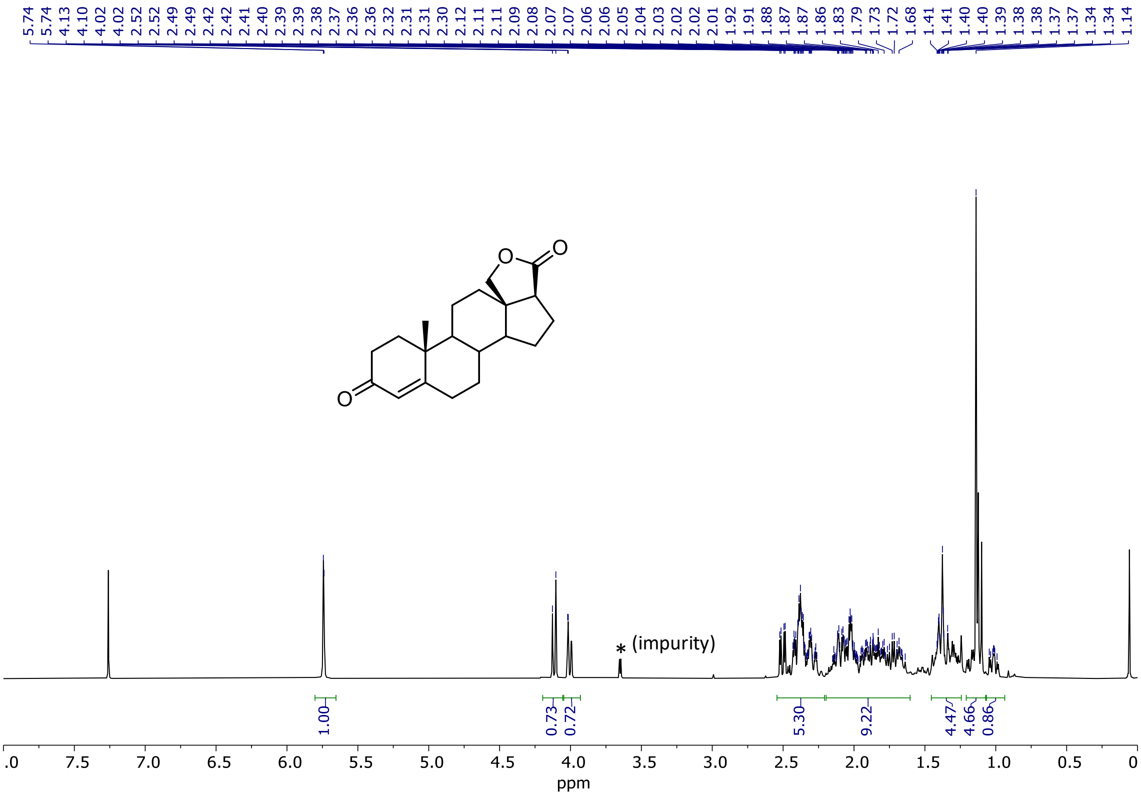

^{13}C-NMR of **13a** in $CDCl_3$

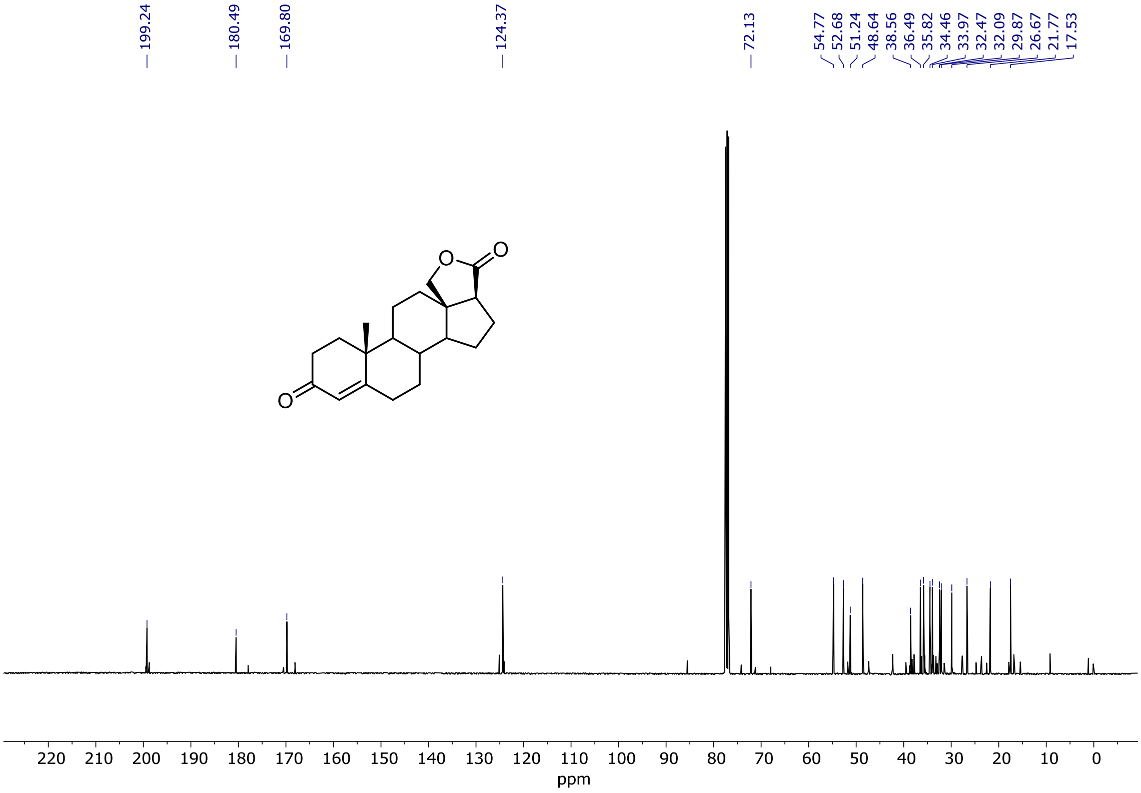

Note: the additional small peaks correspond to an unidentified oxidation product that could not be removed.

^{1}H-NMR of **15a** in $CDCl_3$

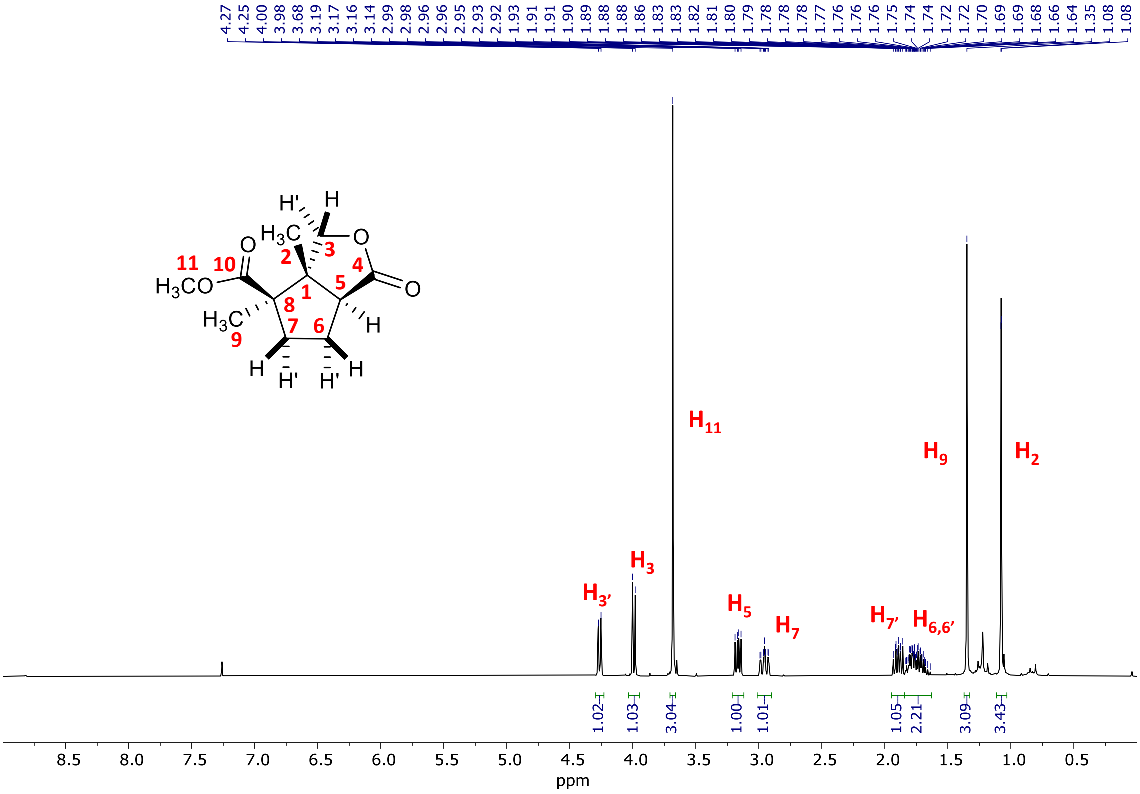

^{13}C-NMR of **15a** in $CDCl_3$

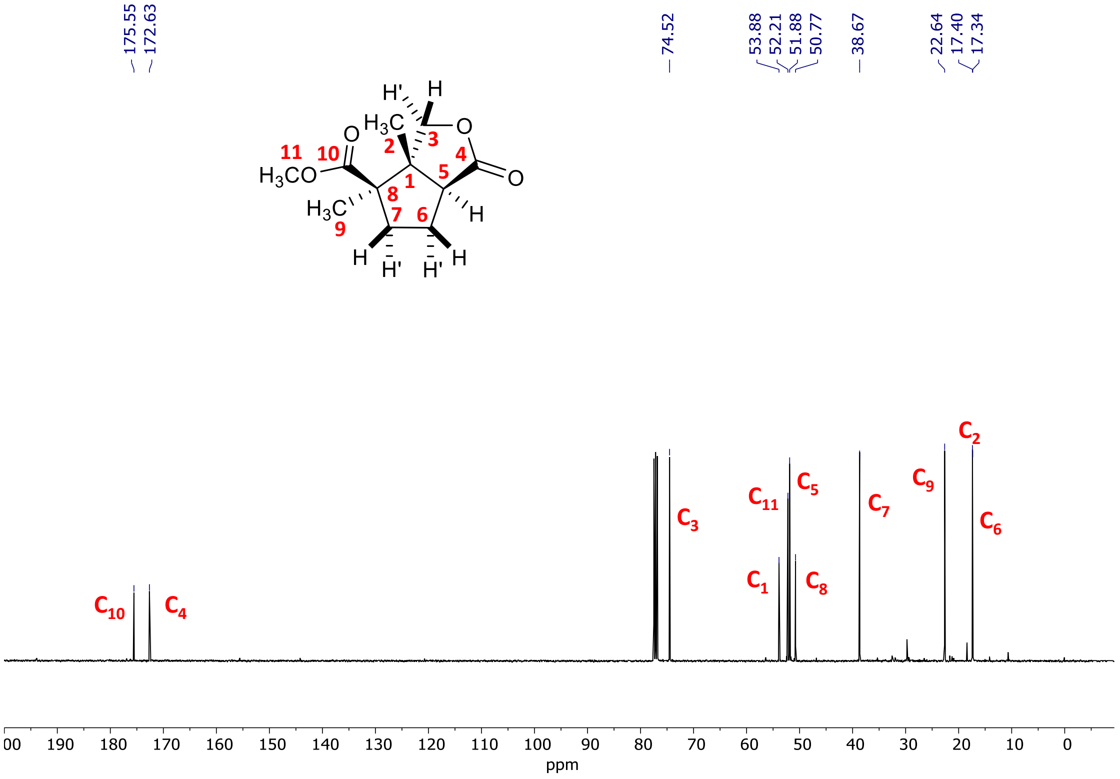

^{1}H-^{1}H COSY of **15a** in $CDCl_3$

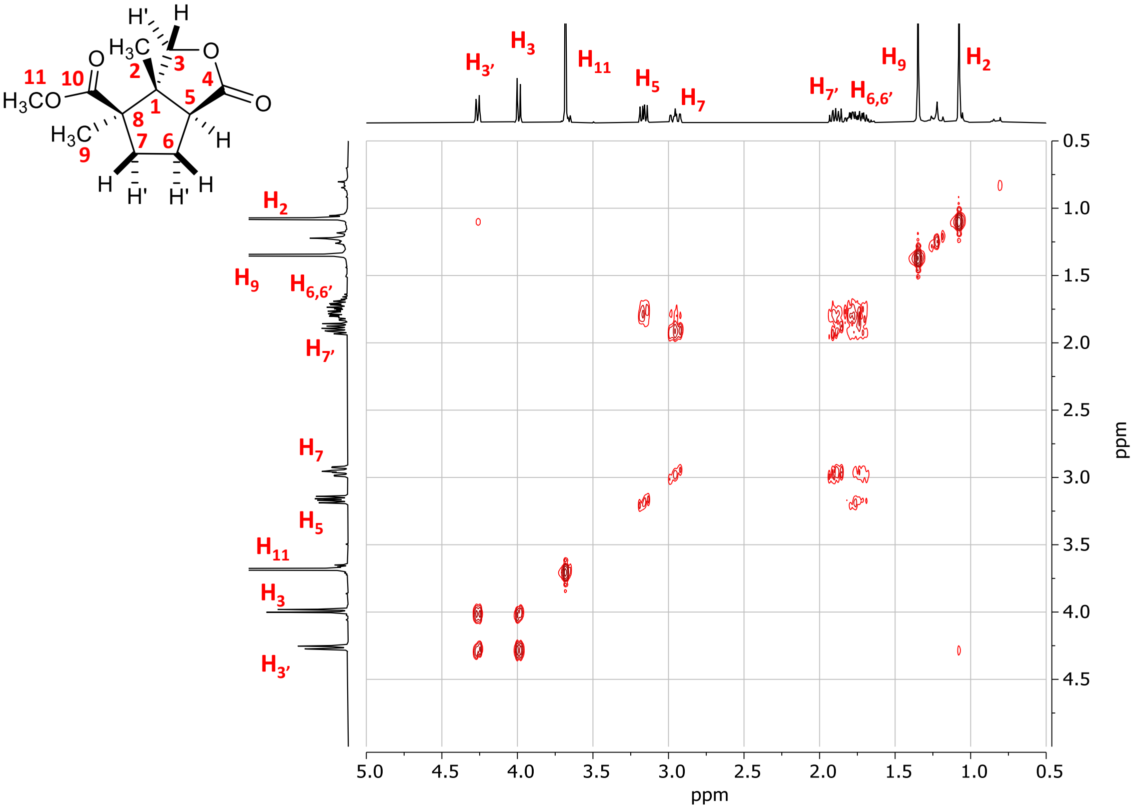

^{1}H-^{13}C HSQCed of **15a** in $CDCl_3$

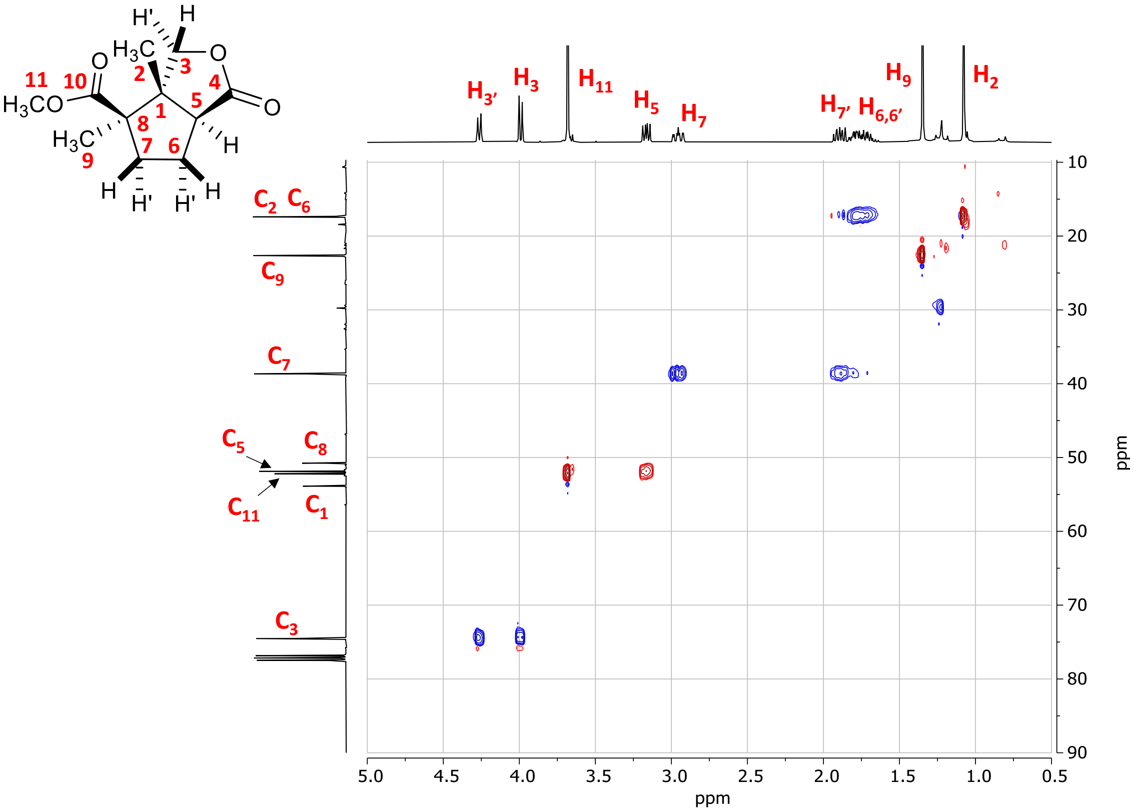

DEPTQ of **15a** in $CDCl_3$

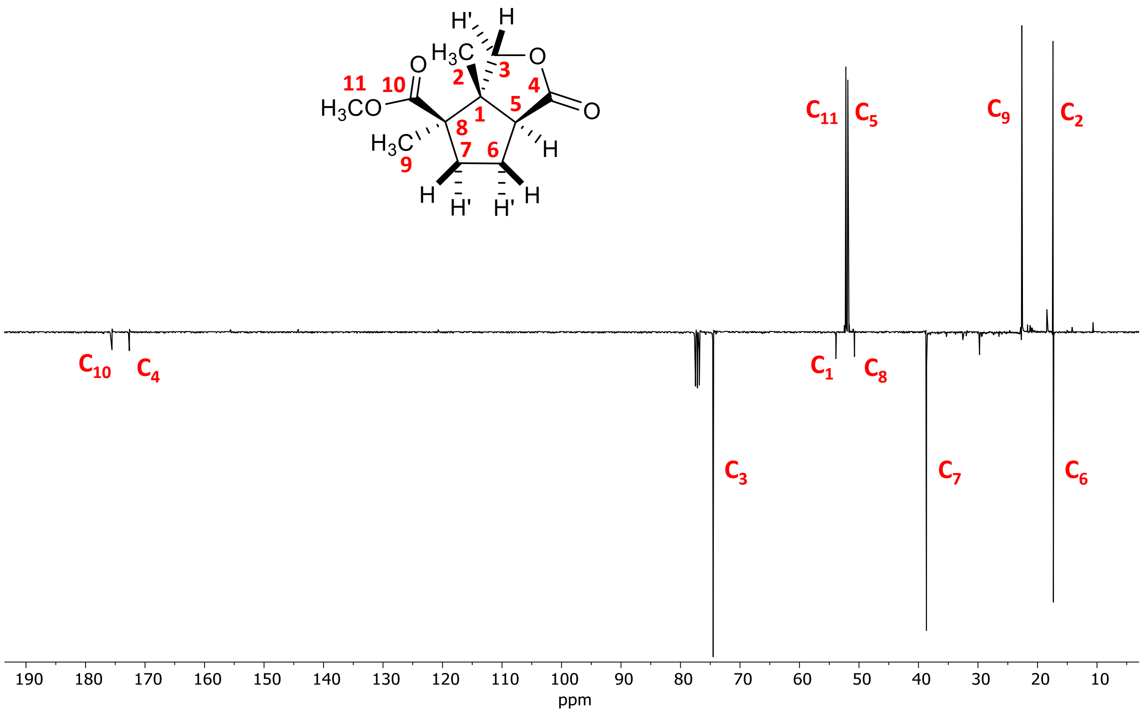

^{1}H-^{1}H TOCSY of **15a** in $CDCl_3$

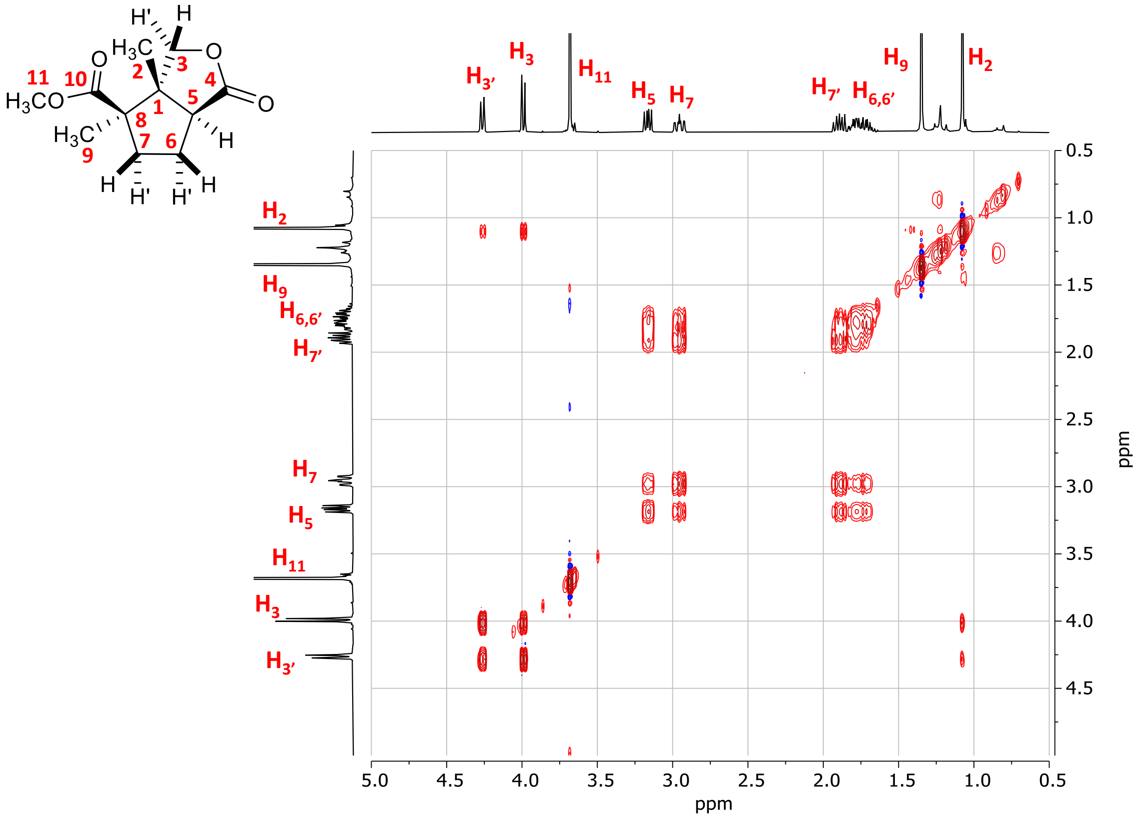

^{1}H-^{13}C HMBC of **15a** in $CDCl_3$

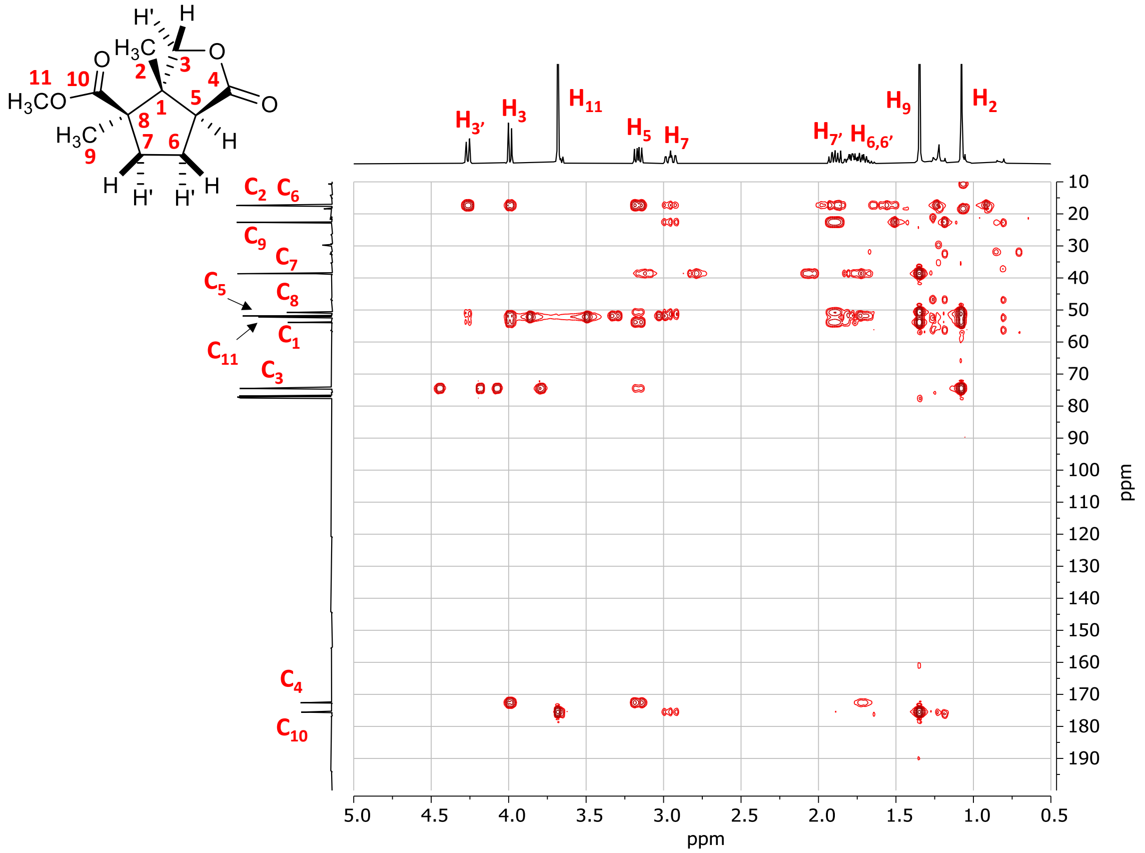

^{1}H-^{1}H NOESY of **15a** in $CDCl_3$

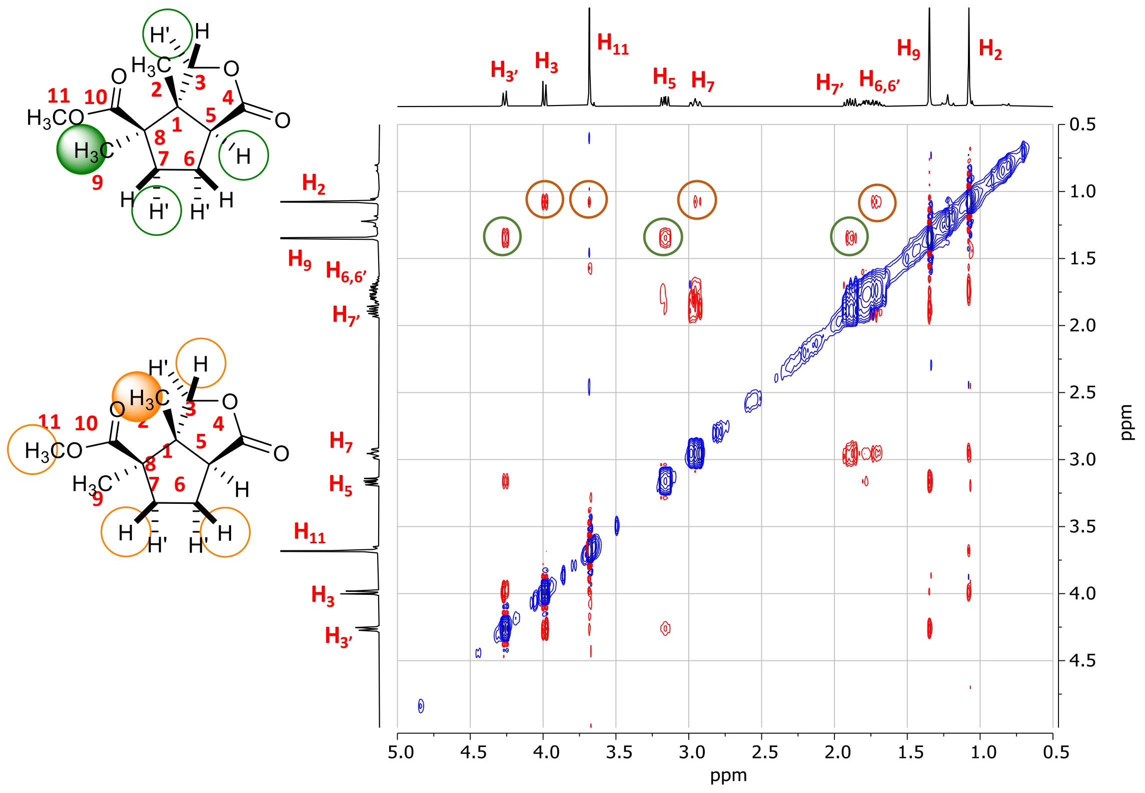

Selective NOESY experiments of **15a** in $CDCl_3$

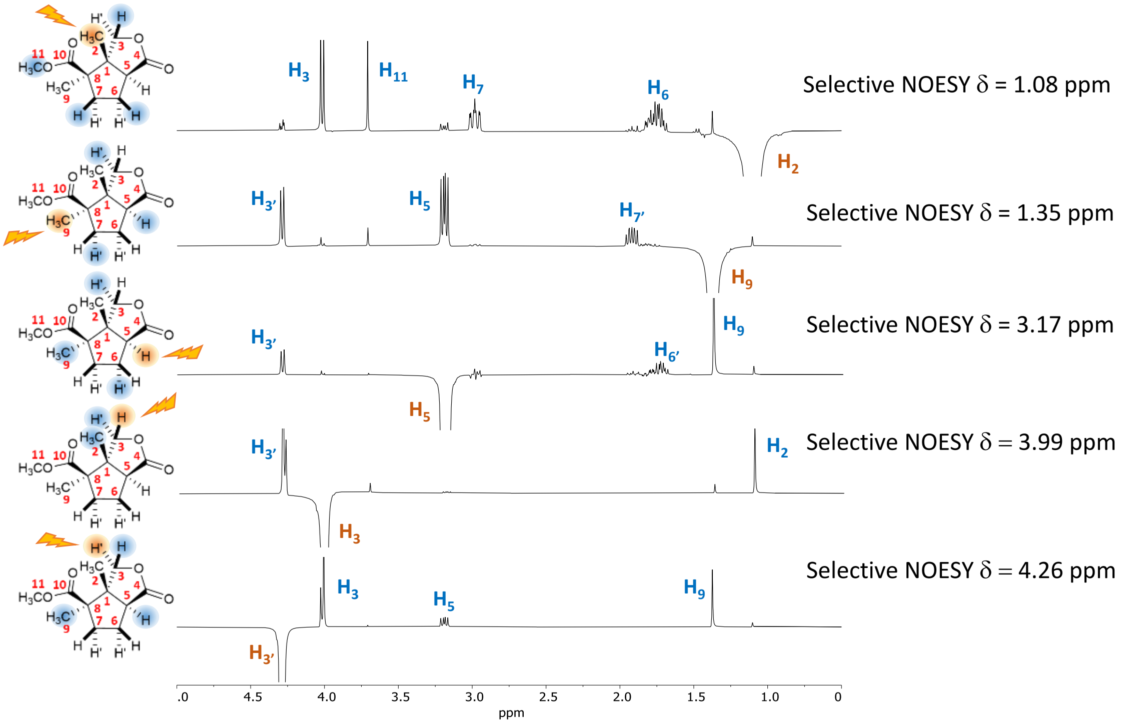

^{1}H-NMR of **16a** in $CDCl_3$

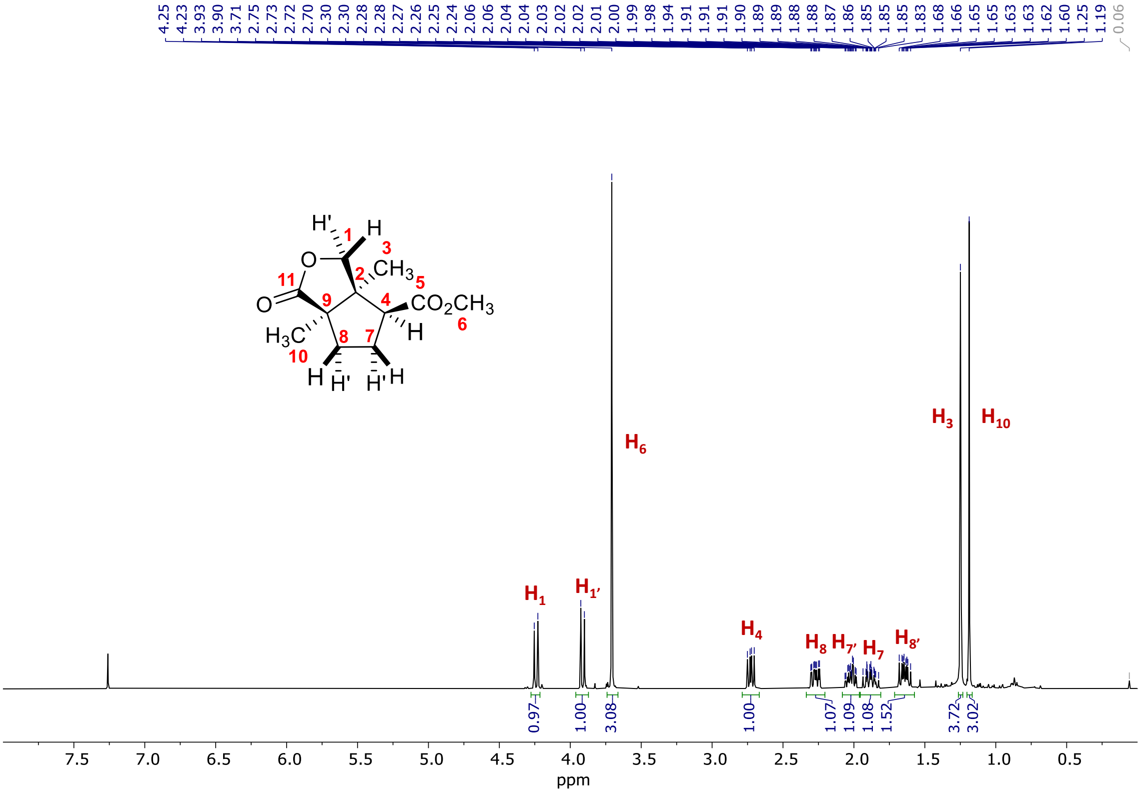

^{13}C-NMR of **16a** in $CDCl_3$

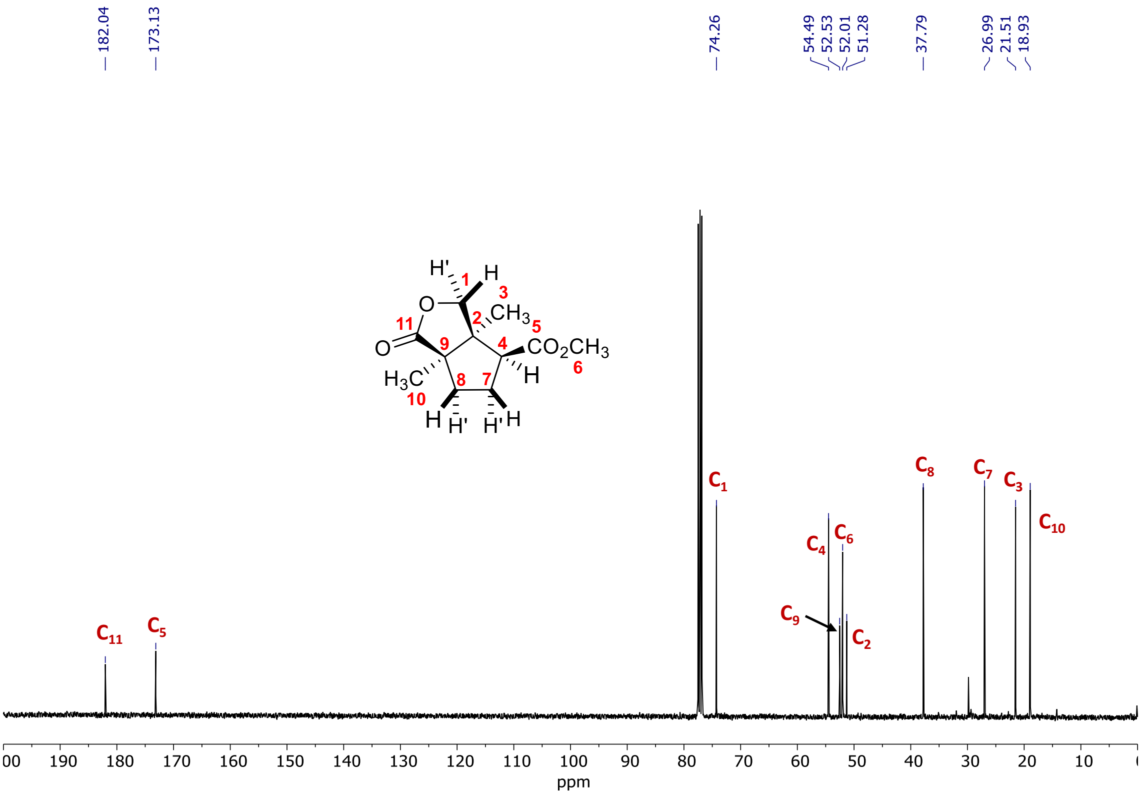

^{1}H-^{1}H COSY of **16a** in $CDCl_3$

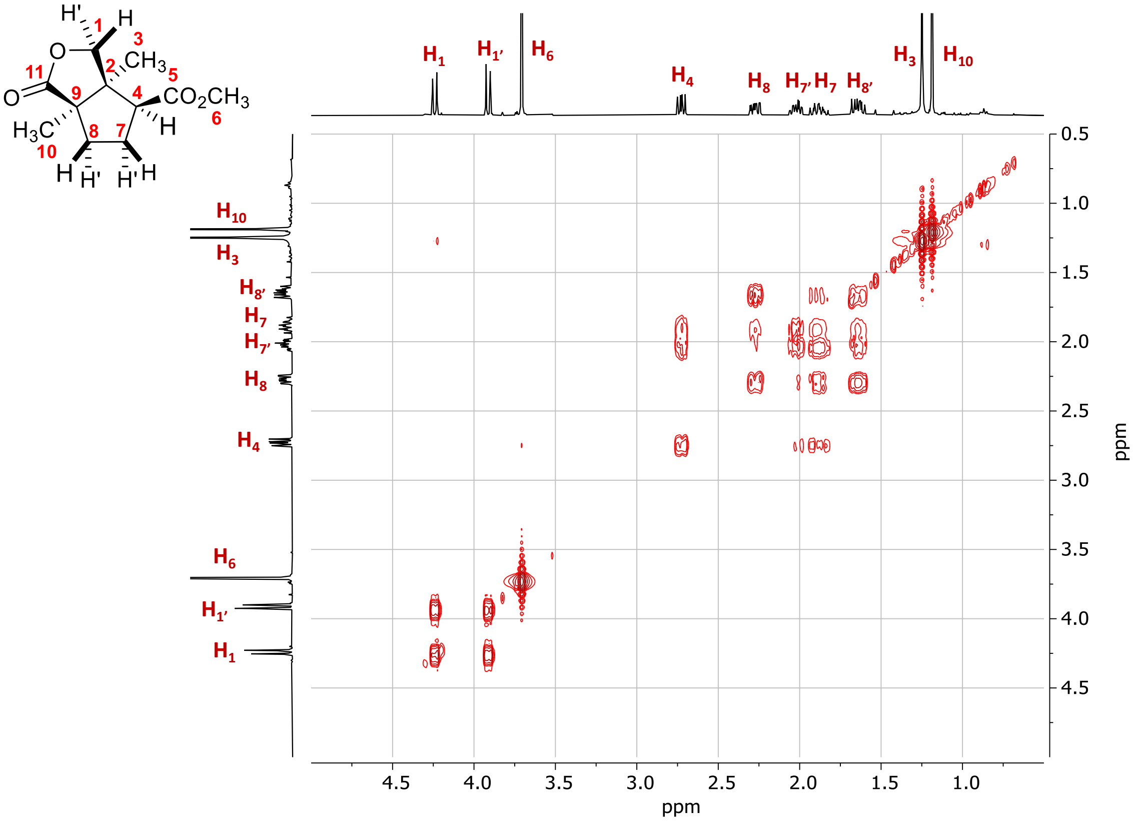

^{1}H-^{13}C HSQCed of **16a** in $CDCl_3$

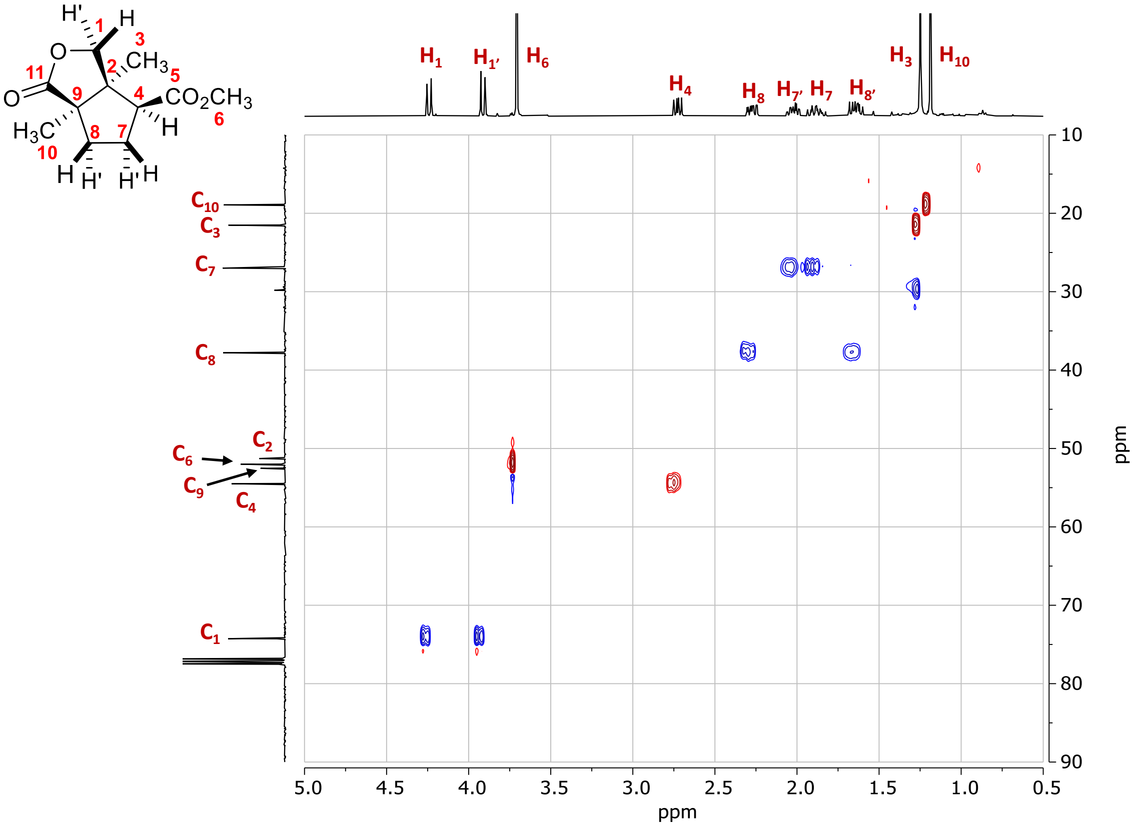

DEPTQ of **16a** in $CDCl_3$

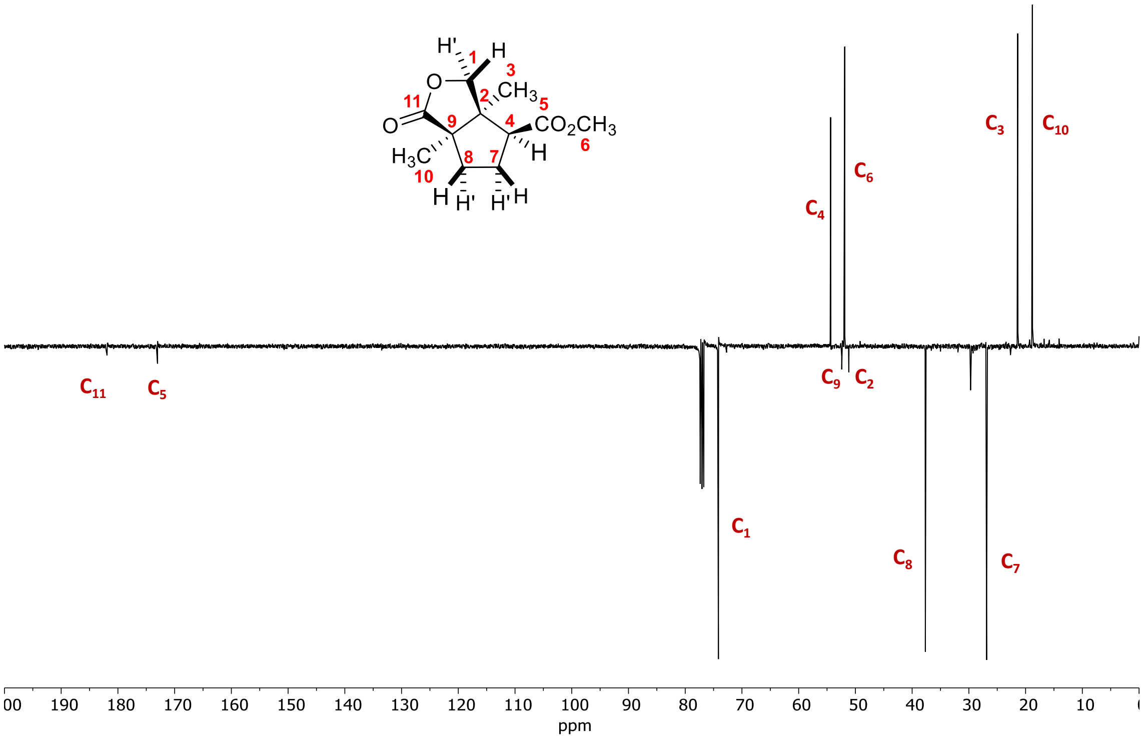

^{1}H-^{13}C HMBC of **16a** in $CDCl_3$

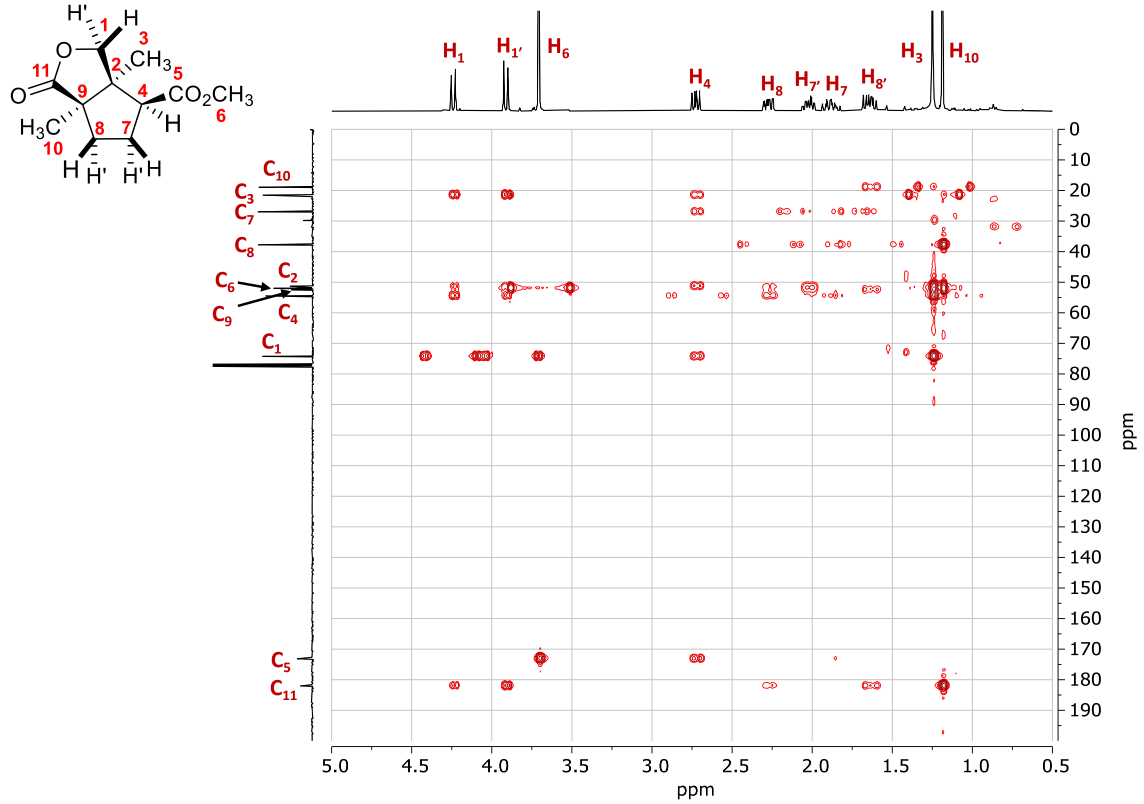

^1H-^1H TOCSY of **16a** in $CDCl_3$

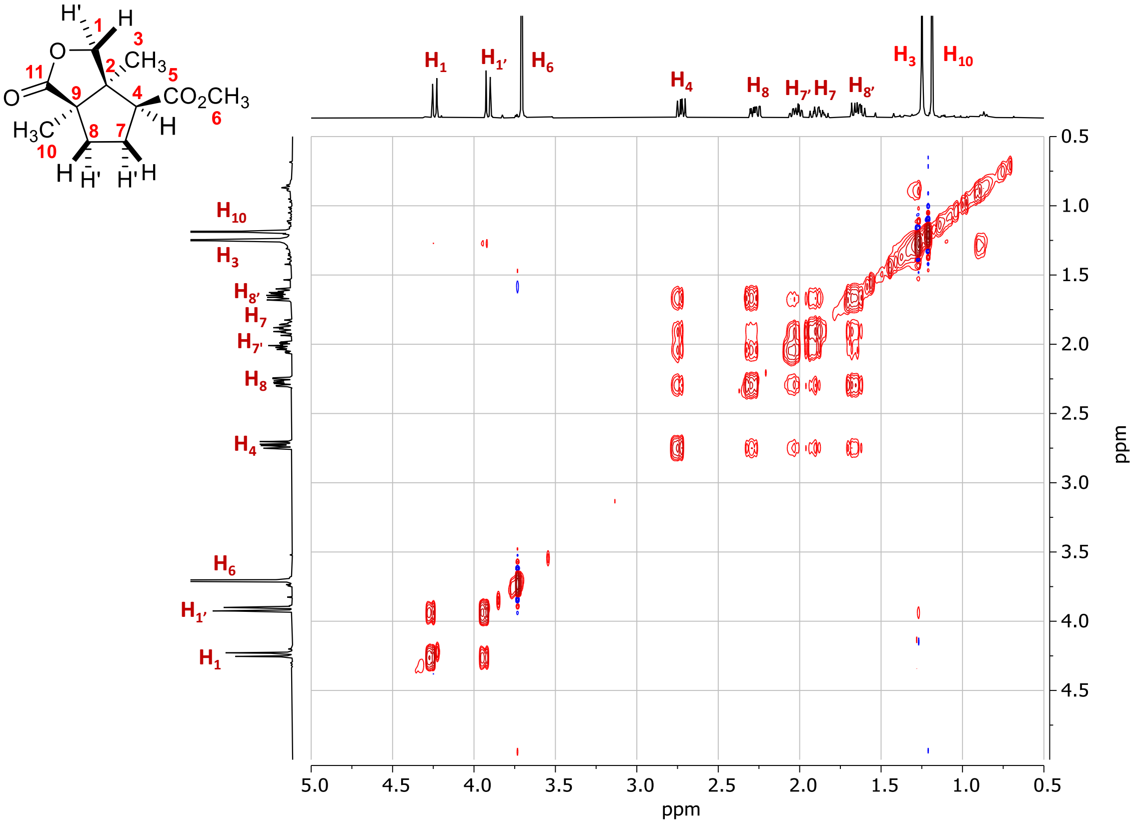

^1H-^1H NOESY of **16a** in $CDCl_3$

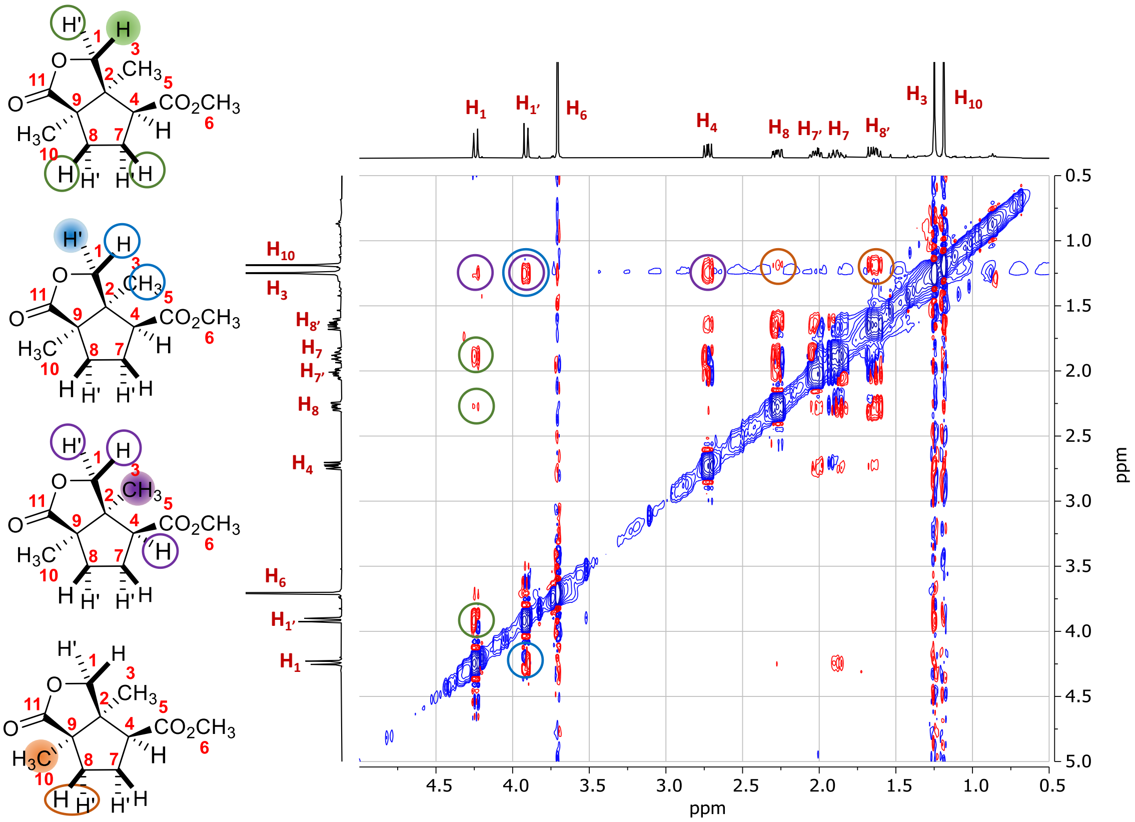

Selective NOESY experiments of **16a** in $CDCl_3$

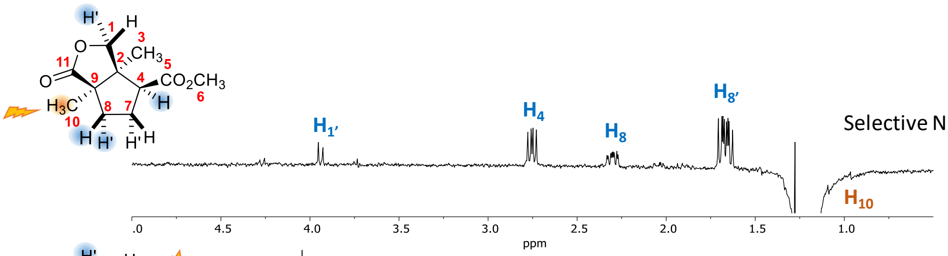

Selective NOESY δ = 1.19 ppm

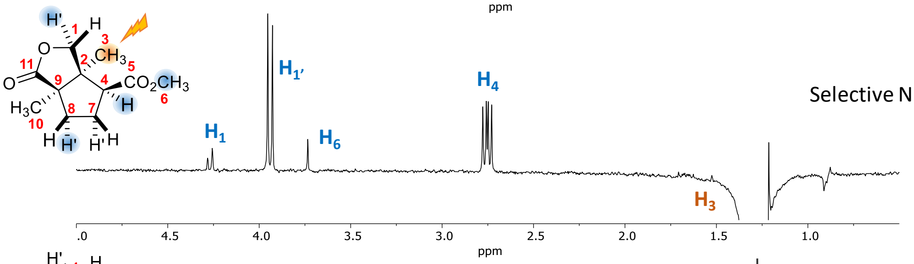

Selective NOESY δ = 1.25 ppm

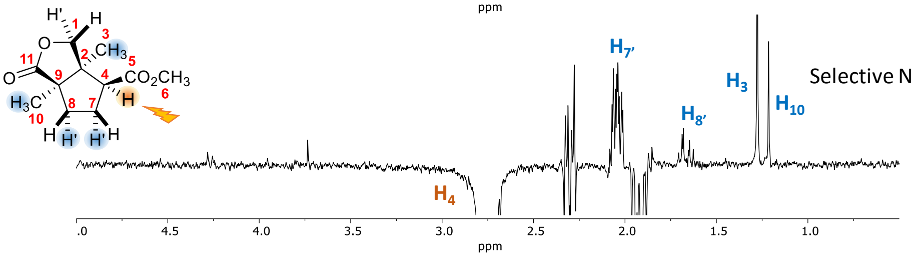

Selective NOESY δ = 2.73 ppm

^{1}H-NMR of **16b** in CDCl$_3$

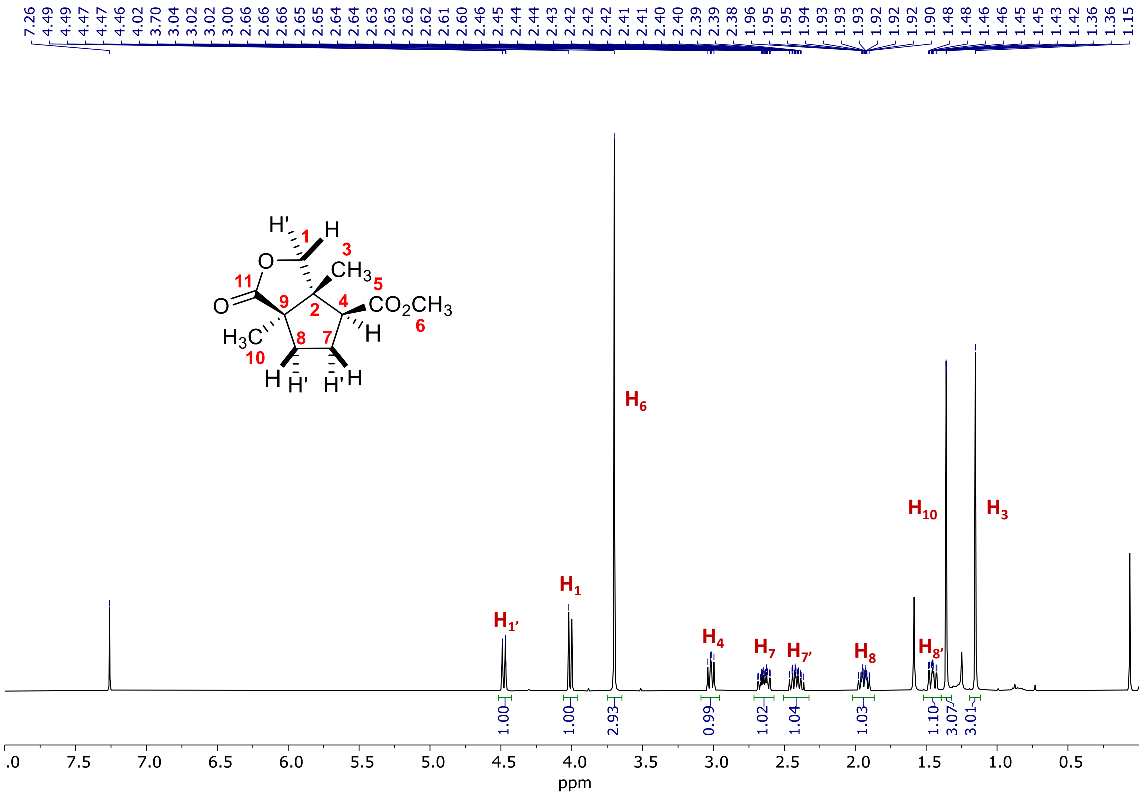

^{13}C-NMR of **16b** in CDCl$_3$

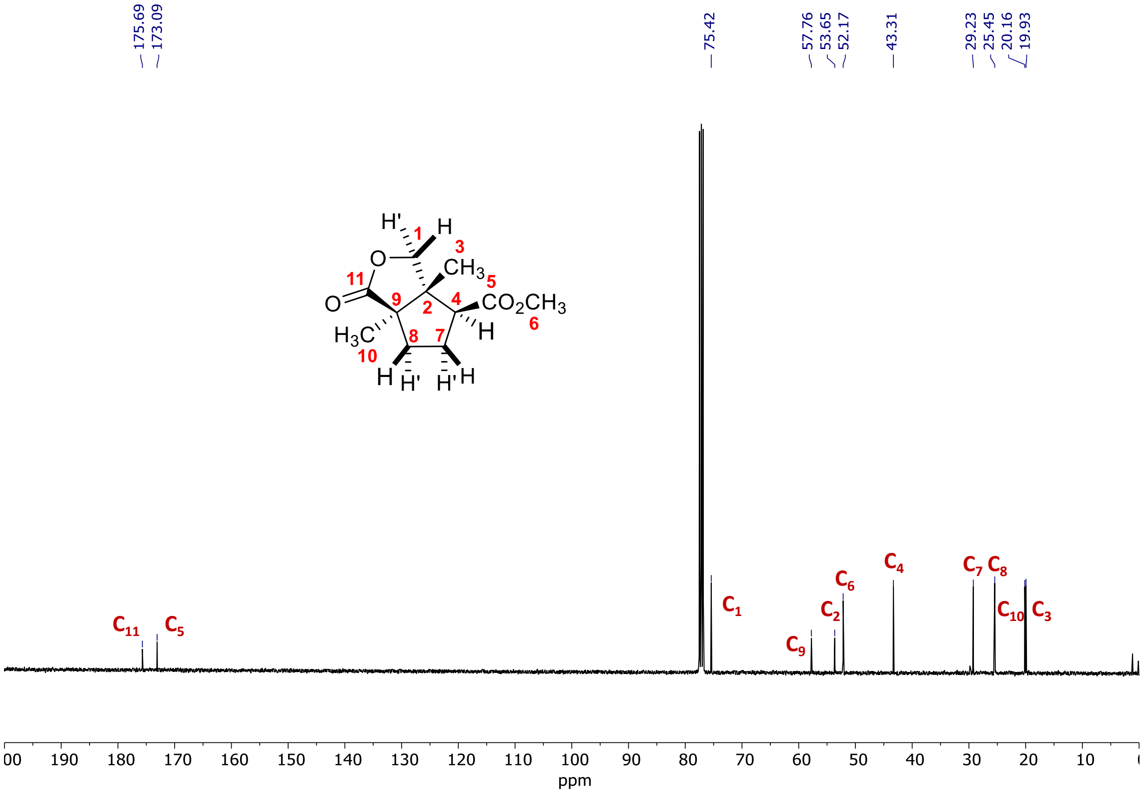

^{1}H-^{1}H COSY of **16b** in $CDCl_3$

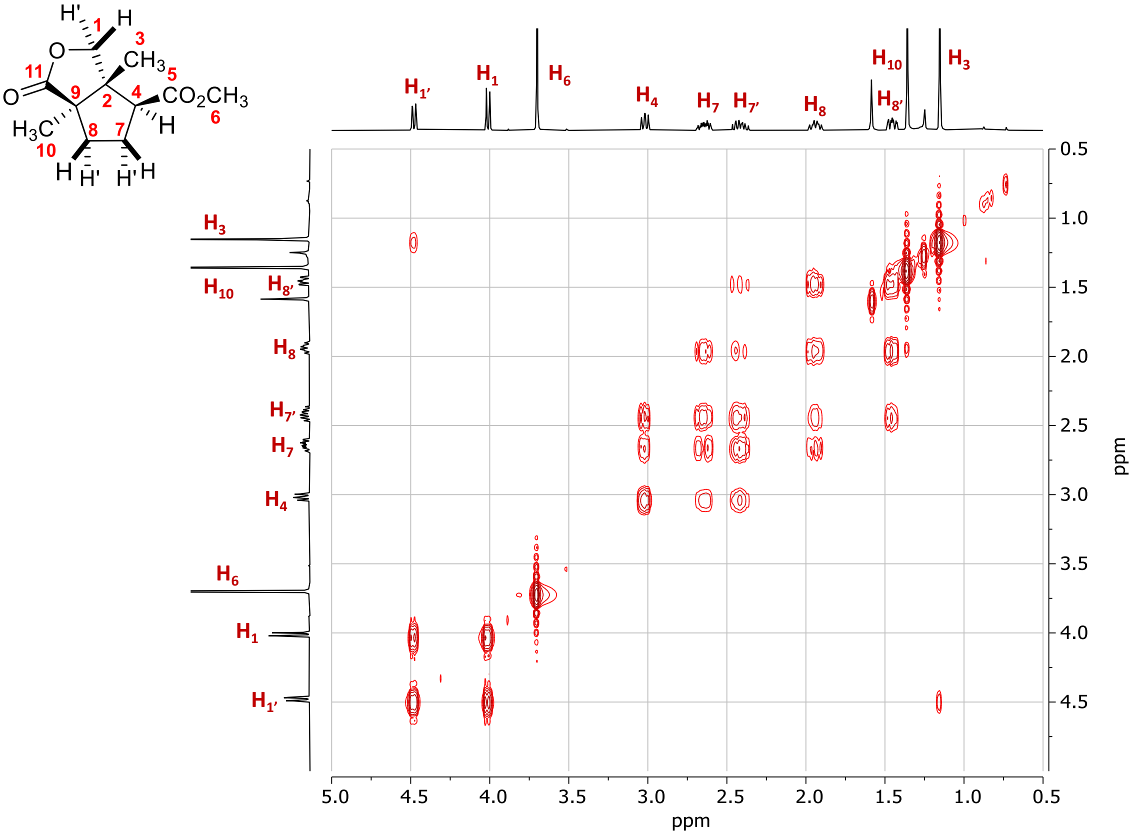

^{1}H-^{13}C HSQCed of **16b** in $CDCl_3$

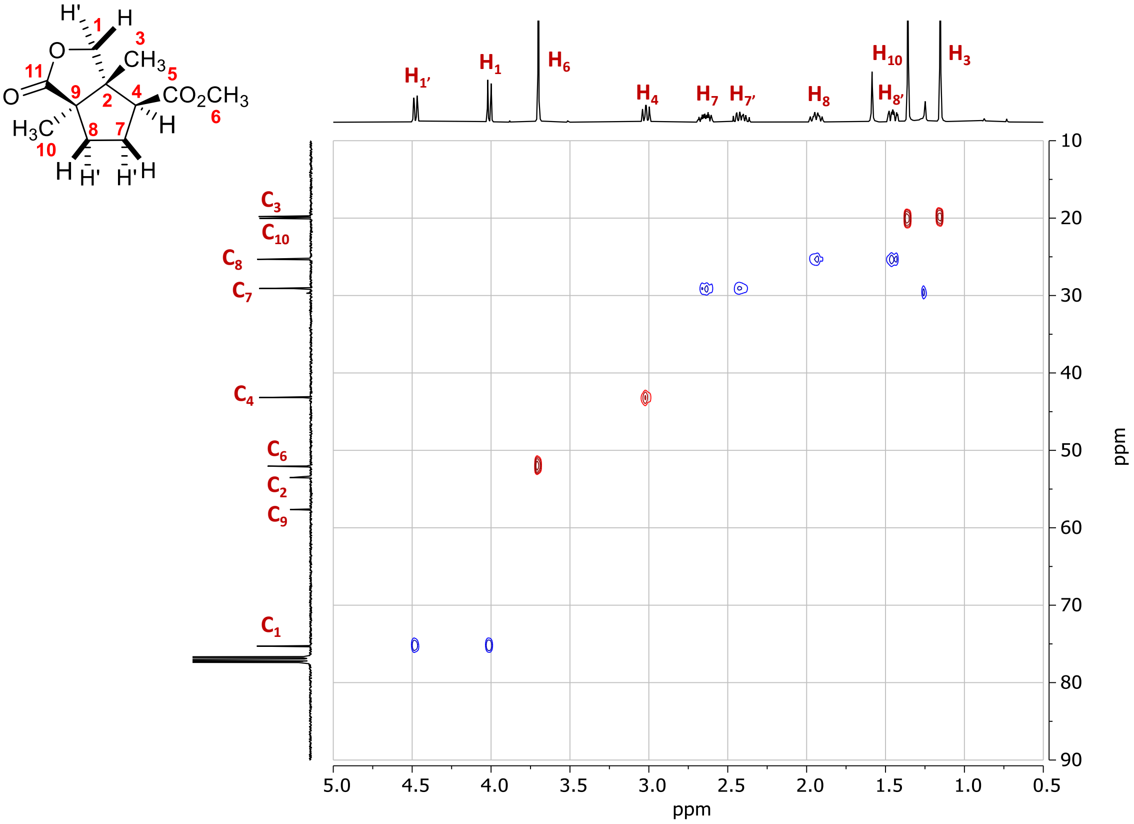

DEPTQ of **16b** in $CDCl_3$

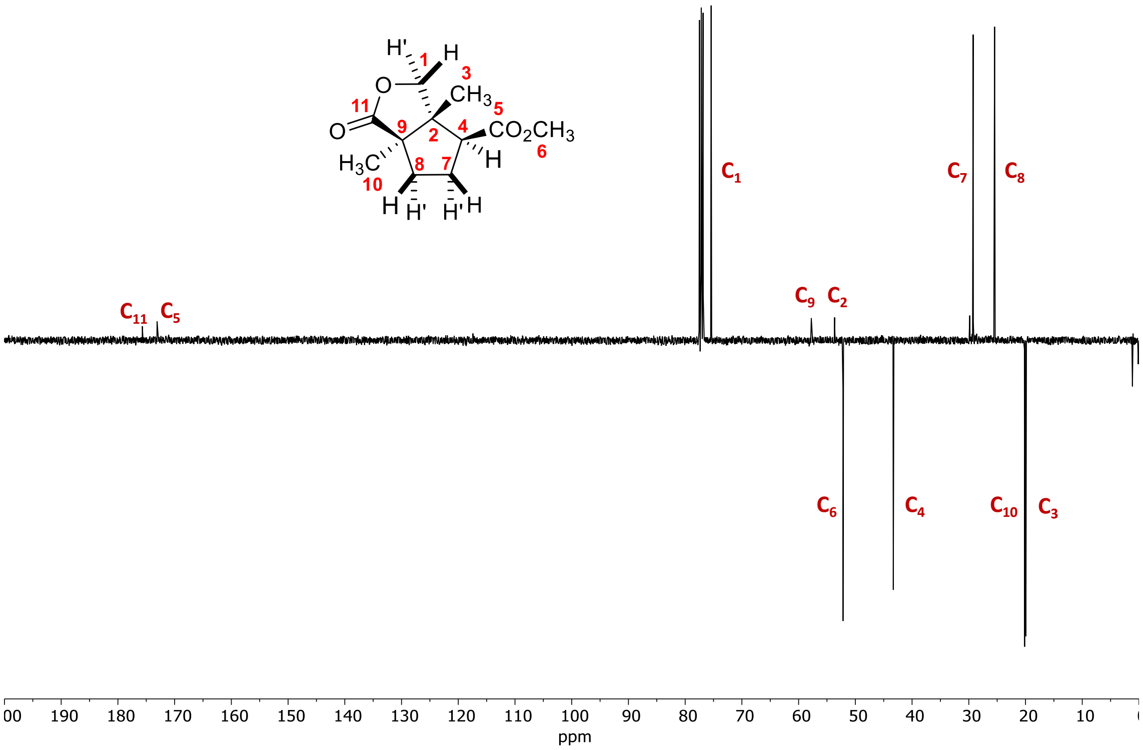

^1H-^{13}C HMBC of **16b** in $CDCl_3$

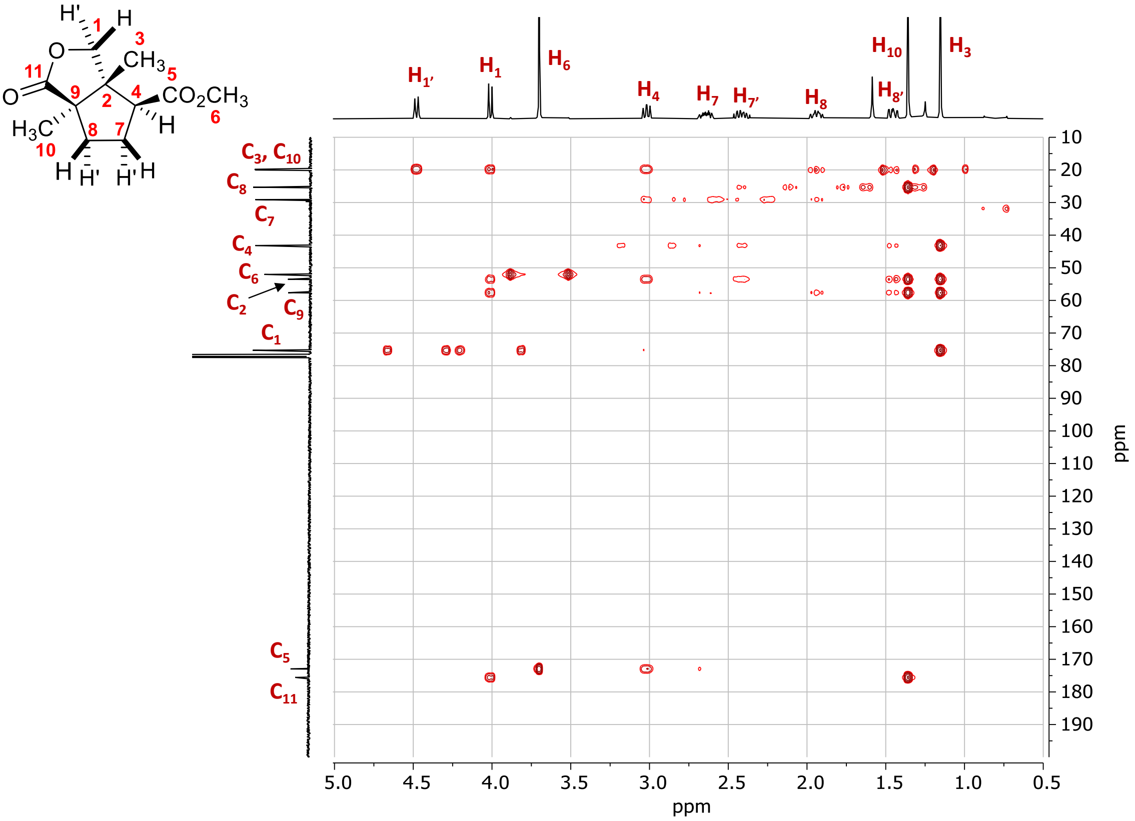

^{1}H-^{1}H TOCSY of **16b** in CDCl$_3$

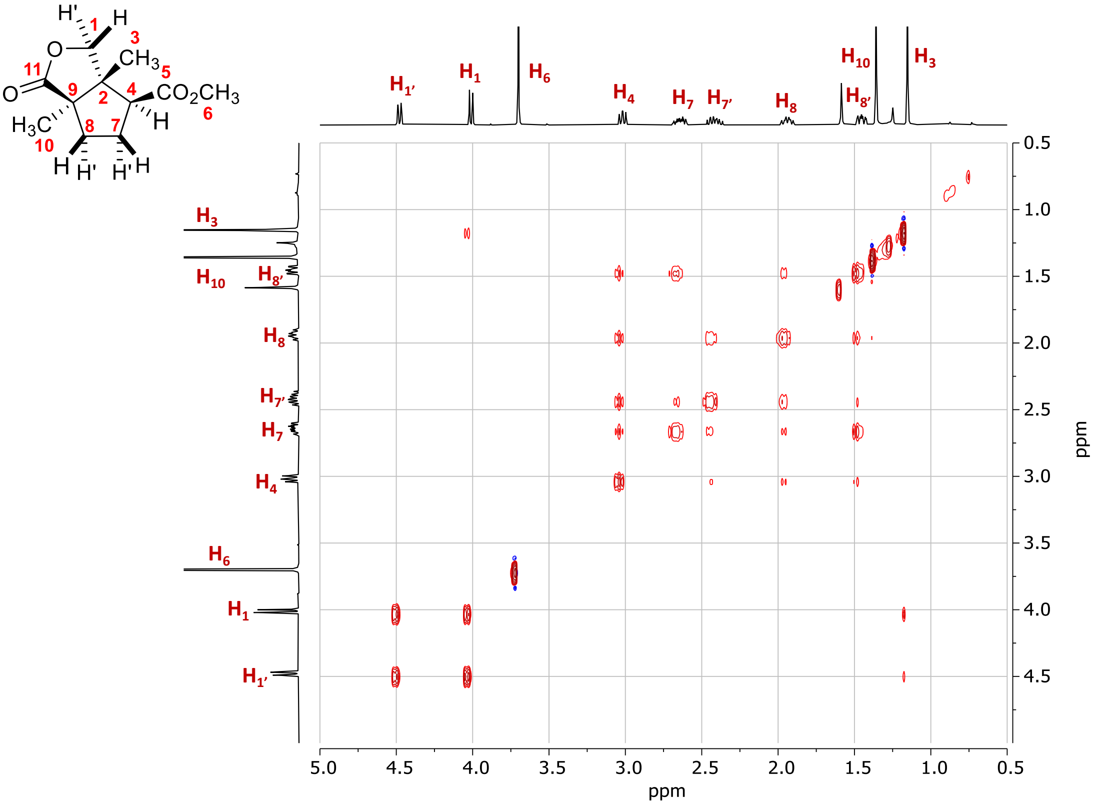

^{1}H-^{1}H NOESY of **16b** in CDCl$_3$

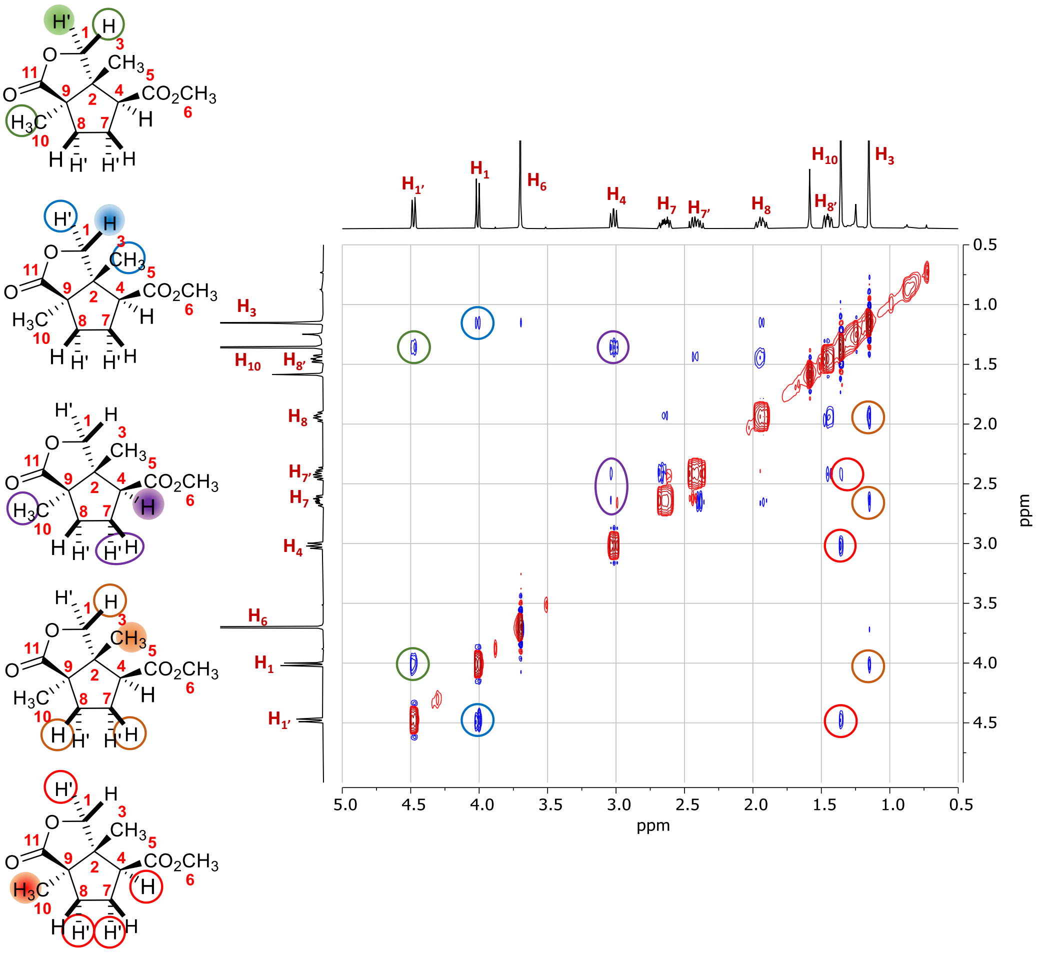

Selective NOESY experiments of **16b** in $CDCl_3$

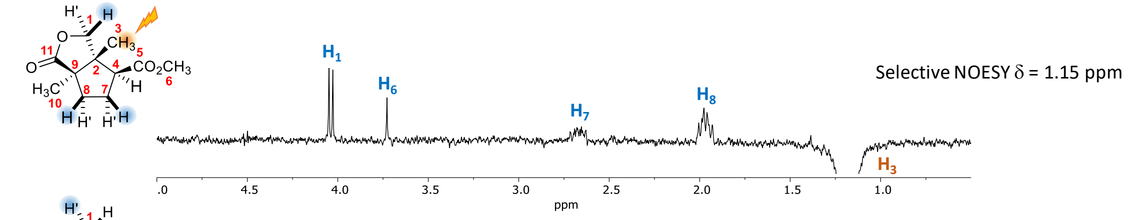

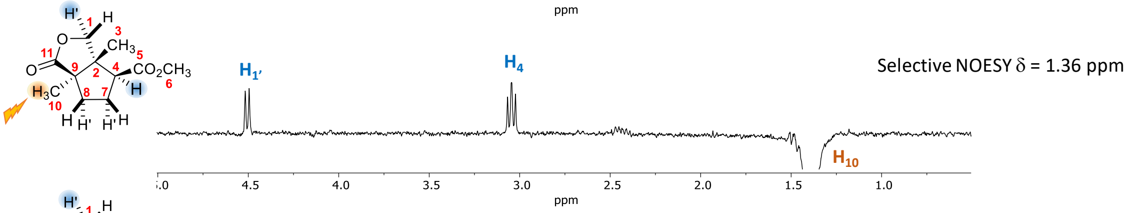

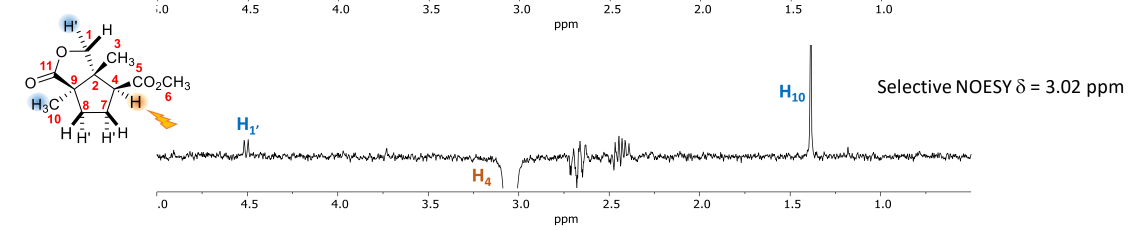

^{1}H-NMR of **16c** in $CDCl_3$

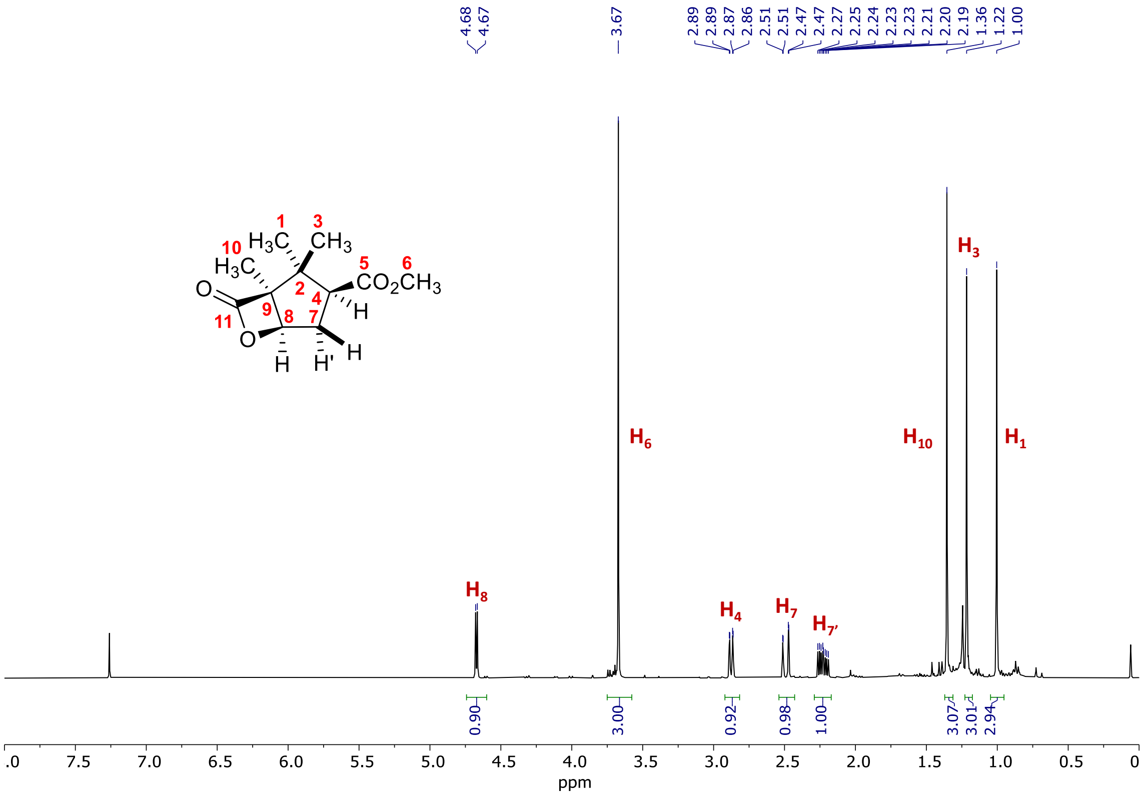

^{13}C-NMR of **16c** in $CDCl_3$

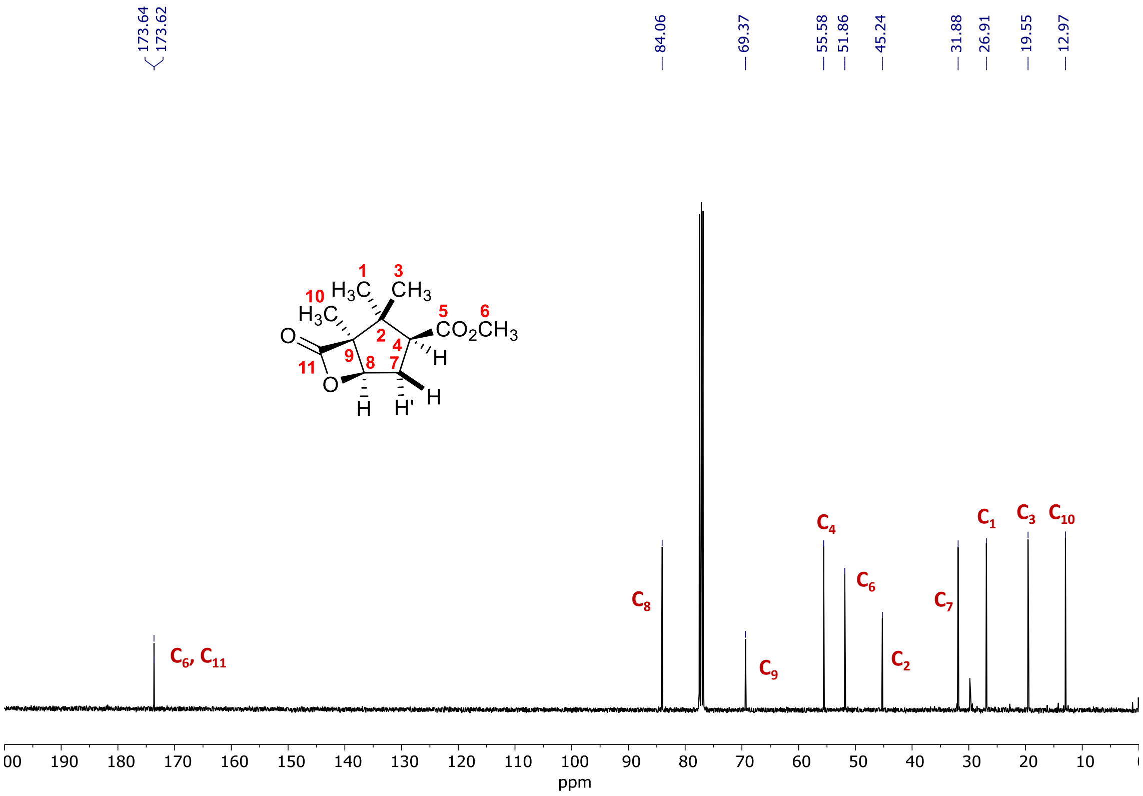

^1H-^1H COSY of **16c** in $CDCl_3$

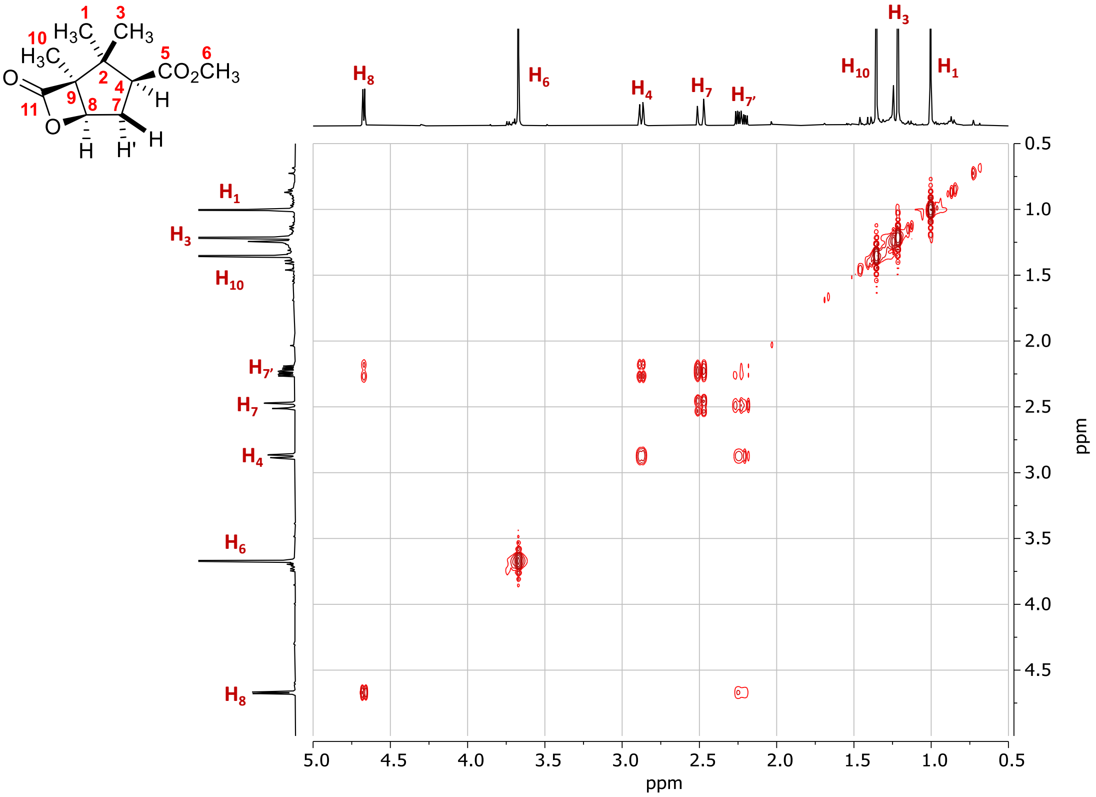

^1H-^{13}C HSQCed of **16c** in $CDCl_3$

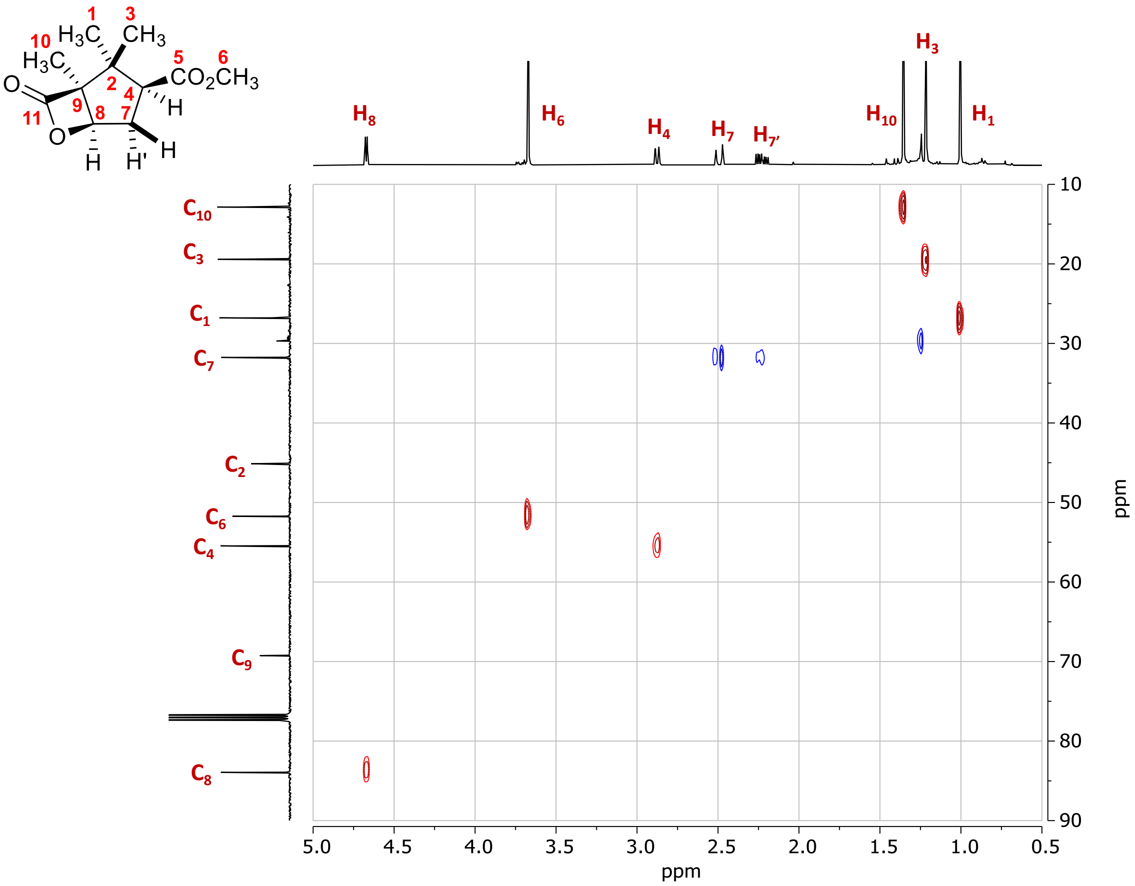

DEPTQ of **16c** in $CDCl_3$

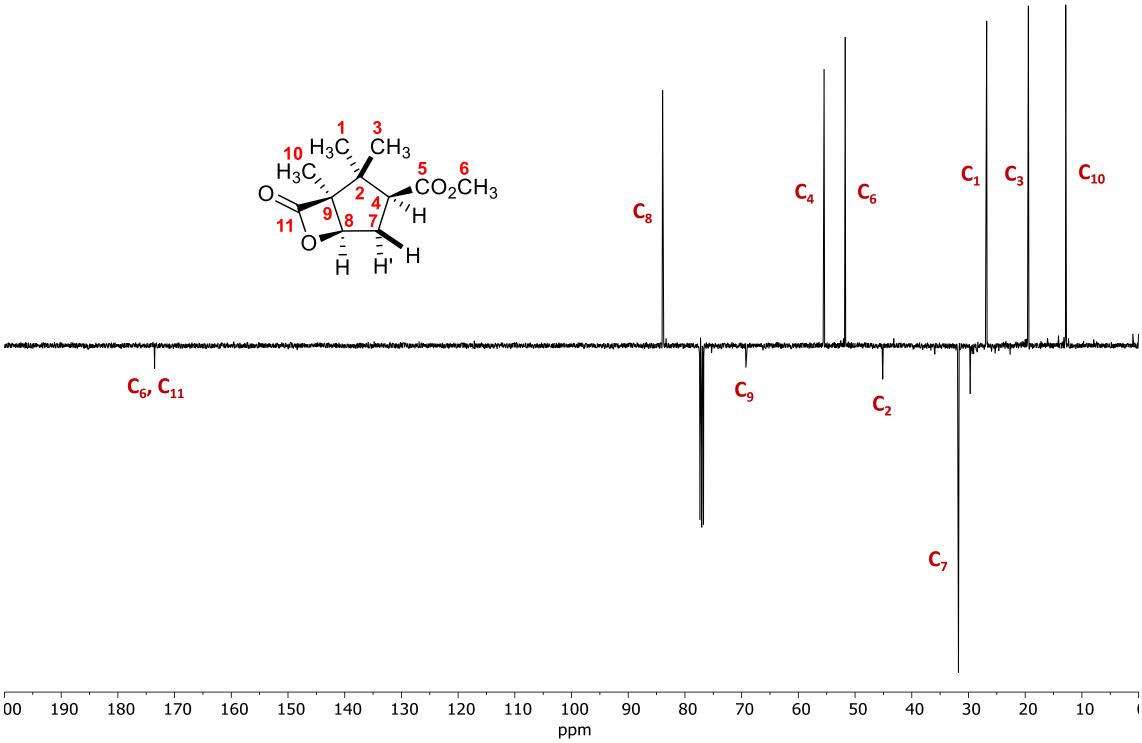

^{1}H-^{13}C HMBC of **16c** in $CDCl_3$

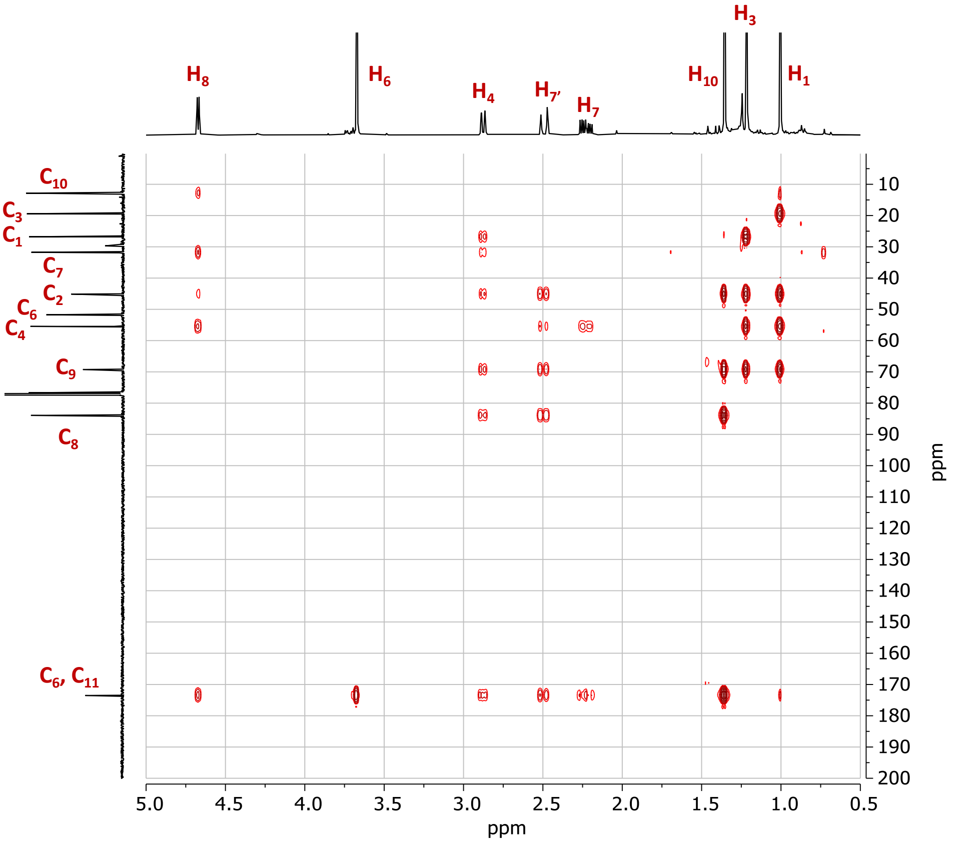

^{1}H-^{1}H TOCSY of **16c** in $CDCl_3$

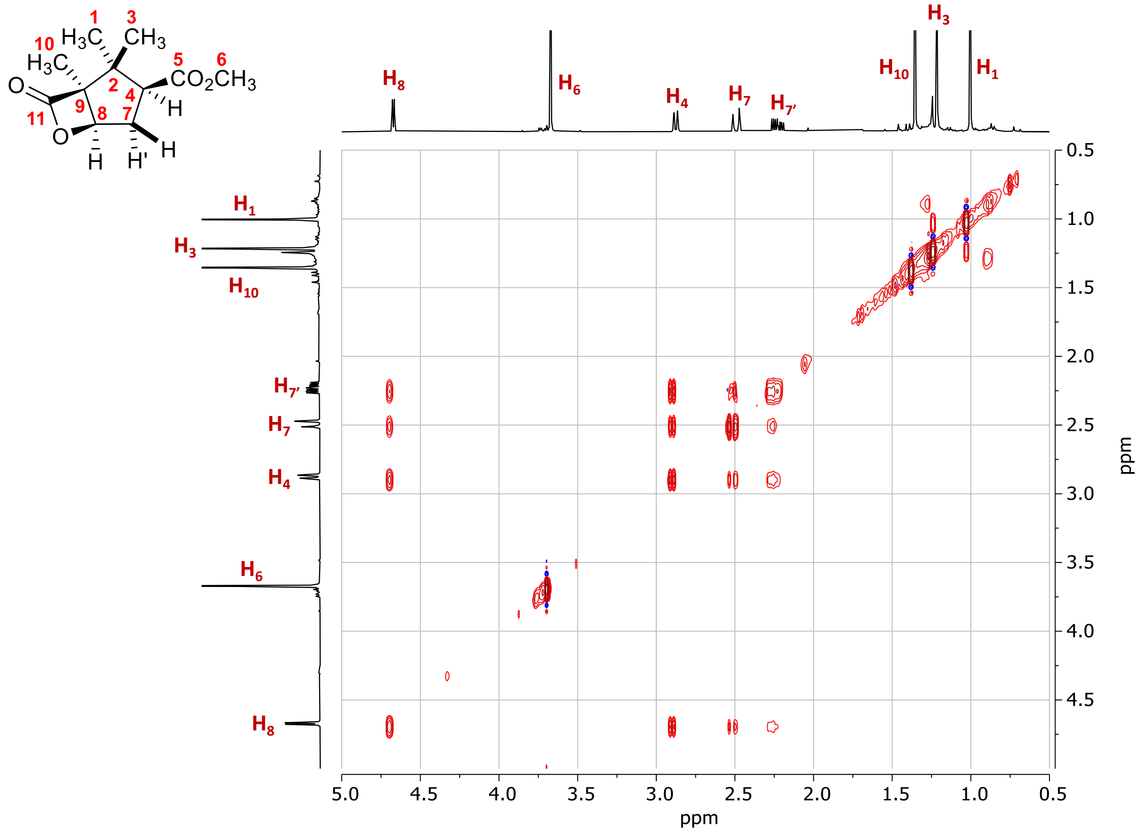

^{1}H-^{1}H NOESY of **16c** in $CDCl_3$

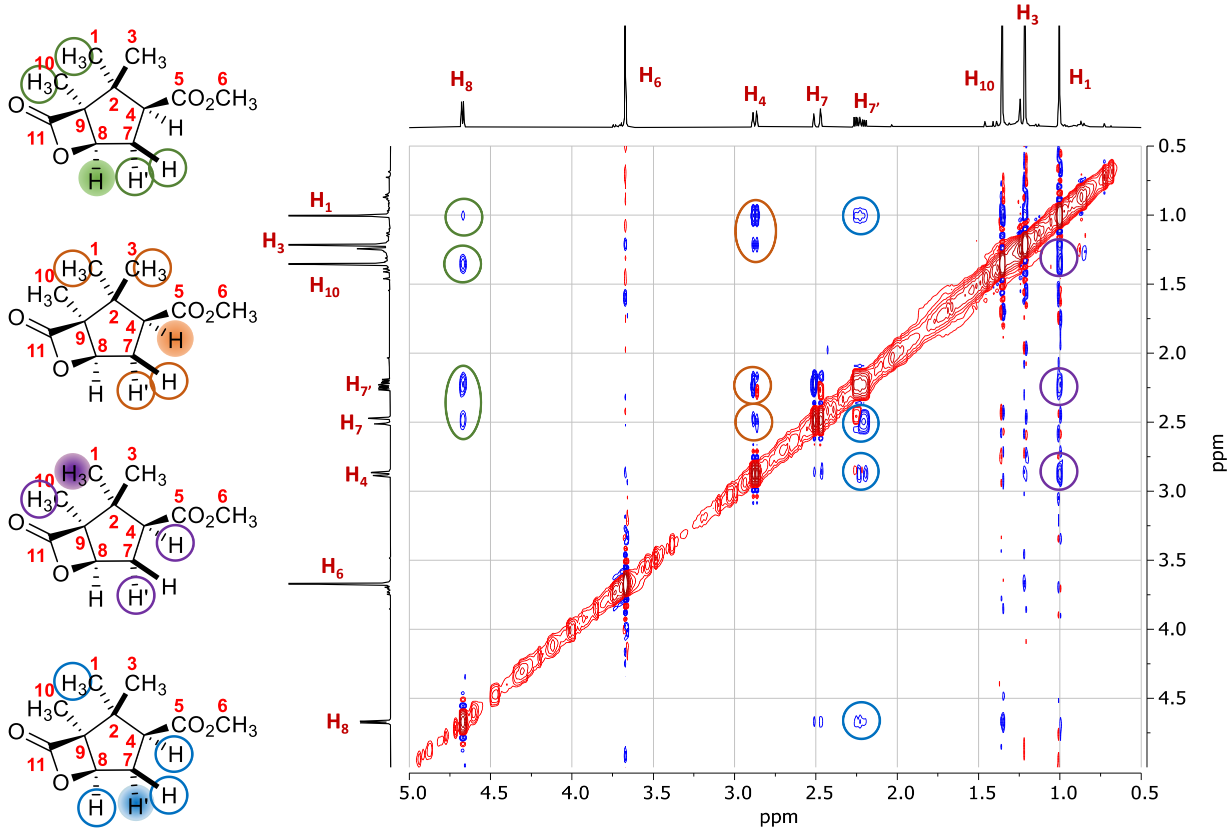

Selective NOESY experiments of **16c** in $CDCl_3$

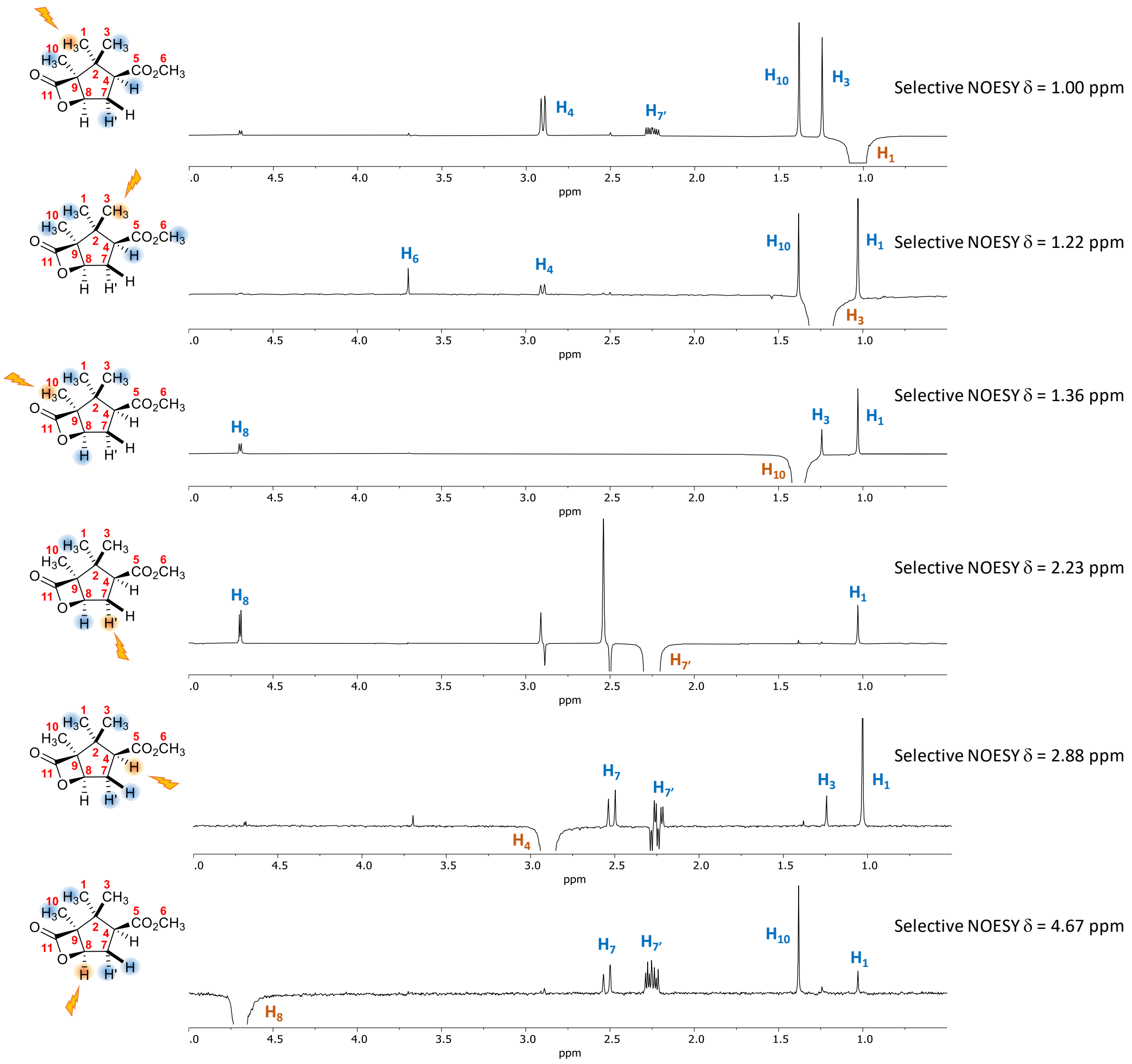

^{1}H-NMR of **17a** in $CDCl_3$

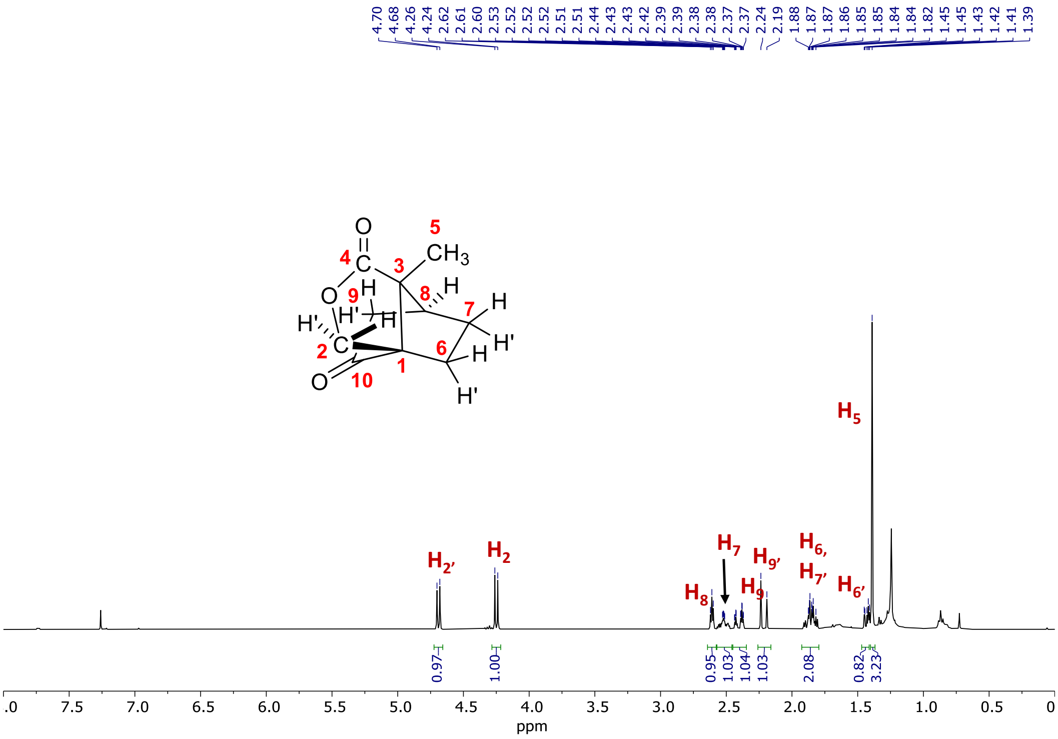

^{13}C-NMR of **17a** in $CDCl_3$

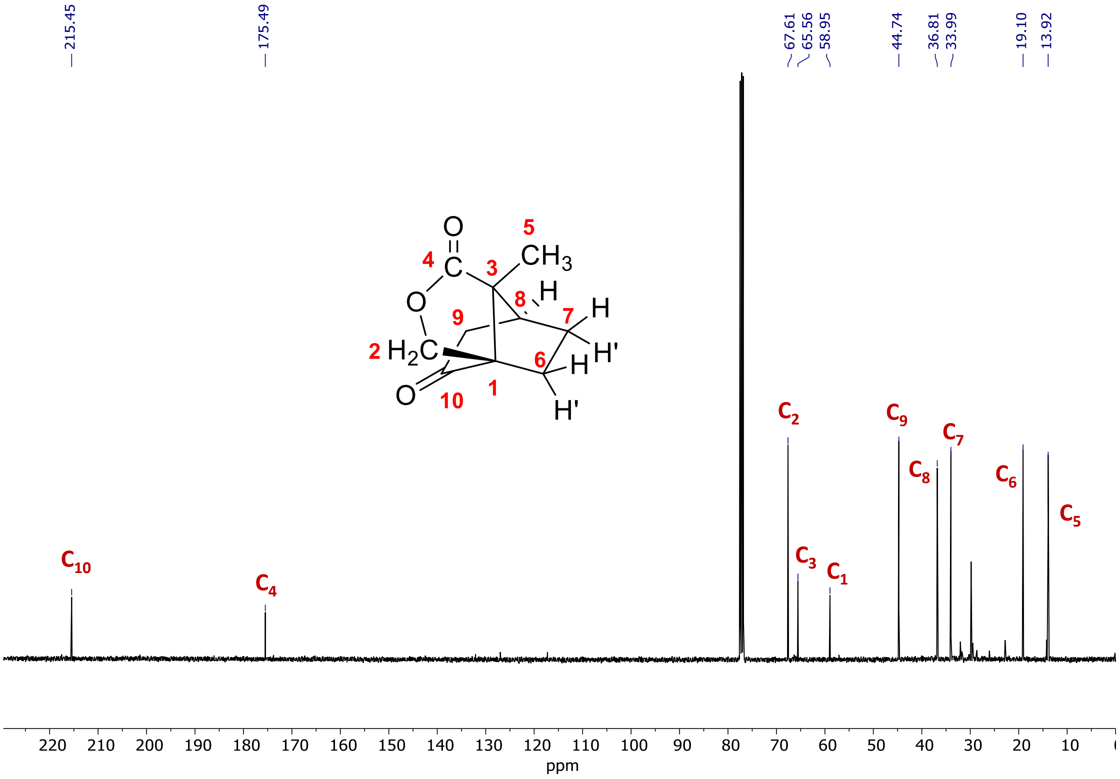

^{1}H-^{1}H COSY of **17a** in $CDCl_3$

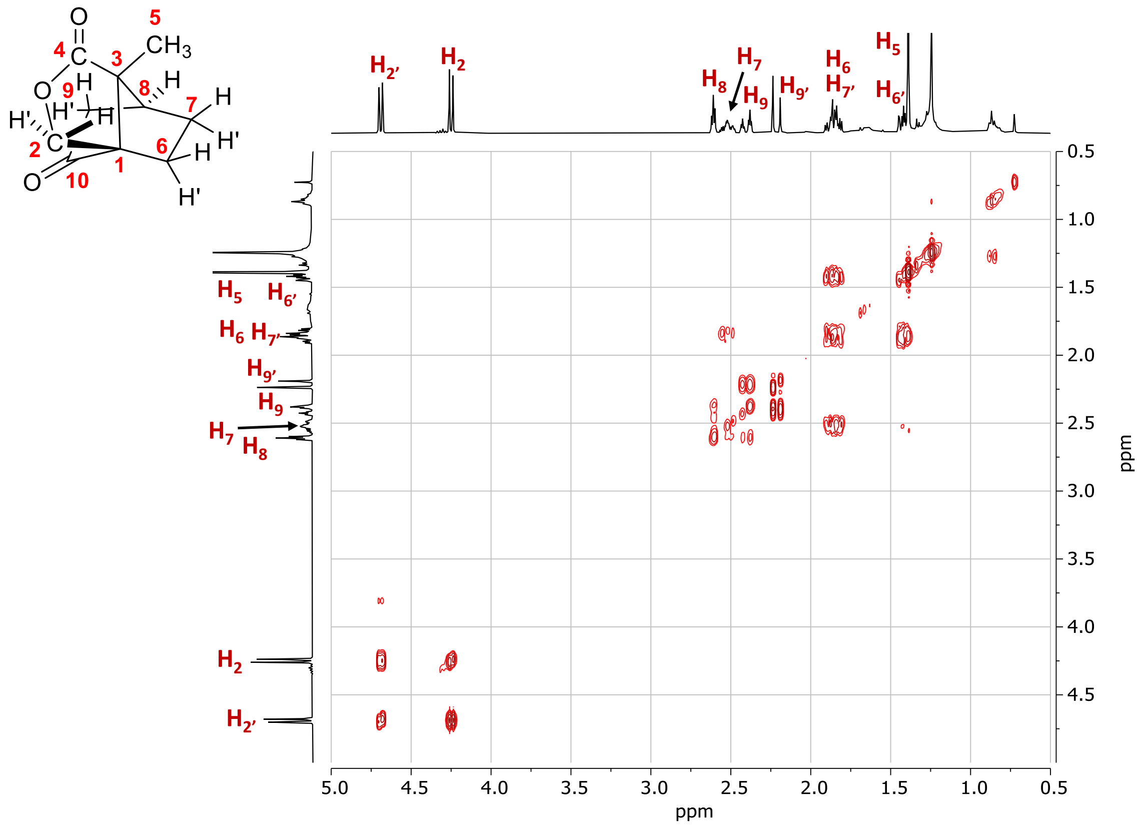

^{1}H-^{13}C HSQCed of **17a** in $CDCl_3$

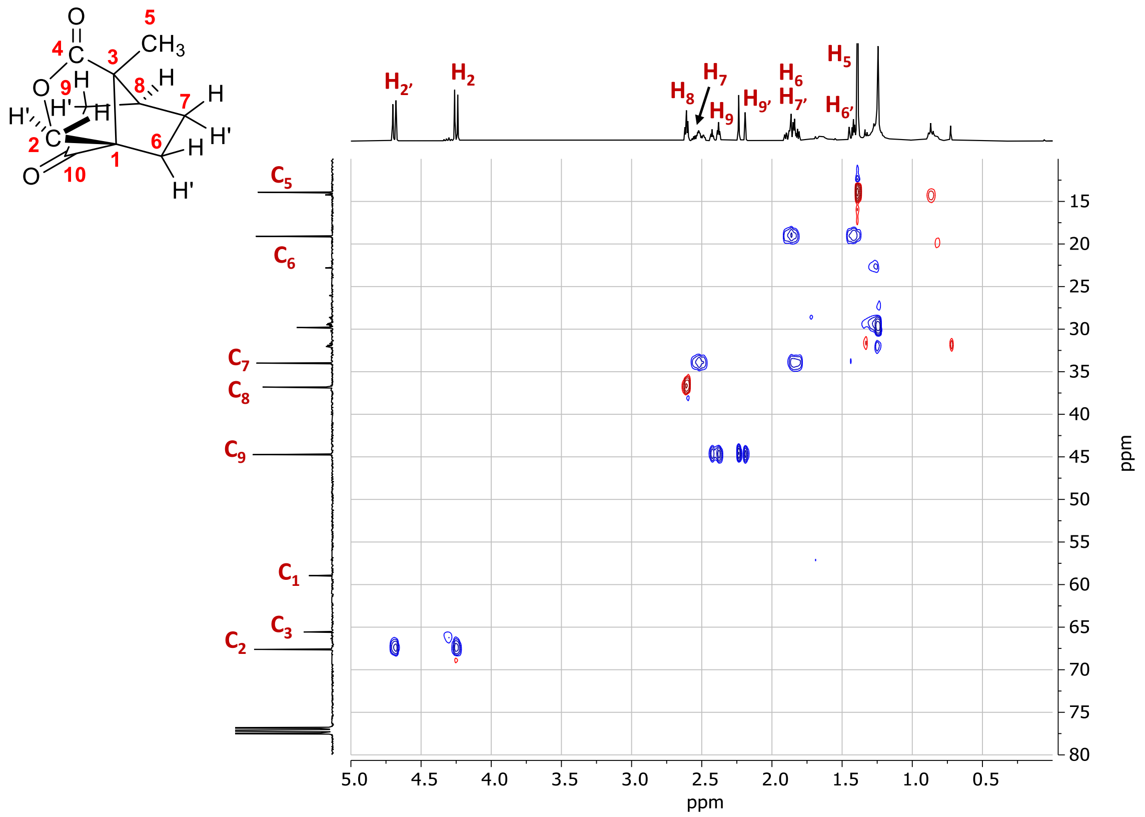

DEPTQ of **17a** in $CDCl_3$

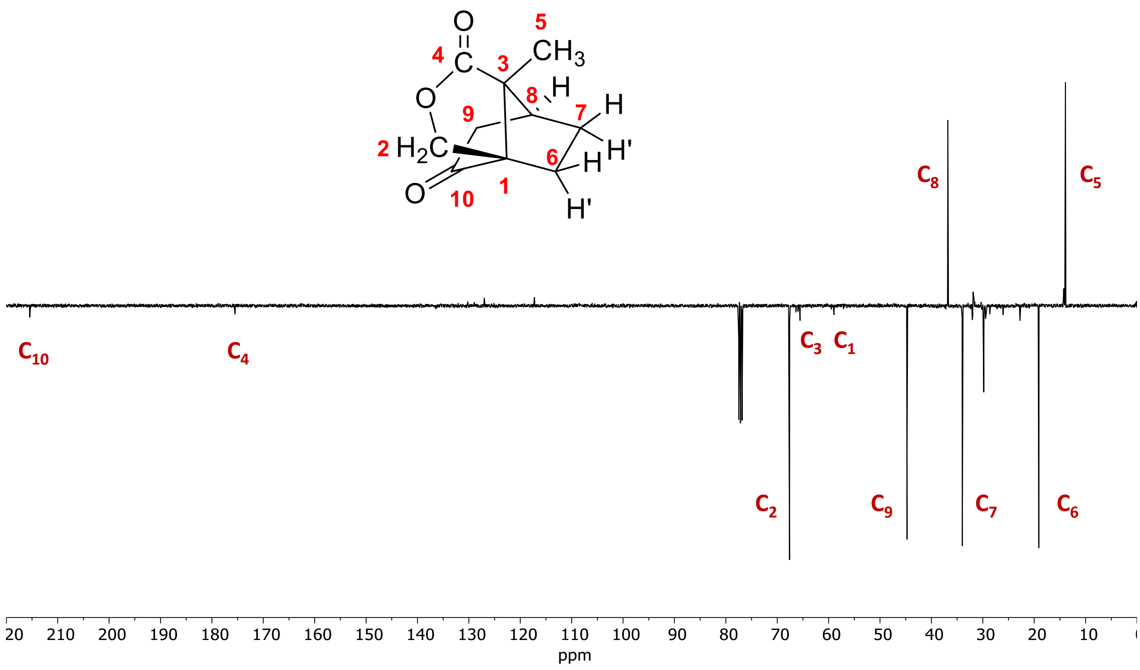

DEPT135 of **17a** in $CDCl_3$

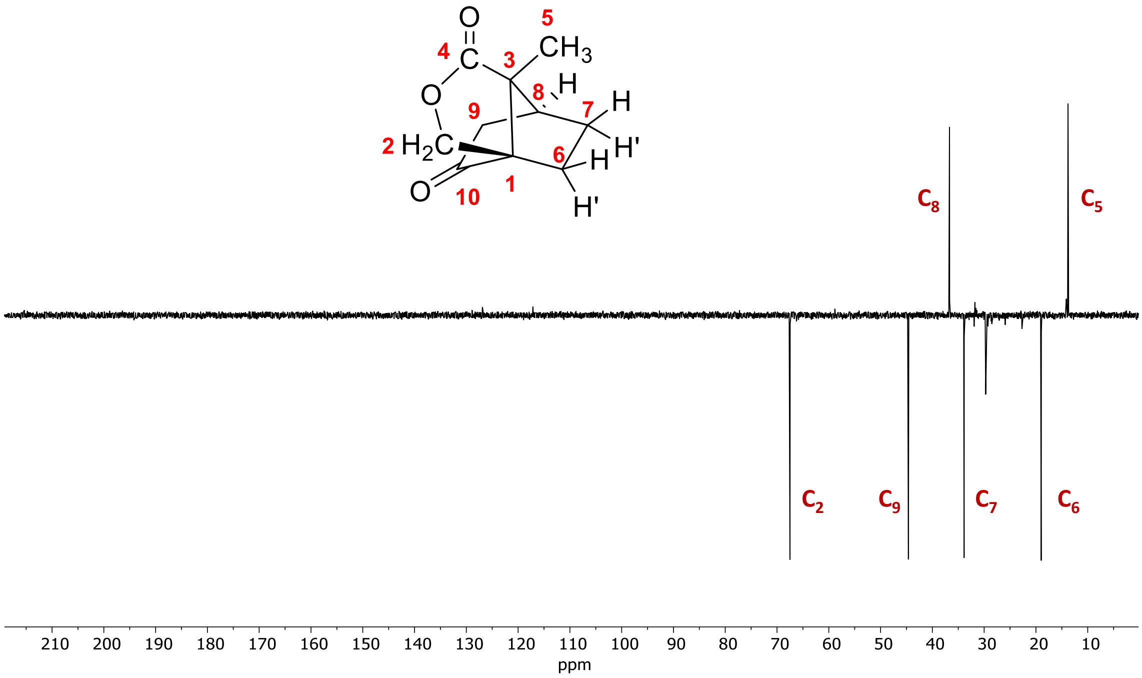

^{1}H-^{13}C HMBC of **17a** in CDCl$_3$

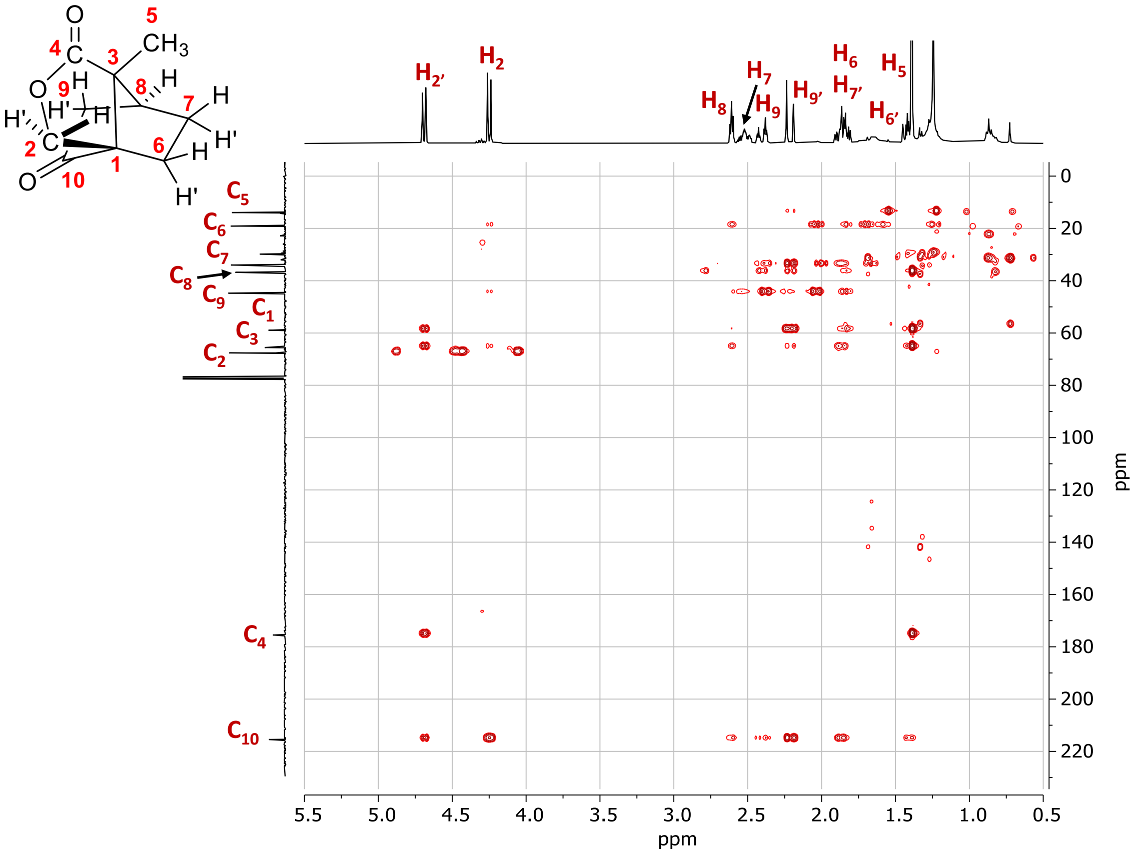

^{1}H-^{1}H TOCSY of **17a** in CDCl$_3$

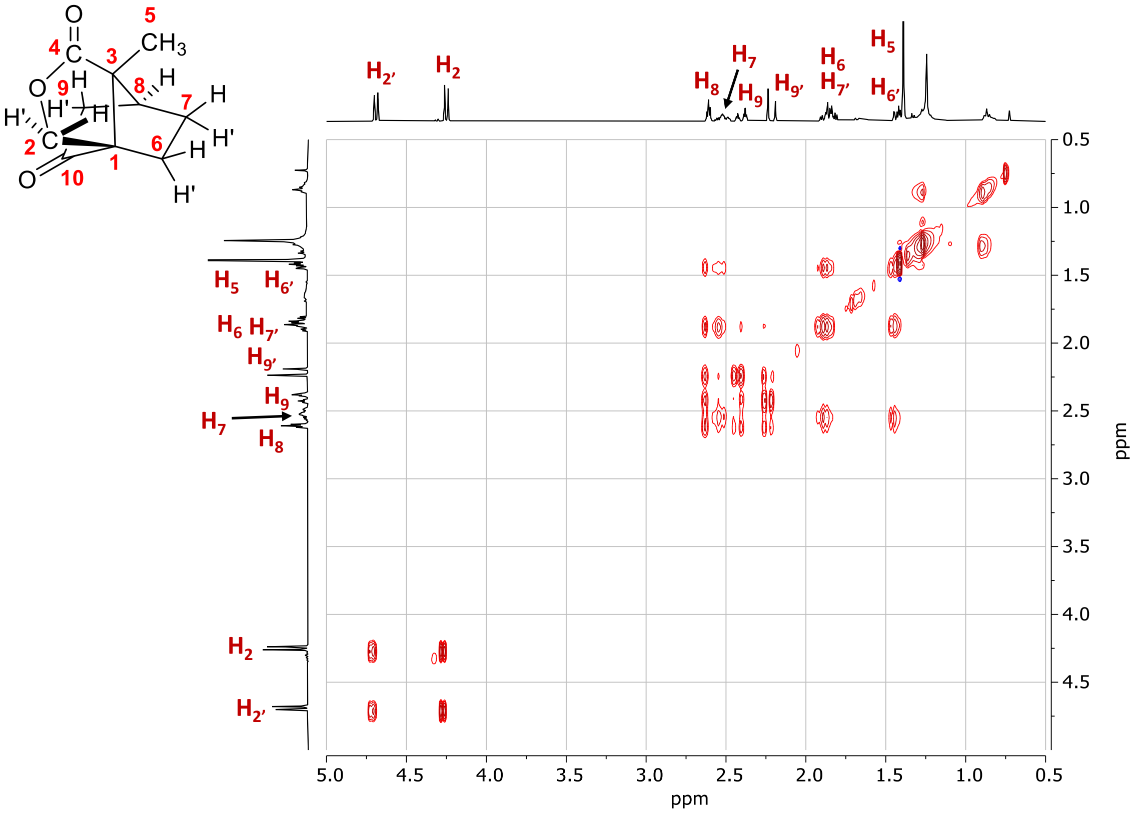

^1H-^1H NOESY of **17a** in $CDCl_3$

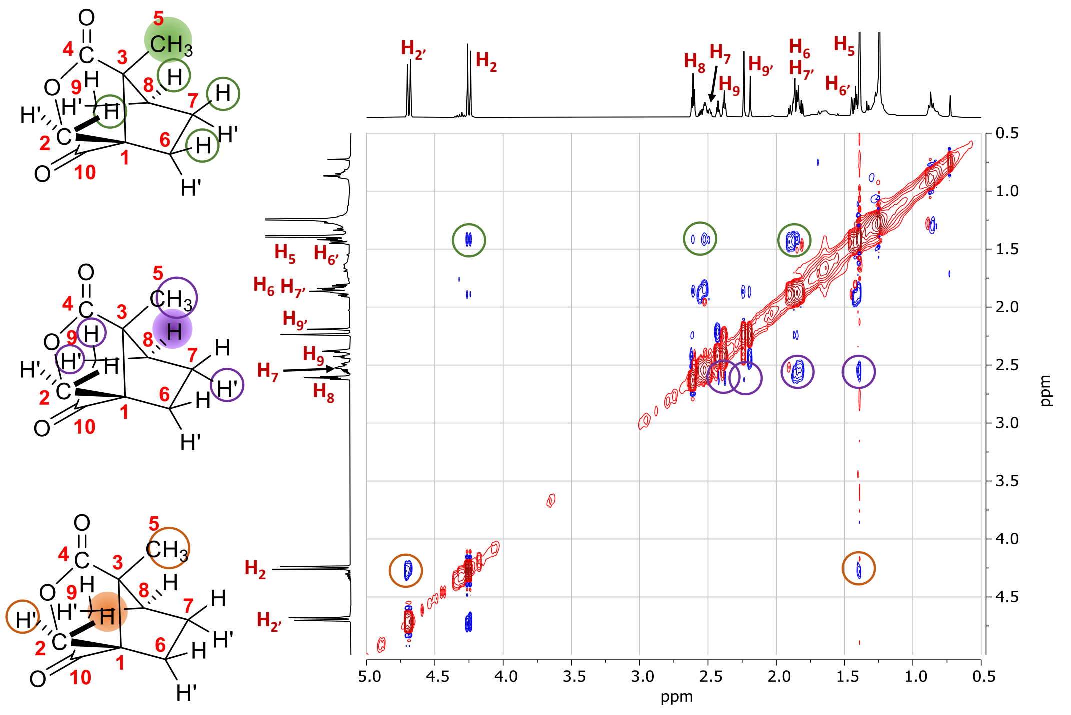

Selective NOESY experiments of **17a** in $CDCl_3$

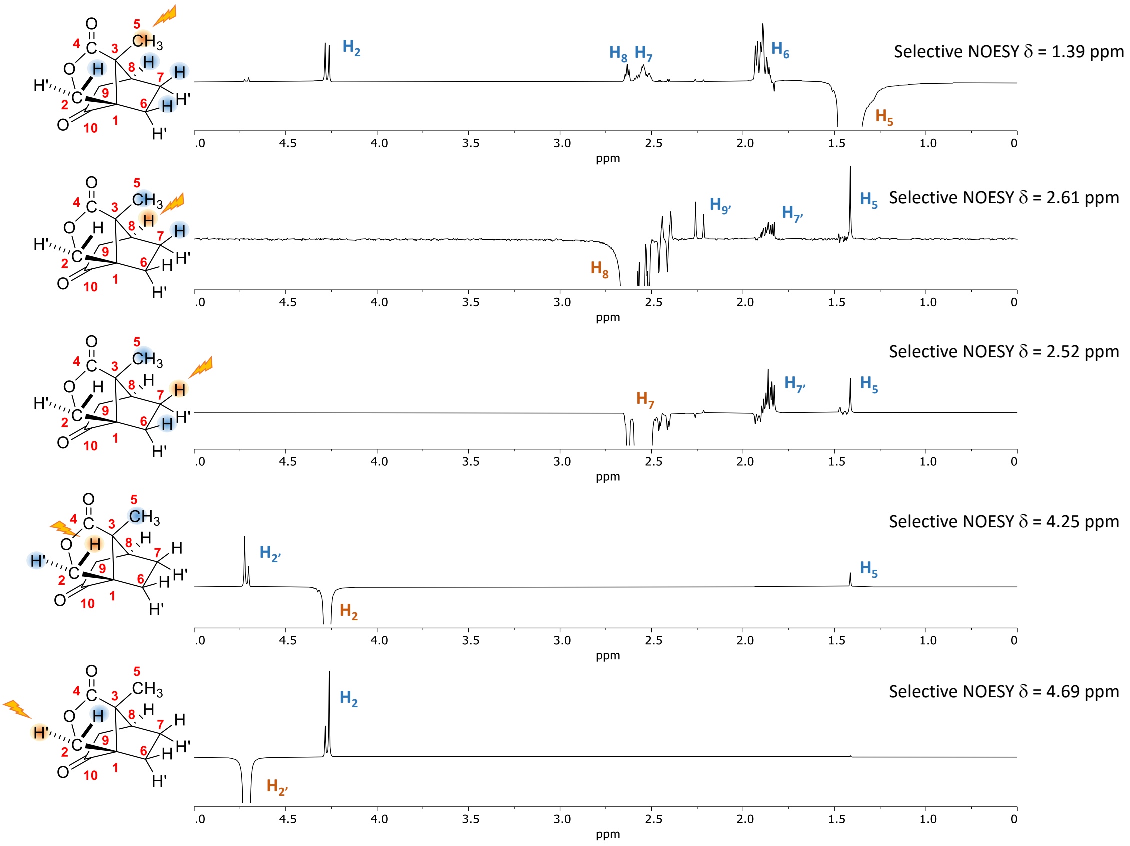

^{1}H-NMR of **17b** in $CDCl_3$

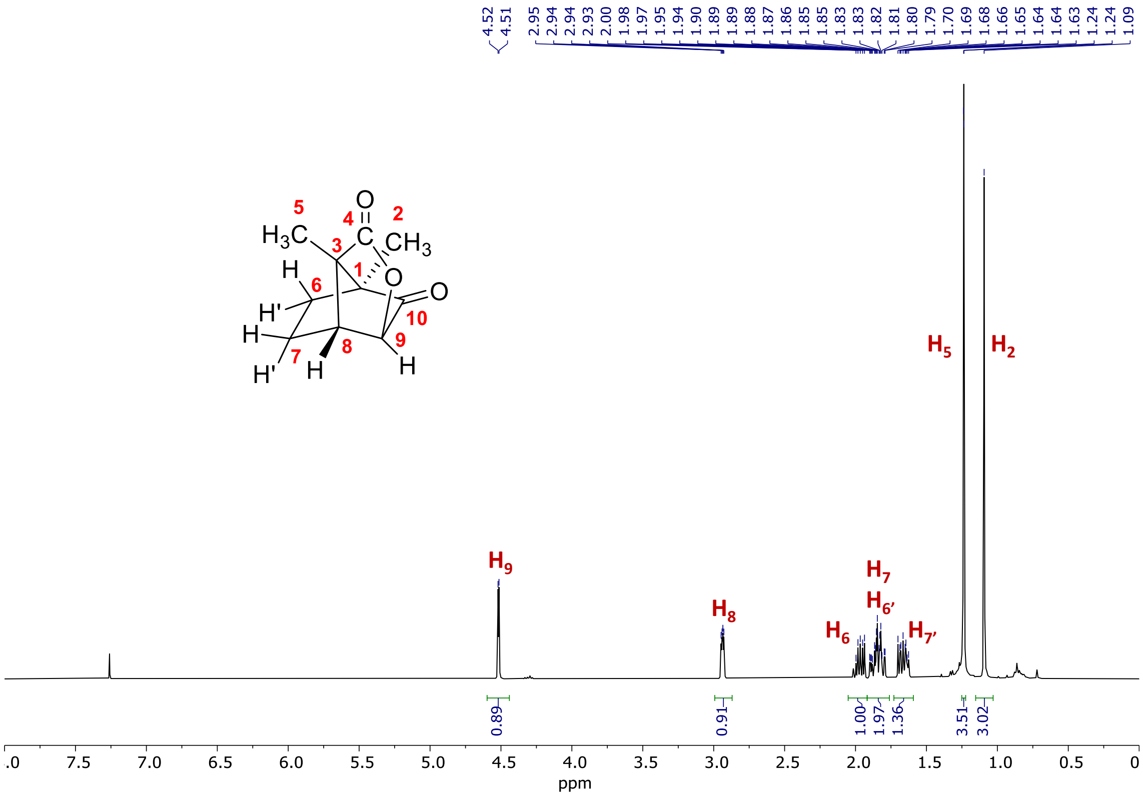

^{13}C-NMR of **17b** in $CDCl_3$

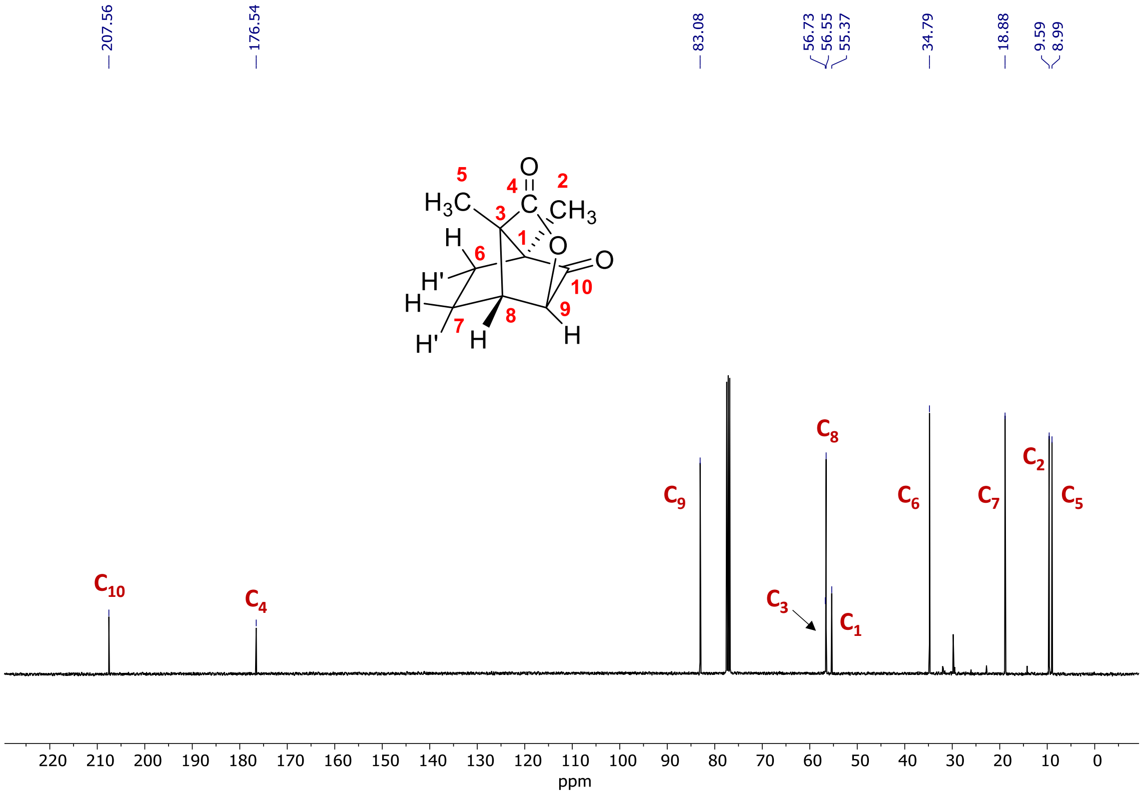

^{1}H-^{1}H COSY of **17b** in $CDCl_3$

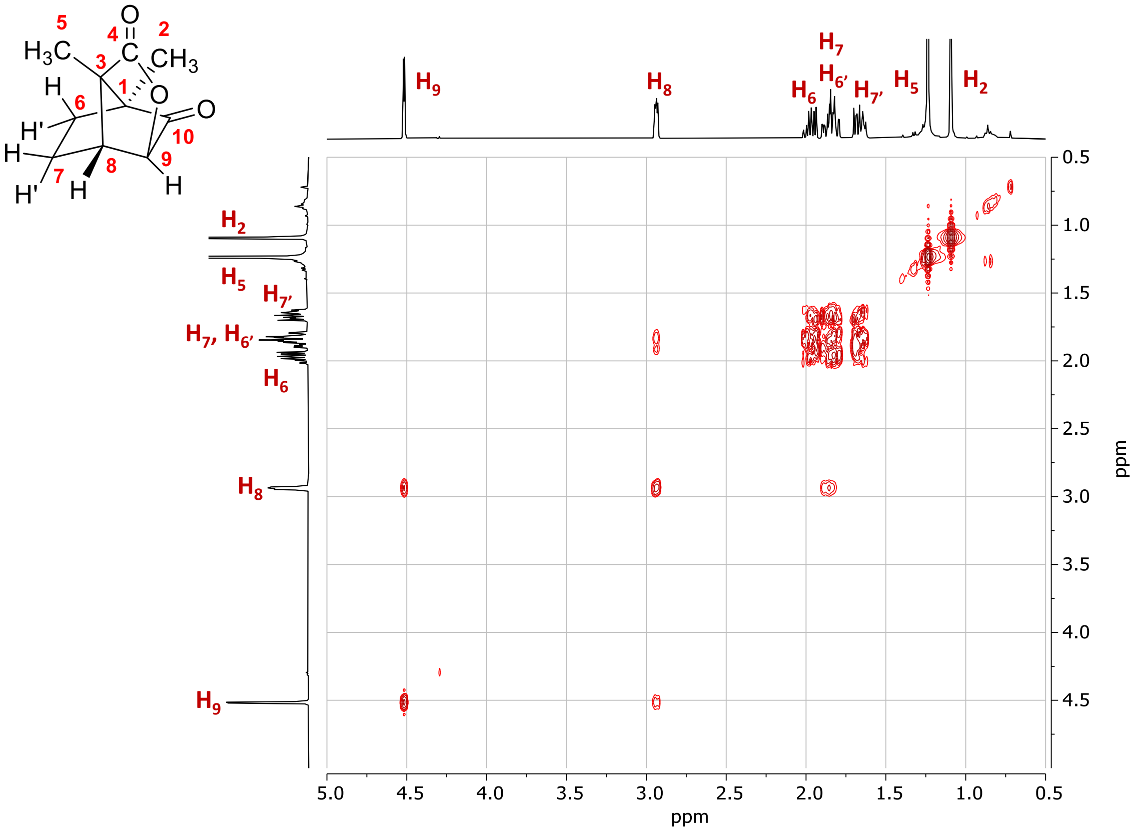

^{1}H-^{13}C HSQCed of **17b** in $CDCl_3$

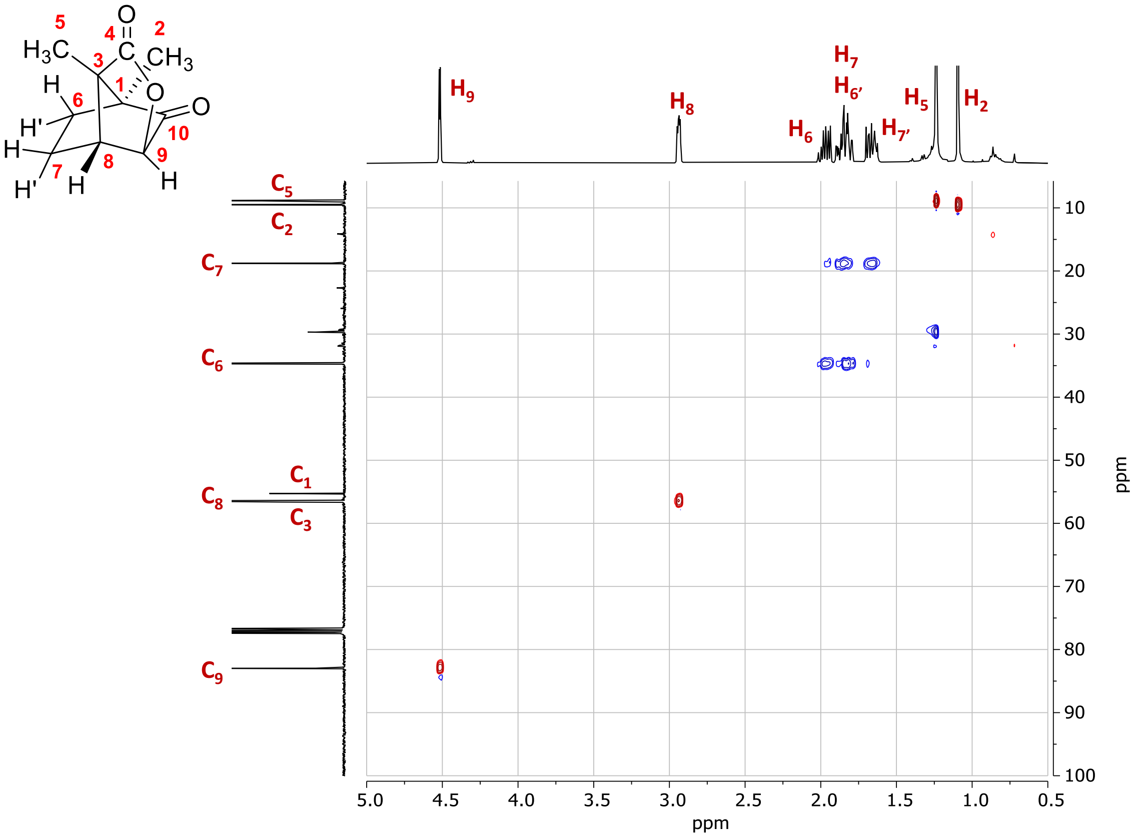

DEPTQ of **17b** in $CDCl_3$

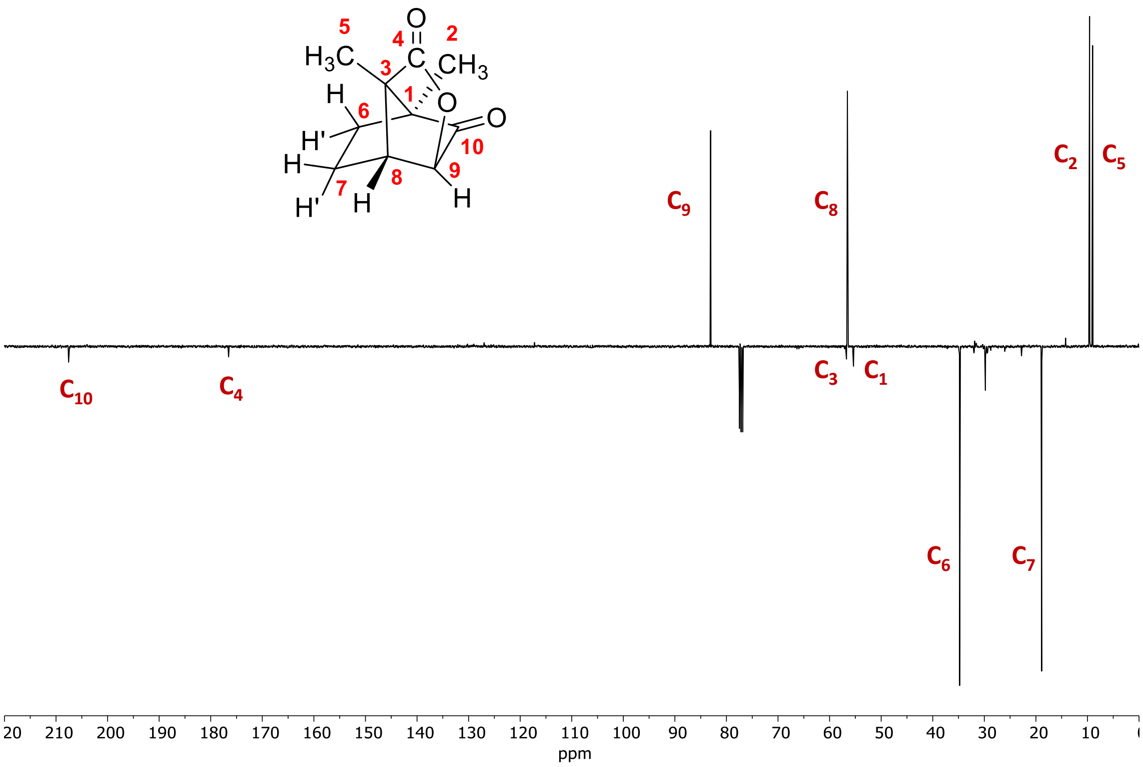

DEPT135 of **17b** in $CDCl_3$

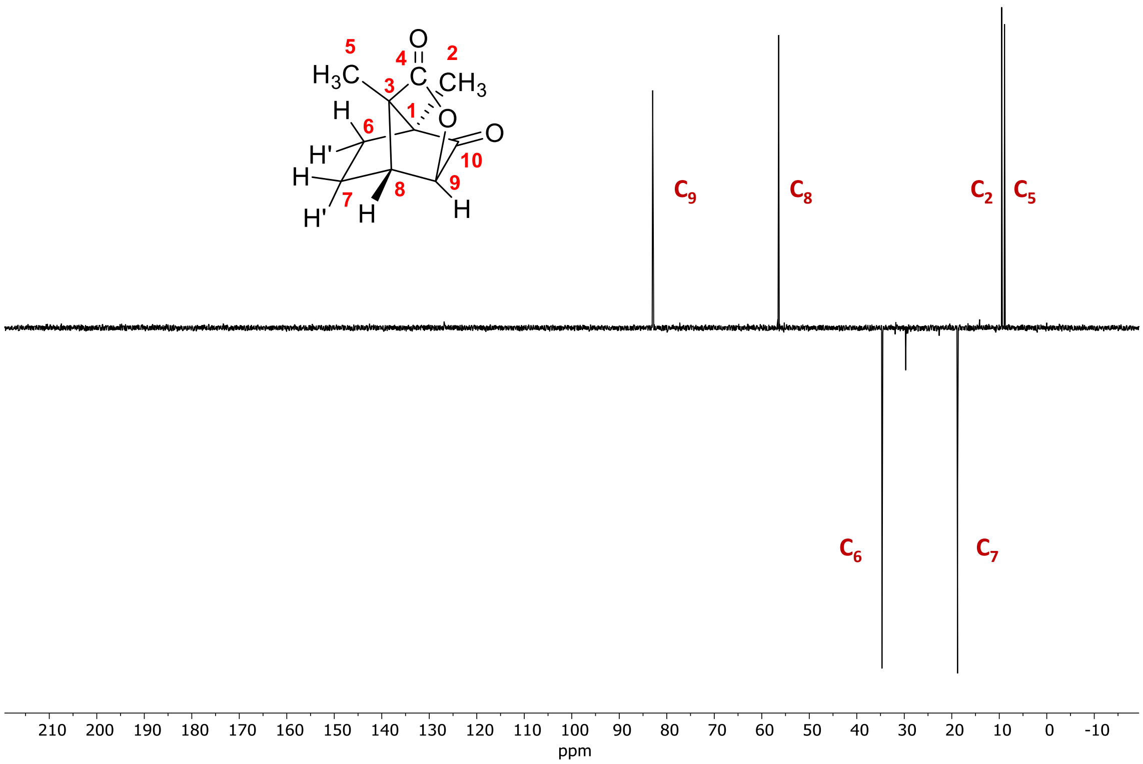

^{1}H-^{13}C HMBC of **17b** in $CDCl_3$

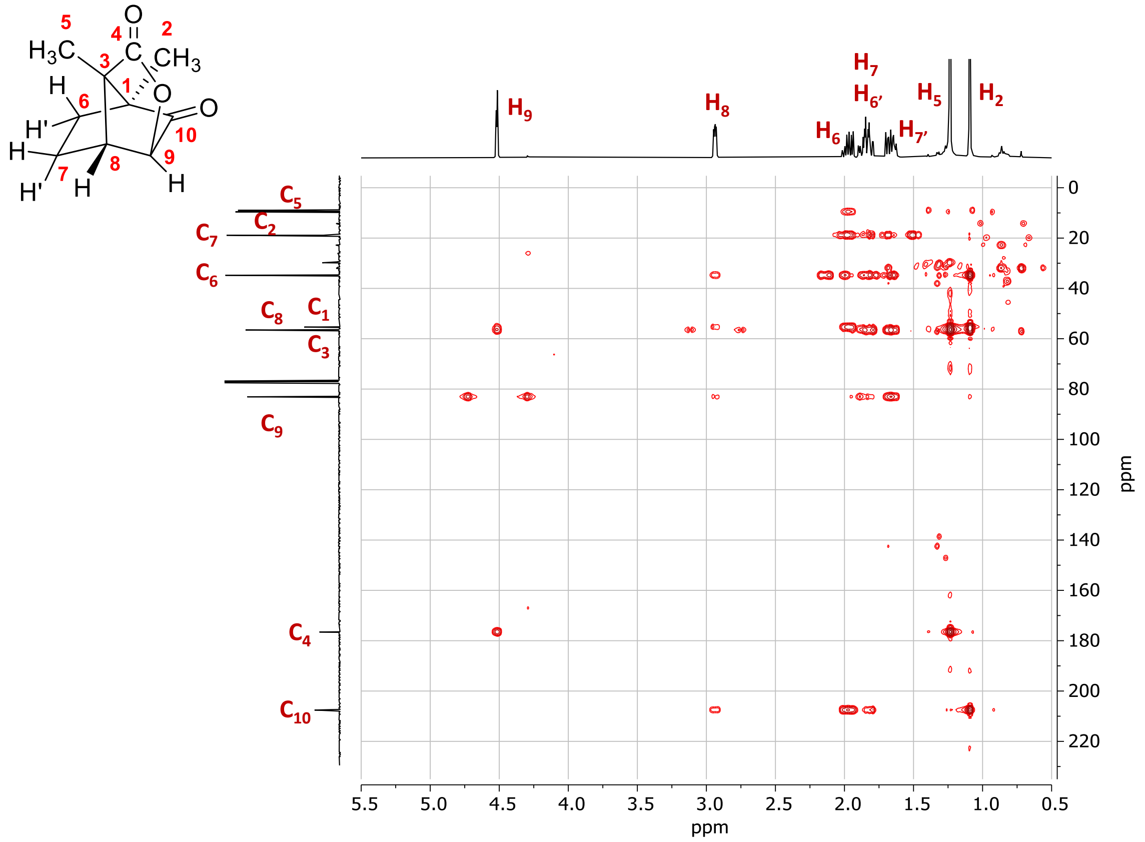

^{1}H-^{1}H TOCSY of **17b** in $CDCl_3$

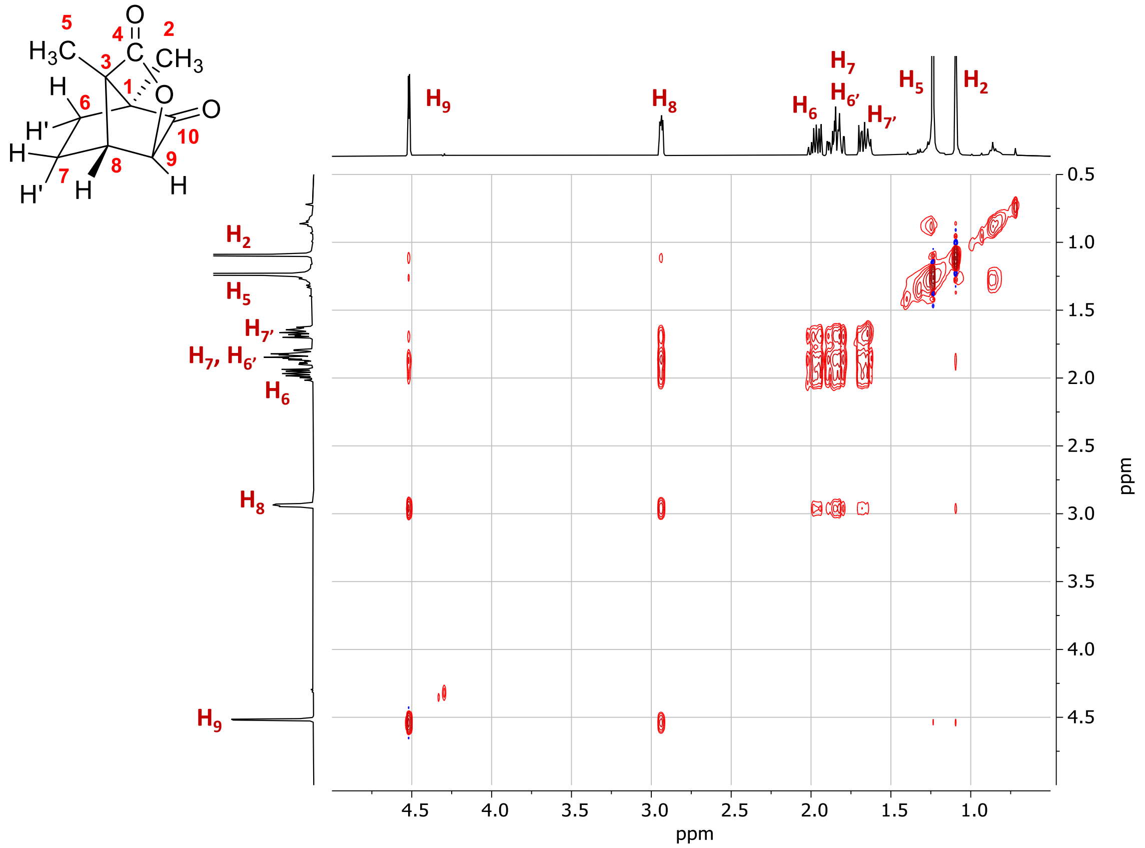

^{1}H-^{1}H NOESY of **17b** in $CDCl_3$

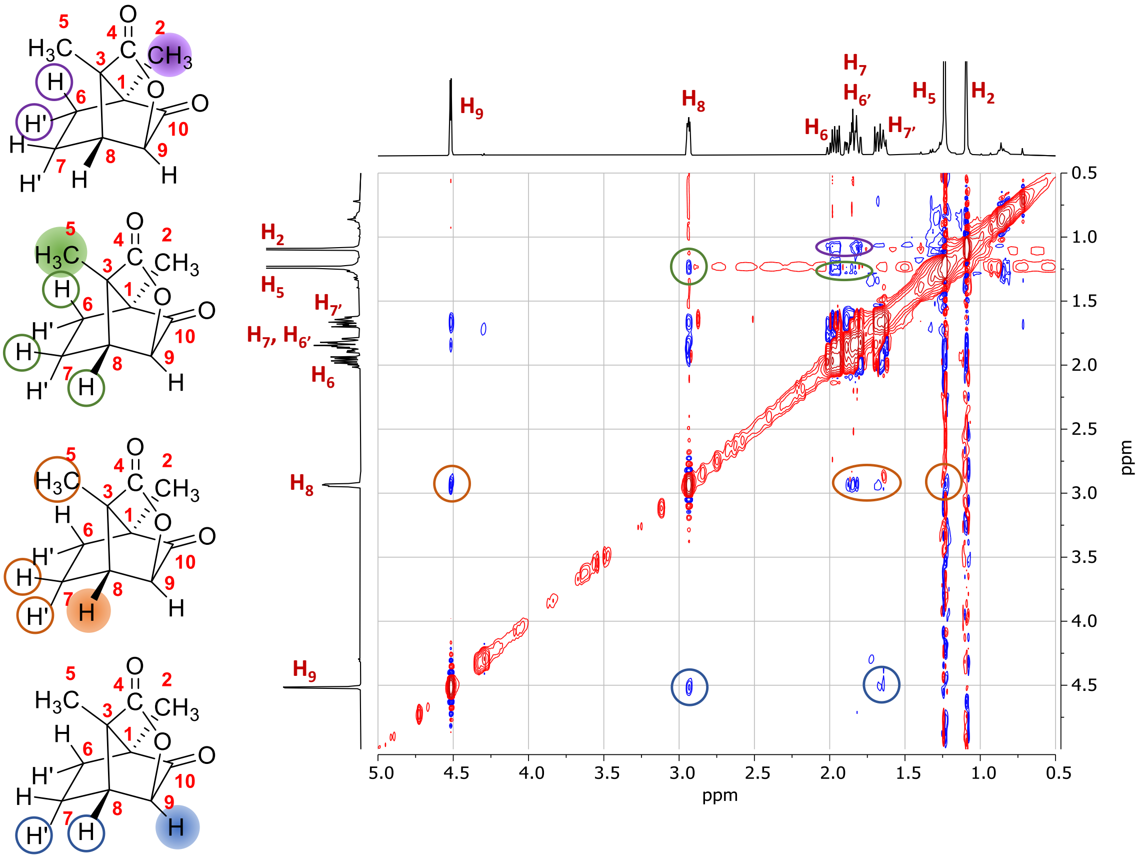

Selective NOESY experiments of **17b** in $CDCl_3$

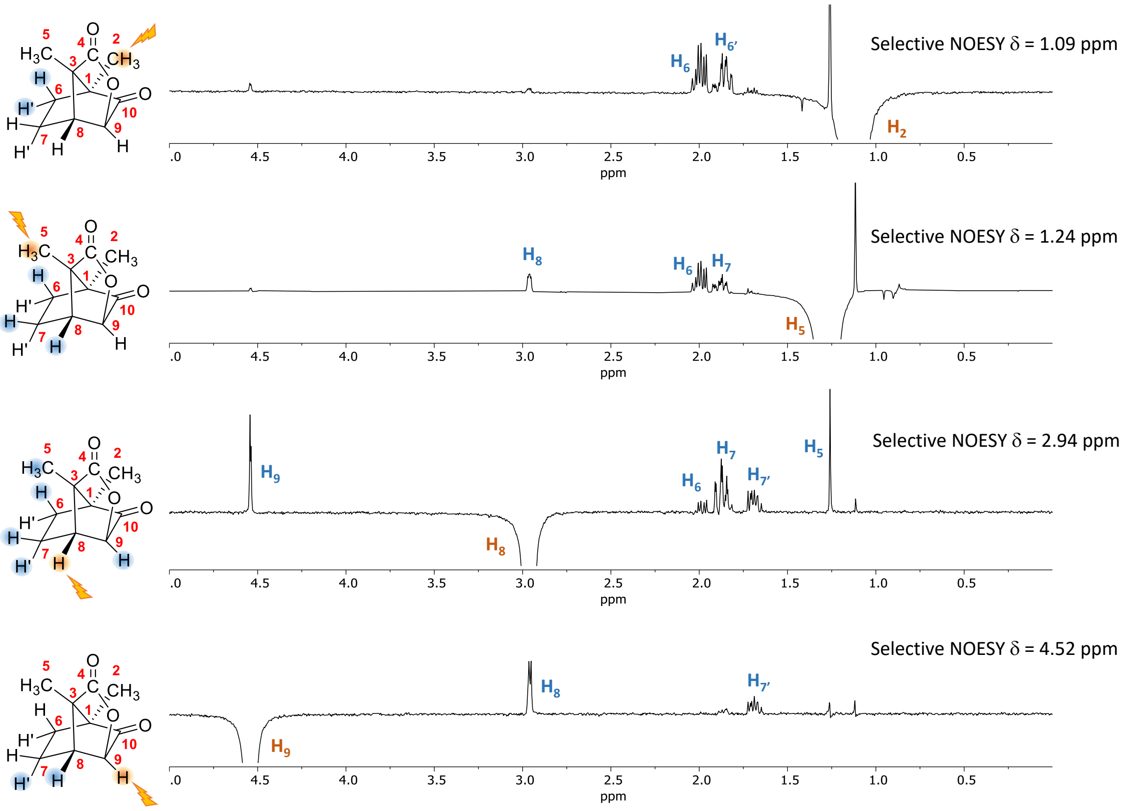

^{1}H-NMR of **18a** in $CDCl_3$

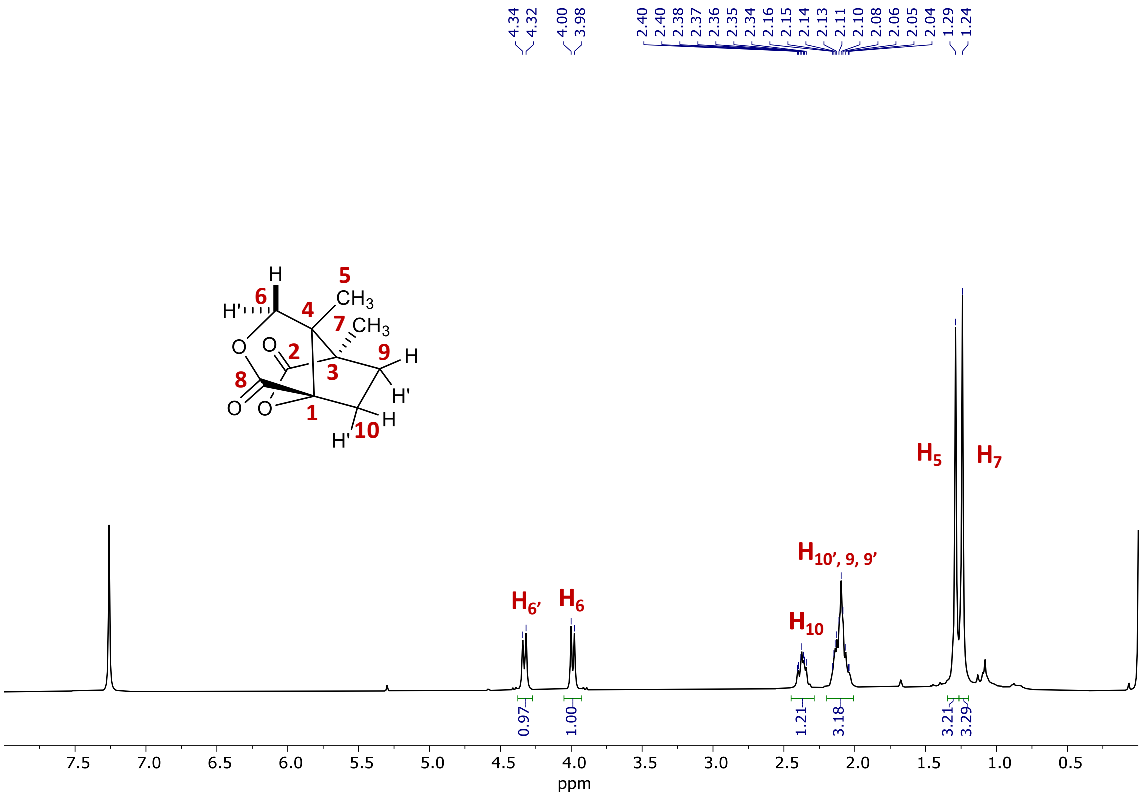

^{13}C-NMR of **18a** in $CDCl_3$

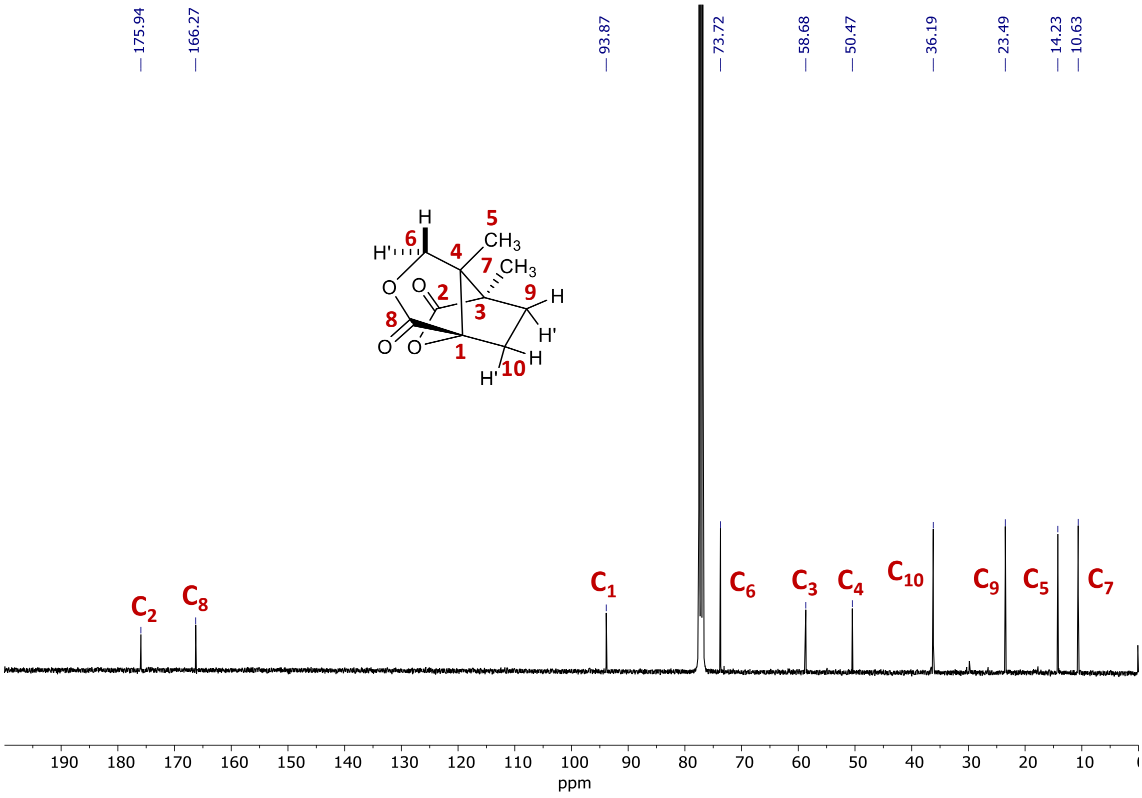

^{1}H-^{1}H COSY of **18a** in $CDCl_3$

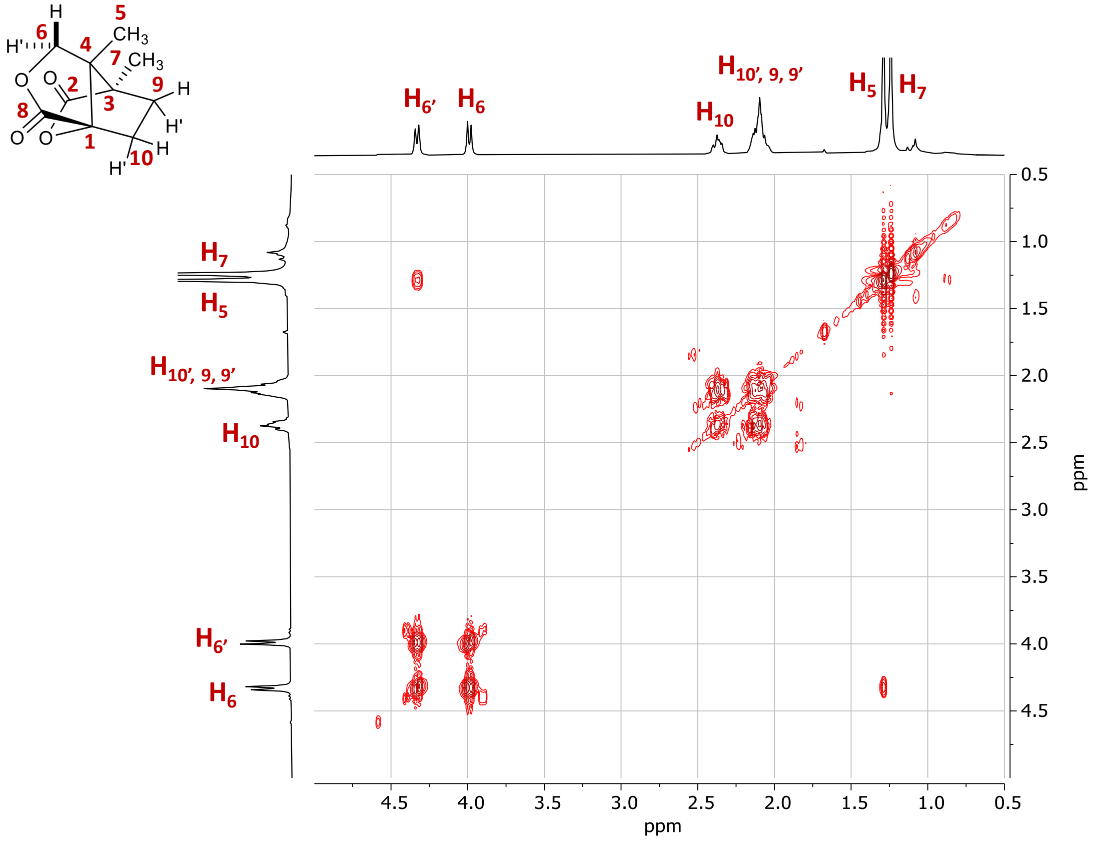

^{1}H-^{13}C HSQCed of **18a** in $CDCl_3$

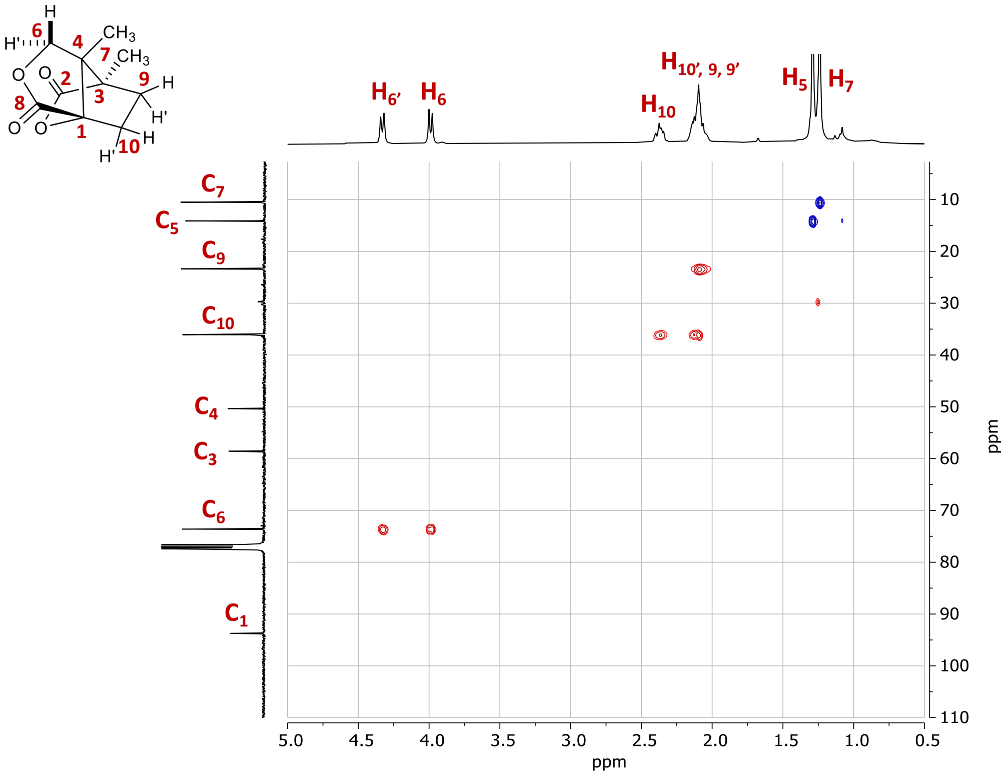

DEPTQ of **18a** in $CDCl_3$

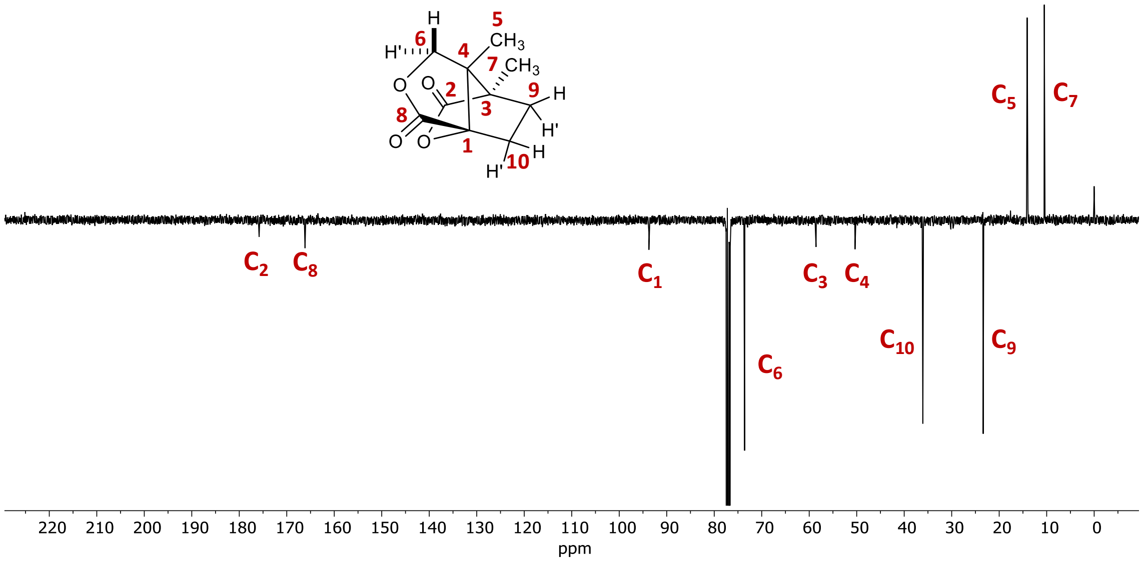

^{1}H-^{13}C HMBC of **18a** in $CDCl_3$

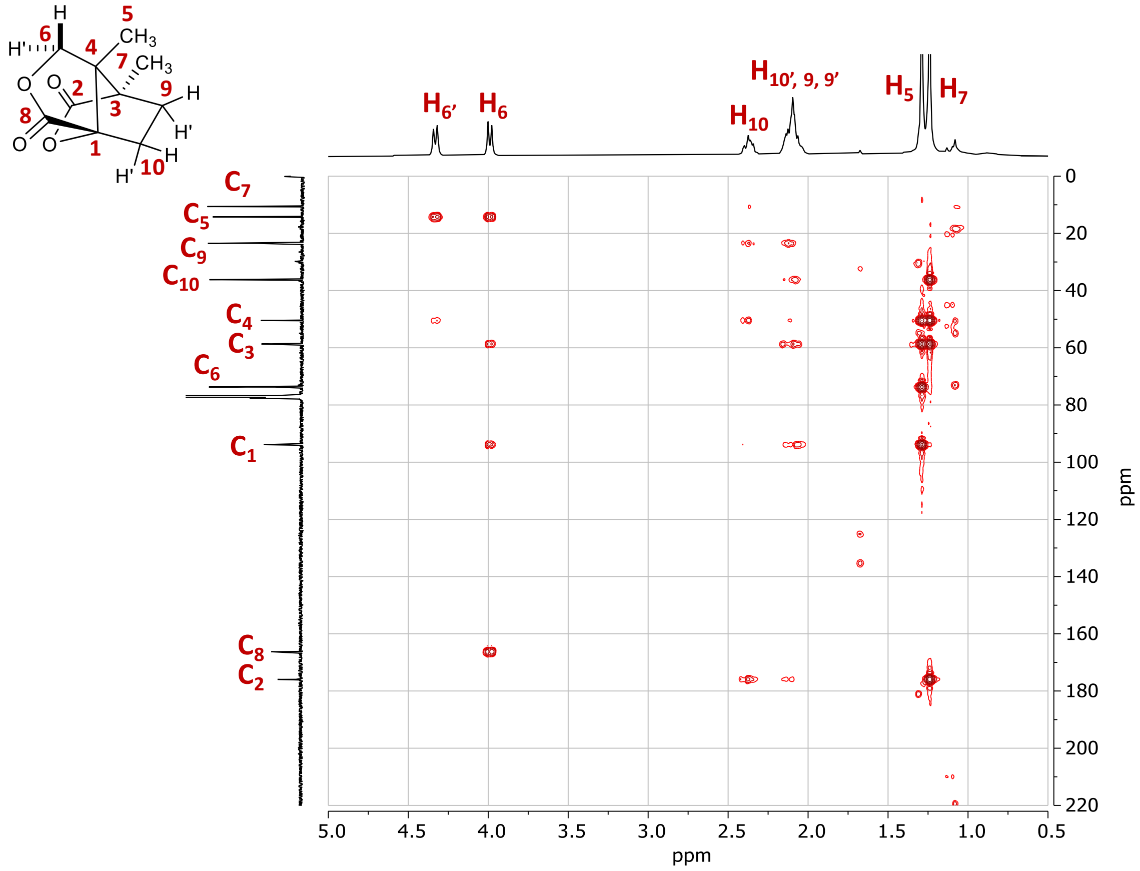

^{1}H-^{1}H TOCSY of **18a** in $CDCl_3$

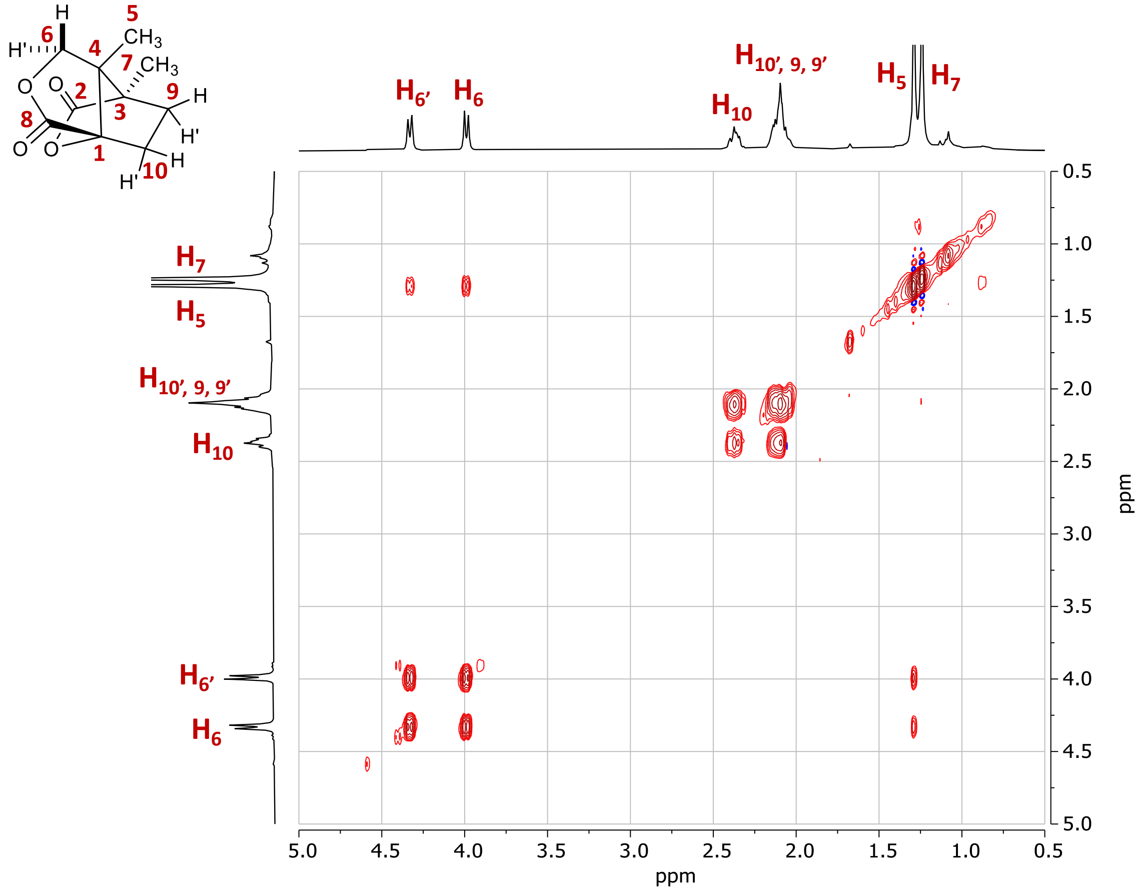

^{1}H-^{1}H NOESY of **18a** in $CDCl_3$

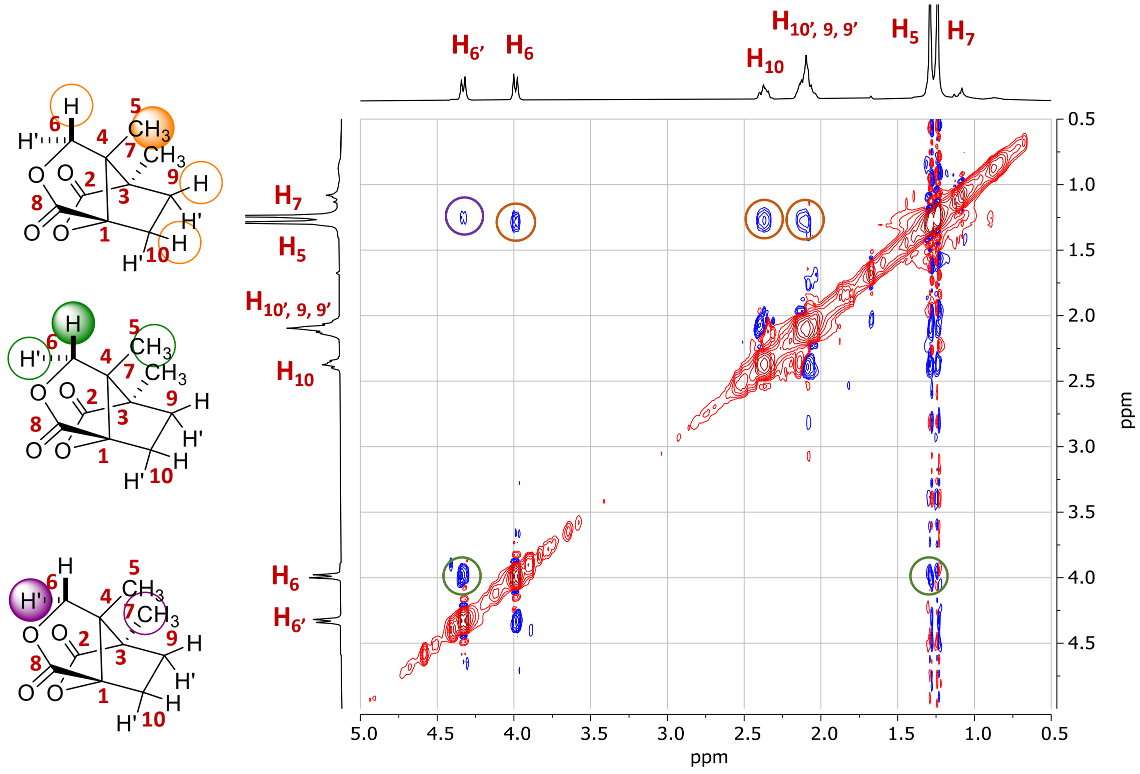

^{1}H-NMR of **18b** in $CDCl_3$

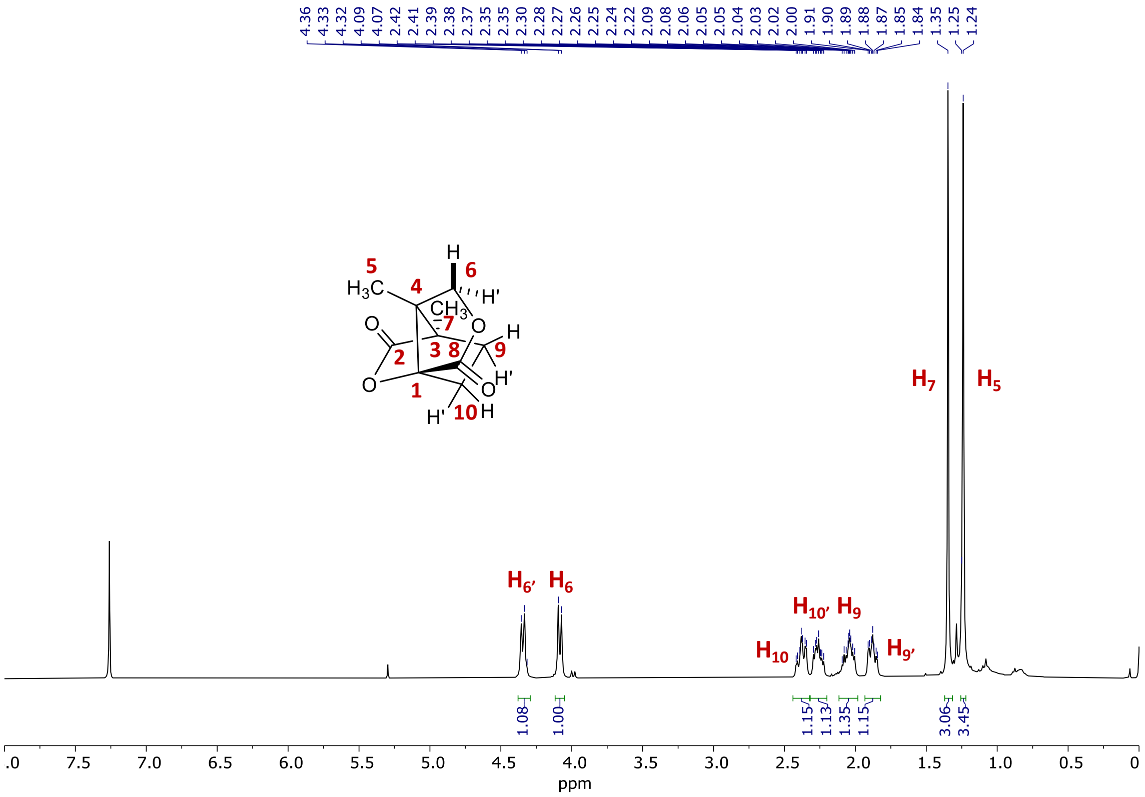

^{13}C-NMR of **18b** in $CDCl_3$

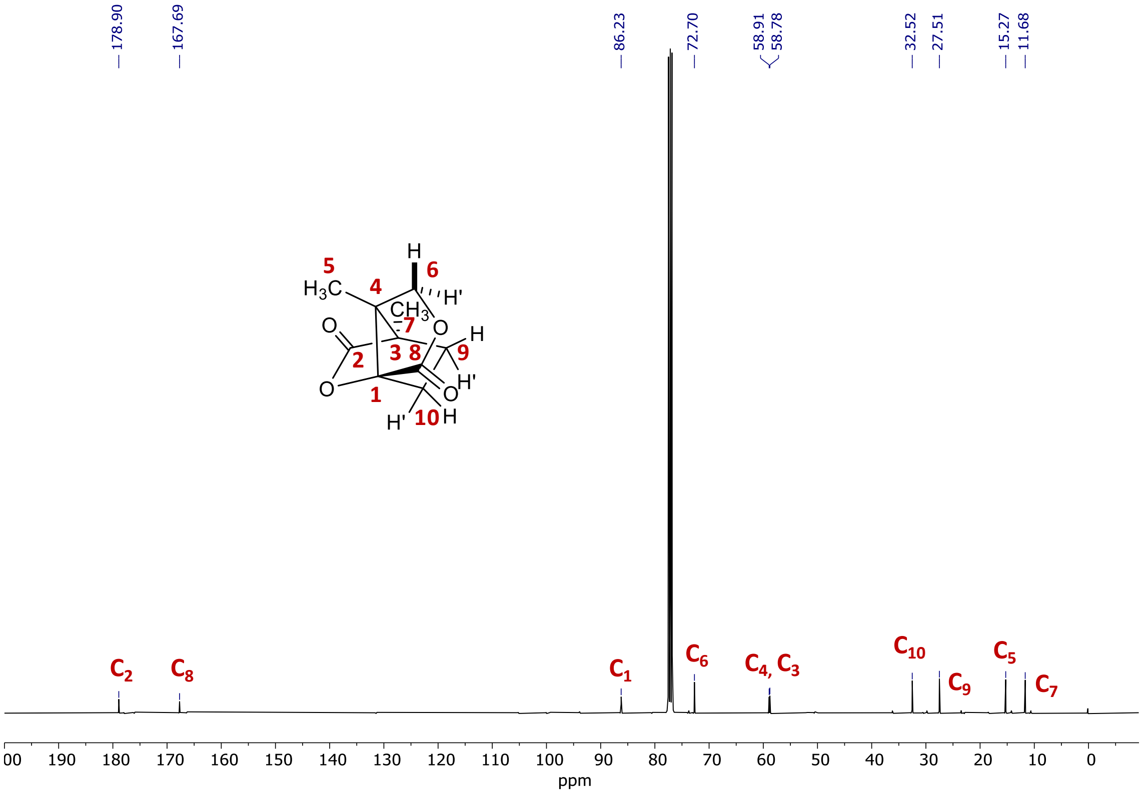

^{1}H-^{1}H COSY of **18b** in $CDCl_3$

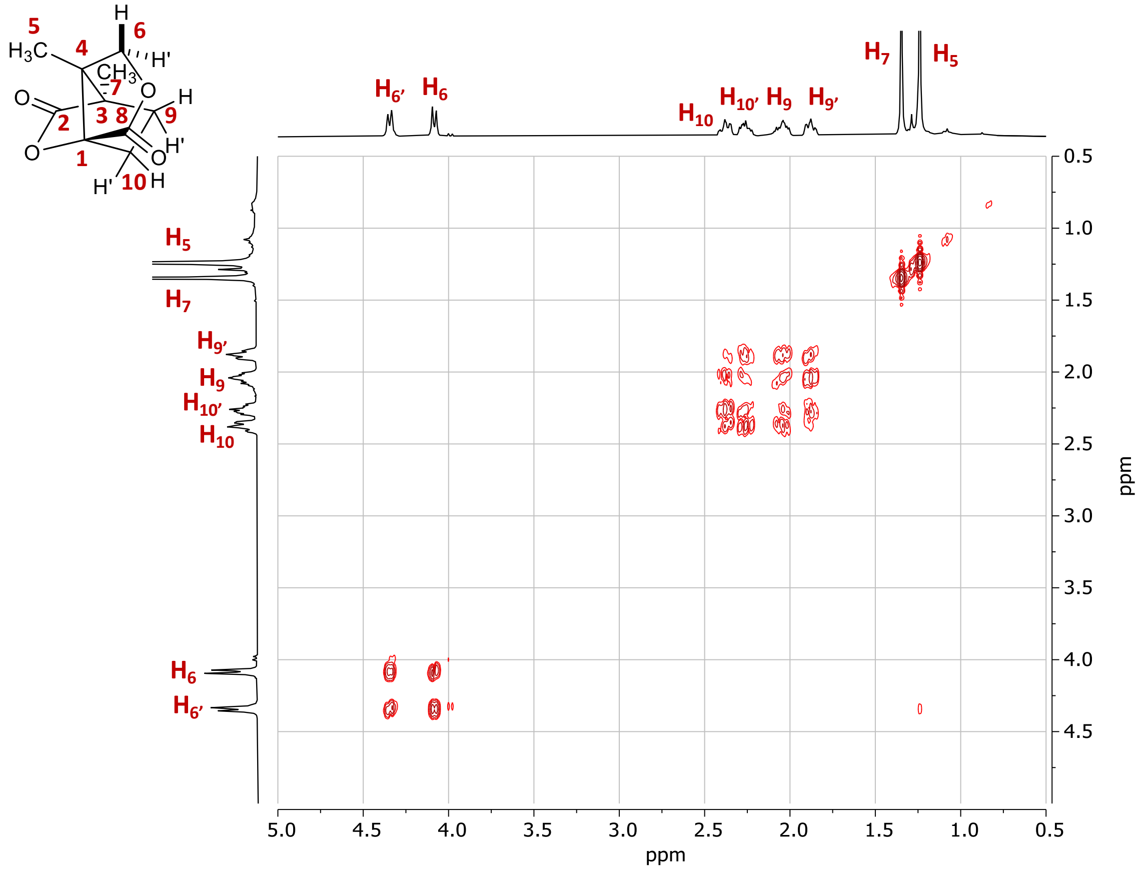

^{1}H-^{13}C HSQCed of **18b** in $CDCl_3$

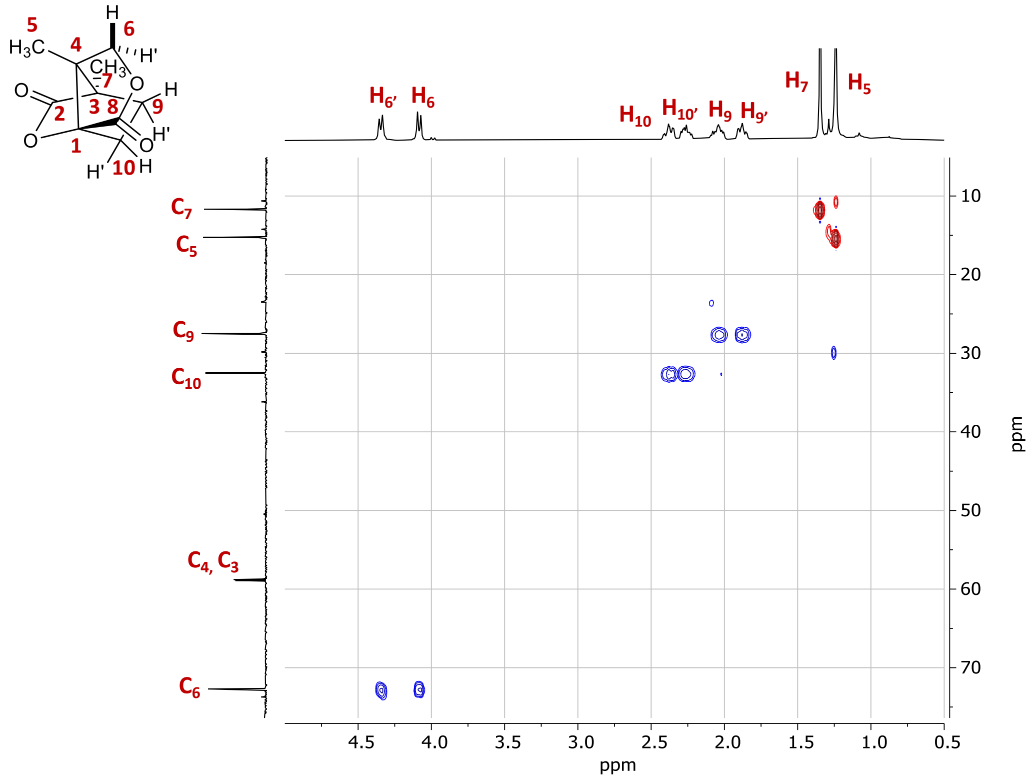

DEPTQ of **18b** in $CDCl_3$

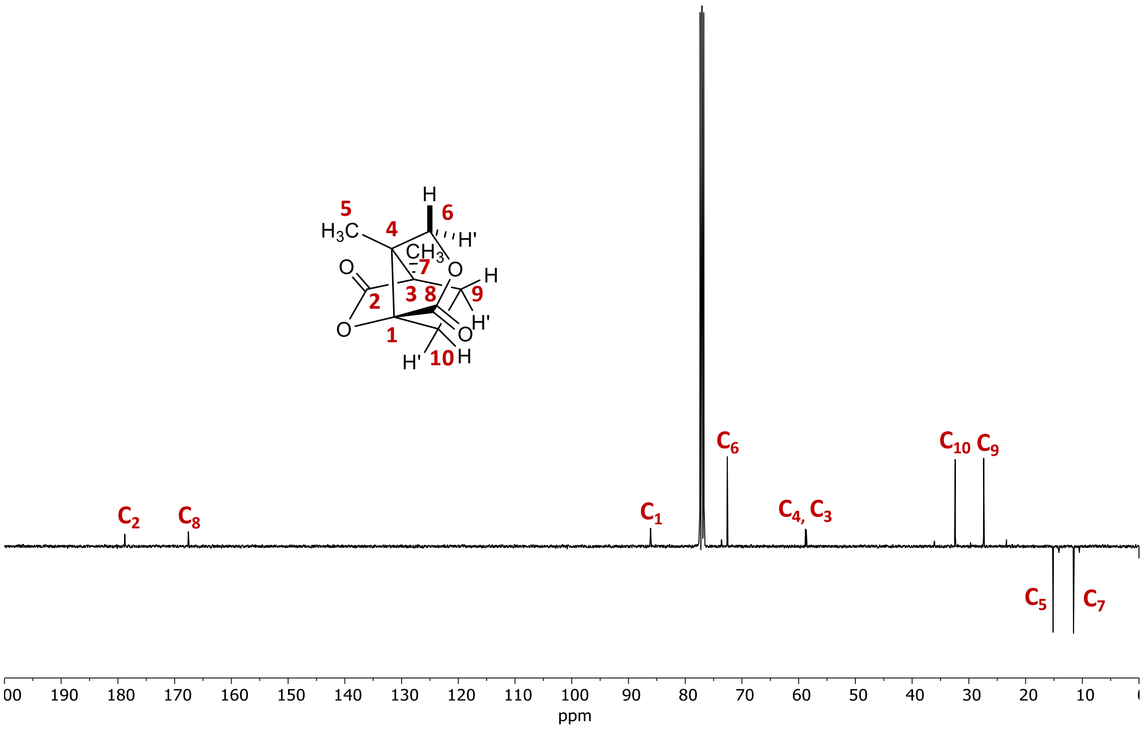

^1H-^{13}C HMBC of **18b** in $CDCl_3$

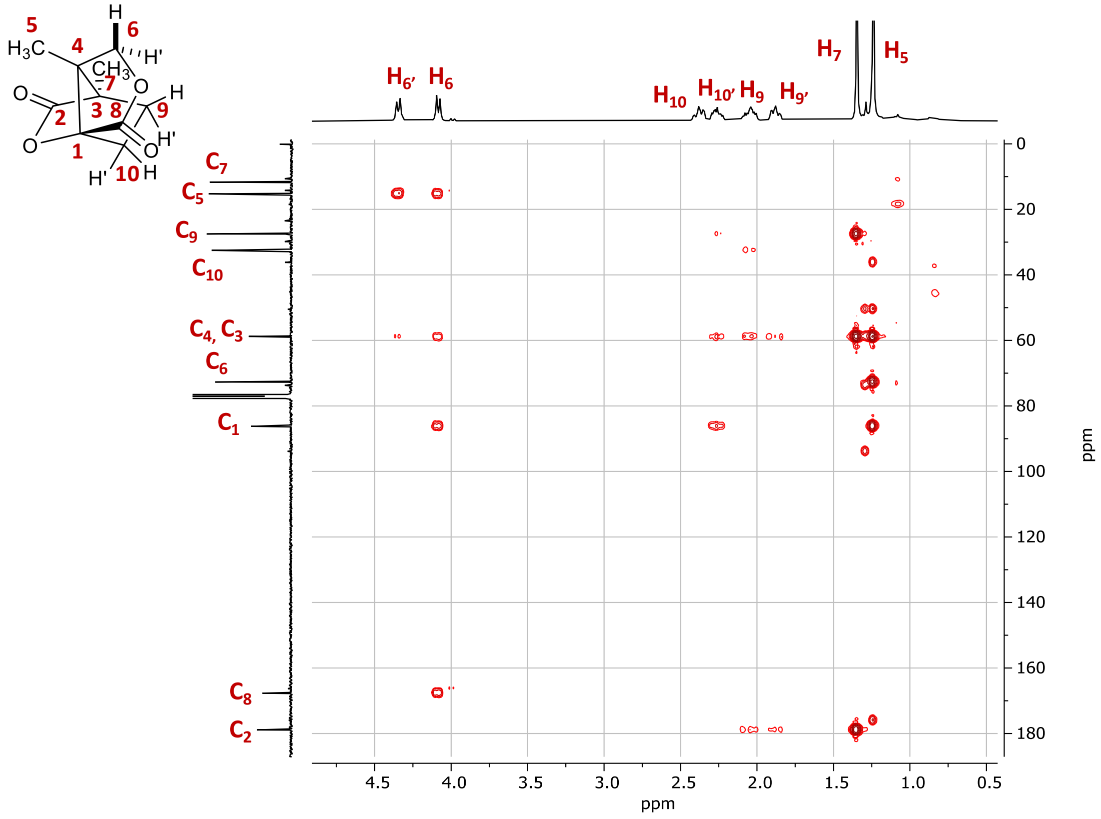

^{1}H-^{1}H TOCSY of **18b** in $CDCl_3$

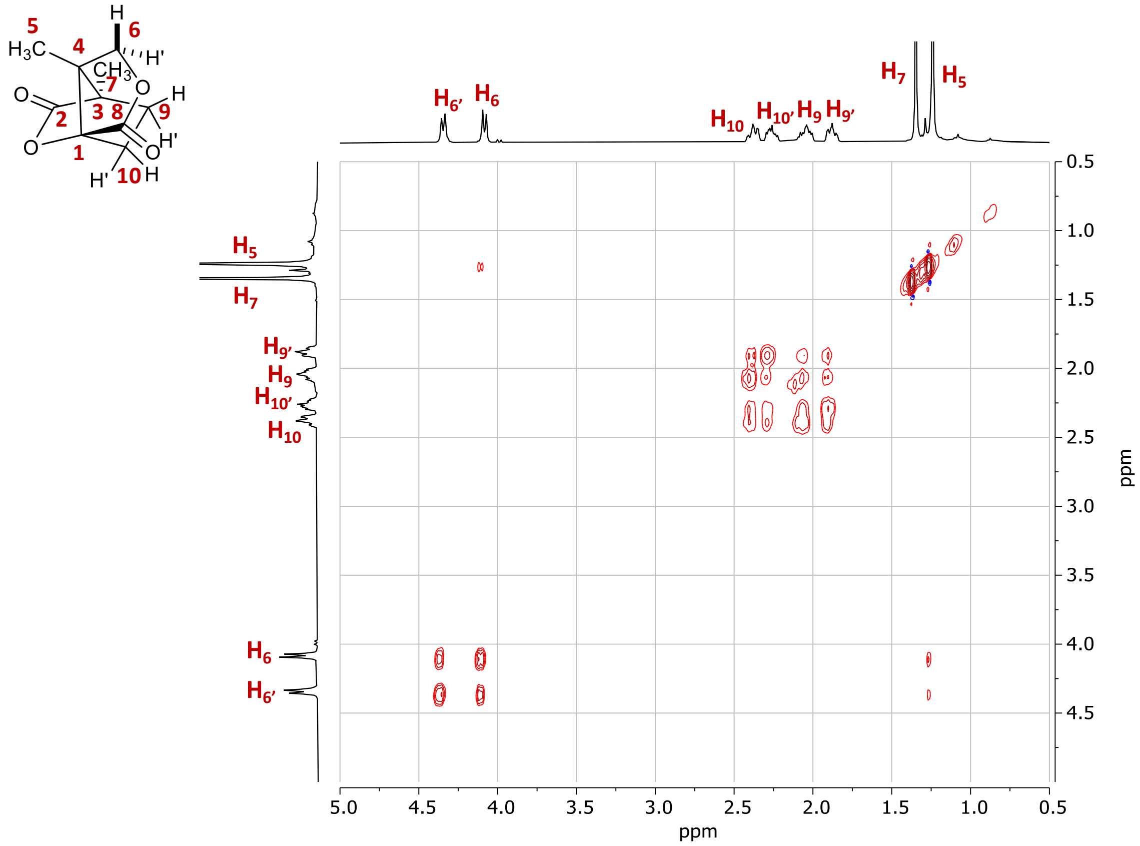

^{1}H-^{1}H NOESY of **18b** in $CDCl_3$

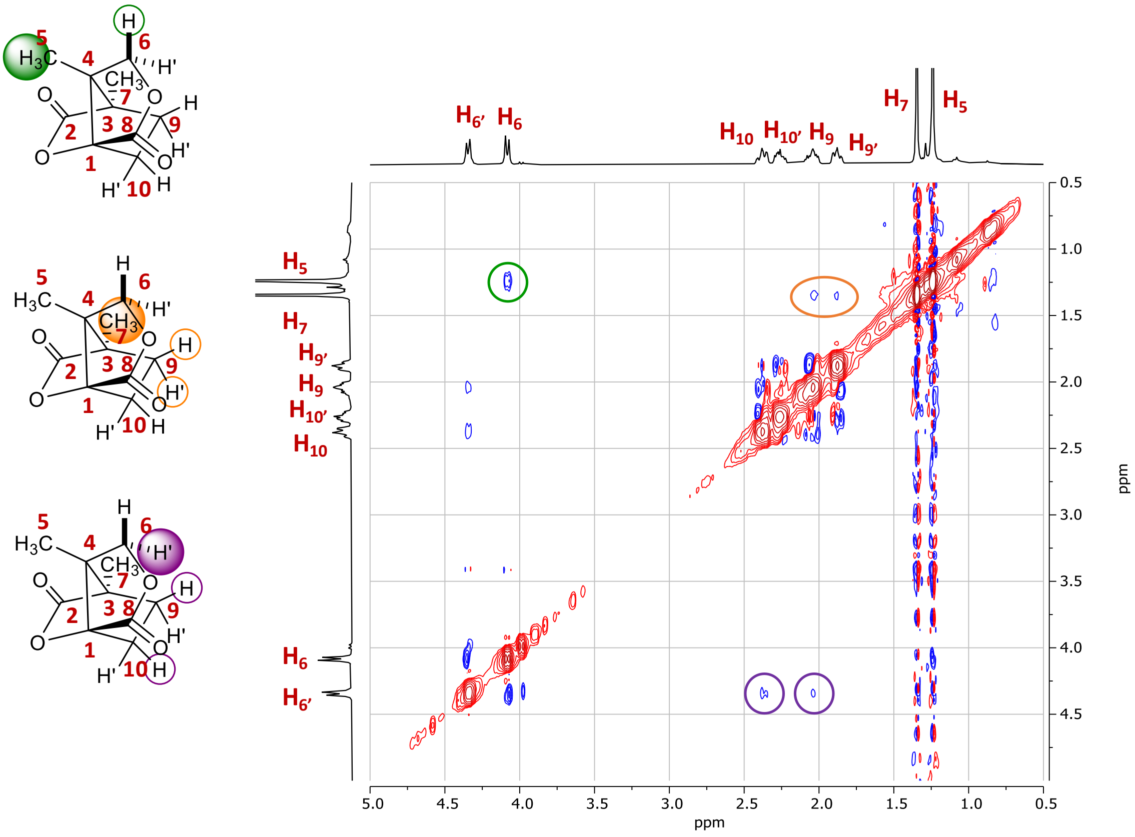

^{1}H-NMR of **19a** in $CDCl_3$

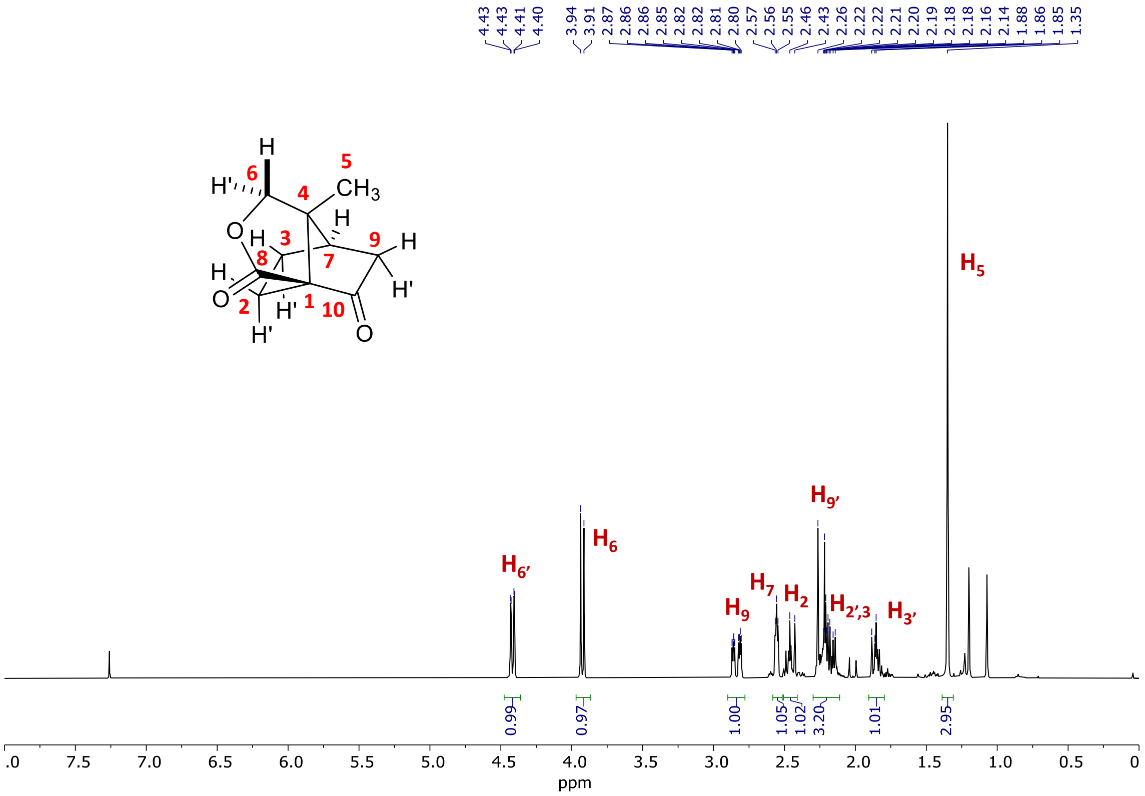

^{13}C-NMR of **19a** in $CDCl_3$

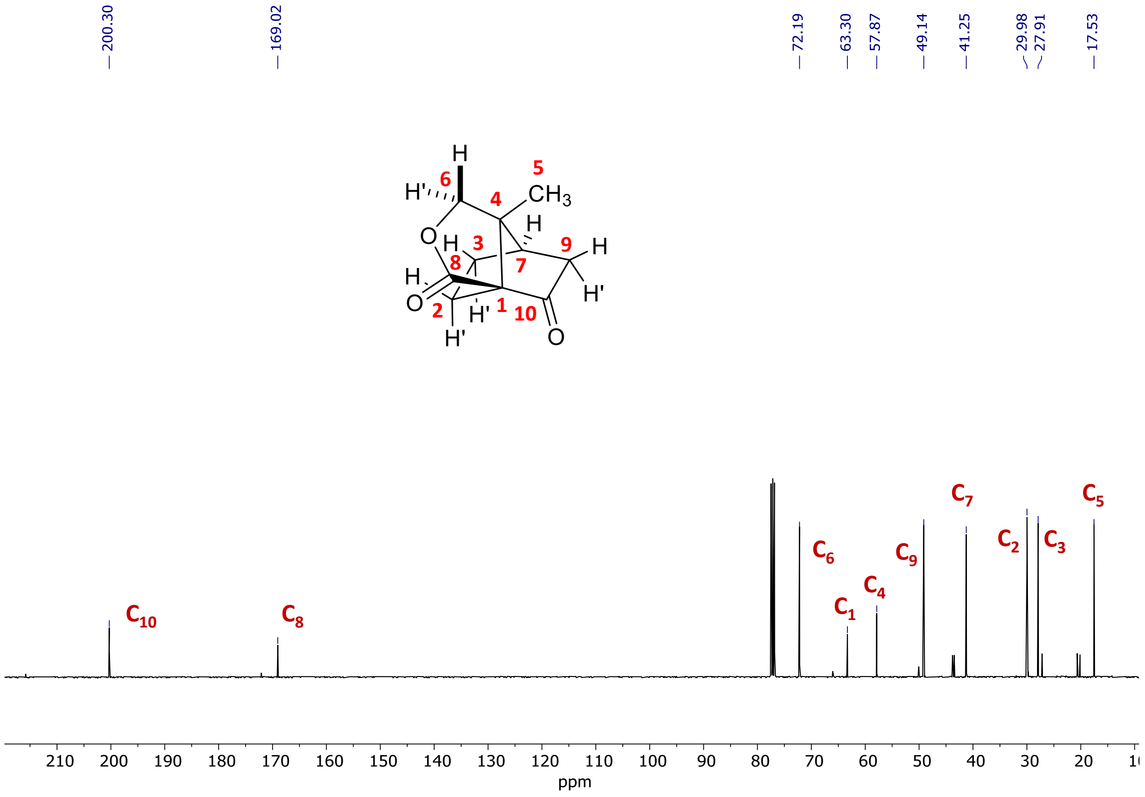

^{1}H-^{1}H COSY of **19a** in $CDCl_3$

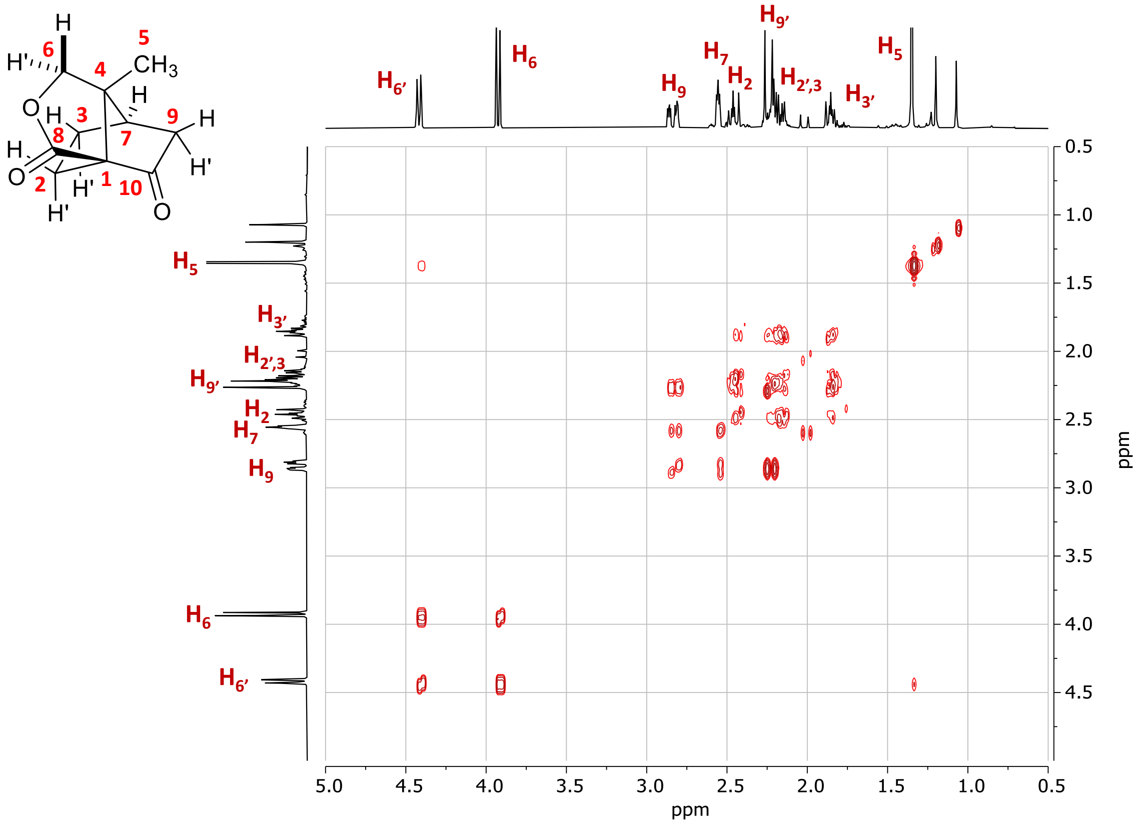

^{1}H-^{13}C HSQCed of **19a** in $CDCl_3$

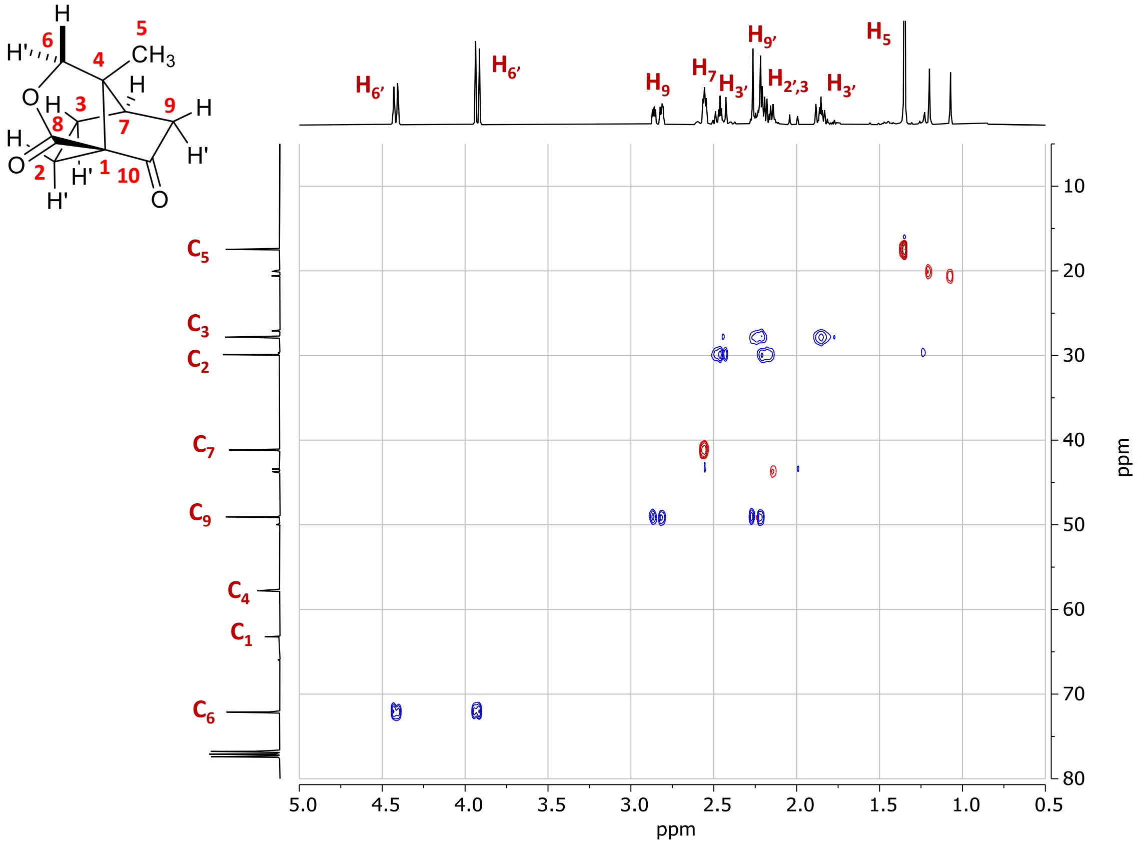

DEPTQ of **19a** in $CDCl_3$

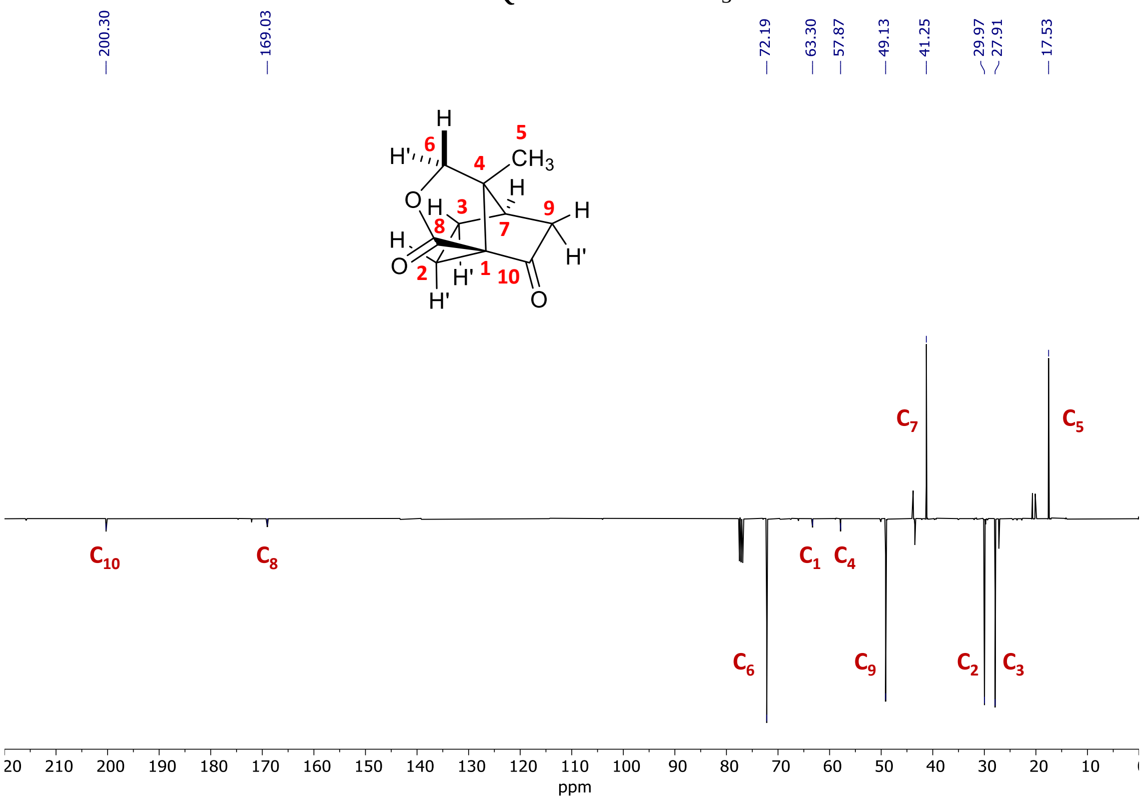

DEPT135 of **19a** in $CDCl_3$

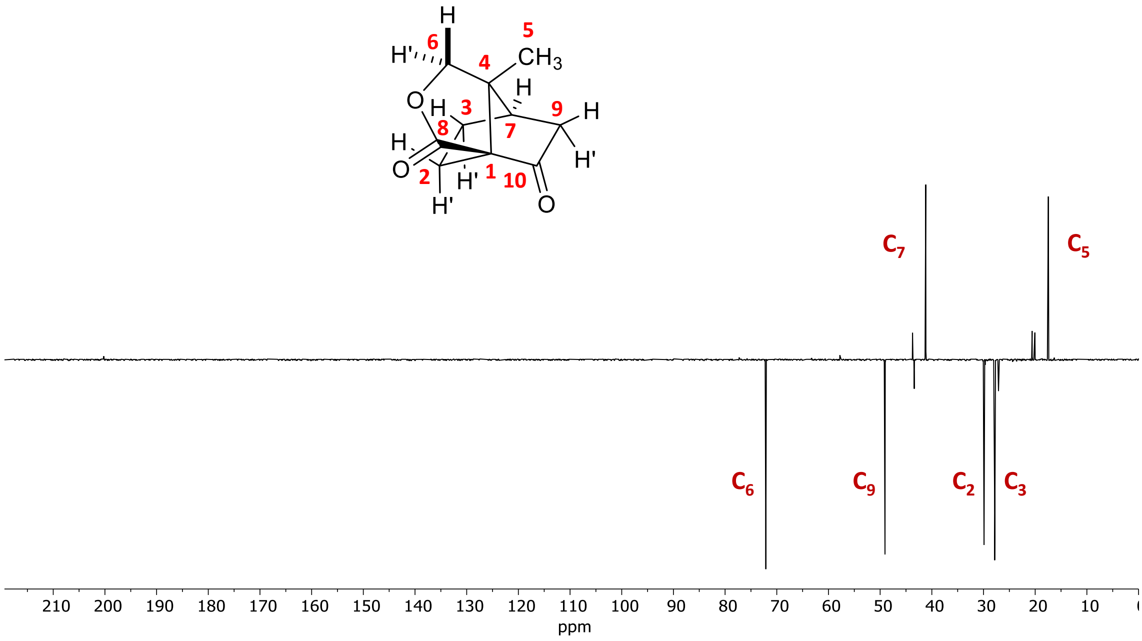

^{1}H-^{13}C HMBC of **19a** in $CDCl_3$

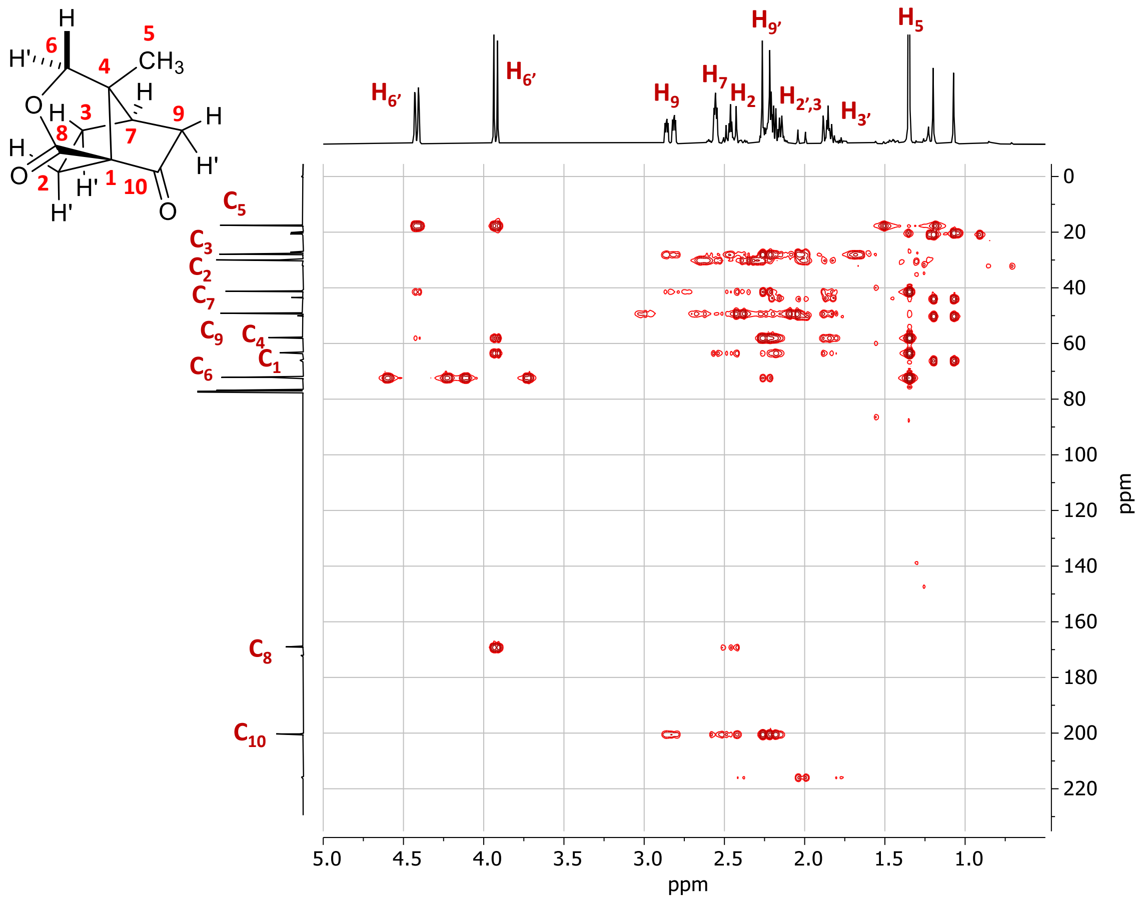

^{1}H-^{1}H TOCSY of **19a** in $CDCl_3$

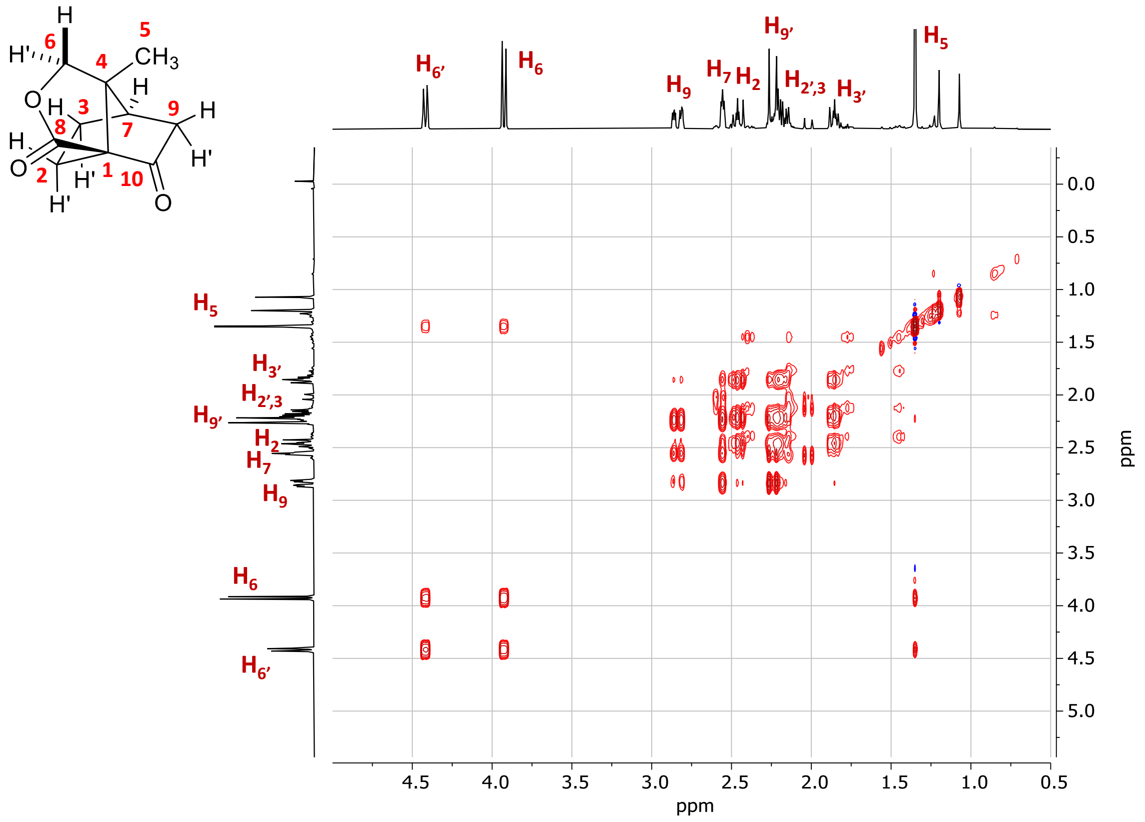

^{1}H-^{1}H NOESY of **19a** in $CDCl_3$

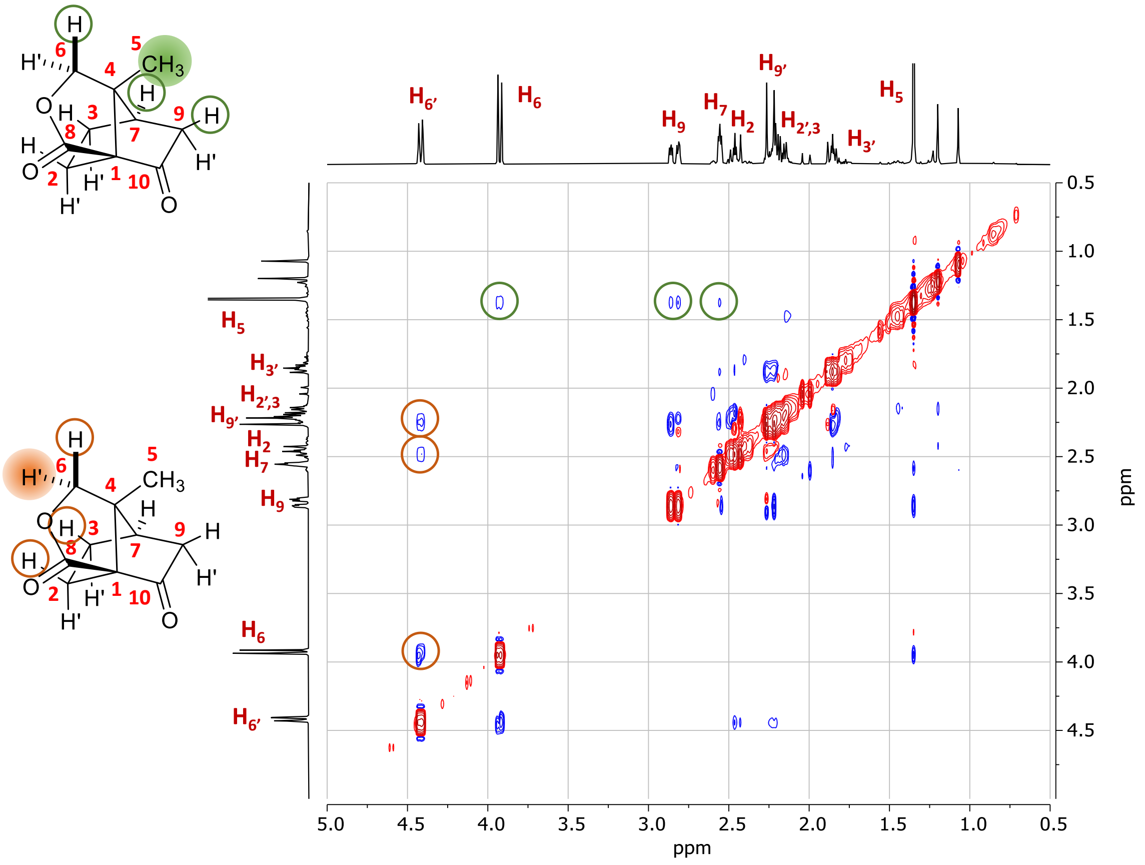

Selective NOESY experiments of **19a** in $CDCl_3$

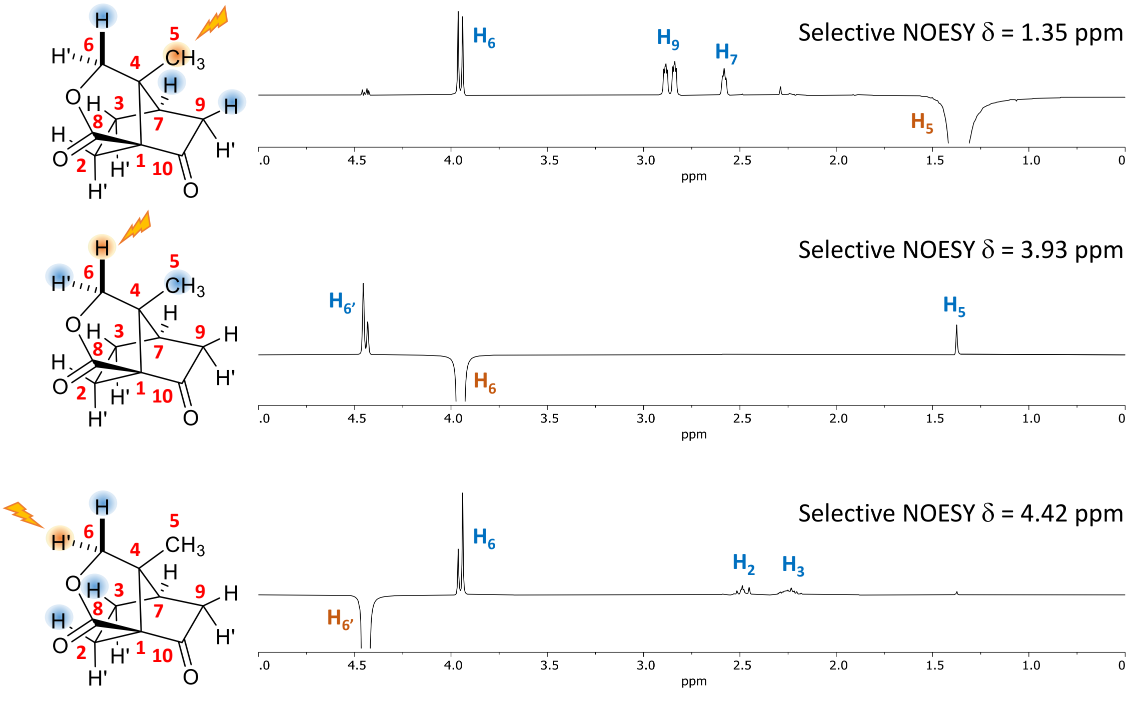

^{1}H-NMR of **19b** in $CDCl_3$

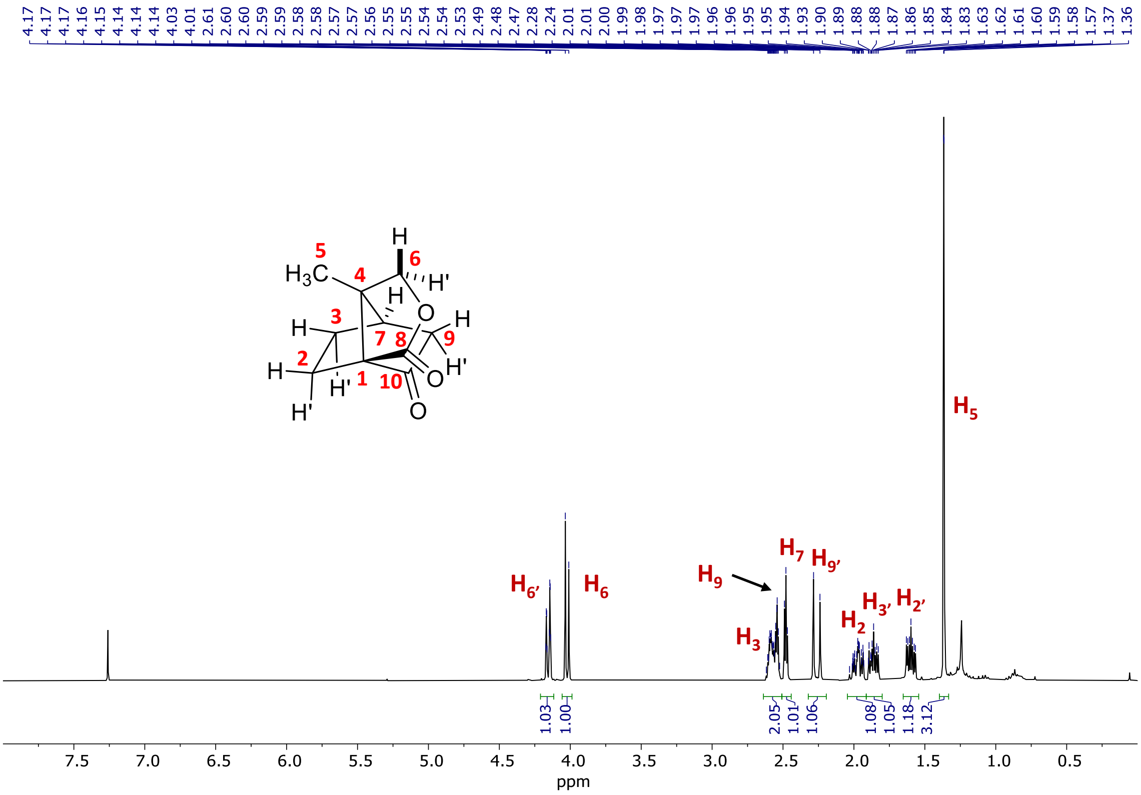

^{13}C-NMR of **19b** in $CDCl_3$

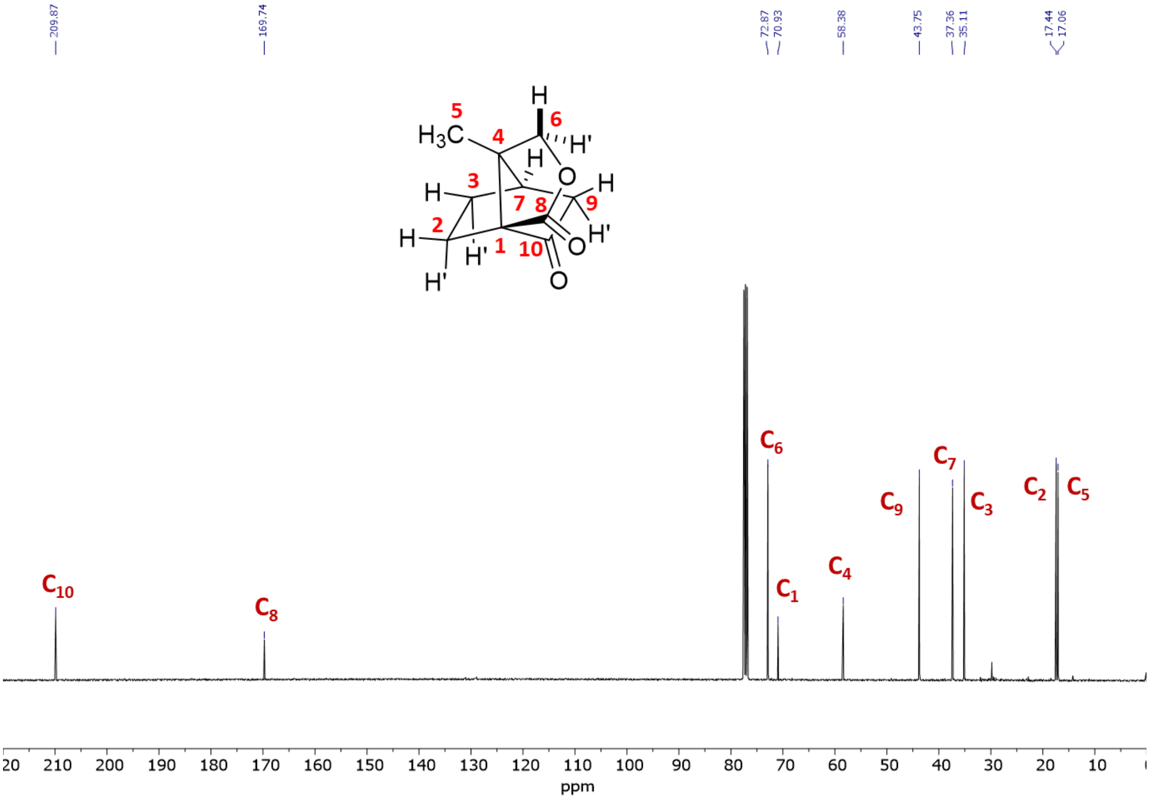

^{1}H-^{1}H COSY of **19b** in $CDCl_3$

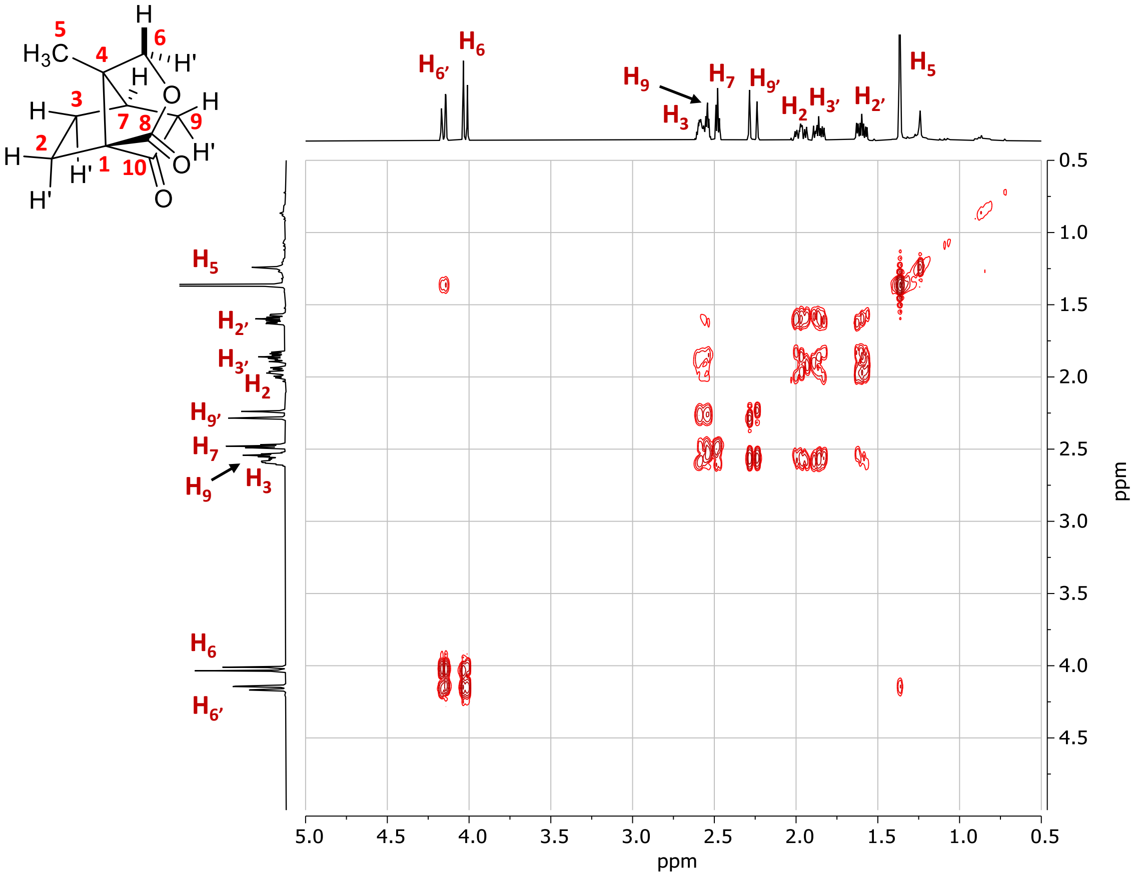

^{1}H-^{13}C HSQCed of **19b** in $CDCl_3$

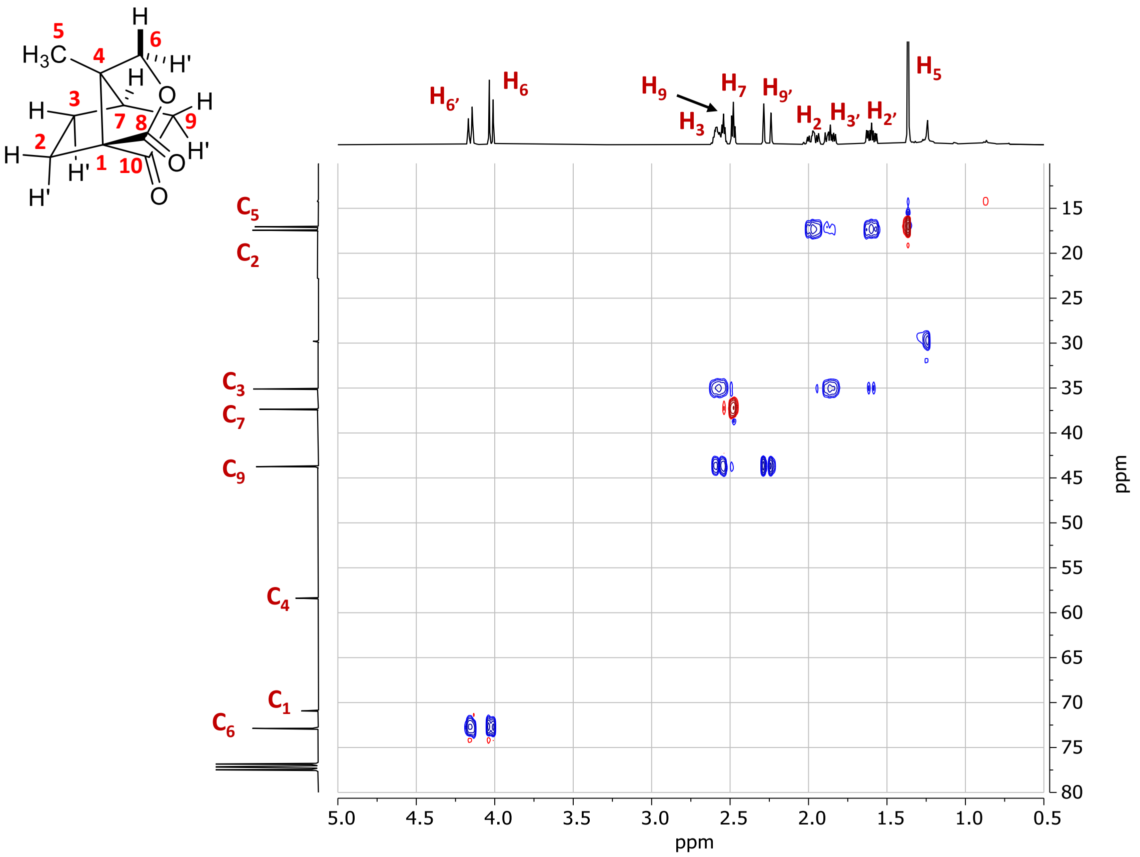

DEPTQ of **19b** in $CDCl_3$

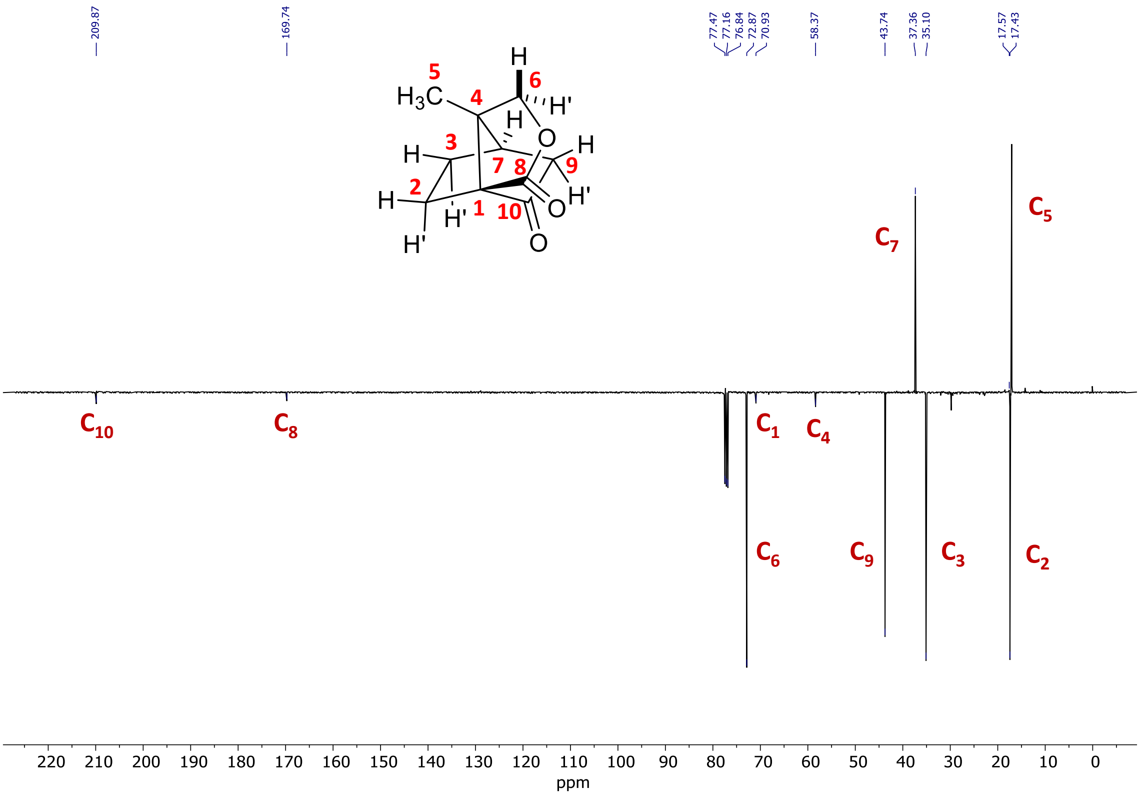

DEPT135 of **19b** in $CDCl_3$

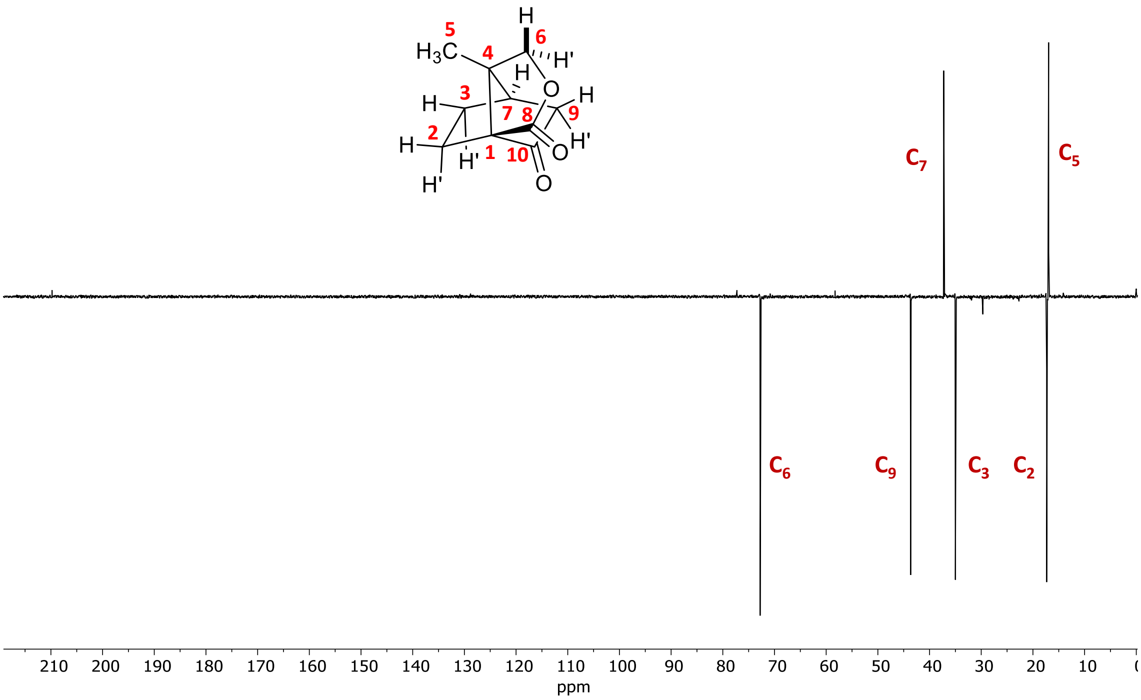

^{1}H-^{13}C HMBC of **19b** in $CDCl_3$

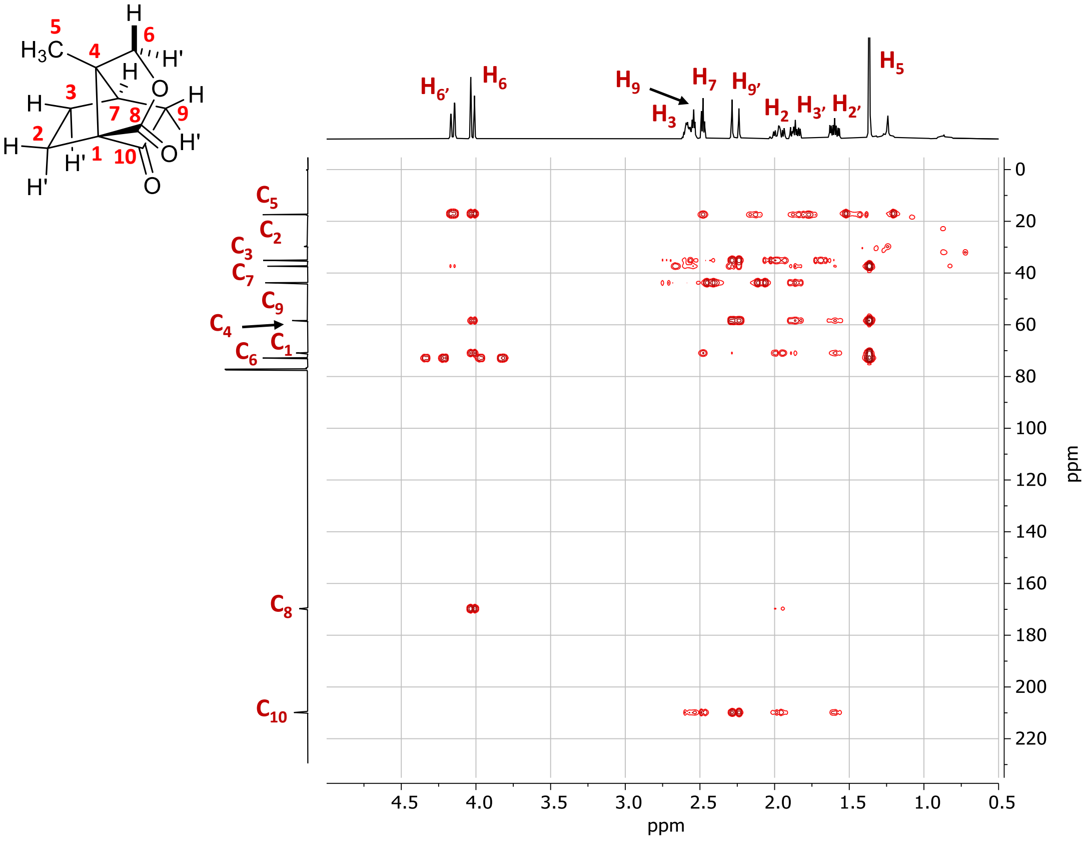

^{1}H-^{1}H TOCSY of **19b** in $CDCl_3$

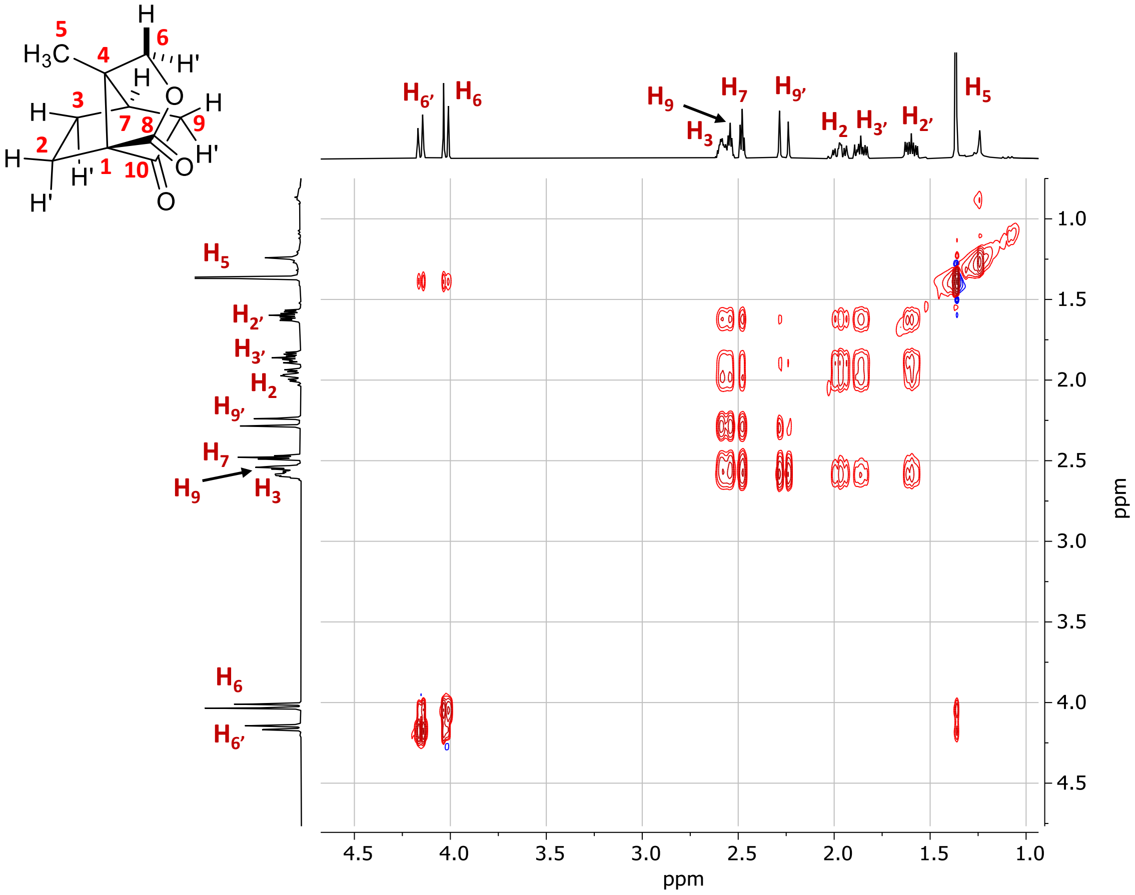

^{1}H-^{1}H NOESY of **19b** in $CDCl_3$

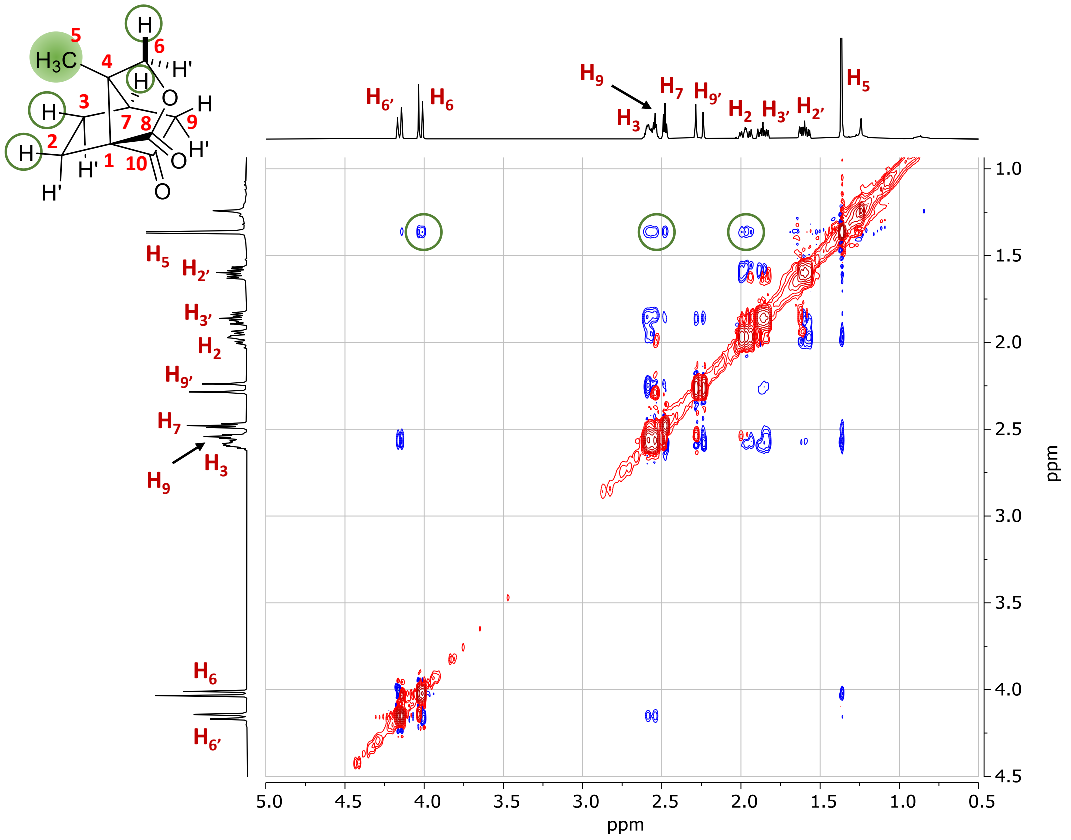

Selective NOESY experiments of **19b** in $CDCl_3$

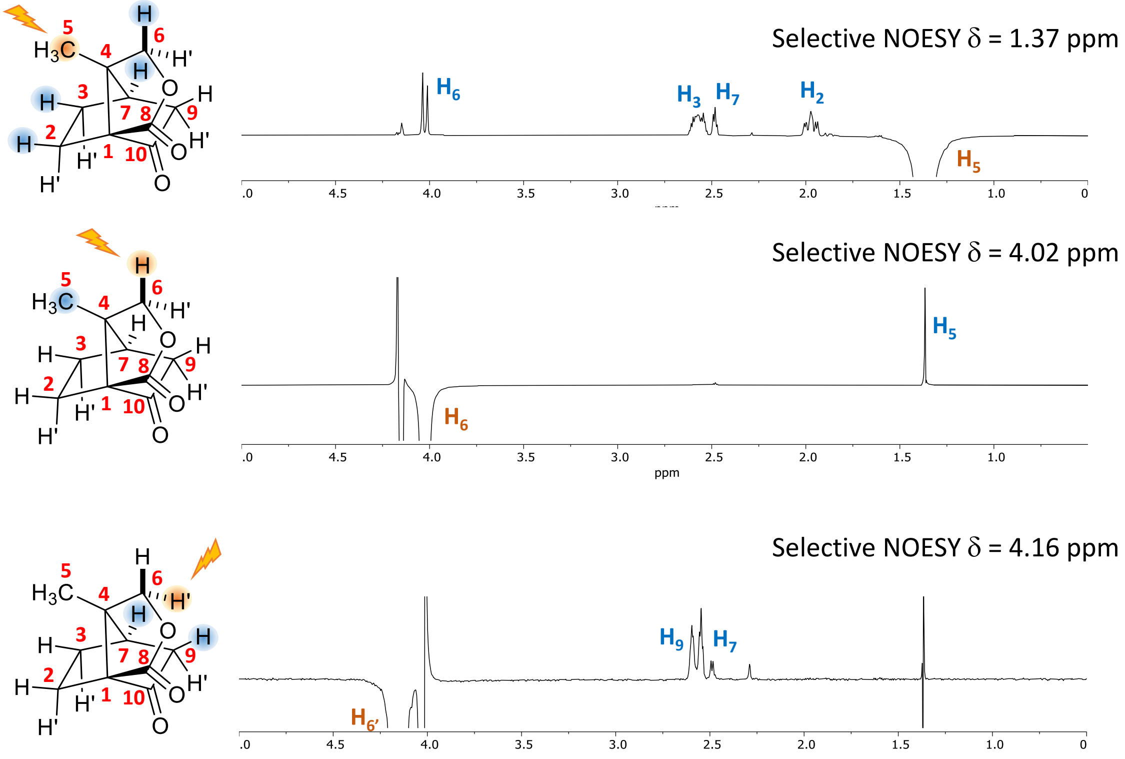

^{1}H-NMR of **19a-I** in $CDCl_3$

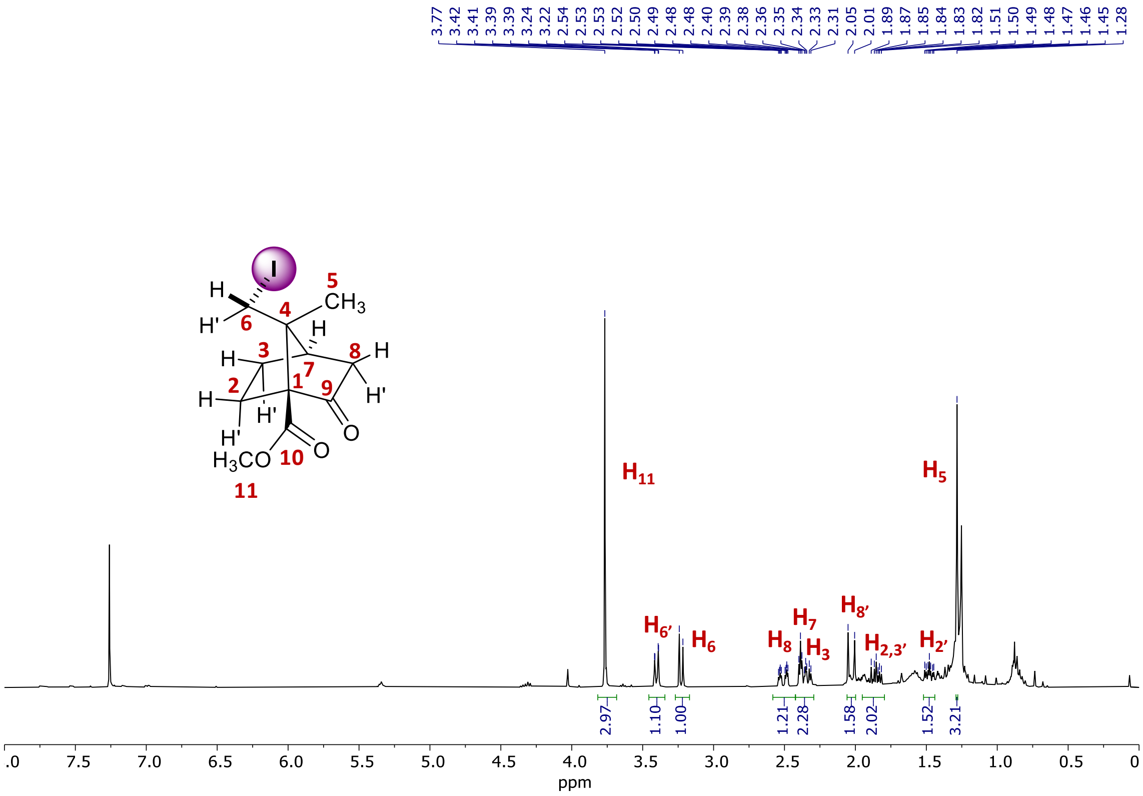

^{13}C-NMR of **19a-I** in $CDCl_3$

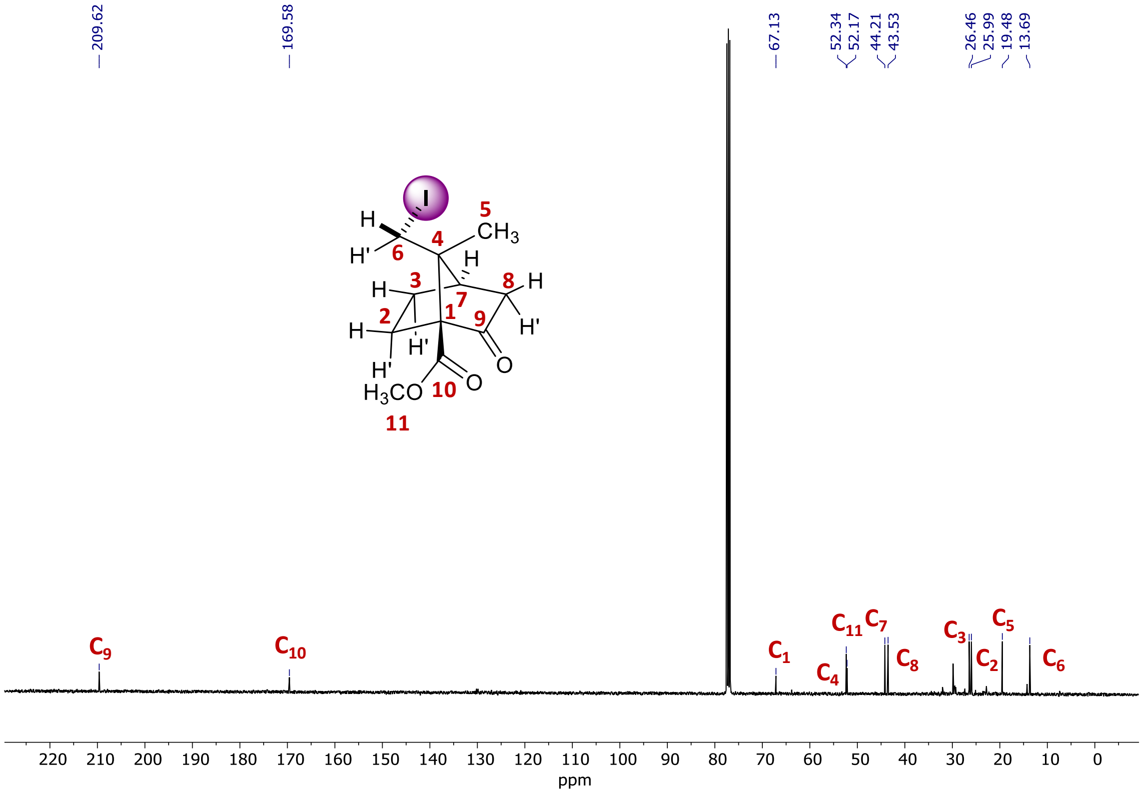

^1H-^1H COSY of **19a-I** in $CDCl_3$

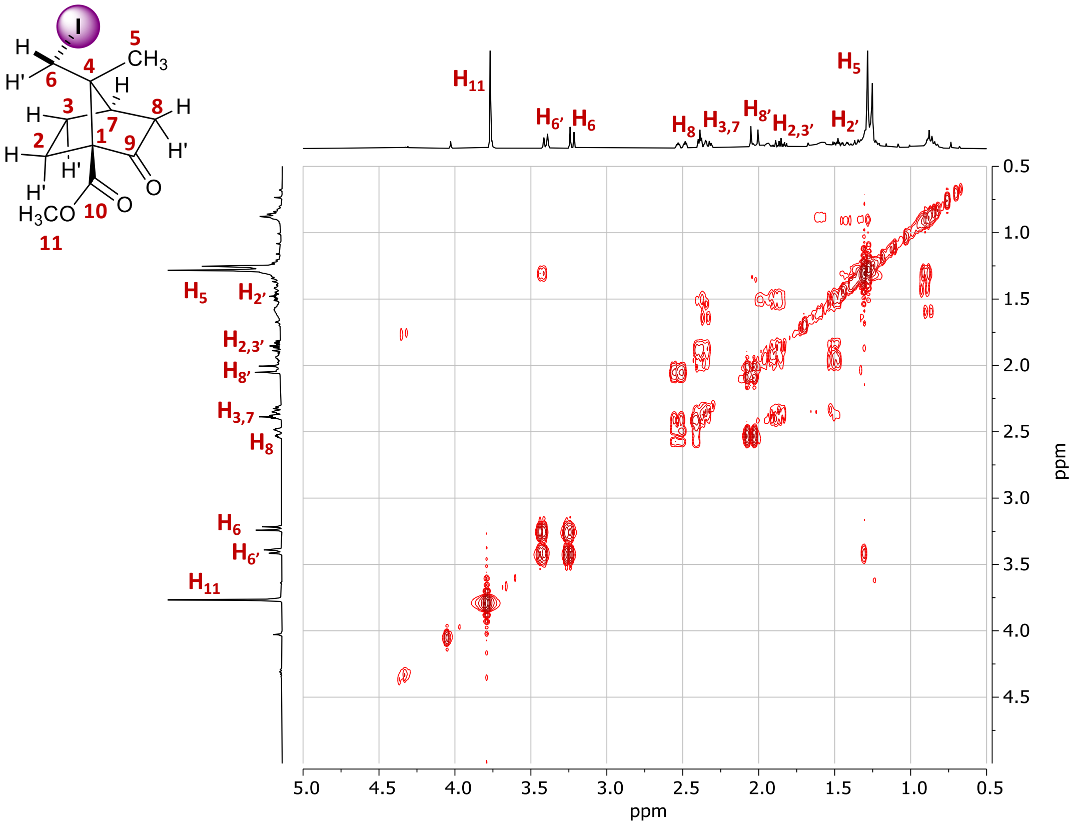

^1H-^{13}C HSQCed of **19a-I** in $CDCl_3$

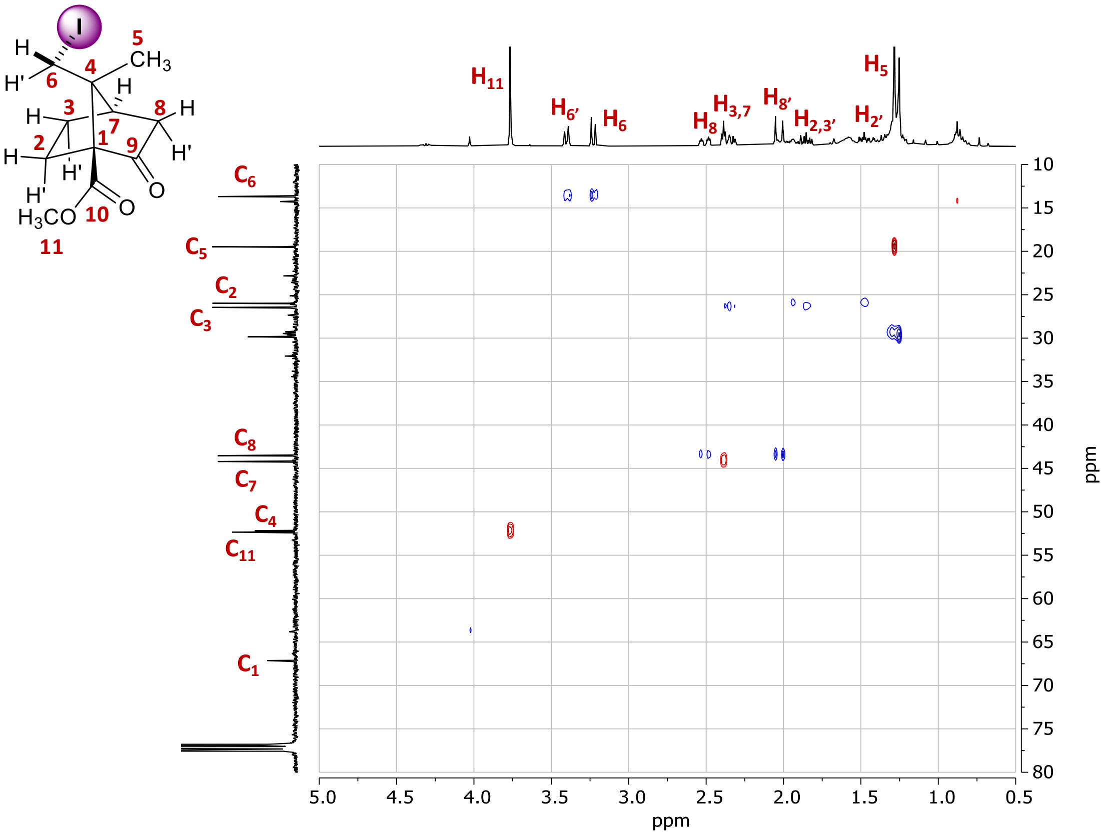

DEPTQ of **19a-I** in $CDCl_3$

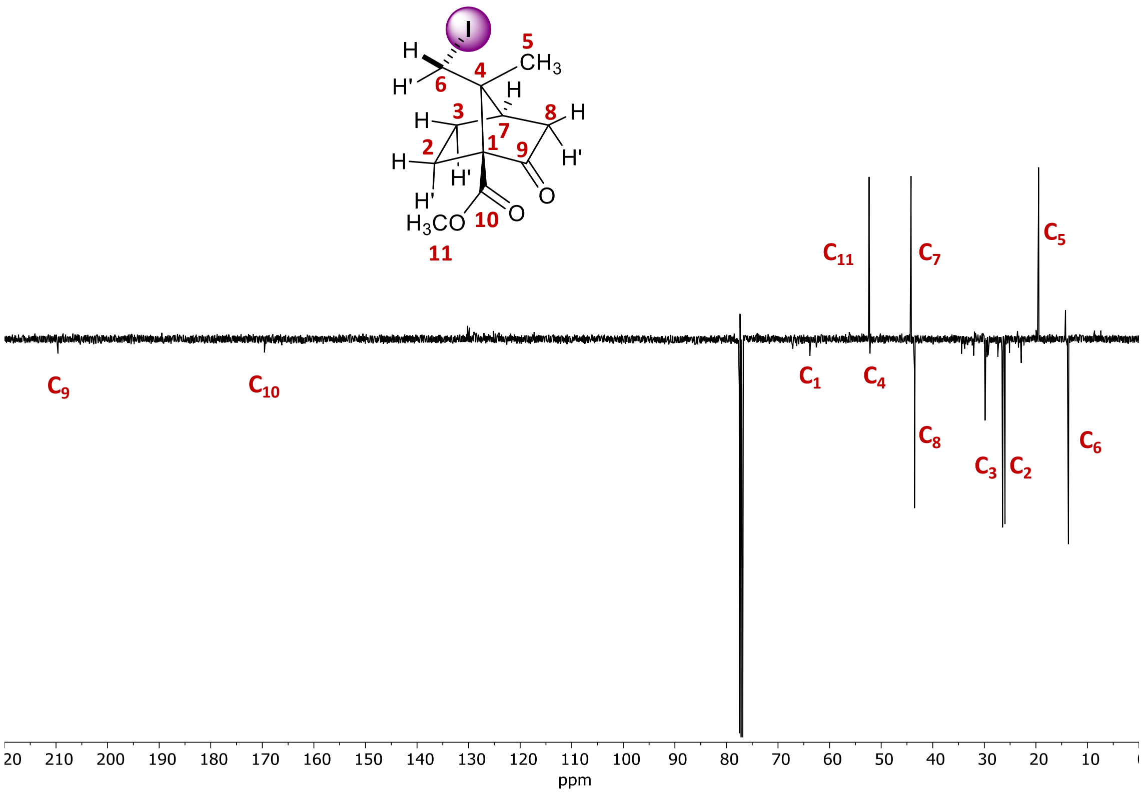

^{1}H-^{13}C HMBC of **19a-I** in $CDCl_3$

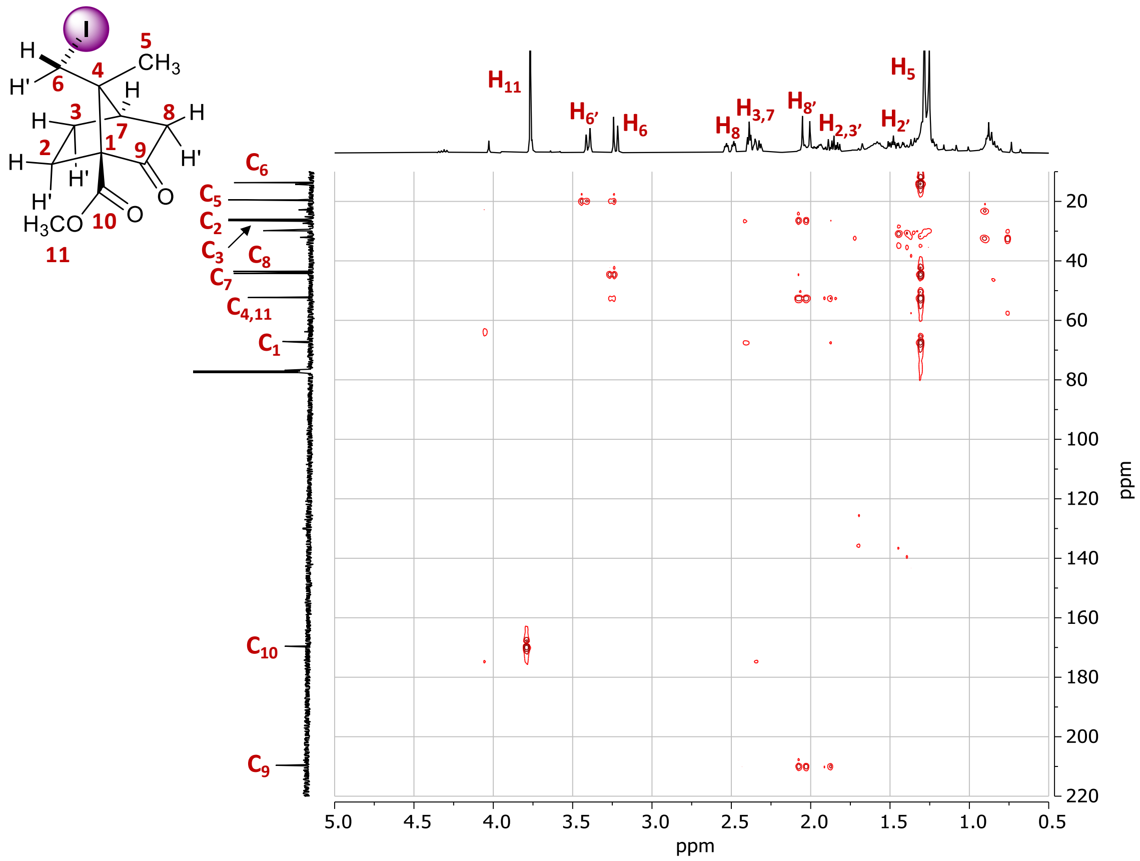

^{1}H-^{1}H TOCSY of **19a-I** in $CDCl_3$

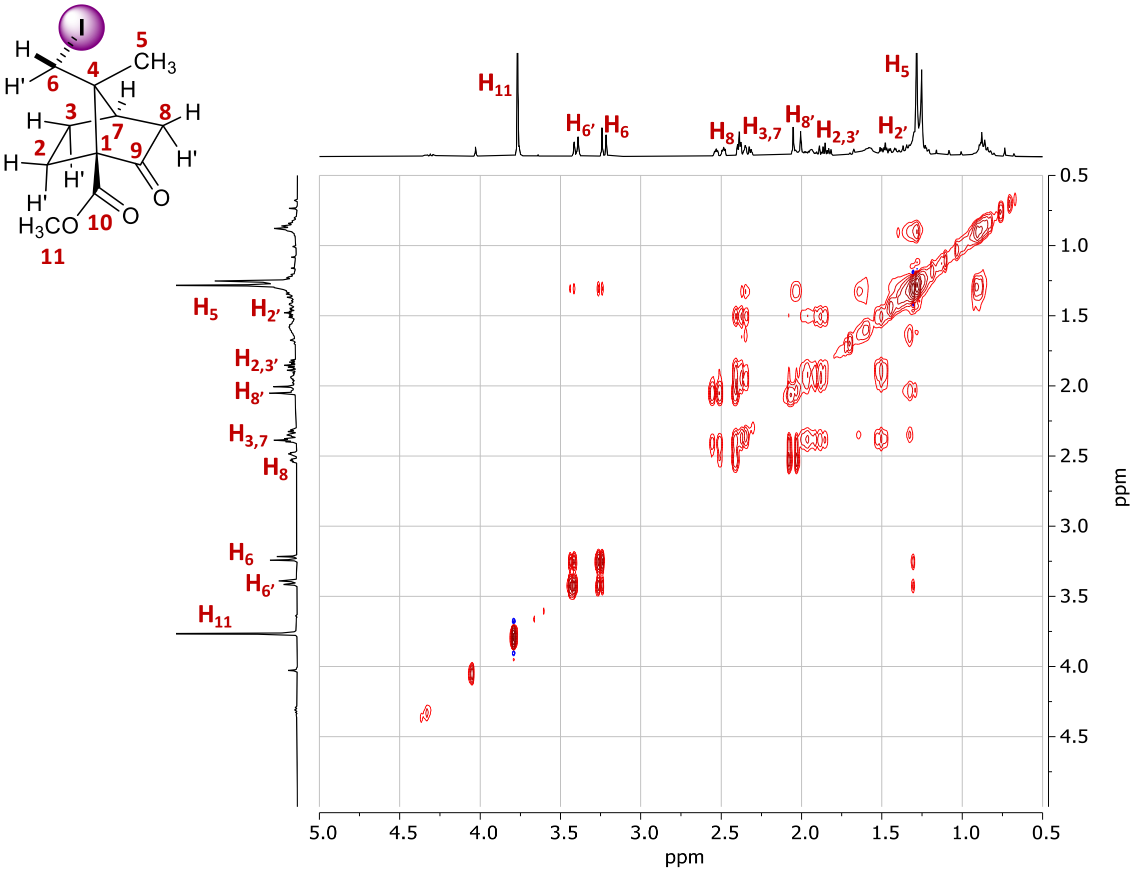

^{1}H-^{1}H NOESY of **19a-I** in $CDCl_3$

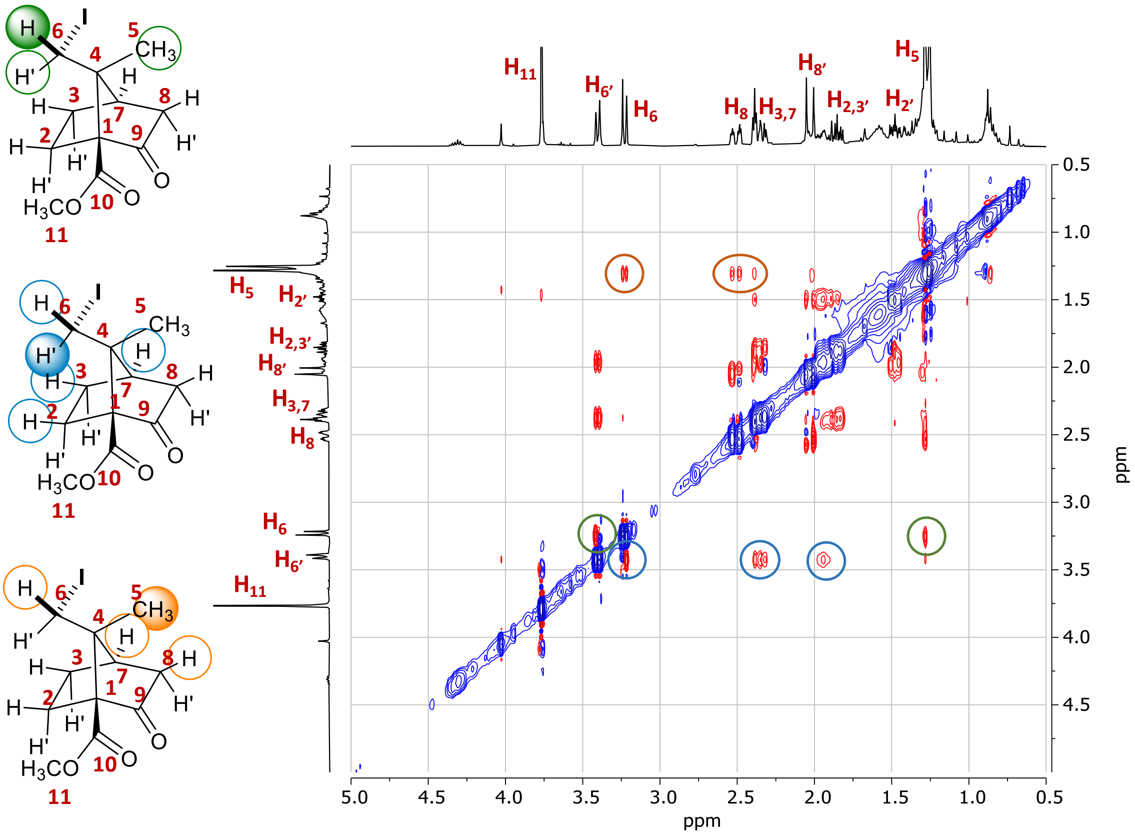

Selective NOESY experiments of **19a-I** in $CDCl_3$

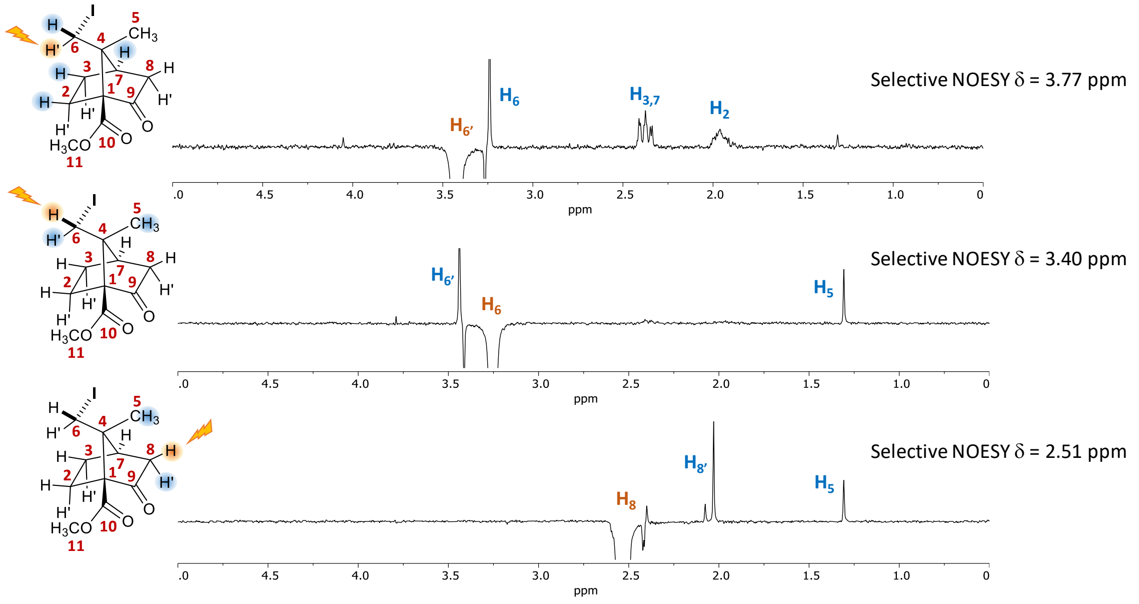

^{1}H-NMR of **19b-I** in $CDCl_3$

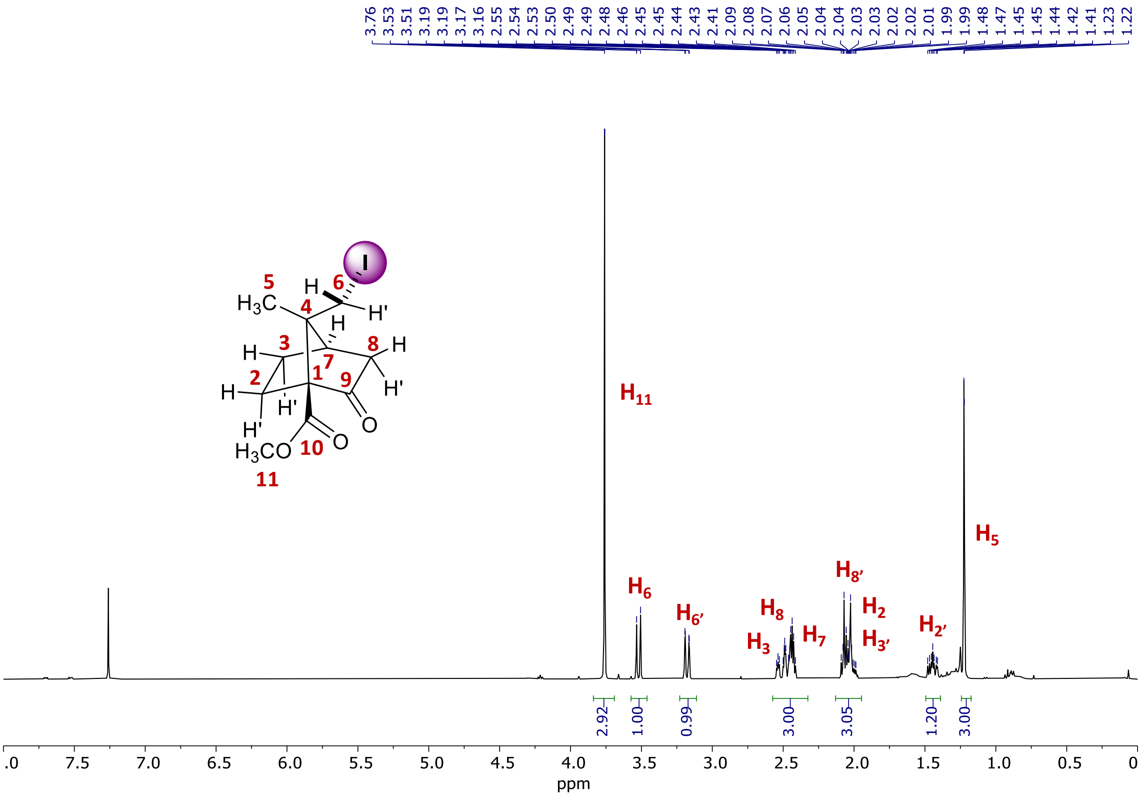

^{13}C-NMR of **19b-I** in $CDCl_3$

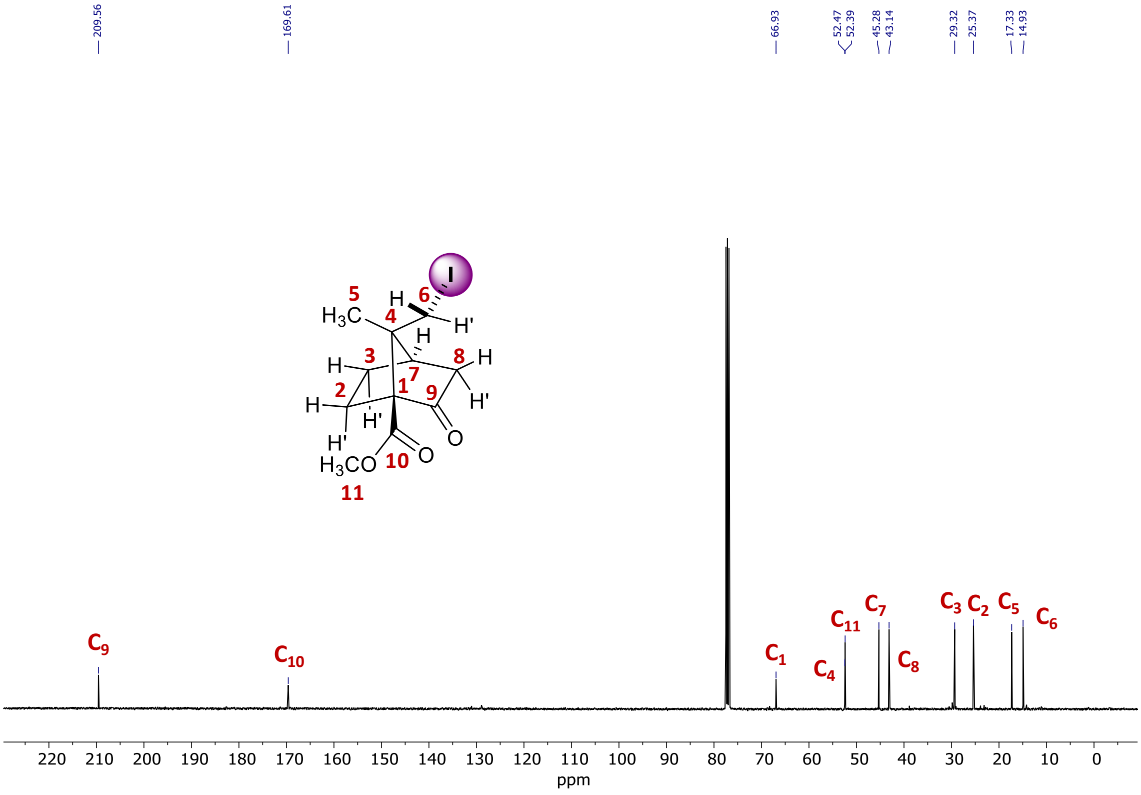

^{1}H-^{1}H COSY of **19b-I** in $CDCl_3$

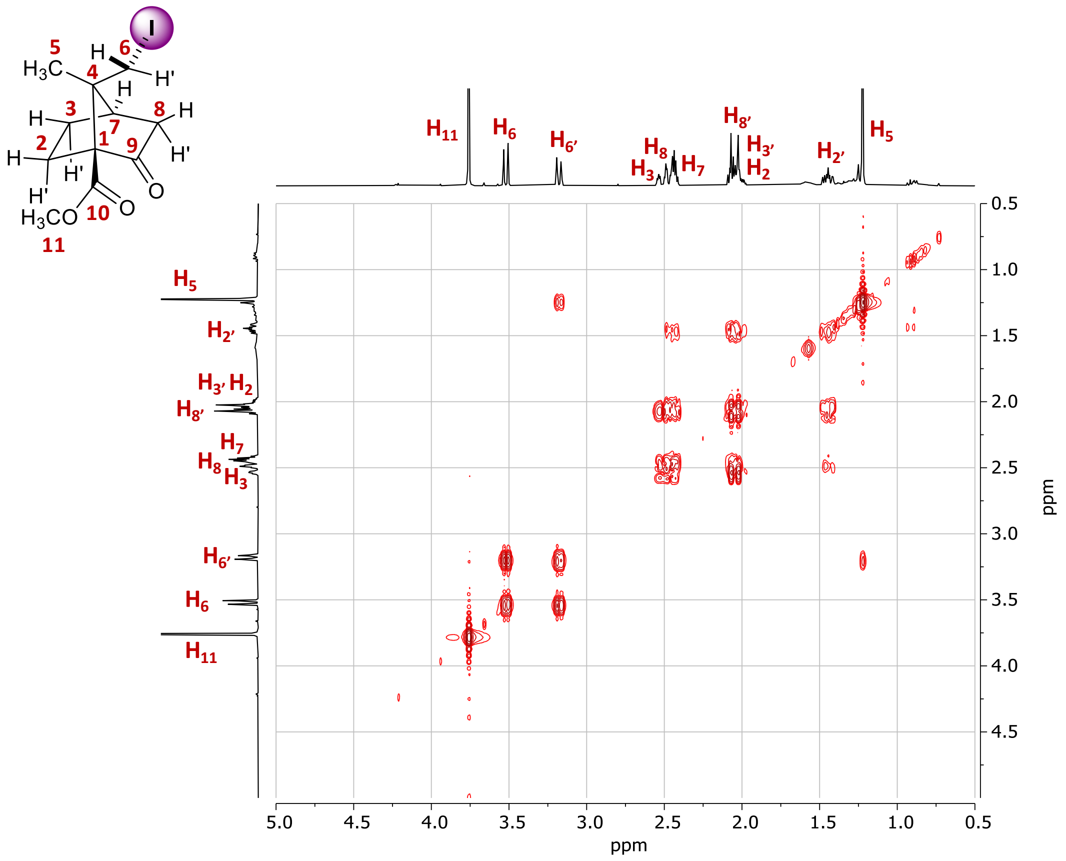

^{1}H-^{13}C HSQCed of **19b-I** in $CDCl_3$

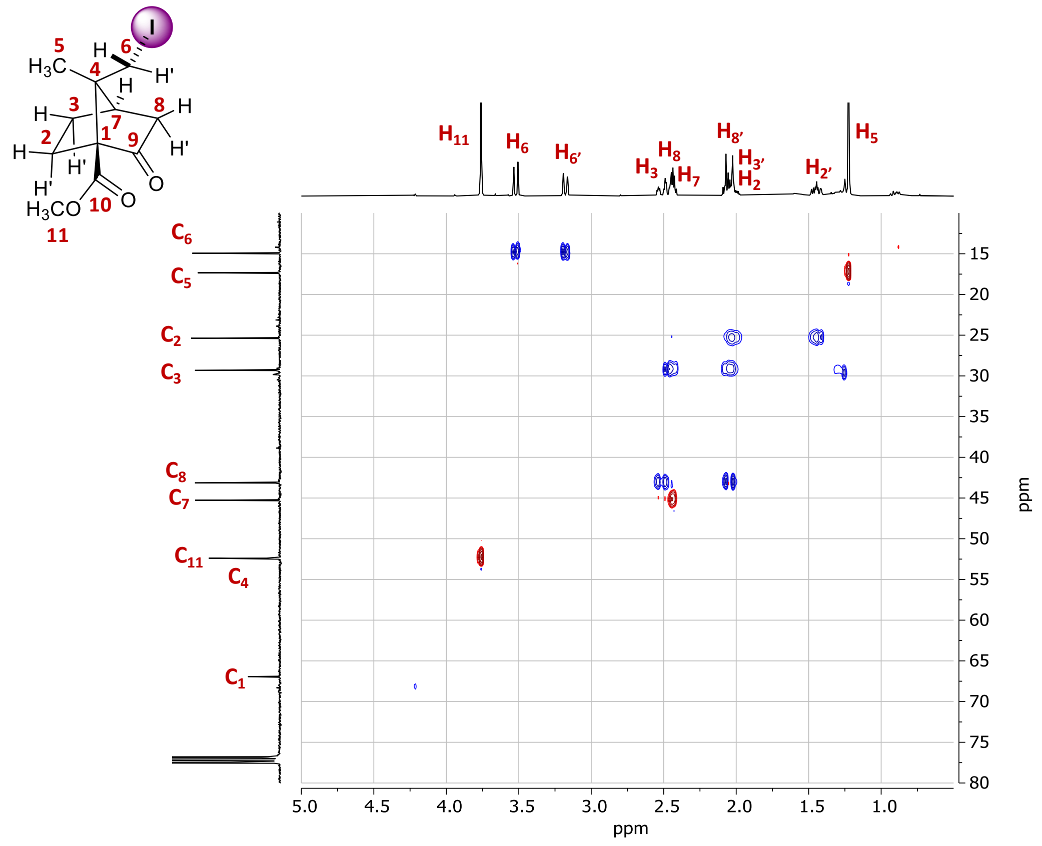

DEPTQ of **19b-I** in $CDCl_3$

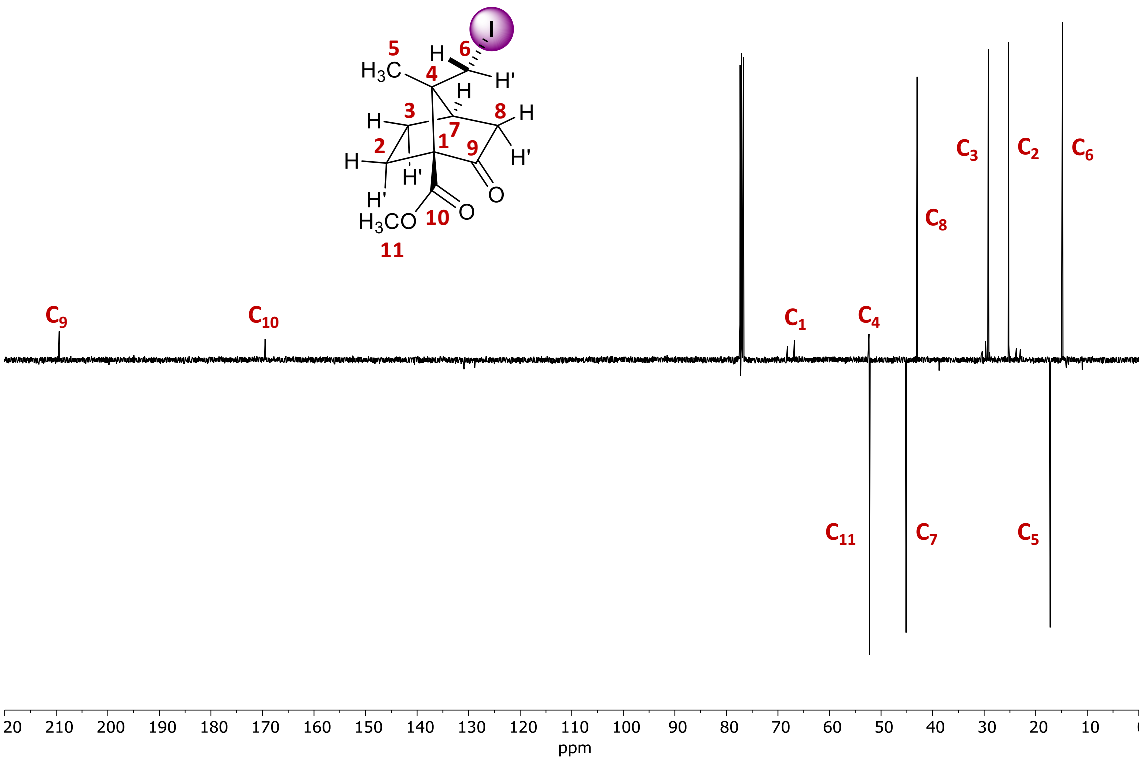

DEPT135 of **19b-I** in $CDCl_3$

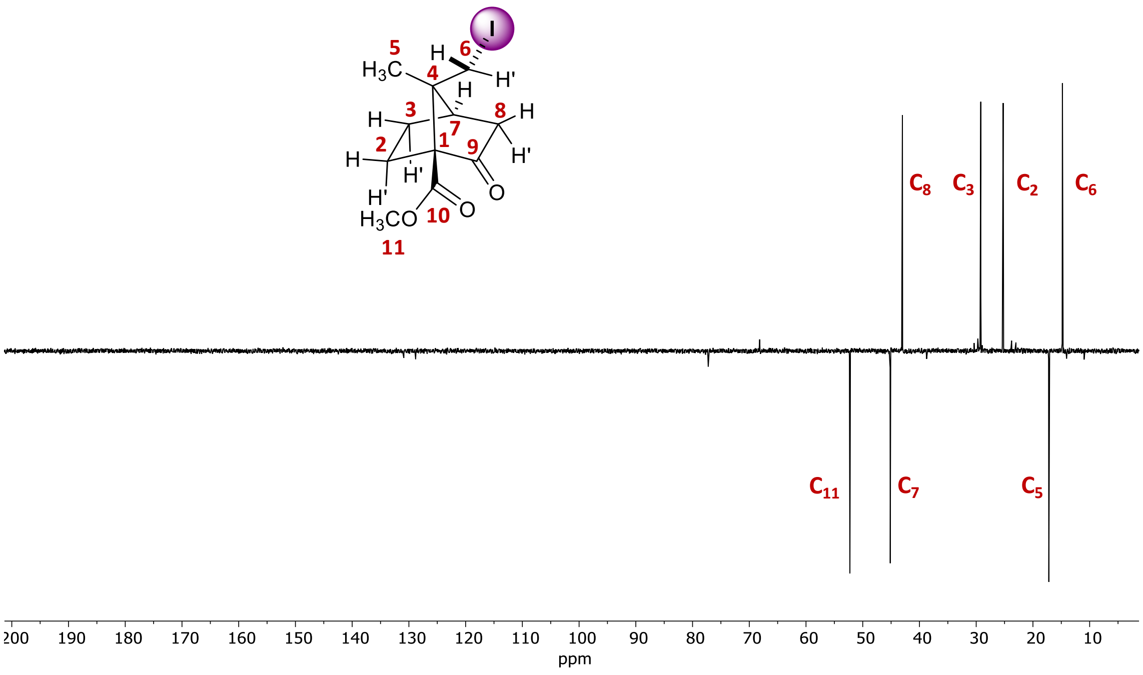

^{1}H-^{13}C HMBC of **19b-I** in $CDCl_3$

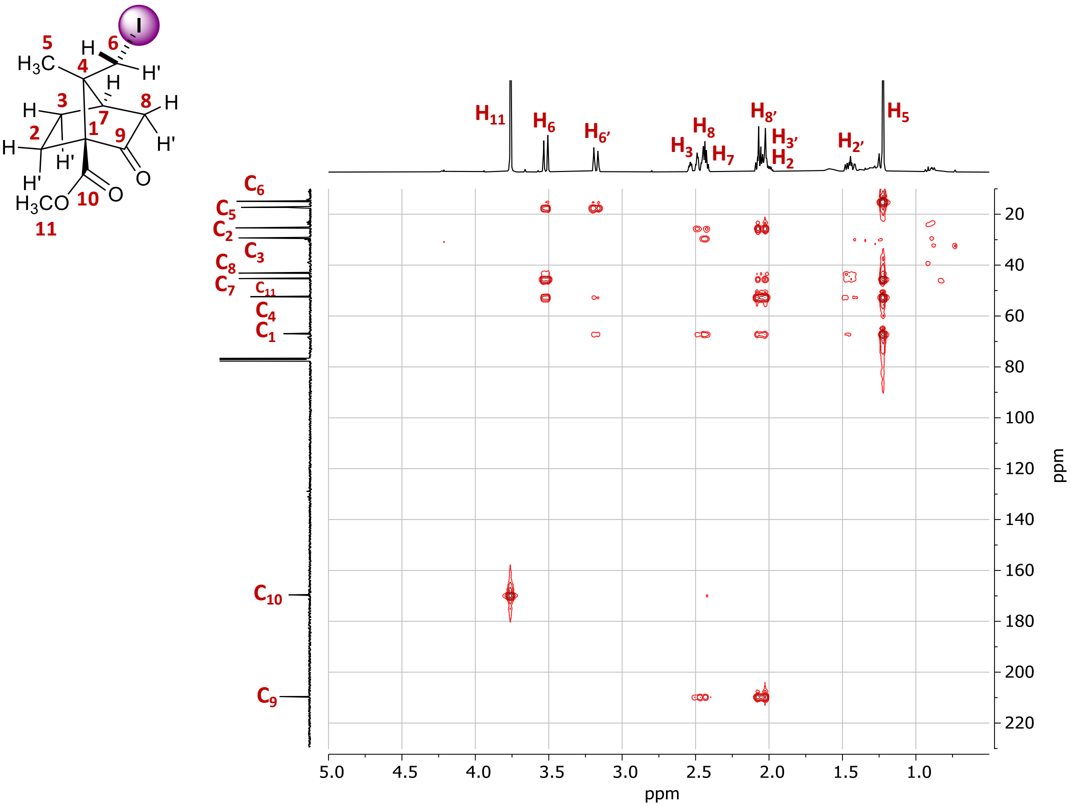

^{1}H-^{1}H TOCSY of **19b-I** in $CDCl_3$

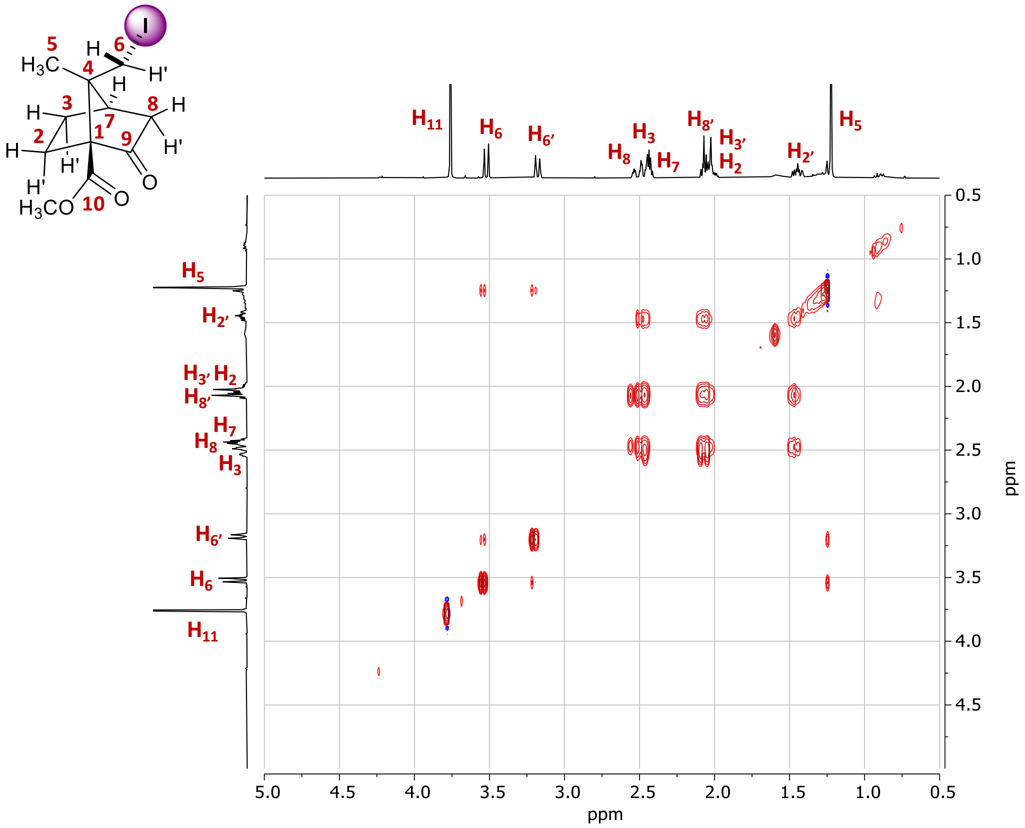

^1H-^1H NOESY of **19b-I** in $CDCl_3$

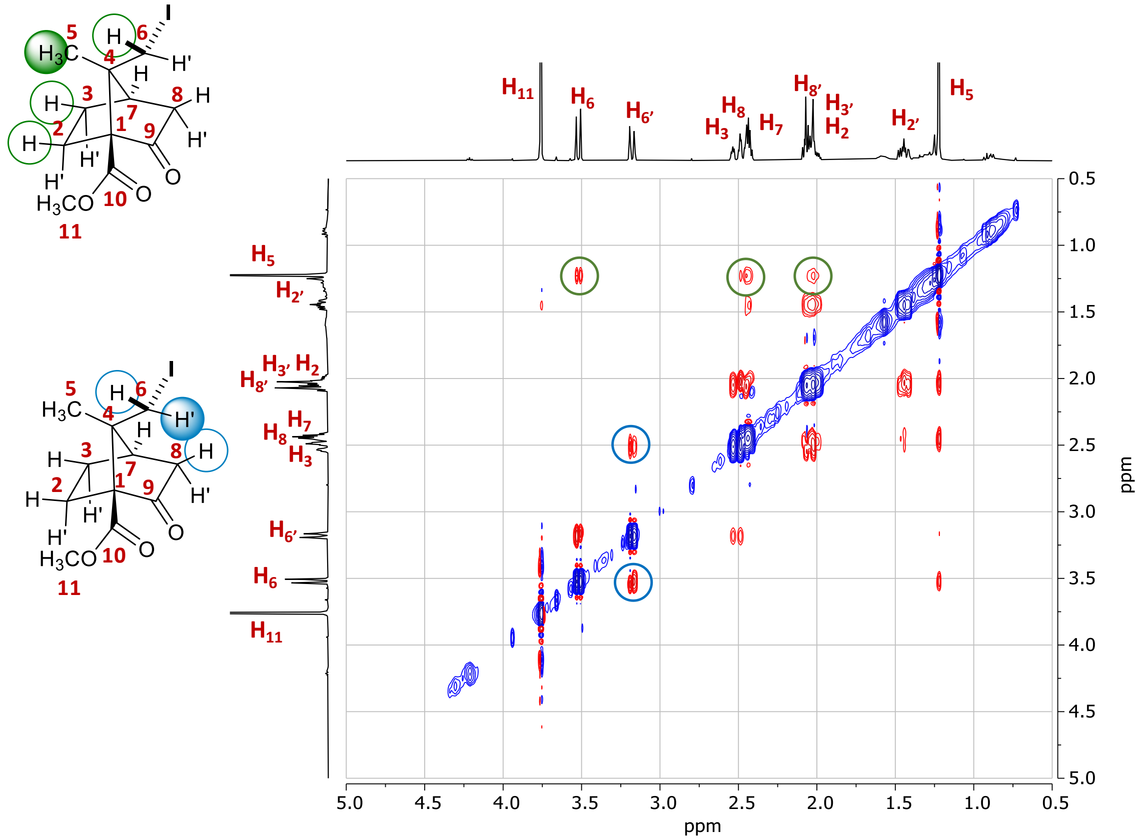

Selective NOESY experiments of **19b-I** in $CDCl_3$

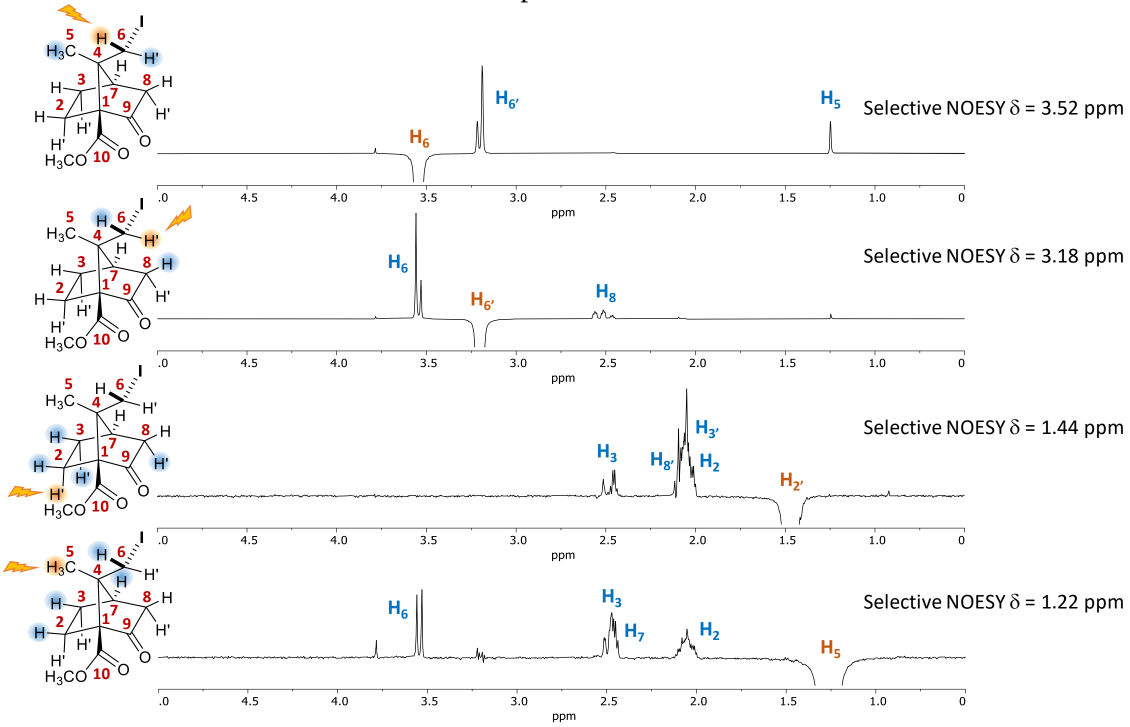

^{1}H-NMR of **19b-I^{COOH}** in $CDCl_3$

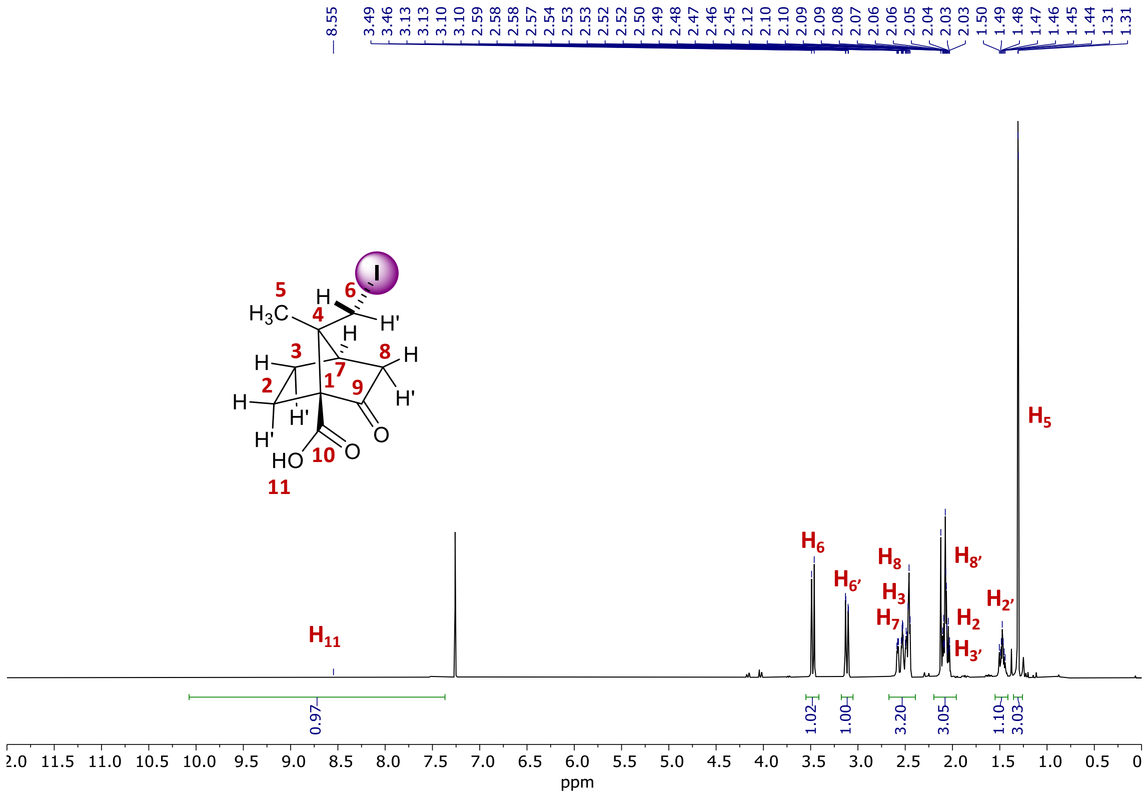

^{13}C-NMR of **19b-I^{COOH}** in $CDCl_3$

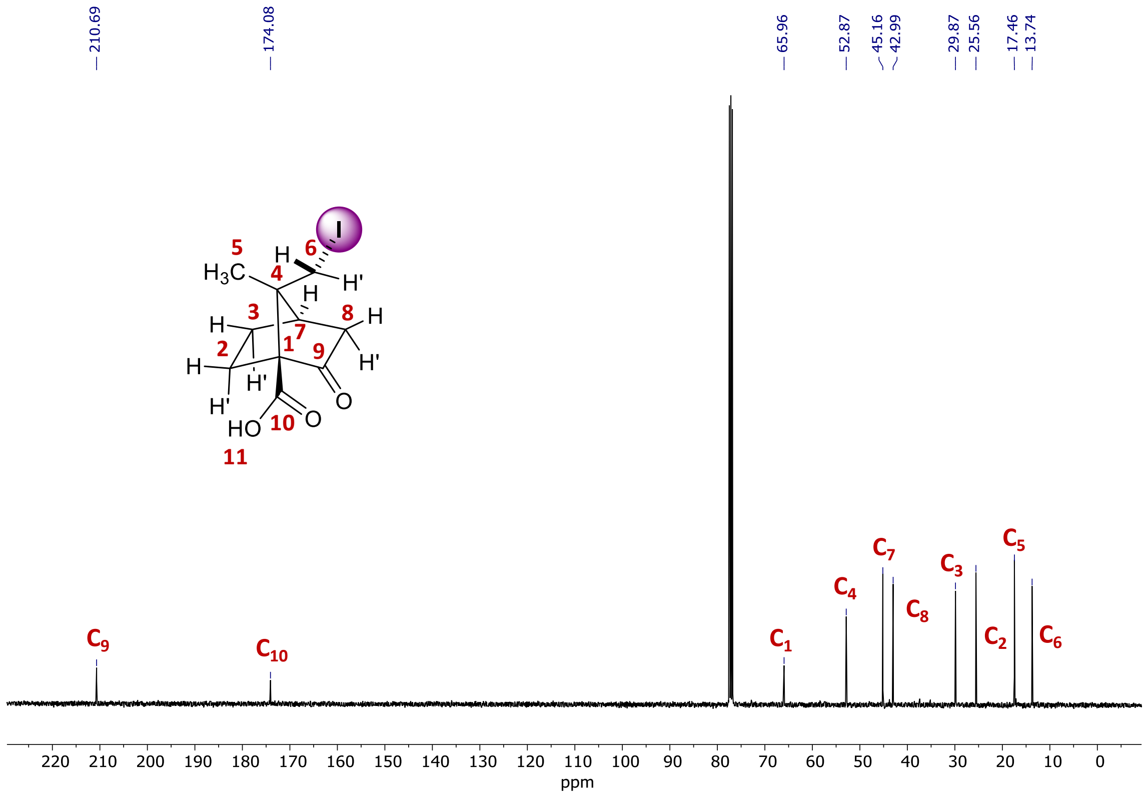

^{1}H-^{1}H COSY of **19b-I^COOH** in $CDCl_3$

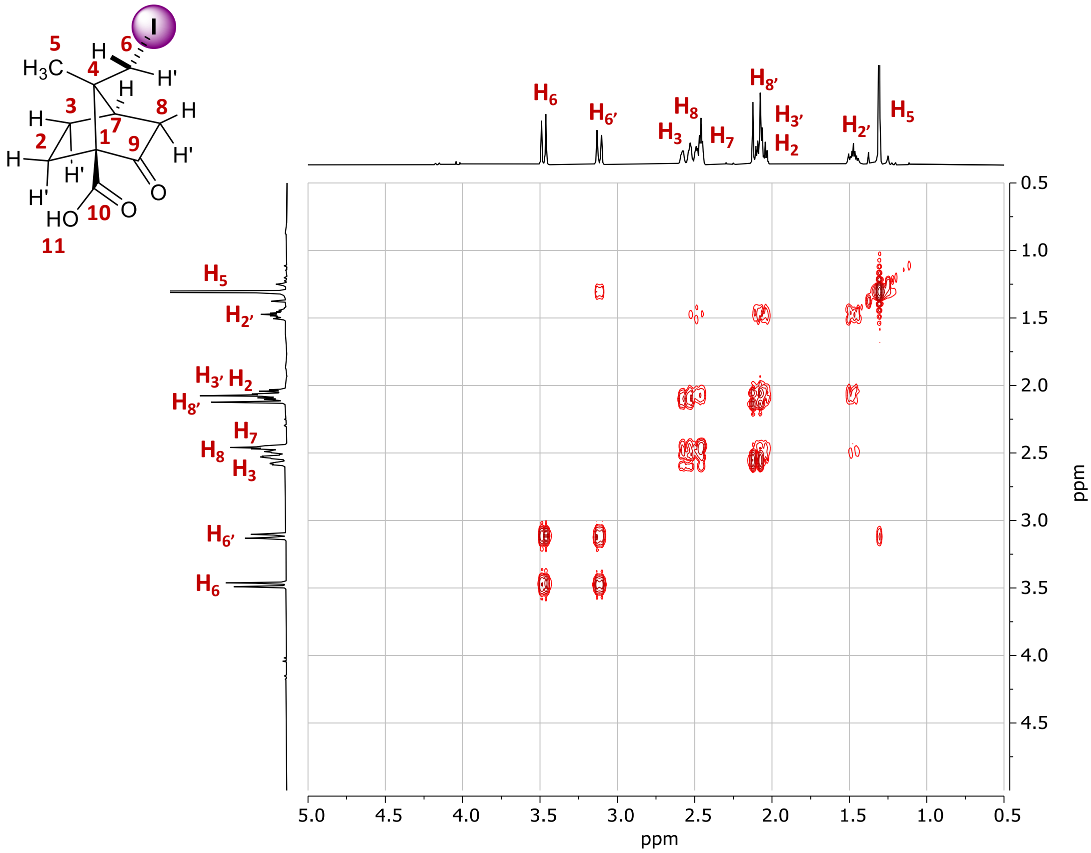

^{1}H-^{13}C HSQCed of **19b-I^COOH** in $CDCl_3$

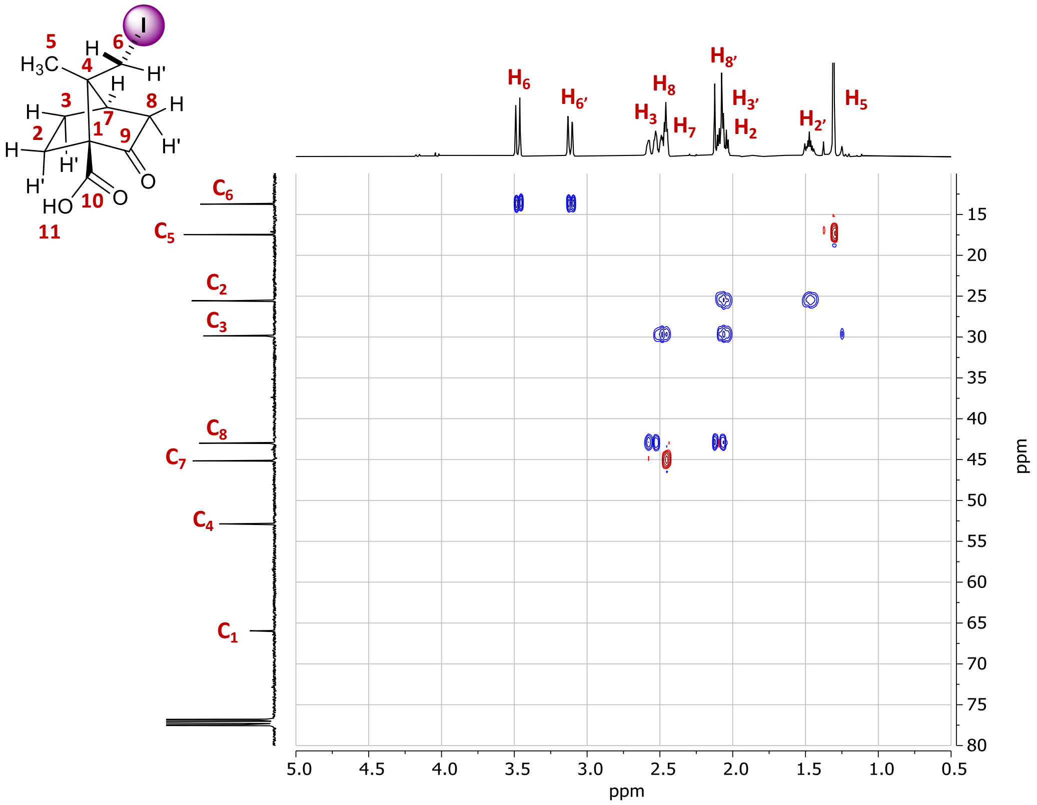

DEPTQ of **19b-I^{COOH}** in $CDCl_3$

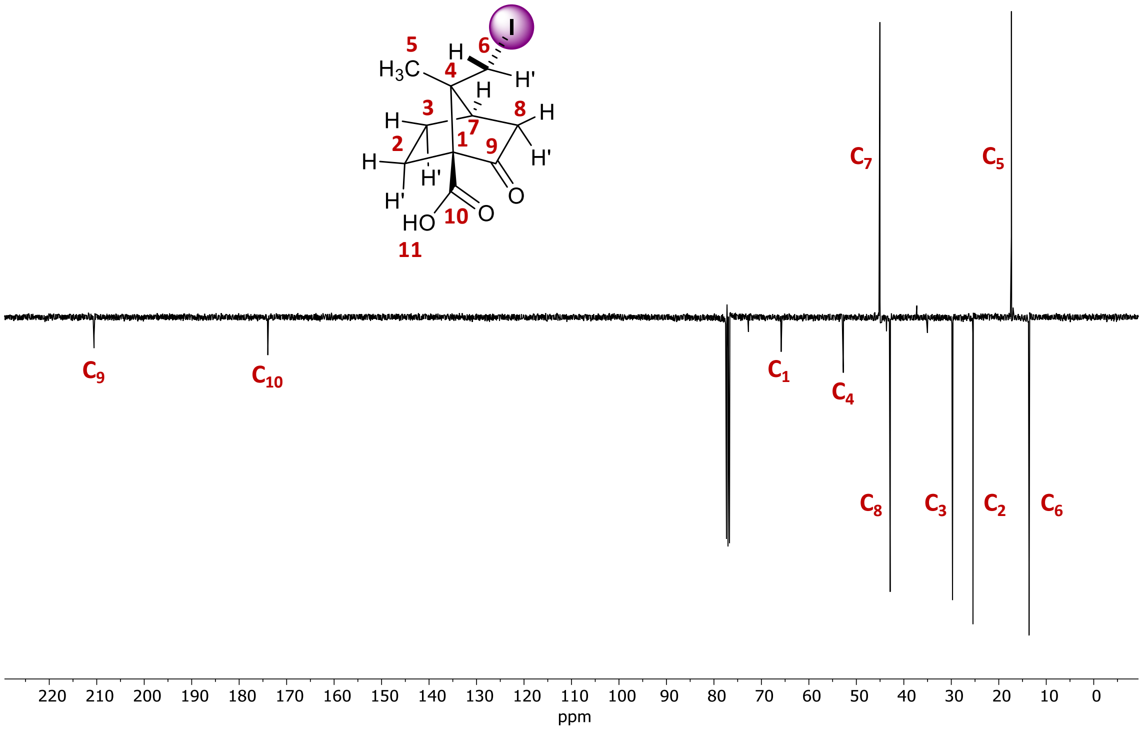

^1H-^{13}C HMBC of **19b-I^{COOH}** in $CDCl_3$

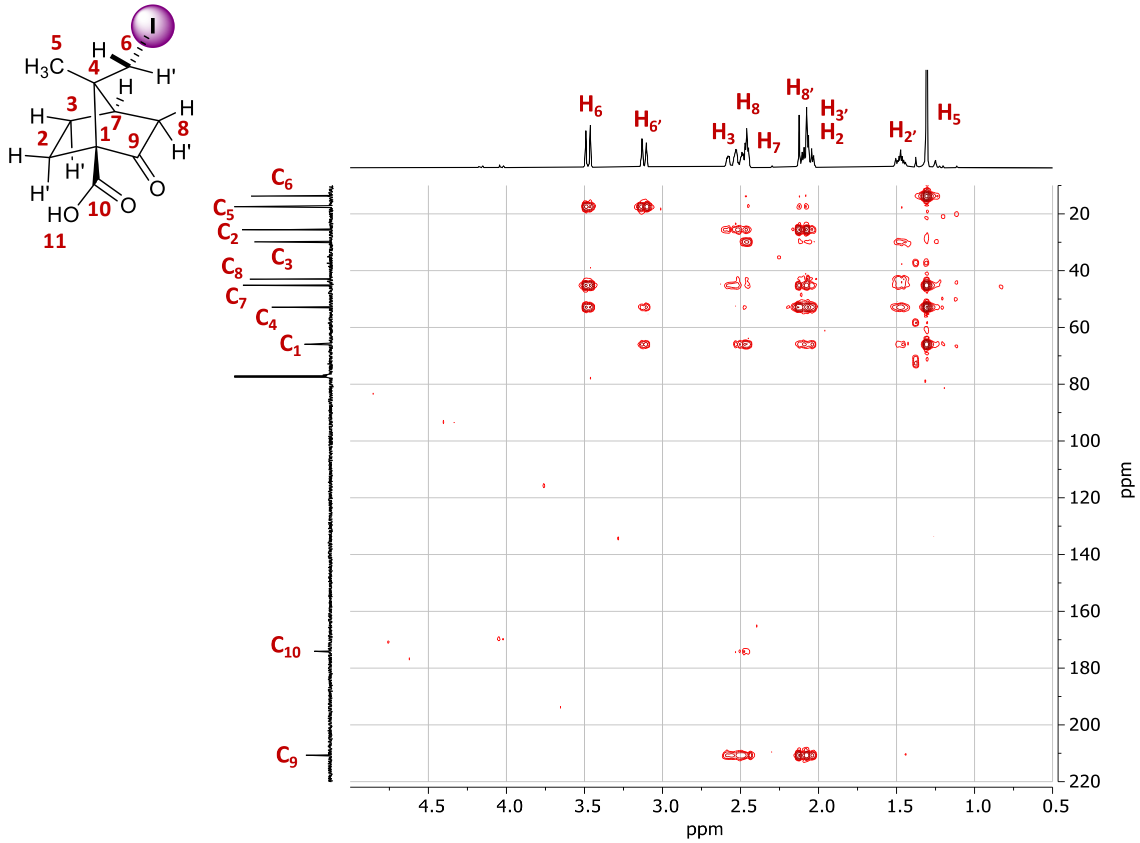

^{1}H-^{1}H TOCSY of **19b-I^{COOH}** in $CDCl_3$

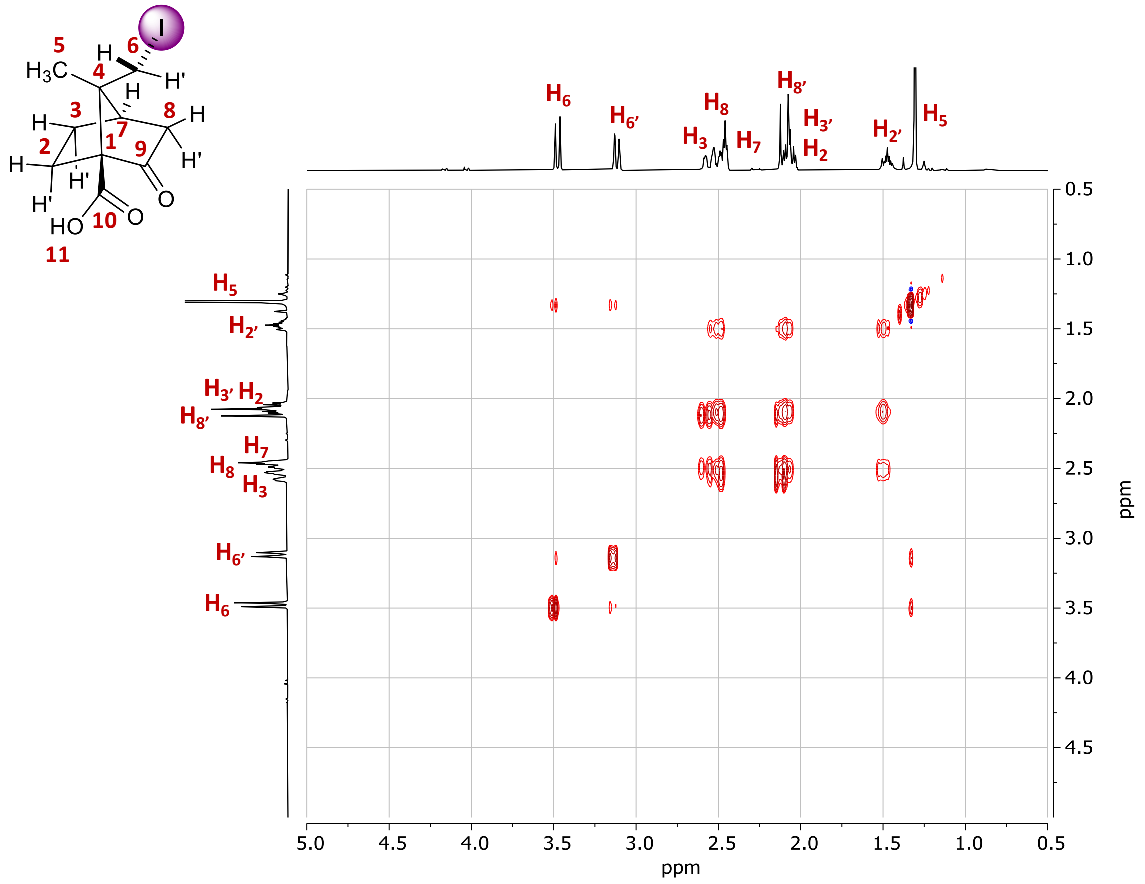

^{1}H-^{1}H NOESY of **19b-I^{COOH}** in $CDCl_3$

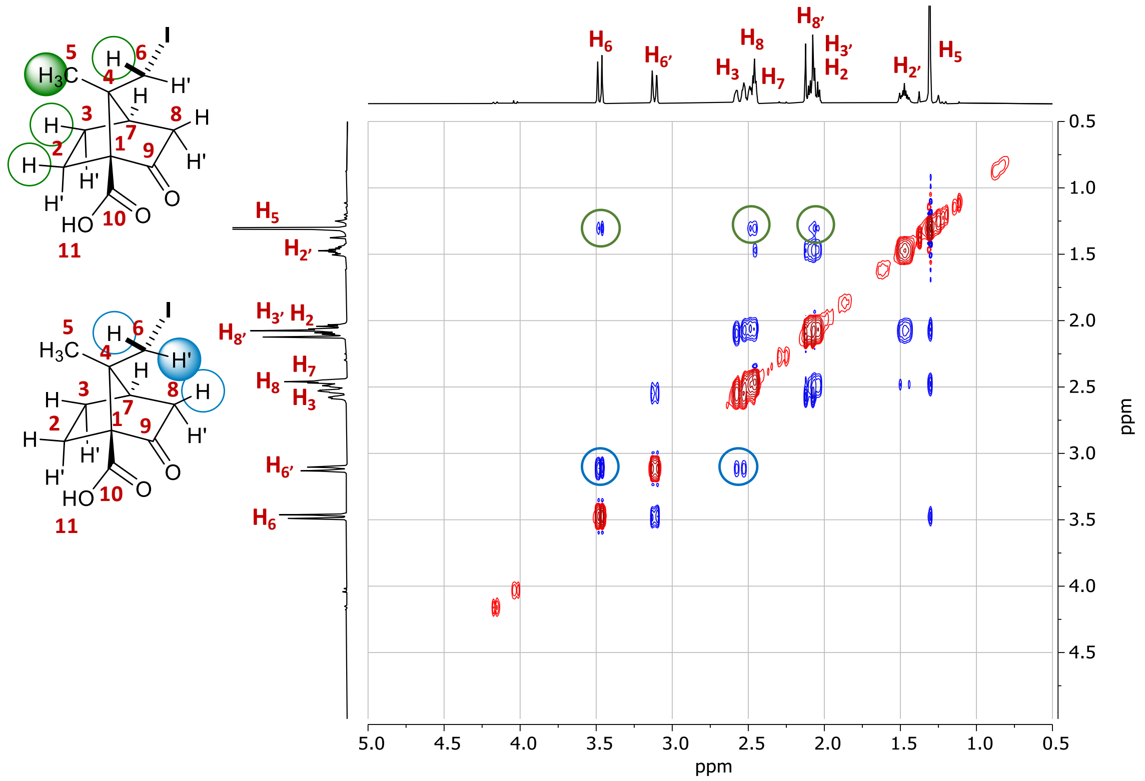

Selective NOESY experiments of **19b-I^{COOH}** in $CDCl_3$

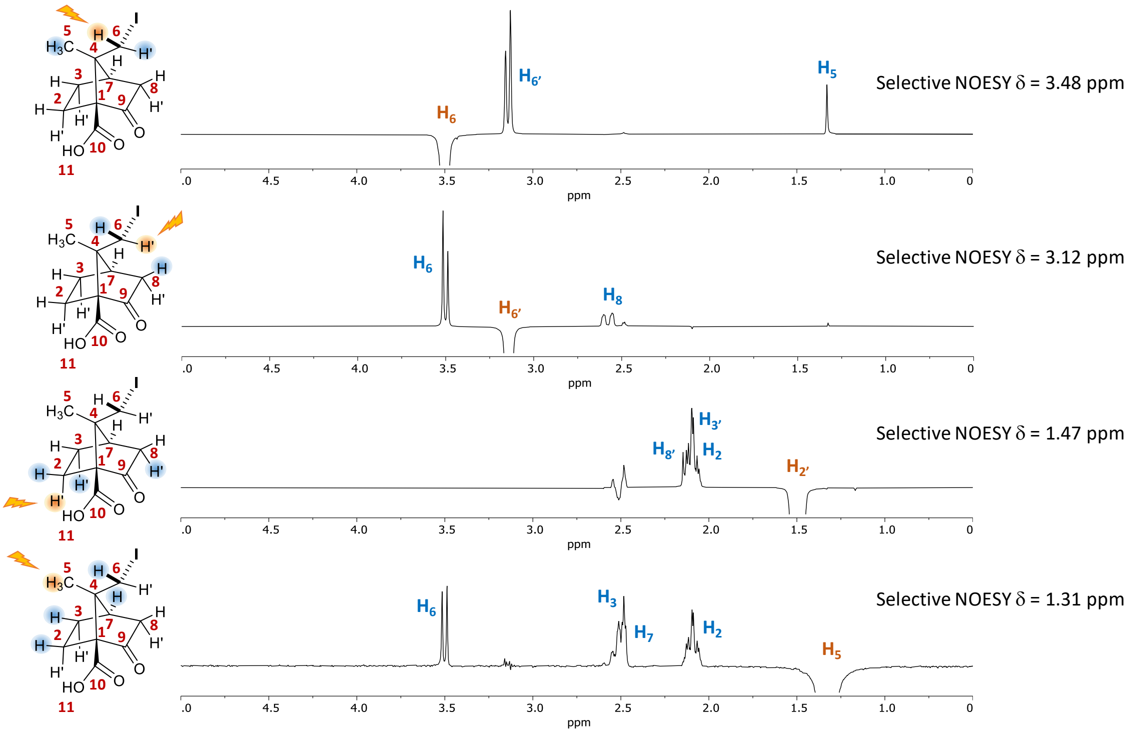

10. References

(1) Chen, M. S.; White, M. C. A Predictably Selective Aliphatic C–H Oxidation Reaction for Complex Molecule Synthesis. *Science* **2007**, *318*, 783-787.

(2) Ottenbacher, R. V.; Bryliakov, K. P.; Talsi, E. P. Non-Heme Manganese Complexes Catalyzed AsymmetricEpoxidation of Olefins by Peracetic Acid and Hydrogen Peroxide. *Adv. Synth. Catal.* **2011**, *353*, 885-889.

(3) Cussó, O.; Garcia-Bosch, I.; Font, D.; Ribas, X.; Lloret-Fillol, J.; Costas, M. Highly Stereoselective Epoxidation with H_2O_2 Catalyzed by Electron-Rich Aminopyridine Manganese Catalysts. *Org. Lett.* **2013**, *15*, 6158-6161.

(4) Cianfanelli, M.; Olivo, G.; Milan, M.; Klein Gebbink, R. J. M.; Ribas, X.; Bietti, M.; Costas, M. Enantioselective C-H Lactonization of Unactivated Methylenes Directed by Carboxylic Acids. *J. Am. Chem. Soc.* **2020**, *142*, 1584-1593.

(5) Milan, M.; Bietti, M.; Costas, M. Highly Enantioselective Oxidation of Nonactivated Aliphatic C-H Bonds with Hydrogen Peroxide Catalyzed by Manganese Complexes. *ACS Cent. Sci.* **2017**, *3*, 196-204.

(6) Vicens, L.; Bietti, M.; Costas, M. General Access to Modified α-Amino Acids by Bioinspired Stereoselective γ-C−H Bond Lactonization. *Angew. Chem. Int. Ed.* **2021**, *60*, 4740-4746.

(7) Skodje, R. T.; Truhlar, D. G. A General Small-Curvature Approximation for Transition-State-Theory Transmission Coefficients. *J. Phys. Chem.* **1981**, *85*, 3019–3023.

(8) E. Tiesinga, P. J. Mohr, D. B. Newell, B. N. Taylor "The 2018 CODATA Recommended Values of the Fundamental Physical Constants" (Web Version 8.0), 2019. Available at http://physics.nist.gov/constants, National Institute of Standards and Technology, Gaithersburg, MD 20899.

(9) (a) Johnson, E. R.; Keinan, S.; Mori-Sánchez, P.; Contreras-García, J.; Cohen, A. J.; Yang, W. Revealing Noncovalent Interactions. *J. Am. Chem. Soc.* **2010**, *132*, 6498-6506; (b) Contreras-Garcia, J.; Johnson, E. R.; Keinan, S.; Chaudret, R.; Piquemal, J. P.; Beratan, D. N.; Yang, W. NCIPLOT: a program for plotting non-covalent interaction regions. *J. Chem. Theory Comput.* **2011**, *7*, 625-632.

(10) Frisch, M. J.; Trucks, G. W.; Schlegel, H. B.; Scuseria, G. E.; Robb, M. A.; Cheeseman, J. R.; Scalmani, G.; Barone, V.; Petersson, G. A.; Nakatsuji, H.; Li, X.; Caricato, M.; Marenich, A. V.; Bloino, J.; Janesko, B. G.; Gomperts, R.; Mennucci, B.; Hratchian, H. P.; Ortiz, J. V.; Izmaylov, A. F.; Sonnenberg, J. L.; Williams; Ding, F.; Lipparini, F.; Egidi, F.; Goings, J.; Peng, B.; Petrone, A.; Henderson, T.; Ranasinghe, D.; Zakrzewski, V. G.; Gao, J.; Rega, N.; Zheng, G.; Liang, W.; Hada, M.; Ehara, M.; Toyota, K.; Fukuda, R.; Hasegawa, J.; Ishida, M.; Nakajima, T.; Honda, Y.; Kitao, O.; Nakai, H.; Vreven, T.; Throssell, K.; Montgomery Jr., J. A.; Peralta, J. E.; Ogliaro, F.; Bearpark, M. J.; Heyd, J. J.; Brothers, E. N.; Kudin, K. N.; Staroverov, V. N.; Keith, T. A.; Kobayashi, R.; Normand, J.; Raghavachari, K.; Rendell, A. P.; Burant, J. C.; Iyengar, S. S.; Tomasi, J.; Cossi, M.; Millam, J. M.; Klene, M.; Adamo, C.; Cammi, R.; Ochterski, J. W.; Martin, R. L.; Morokuma, K.; Farkas, O.; Foresman, J. B.; Fox, D. J. Wallingford, CT, 2016.

(11) (a) Becke, A. D. Density-functional thermochemistry. III. The role of exact exchange. *J. Chem. Phys.* **1993**, *98*, 5648-5652; (b) Lee, C.; Yang, W.; Parr, R. G. Development of the Colle-Salvetti correlation-energy formula into a functional of the electron density. *Phys. Rev. B* **1988**, *37*, 785-789.

(12) (a) Grimme, S.; Ehrlich, S.; Goerigk, L. Effect of the damping function in dispersion corrected density functional theory. *J. Comput. Chem.* **2011**, *32*, 1456-1465; (b) Grimme, S.; Antony, J.; Ehrlich, S.; Krieg, H. A consistent and accurate ab initio parametrization

of density functional dispersion correction (DFT-D) for the 94 elements H-Pu. *J. Chem. Phys.* **2010**, *132*, 154104; (c) Becke, A. D.; Johnson, E. R. Exchange-hole dipole moment and the dispersion interaction revisited. *J. Phys. Chem. B* **2007**, *127*, 154108.
(13) Marenich, A. V.; Cramer, C. J.; Truhlar, D. G. Universal Solvation Model Based on Solute Electron Density and on a Continuum Model of the Solvent Defined by the Bulk Dielectric Constant and Atomic Surface Tensions. *J. Phys. Chem. B* **2009**, *113*, 6378-6396.
(14) Schäfer, A.; Horn, H.; Ahlrichs, R. Fully optimized contracted Gaussian basis sets for atoms Li to Kr. *J. Chem. Phys.* **1992**, *97*, 2571-2577.
(15) Schäfer, A.; Huber, C.; Ahlrichs, R. Fully optimized contracted Gaussian basis sets of triple zeta valence quality for atoms Li to Kr. *J. Chem. Phys.* **1994**, *100*, 5829-5835.
(16) Richers, J.; Heilmann, M.; Drees, M.; Tiefenbacher, K. Synthesis of Lactones via C-H Functionalization of Nonactivated C(sp^3)-H Bonds. *Org. Lett.* **2016**, *18*, 6472-6475.
(17) Haynes, S. W.; Sydor, P. K.; Corre, C.; Song, L.; Challis, G. L. Stereochemical elucidation of streptorubin B. *J. Am. Chem. Soc.* **2011**, *133*, 1793-1798.
